# Design, synthesis and biological evaluation of novel triazoloquinazolinone and imidazoquinazolinone derivatives as allosteric inhibitors of SHP2 phosphatase

**DOI:** 10.1080/14756366.2022.2078968

**Published:** 2022-05-29

**Authors:** Wenjun Ye, Ye Liu, Qian Ren, Tianhui Liao, Yumei Chen, Dongmei Chen, Sisi Wang, Lihong Yao, Yihe Jia, Chunshen Zhao, Zhixu Zhou

**Affiliations:** a School of Pharmaceutical Sciences, Guizhou University, Guiyang, China;; b Guizhou Engineering Laboratory for Synthetic Drugs, Guiyang, China;; c Department of Medicinal Chemistry, China Pharmaceutical University, Nanjing, China

**Keywords:** SHP2, allosteric inhibitors, synthesis, antitumor activity

## Abstract

A series of novel triazoloquinolinone and imidazoquinazolinone derivatives were designed and synthesised, and their biological activities against SHP2 protein and melanoma A357 cell line were evaluated in vitro. The results show that some target compounds have moderate to excellent inhibitory activity on SHP2 protein and melanoma A357 cell line. Structure-activity relationships (SARs) showed that both imidazoquinazolinone and triazoloquinazolinone derivatives have good SHP2 protein kinase and melanoma cell line A357 inhibitory activity. The results of molecular docking also showed that the cores of imidazoquinazolinone and triazoloquinazolinone have a certain affinity for SHP2 protein at the same time. Compared with SHP244, the target compounds have quite good liver microsomal stability and has more drug potential. The most promising compound **B1** has a strong inhibitory effect on the melanoma cell line A357 at 100 µM (76.15% inhibition).

**ARTICLE HISTORY**
Received 8 February 2022
Revised 9 May 2022
Accepted 13 May 2022

## 1. Introduction

With the development of molecular biology science and technology, there are more and more tumour pathogenesis in terms of molecular level and infiltration, metastasis, growth and proliferation of blood vessels have been reported. Molecular targeted therapy of tumours has become more important and has incomparable advantages compared with traditional treatments[1–4]. Tumour molecular targeted therapy takes the peculiar changes of tumour cells as the target of action, and targets the specificity of cancer cells. While exerting anti-tumour activity, it reduces toxic and side effects to normal cells.

The drugs for molecular targeted therapy are mainly divided into three categories: monoclonal antibody drugs, signal transduction inhibitors and anti-angiogenesis drugs. Information transduction between cells can be achieved through direct contact between neighbouring cells, but more importantly and more commonly, the cells secrete various chemical substances to regulate the metabolism and functions of themselves and other cells. Among them, the initiation, proliferation and termination of signal cascades that control many normal cellular processes depend on the phosphorylation state of tyrosine in the signal protein, and are affected by protein tyrosine kinase (PTK, phosphorylation) and protein tyrosine. Regulation of acid phosphatase (PTP, dephosphorylation)[5–7]. Because they play an important role in signal transduction, the abnormal regulation of these enzymes often leads to the occurrence of numerous human diseases, including diabetes, cancer and autoimmune diseases[8]. SHP2 is the only proto-oncoprotein in the PTP family and is a good target for the treatment of cancer[9]. In the signalling pathways induced by a variety of growth factors, cytokines and extracellular matrix receptors, SHP2 is located at the common node downstream of RTK, so it participates in multiple intracellular oncogenic signal transduction cascades, such as JAK/STAT1, In the RAS/RAf/ERK, RAS/ERK/MAPK, and PD-1/PD-L1 signalling pathways, the abnormal expression of SHP2 can lead to dysregulation of multiple signalling pathways, thereby promoting the occurrence and development of cancer[10–15].

According to the structural characteristics of SHP2, it contains two SH2 domains and one PTP domain[16–19]. The two small molecule inhibitors of SHP2 that have been reported are divided into two categories: catalytic site and allosteric site inhibitors. The highly conserved sequence characteristics of the PTP catalytic site is therefore difficult to obtain highly selective PTP domain-targeted inhibitors, and the inhibitor binds to the region outside the PTP catalytic pocket, which shows that it is very important to other members of the phosphatase family with higher selectivity. Among the newly discovered two allosteric binding sites of SHP2, the "latch" site has a major breakthrough. The existing allosteric inhibitors at the "latch" site are all triazoloquinazolinone structures (Figure 1), and their druggability is poor[20–28]. Therefore, the research and development of SHP2 "latch" site allosteric inhibitors with novel structures and lower toxic and side effects has important scientific and practical significance.

In this paper, the interaction between the amino acid residues of compound **1** and SHP2 protein was investigated. On the basis of retaining the interaction between the amino acid residues of compound **1** and SHP2 protein and previous work, in order to

**CONTACT** Zhixu Zhou zhixuzhou@126.com School of Pharmaceutical Sciences, Guizhou University; Guizhou Engineering Laboratory for Synthetic Drugs, Guiyang 550025, China

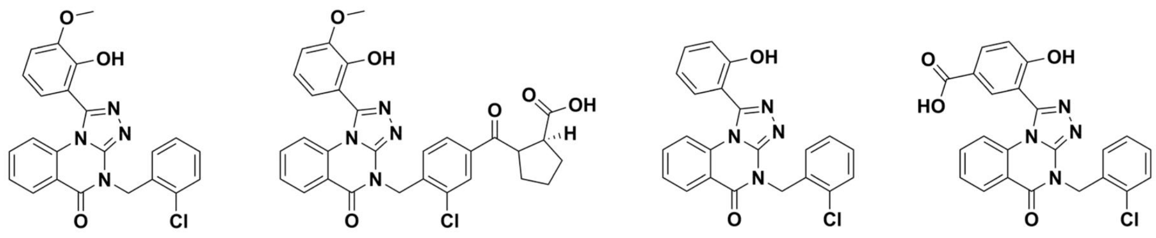

**Figure 1.** "Latch" site Inhibitors of SHP2.

further enrich the structure of the class of inhibitors, a number of published triazoloquinazolinones and imidazoquinazolinones compounds were designed and synthesized[29].

## 2. Experimental

### 2.1. Docking studies

To further clarify the binding mode of the compound, a docking analysis was carried out. The three-dimensional protein crystal structure of the SHP2 (PDB ID: 6BMR) was downloaded by the RCSB PDB protein database (https://www.rcsb.org/). The X-ray structure was prepared by MOE software to "Protonate 3D", delete water molecules and heteroligands or ions and adding the receptor surface, docked with **A35** and **A16** processed by "Minimize Energy" of ChemBio 3D[30,31]. The docking site is defined by the atoms of the ligand. The triangle placement method and the London dG application scoring function score the generation posture of the docked ligands. The best 5 poses for each compound were retained, and further analysed using the MOE tool "ligand interaction".

### 2.2. Chemistry

The melting point was obtained on the Microscopic Melting Point Tester X-4 (Beijing Taike, China) without correction. $^{1}$H NMR and $^{13}$C NMR spectra were recorded on a Bruker ARX-400, 400 MHz spectrometer (Bruker Bioscience, USA) with TMS as an internal control. Mass spectra were recorded in ESI pattern on an Agilent 1100 LC-MS device (Agilent Technologies, USA). Reactions' time and purity of the products were monitored by TLC on FLUKA silica gel aluminium cards (0.2 mm thickness) with Ultraviolet indicator 254 nm. Unless otherwise indicated, all materials were obtained from commercially available sources and were used without further purification.

### 2.3. General procedures

#### 2.3.1. General procedure for preparation of triazoloquinazolinones (6a–6e)

In a 500 mL single-neck flask, dissolve isatoic anhydride (50.00 g, 306.50 mmol) in 250 mL ethyl acetate, and add the corresponding arylmethyl primary amine (306.50 mmol) while stirring. After the addition, the reaction was heated to 40 °C for 2 h, and TLC monitored the completion of the reaction. The reaction solution was raised to room temperature, and the solvent ethyl acetate was evaporated under reduced pressure to obtain a white solid. The white solid was immersed in methanol, stirred, and filtered to remove insoluble impurities. Water was added to the filtrate for stirring, the solid was removed, filtered with suction, the filter cake was washed twice with petroleum ether, and filtered with suction to obtain the compounds ***6a–6e***.

#### 2.3.2. 2-Amino-N-(furan-2-ylmethyl)benzamide (6a)

White solid; yield: 92.4%; proceed to the next step without characterisation.

#### 2.3.3. 2-Amino-N-(thiophen-2-ylmethyl)benzamide (6b)

White solid; yield: 91.4%; proceed to the next step without characterisation.

#### 2.3.4. 2-Amino-N-(2-chlorobenzyl)benzamide (6c)

White solid; yield: 90.9%; proceed to the next step without characterisation.

#### 2.3.5. 2-Amino-N-(4-fluorobenzyl)benzamide (6d)

White solid; yield: 90.8%; proceed to the next step without characterisation.

#### 2.3.6. 2-Amino-N-(4-methoxybenzyl)benzamide (6e)

White solid; yield: 92.2%; proceed to the next step without characterisation.

#### 2.3.7. General procedure for preparation of triazoloquinazolinones (7a–7e)

In a 500 mL single-neck flask, dissolve compounds ***6a–6e***1 (191.78 mmol) in 250 mL of ethanol, add potassium hydroxide (23.67 g, 421.85 mmol) under stirring, and slowly add carbon disulphide (146.02 g, 1917.78 mmol) dropwise After the addition, the reaction was heated to 55 °C for 16 h, and TLC monitored the completion of the reaction. The reaction solution was brought to room temperature, a solid was precipitated, and then filtered with suction. The filter cake was washed once with water and once with acetone, and then filtered with suction to obtain the compounds ***7a–7e***.

#### 2.3.8. 3-(Furan-2-ylmethyl)-2-thioxo-2,3-dihydroquinazolin-4(1H)-one (7a)

White solid; yield: 85.8%; proceed to the next step without characterisation.

#### *2.3.9. 3-(Thiophen-2-ylmethyl)-2-thioxo-2,3-dihydroquinazolin-4(1H)-one (7b)*
White solid; yield: 91.42%; proceed to the next step without characterisation.

#### *2.3.10. 3–(2-Chlorobenzyl)-2-thioxo-2,3-dihydroquinazolin-4(1H)-one (7c)*
White solid; yield: 88.8%; proceed to the next step without characterisation.

#### *2.3.11. 3–(4-Fluorobenzyl)-2-thioxo-2,3-dihydroquinazolin-4(1H)-one (7d)*
White solid; yield: 88.0%; proceed to the next step without characterisation.

#### *2.3.12. 3–(4-Methoxybenzyl)-2-thioxo-2,3-dihydroquinazolin-4(1H)-one (7e)*
White solid; yield: 87.3%; proceed to the next step without characterisation.

#### *2.3.13. General procedure for preparation of triazoloquinazolinones (8a–8e)*
In a 250 mL single-neck flask, dissolve compounds ***7a–7e*** (33.03 mmol) in 100 mL of isopropanol, add hydrazine hydrate (31.00 g, 619.26 mmol) under stirring, and after the addition, heat the reaction to 90 °C Reaction for 16 h. TLC monitors the completion of the reaction, the reaction solution is brought to room temperature, a solid is precipitated, and suction filtration is performed. The filter cake is washed once with clear water and once with ethyl acetate, and compounds ***8a–8e*** is obtained by suction filtration.

#### *2.3.14. (E)-3-(furan-2-ylmethyl)-2-hydrazono-2,3-dihydroquinazolin-4(1H)-one (8a)*
White solid; yield: 87.7%; proceed to the next step without characterisation.

#### *2.3.15. (E)-2-hydrazono-3-(thiophen-2-ylmethyl)-2,3-dihydroquinazolin-4(1H)-one (8b)*
White solid; yield: 85.7%; proceed to the next step without characterisation.

#### *2.3.16. (E)-3–(2-chlorobenzyl)-2-hydrazono-2,3-dihydroquinazolin-4(1H)-one (8c)*
White solid; yield: 86.6%; proceed to the next step without characterisation

#### *2.3.17. (E)-3–(4-fluorobenzyl)-2-hydrazono-2,3-dihydroquinazolin-4(1H)-one (8d)*
White solid; yield: 81.6%; proceed to the next step without characterisation.

#### *2.3.18. (E)-2-hydrazono-3–(4-methoxybenzyl)-2,3-dihydroquinazolin-4(1H)-one (8e)*
White solid; yield: 85.6%; proceed to the next step without characterisation.

#### *2.3.19. General procedure for preparation of triazoloquinazolinones (9a–9e)*
In a 250 mL single-neck flask, dissolve compounds ***8a–8e*** (16.63 mmol) in 100 mL of *N,N*-dimethylformamide, and slowly add chloroacetyl chloride (2.82 g, 24.97 mmol) dropwise with stirring in an ice bath After the addition, react for half an hour in an ice bath, and then heat the reaction system to 100 °C for 5 h. TLC monitors the completion of the reaction. The reaction solution was cooled to room temperature, water was added to quench the reaction, the solid was stirred, and filtered with suction. The filter cake was washed twice with petroleum ether and twice with methyl *tert*-butyl ether, and the crude compounds ***9a-9e*** was produced by suction filtration.

#### *2.3.20. 1-(Chloromethyl)-4-(furan-2-ylmethyl)-[1,2,4]triazolo[4,3-a]quinazolin-5(4H)-one (9a)*
White solid; yield: 78.2%; proceed to the next step without characterisation.

#### *2.3.21. 1-(Chloromethyl)-4-(thiophen-2-ylmethyl)-[1,2,4]triazolo[4,3-a]quinazolin-5(4H)-one (9b)*
White solid; yield: 88.9%; proceed to the next step without characterisation.

#### *2.3.22. 4–(2-Chlorobenzyl)-1-(chloromethyl)-[1,2,4]triazolo[4,3-a]quinazolin-5(4H)-one (9c)*
White solid; yield: 87.1%; proceed to the next step without characterisation.

#### *2.3.23. 1-(Chloromethyl)-4–(4-fluorobenzyl)-[1,2,4]triazolo[4,3-a]quinazolin-5(4H)-one (9d)*
White solid; yield: 86.7%; proceed to the next step without characterisation.

#### *2.3.24. 1-(Chloromethyl)-4–(4-methoxybenzyl)-[1,2,4]triazolo[4,3-a]quinazolin-5(4H)-one (9e)*
White solid; yield: 77.1%; proceed to the next step without characterisation.

#### *2.3.25. General procedure for the preparation of triazoloquinazolinone derivatives (A1-A28)*
In a 100 mL single-neck flask, dissolve compounds ***9a-9e*** (0.56 mmol) in 20 mL of acetonitrile, add potassium carbonate (0.23 g, 1.67 mmol) and the corresponding nitrogen-containing reagent (0.69 mmol) while stirring, and add Afterwards, the reaction system was heated to 50 °C for 4 h, and TLC monitored the completion of the reaction. The reaction solution was cooled to room temperature, water was added to quench the reaction, the solid was stirred out, filtered with suction, and the filter cake was washed twice with acetone to obtain pure compounds ***A1-A28***.

### 2.3.26. 4-(furan-2-ylmethyl)-1-(piperidin-1-ylmethyl)-[1,2,4]triazolo [4,3-a]quinazolin -5(4H)-one (A1)

White solid; yield: 65.2%; *m.p.*: 2 0 0 ~ 202 °C; $^1$H NMR (400 MHz, $CDCl_3$) $\delta$ (ppm) = 8.43 (d, $J$ = 7.8 Hz), 8.39 (d, $J$ = 8.4 Hz), 7.80 (t, $J$ = 7.4 Hz), 7.54 (t, $J$ = 7.6 Hz), 7.35 (s), 6.60 (d, $J$ = 2.7 Hz), 6.31 (s), 5.57 (s), 3.94 (s), 2.56 (s), 1.61 (s), 1.56 (s); HRMS(ESI)$(M+H)^+$, m/z: calcd for $C_{20}H_{21}N_5O_2$ 363.1695, found 364.1766.

### 2.3.27. 4-(Furan-2-ylmethyl)-1-(((4-methoxybenzyl)amino)methyl)-[1,2,4]triazolo[4,3-a]quinazolin-5(4H)-one (A2)

White solid; yield: 59.3%; *m.p.*: 1 5 2 ~ 154 °C; $^1$H NMR (400 MHz, $CDCl_3$) $\delta$ (ppm) = 8.42 (d, $J$ = 7.7 Hz), 8.06 (d, $J$ = 8.4 Hz), 7.71 (t, $J$ = 7.7 Hz), 7.52 (t, $J$ = 7.6 Hz), 7.28 (s), 7.26 (s), 6.87 (d, $J$ = 8.3 Hz), 6.57 (s), 6.30 (s), 5.55 (s), 4.28 (s), 3.90 (s), 3.80 (s), 1.85 (s); HRMS(ESI)$(M+H)^+$, m/z: calcd for $C_{23}H_{21}N_5O_3$ 415.1644, found 416.1718.

### 2.3.28. 4-(Furan-2-ylmethyl)-1-((4-hydroxypiperidin-1-yl)methyl)-[1,2,4] triazolo[4,3-a]quinazolin-5(4H)-one (A3)

White solid; yield: 70.8%; *m.p.*: 2 2 8 ~ 230 °C; $^1$H NMR (400 MHz, $CDCl_3$) $\delta$ (ppm) = 8.45 (d, $J$ = 7.8 Hz, 1H), 8.31 (d, $J$ = 8.4 Hz, 1H), 7.80 (t, $J$ = 7.6 Hz, 1H), 7.55 (t, $J$ = 7.6 Hz, 1H), 7.35 (s, 1H), 6.60 (d, $J$ = 2.8 Hz, 1H), 6.31 (s, 1H), 5.57 (s, 2H), 4.00 (s, 2H), 3.77 (s, 1H), 2.94 – 2.86 (m, 2H), 2.40 (t, $J$ = 10.1 Hz, 2H), 1.91 (d, $J$ = 14.0 Hz, 2H), 1.54 (s, 1H), 1.42 (d, $J$ = 3.9 Hz, 1H); HRMS(ESI)$(M+H)^+$, m/z: calcd for $C_{20}H_{21}N_5O_3$ 379.1644, found 380.1718.

### 2.3.29. 4-(Furan-2-ylmethyl)-1-((4-methylpiperidin-1-yl)methyl)-[1,2,4] triazolo[4,3-a]quinazolin-5(4H)-one (A4)

White solid; yield: 66.7%; *m.p.*: 2 3 0 ~ 232 °C; $^1$H NMR (400 MHz, $CDCl_3$) $\delta$ (ppm) = 8.44 (d, $J$ = 10.3 Hz), 8.37 (d, $J$ = 8.8 Hz), 7.83 – 7.78 (m), 7.56 – 7.52 (m), 7.35 (s), 6.60 (d, $J$ = 2.9 Hz), 6.31 (s), 5.57 (s), 3.95 (s), 2.91 (d, $J$ = 11.5 Hz), 2.23 (t, $J$ = 11.7 Hz), 1.64 (d, $J$ = 14.8 Hz), 1.25 (s), 1.15 (d, $J$ = 12.3 Hz), 0.90 (d, $J$ = 6.3 Hz); HRMS(ESI)$(M+H)^+$, m/z: calcd for $C_{22}H_{23}ClN_5O_2$ 377.1852, found 378.1923.

### 2.3.30. 4-(Furan-2-ylmethyl)-1-((3-methylpiperidin-1-yl)methyl)-[1,2,4] triazolo[4,3-a]quinazolin-5(4H)-one (A5)

White solid; yield: 66.7%; *m.p.*: 2 1 5 ~ 217 °C; $^1$H NMR (400 MHz, $CDCl_3$) $\delta$ (ppm) = 8.43 (d, $J$ = 7.9 Hz, 1H), 8.39 (d, $J$ = 8.4 Hz, 1H), 7.79 (t, $J$ = 7.8 Hz, 1H), 7.53 (t, $J$ = 7.6 Hz, 1H), 7.35 (s, 1H), 6.60 (s, 1H), 6.31 (s, 1H), 5.57 (s, 2H), 3.94 (s, 2H), 2.83 (s, 2H), 2.19 (t, $J$ = 10.4 Hz, 1H), 1.90 (t, $J$ = 10.4 Hz, 1H), 1.68 (d, $J$ = 10.0 Hz, 2H), 1.54 – 1.42 (m, 1H), 1.02 – 0.91 (m, 1H), 0.87 (d, $J$ = 6.5 Hz, 3H); HRMS(ESI)$(M+H)^+$, m/z: calcd for $C_{22}H_{23}ClN_5O_2$ 377.1852, found 378.1924.

### 2.3.31. 4-(Furan-2-ylmethyl)-1-((2-methylpiperidin-1-yl)methyl)-[1,2,4] triazolo[4,3-a]quinazolin-5(4H)-one (A6)

White solid; yiled: 66.7%; *m.p.*: 2 2 4 ~ 226 °C; $^1$H NMR (400 MHz, $CDCl_3$) $\delta$ (ppm) = 8.73 (d, $J$ = 8.3 Hz, 1H), 8.43 (d, $J$ = 7.8 Hz, 1H), 7.79 (t, $J$ = 7.8 Hz, 1H), 7.53 (t, $J$ = 7.6 Hz, 1H), 7.35 (s, 1H), 6.60 (s, 1H), 6.31 (s, 1H), 5.56 (s, 2H), 4.33 (d, $J$ = 13.8 Hz, 1H), 3.89 (d, $J$ = 13.7 Hz, 1H), 2.66 (s, 2H), 2.30 (s, 1H), 1.71 (s, 2H), 1.53 – 1.37 (m, 4H), 1.26 (d, $J$ = 6.3 Hz, 3H); HRMS(ESI)(M + H)+, m/z: calcd for $C_{22}H_{23}ClN_5O_2$ 377.1852, found 378.1925.

### 2.3.32. 1-(Morpholinomethyl)-4-(thiophen-2-ylmethyl)-[1,2,4]triazolo [4,3-a] quinazolin-5(4H)-one (A7)

White solid; yield: 65.2%; *m.p.*: 2 1 4 ~ 216 °C; $^1$H NMR (400 MHz, DMSO-$d_6$) $\delta$ 8.29 (d, $J$ = 7.9 Hz, 1H), 8.24 (d, $J$ = 8.3 Hz, 1H), 7.97 (t, $J$ = 7.7 Hz, 1H), 7.63 (t, $J$ = 7.4 Hz, 1H), 7.43 (d, $J$ = 4.7 Hz, 1H), 7.25 (s, 1H), 6.97 (s, 1H), 5.53 (s, 2H), 4.01 (s, 2H), 3.54 (s, 4H), 2.51 (s, 4H); HRMS(ESI)$(M+H)^+$, m/z: calcd for $C_{19}H_{19}N_5O_2S$ 381.1259, found 382.1329.

### 2.3.33. 1-((1 h-pyrazol-1-yl)methyl)-4-(thiophen-2-ylmethyl)-[1,2,4] triazolo[4,3-a] quinazolin-5(4H)-one (A8)

White solid; yield: 80.5%; *m.p.*: 2 7 8 ~ 280 °C; $^1$H NMR (400 MHz, DMSO-$d_6$) $\delta$ 8.32 (d, $J$ = 8.0 Hz, 1H), 7.88 (d, $J$ = 9.2 Hz, 2H), 7.64 (s, 1H), 7.48 (s, 2H), 7.30 (s, 1H), 7.01 (s, 1H), 6.34 (s, 1H), 6.08 (s, 1H), 5.59 (s, 2H). HRMS(ESI)$(M+H)^+$, m/z: calcd for $C_{18}H_{14}N_6OS$ 362.0950, found 363.1016.

### 2.3.34. 4–(2-Chlorobenzyl)-1–(3-((diethylamino)methyl)-4-hydroxy-5-methoxyphenyl)-[1,2,4]triazolo[4,3-a]quinazolin-5(4H)-one (A9)

White solid; yield: 62.5%; *m.p.*: 2 3 9 ~ 241 °C; $^1$H NMR (400 MHz, DMSO-$d_6$) $\delta$ 8.28 (d, $J$ = 7.8 Hz, 1H), 8.23 (d, $J$ = 8.4 Hz, 1H), 7.96 (t, $J$ = 7.8 Hz, 1H), 7.62 (t, $J$ = 7.5 Hz, 1H), 7.43 (d, $J$ = 4.8 Hz, 1H), 7.25 (s, 1H), 6.97 (s, 1H), 5.53 (s, 2H), 3.99 (s, 2H), 2.59 (d, $J$ = 61.8 Hz, 4H), 2.28 (s, 4H), 2.12 (s, 3H); HRMS(ESI)$(M+H)^+$, m/z: calcd for $C_{20}H_{22}N_6OS$ 394.1576, found 395.1645.

### 2.3.35. 4–(2-Chlorobenzyl)-1–(4-hydroxy-3-methoxy-5-(pyrrolidin-1-ylmethyl)phenyl)-[1,2,4] triazolo[4,3-a]quinazolin-5(4H)-one (A10)

White solid; yield: 63.6%; *m.p.*: 2 5 0 ~ 252 °C; $^1$H NMR (400 MHz, DMSO-$d_6$) $\delta$ 8.30 – 8.24 (m, 2H), 7.96 (t, $J$ = 7.8 Hz, 1H), 7.62 (t, $J$ = 7.6 Hz, 1H), 7.43 (d, $J$ = 5.0 Hz, 1H), 7.25 (s, 1H), 6.99 – 6.95 (m, 1H), 5.53 (s, 2H), 3.94 (s, 2H), 2.48 (s, 4H), 1.46 (s, 4H), 1.40 (s, 2H); HRMS(ESI)$(M+H)^+$, m/z: calcd for $C_{20}H_{21}N_5OS$ 379.1467, found 380.1532.

### 2.3.36. 4–(2-Chlorobenzyl)-1-(morpholinomethyl)-[1,2,4]triazolo [4,3-a]quinazolin-5(4H)-one (A11)

White solid; yield: 65.2%; *m.p.*: 2 1 3 ~ 215 °C; $^1$H NMR (400 MHz, DMSO-$d_6$) $\delta$ 8.31 (d, $J$ = 8.4 Hz, 1H), 8.28 (d, $J$ = 7.9 Hz, 1H), 8.01 (t, $J$ = 7.4 Hz, 1H), 7.65 (t, $J$ = 7.5 Hz, 1H), 7.53 (d, $J$ = 7.9 Hz, 1H), 7.31 (t, $J$ = 7.1 Hz, 1H), 7.20 (d, $J$ = 6.9 Hz, 2H), 5.42 (s, 2H), 4.02 (s, 2H), 3.57 (s, 4H), 2.54 (s, 4H); HRMS(ESI)$(M+H)^+$, m/z: calcd for $C21H_{20}ClN_5O_2$ 409.1306, found 410.1368.

### 2.3.37. 4–(4-Fluorobenzyl)-1-(morpholinomethyl)-[1,2,4]triazolo [4,3-a] quinazolin-5(4H)-one (A12)

White solid; yield: 65.2%; *m.p.*: 2 4 5 ~ 247 °C; $^1$H NMR (400 MHz, DMSO-$d_6$) $\delta$ 8.27 (d, $J$ = 4.6 Hz, 2H), 7.97 (t, $J$ = 7.7 Hz, 1H), 7.62 (t, $J$ = 7.5 Hz, 1H), 7.56 – 7.49 (m, 2H), 7.14 (t, $J$ = 8.7 Hz, 2H), 5.37 (s, 2H), 4.01 (s, 2H), 3.54 (s, 4H), 2.51 (s, 4H); HRMS(ESI)$(M+H)^+$, m/z: calcd for $C_{21}H_{20}FN_5O_2$ 393.1601, found 394.1670.

### 2.3.38. 4–(4-Fluorobenzyl)-1-(pyrrolidin-1-ylmethyl)-[1,2,4]triazolo [4,3-a]quinazolin-5(4H)-one (A13)

White solid; yield: 65.2%; *m.p.*: 1 4 5 ~ 147 °C; $^1$H NMR (400 MHz, DMSO-$d_6$) $\delta$ 8.31 – 8.22 (m, 2H), 7.97 (t, $J$ = 7.6 Hz, 1H), 7.61 (t, $J$ = 7.5 Hz, 1H), 7.54 – 7.47 (m, 2H), 7.14 (t, $J$ = 8.7 Hz, 2H), 5.37 (s,

2H), 4.10 (s, 2H), 2.59 (s, 4H), 1.71 (s, 4H); HRMS(ESI)(M + H)$^+$, m/z: calcd for $C_{21}H_{20}FN_5O$ 377.1652, found 378.1720.

### 2.3.39. 4–(4-Fluorobenzyl)-1-((4-methylpiperazin-1-yl)methyl)-[1,2,4] triazolo[4,3-a] quinazolin-5(4H)-one (A14)

White solid; yield: 70.8%; *m.p.*: 2 2 3 ~ 225 °C; $^1$H NMR (400 MHz, DMSO-$d_6$) $\delta$ 8.30 − 8.23 (m, 2H), 7.96 (t, $J$ = 7.7 Hz, 1H), 7.62 (t, $J$ = 7.6 Hz, 1H), 7.55 − 7.50 (m, 2H), 7.14 (t, $J$ = 8.7 Hz, 2H), 5.36 (s, 2H), 3.98 (s, 2H), 2.51 (s, 4H), 2.29 (s, 4H), 2.12 (s, 3H); HRMS(ESI)(M + H)$^+$, m/z: calcd for $C_{22}H_{23}FN_6O$ 406.1917, found 407.1987.

### 2.3.40. 4–(4-Fluorobenzyl)-1-(piperidin-1-ylmethyl)-[1,2,4]triazolo [4,3-a]quinazolin-5(4H)-one (A15)

White solid; yield: 72.0%; *m.p.*: 1 8 9 ~ 191 °C; $^1$H NMR (400 MHz, $CDCl_3$) $\delta$ (ppm) = 8.40 (dd, $J$ = 13.4, 8.3 Hz, 2H), 7.80 (t, $J$ = 7.7 Hz, 1H), 7.76 − 7.68 (m, 2H), 7.54 (t, $J$ = 7.5 Hz, 1H), 6.99 (t, $J$ = 8.6 Hz, 2H), 5.50 (s, 2H), 3.94 (s, 2H), 2.55 (s, 4H), 1.52 (d, $J$ = 32.3 Hz, 6H); HRMS(ESI)(M + H)$^+$, m/z: calcd for $C_{22}H_{22}FN_5O$ 391.1808, found 392.1882.

### 2.3.41. 4–(4-Fluorobenzyl)-1-(thiomorpholinomethyl)-[1,2,4]triazolo [4,3-a]quinazolin-5(4H)-one (A16)

White solid; yield: 73.1%; *m.p.*: 2 4 1 ~ 243 °C; $^1$H NMR (400 MHz, $CDCl_3$) $\delta$ (ppm) = 8.48 (d, $J$ = 7.8 Hz, 1H), 8.24 (d, $J$ = 8.3 Hz, 1H), 7.85 (t, $J$ = 7.7 Hz, 1H), 7.80 − 7.73 (m, 2H), 7.60 (t, $J$ = 7.6 Hz, 1H), 7.04 (t, $J$ = 8.6 Hz, 2H), 5.55 (s, 2H), 4.06 (s, 2H), 2.94 (s, 4H), 2.71 (s, 4H); HRMS(ESI)(M + H)$^+$, m/z: calcd for $C_{21}H_{20}FN_5OS$ 409.1373, found 410.1449.

### 2.3.42. 4–(4-Methoxybenzyl)-1-(morpholinomethyl)-[1,2,4]triazolo [4,3-a]quinazolin-5(4H)-one (A17)

White solid; yield: 66.7%; *m.p.*: 2 0 9 ~ 211 °C; $^1$H NMR (400 MHz, DMSO-$d_6$) $\delta$ 8.26 (t, $J$ = 8.4 Hz, 2H), 7.96 (t, $J$ = 7.8 Hz, 1H), 7.62 (t, $J$ = 7.5 Hz, 1H), 7.43 (d, $J$ = 8.2 Hz, 2H), 6.87 (d, $J$ = 8.4 Hz, 2H), 5.32 (s, 2H), 4.01 (s, 2H), 3.70 (s, 3H), 3.54 (s, 4H), 2.51 (s, 4H); HRMS(ESI)(M + H)$^+$, m/z: calcd for $C_{22}H_{23}N_5O_3$ 405.1801, found 406.1866.

### 2.3.43. 4–(4-Methoxybenzyl)-1-(pyrrolidin-1-ylmethyl)-[1,2,4]triazolo [4,3-a] quinazolin-5(4H)-one (A18)

White solid; yield: 65.2%; *m.p.*: 1 4 5 ~ 147 °C; $^1$H NMR (400 MHz, DMSO-$d_6$) $\delta$ 8.25 (t, $J$ = 8.4 Hz, 2H), 7.96 (t, 1 = 7.4 Hz, 1H), 7.61 (t, $J$ = 7.5 Hz, 1H), 7.42 (d, $J$ = 8.4 Hz, 2H), 6.87 (d, $J$ = 8.5 Hz, 2H), 5.32 (s, 2H), 4.09 (s, 2H), 3.70 (s, 3H), 2.59 (s, 4H), 1.71 (s, 4H); HRMS(ESI)(M + H)$^+$, m/z: calcd for $C_{22}H_{23}N_5O_2$ 389.1852, found 390.1918.

### 2.3.44. 4–(4-Methoxybenzyl)-1-((4-methylpiperazin-1-yl)methyl)-[1,2,4]triazolo[4,3-a] quinazolin-5(4H)-one (A19)

White solid; yield: 68.0%; *m.p.*: 2 4 5 ~ 247 °C; $^1$H NMR (400 MHz, DMSO-$d_6$) $\delta$ 8.26 (t, $J$ = 9.1 Hz, 2H), 7.95 (t, $J$ = 7.8 Hz, 1H), 7.61 (t, $J$ = 7.6 Hz, 1H), 7.43 (d, $J$ = 8.3 Hz, 2H), 6.87 (d, $J$ = 8.3 Hz, 2H), 5.32 (s, 2H), 3.98 (s, 2H), 3.71 (s, 3H), 2.52 (s, 4H), 2.29 (s, 4H), 2.12 (s, 3H); HRMS(ESI)(M + H)$^+$, m/z: calcd for $C_{23}H_{26}N_6O_2$ 418.2117, found 419.2184.

### 2.3.45. 4–(4-Methoxybenzyl)-1-(((4-methoxybenzyl)amino)methyl)-[1,2,4]triazolo[4,3-a]quinazolin-5(4H)-one (A20)

White solid; yield: 66.7%; *m.p.*: 1 6 0 ~ 162 °C; $^1$H NMR (400 MHz, DMSO-$d_6$) $\delta$ 8.26 (d, $J$ = 7.7 Hz, 2H), 7.88 (t, $J$ = 7.4 Hz, 1H), 7.62 (s, 1H), 7.41 (d, $J$ = 8.4 Hz, 2H), 7.22 (d, $J$ = 8.3 Hz, 2H), 6.90 − 6.80 (m, 4H), 5.31 (s, 2H), 4.15 (s, 2H), 3.71 (s, 2H), 3.70 (d, $J$ = 2.6 Hz, 6H), 2.92 (s, 1H); HRMS(ESI)(M + H)$^+$, m/z: calcd for $C_{26}H_{25}N_5O_3$ 455.1957, found 456.2023.

### 2.3.46. 4–(4-Methoxybenzyl)-1-(piperidin-1-ylmethyl)-[1,2,4]triazolo [4,3-a] quinazolin-5(4H)-one (A21)

White solid; yield: 69.2%; *m.p.*: 1 9 0 ~ 192 °C; $^1$H NMR (400 MHz, $CDCl_3$) $\delta$ (ppm) = 8.41 (d, $J$ = 7.8 Hz, 1H), 8.36 (d, $J$ = 8.4 Hz, 1H), 7.78 (t, $J$ = 7.9 Hz, 1H), 7.69 (d, $J$ = 8.4 Hz, 2H), 7.52 (t, $J$ = 7.6 Hz, 1H), 6.84 (d, $J$ = 8.5 Hz, 2H), 5.49 (s, 2H), 3.93 (s, 2H), 3.76 (s, 3H), 2.55 (s, 4H), 1.54 (s, 4H), 1.47 (s, 2H); HRMS(ESI)(M + H)$^+$, m/z: calcd for $C_{23}H_{25}N_5O_2$ 403.2008, found 404.2080.

### 2.3.47. 4–(4-Methoxybenzyl)-1-(thiomorpholinomethyl)-[1,2,4]triazolo [4,3-a] quinazolin-5(4H)-one (A22)

White solid; yield: 55.6%; *m.p.*: 2 3 7 ~ 239 °C; $^1$H NMR (400 MHz, $CDCl_3$) $\delta$ (ppm) = 8.43 (d, $J$ = 7.8 Hz, 1H), 8.18 (d, $J$ = 8.4 Hz, 1H), 7.78 (t, $J$ = 7.8 Hz, 1H), 7.69 (d, $J$ = 8.5 Hz, 2H), 7.54 (t, $J$ = 7.6 Hz, 1H), 6.84 (d, $J$ = 8.5 Hz, 2H), 5.48 (s, 2H), 4.01 (s, 2H), 3.76 (s, 3H), 2.90 (s, 4H), 2.67 (s, 4H); HRMS(ESI)(M + H)$^+$, m/z: calcd for $C_{22}H_{23}N_5O_2S$ 421.1572, found 422.1646.

### 2.3.48. 1-((4-Hydroxypiperidin-1-yl)methyl)-4–(4-methoxybenzyl)-[1,2,4]triazolo[4,3-a] quinazolin-5(4H)-one (A23)

White solid; yield: 59.3%; *m.p.*: 2 5 9 ~ 261 °C; $^1$H NMR (400 MHz, $CDCl_3$) $\delta$ (ppm) = 8.42 (d, $J$ = 7.9 Hz, 1H), 8.28 (d, $J$ = 8.4 Hz, 1H), 7.78 (t, $J$ = 7.8 Hz, 1H), 7.69 (d, $J$ = 8.4 Hz, 2H), 7.53 (t, $J$ = 7.6 Hz, 1H), 6.84 (d, $J$ = 8.4 Hz, 2H), 5.48 (s, 2H), 3.98 (s, 2H), 3.76 (s, 3H), 2.86 (s, 2H), 2.39 (s, 2H), 1.89 (s, 2H), 1.55 (d, $J$ = 9.8 Hz, 2H), 1.46 (s, 1H); HRMS(ESI)(M + H)$^+$, m/z: calcd for $C_{23}H_{25}N_5O_3$ 419.1957, found 420.1231.

### 2.3.49. 4–(4-Methoxybenzyl)-1-((4-methylpiperidin-1-yl)methyl)-[1,2,4]triazolo[4,3-a] quinazolin-5(4H)-one (A24)

White solid; yield: 59.3%; *m.p.*: 2 4 0 ~ 242 °C; $^1$H NMR (400 MHz, $CDCl_3$) $\delta$ (ppm) = 8.41 (d, $J$ = 7.9 Hz, 1H), 8.34 (d, $J$ = 8.4 Hz, 1H), 7.78 (t, $J$ = 7.8 Hz, 1H), 7.69 (d, $J$ = 8.4 Hz, 2H), 7.52 (t, $J$ = 7.6 Hz, 1H), 6.84 (d, $J$ = 8.4 Hz, 2H), 5.48 (s, 2H), 3.94 (s, 2H), 3.76 (s, 3H), 2.90 (d, $J$ = 11.2 Hz, 2H), 2.22 (t, $J$ = 11.4 Hz, 2H), 1.65 (s, 2H), 1.41 (s, 1H), 1.14 (d, $J$ = 10.3 Hz, 2H), 0.90 (d, $J$ = 6.4 Hz, 3H); HRMS(ESI)(M + H)$^+$, m/z: calcd for $C_{24}H_{27}N_5O_2$ 417.2165, found 418.2239.

### 2.3.50. 4–(4-Methoxybenzyl)-1-((3-methylpiperidin-1-yl)methyl)-[1,2,4] triazolo[4,3-a] quinazolin-5(4H)-one (A25)

White solid; yield: 63.0%; *m.p.*: 1 5 6 ~ 158 °C; $^1$H NMR (400 MHz, $CDCl_3$) $\delta$ (ppm) = 8.41 (d, $J$ = 7.9 Hz, 1H), 8.37 (d, $J$ = 8.4 Hz, 1H), 7.77 (t, $J$ = 7.8 Hz, 1H), 7.70 (d, $J$ = 8.5 Hz, 2H), 7.52 (t, $J$ = 7.6 Hz, 1H), 6.84 (d, $J$ = 8.5 Hz, 2H), 5.49 (s, 2H), 3.93 (s, 2H), 3.76 (s, 3H), 2.82 (s, 2H), 2.18 (t, $J$ = 10.7 Hz, 1H), 1.89 (t, $J$ = 10.4 Hz, 1H), 1.66 (s, 2H), 1.59 − 1.56 (m, 1H), 1.48 (d, $J$ = 11.8 Hz, 1H), 0.96 (d, $J$ = 10.4 Hz, 1H), 0.87 (d, $J$ = 6.5 Hz, 3H); HRMS(ESI)(M + H)$^+$, m/z: calcd for $C_{24}H_{27}N_5O_2$ 417.2165, found 418.2237.

### 2.3.51. 4–(4-Methoxybenzyl)-1-((2-methylpiperidin-1-yl)methyl)-[1,2,4]triazolo[4,3-a] quinazolin-5(4H)-one (A26)

White solid; yield: 63.0%; *m.p.*: 1 8 4 ∼ 186 °C; $^1$H NMR (400 MHz, $CDCl_3$) $\delta$ (ppm) = 8.70 (d, $J$ = 8.4 Hz, 1H), 8.40 (d, $J$ = 7.8 Hz, 1H), 7.78 (t, $J$ = 7.6 Hz, 1H), 7.69 (d, $J$ = 8.5 Hz, 2H), 7.52 (t, $J$ = 7.5 Hz, 1H), 6.84 (d, $J$ = 8.4 Hz, 2H), 5.48 (s, 2H), 4.32 (d, $J$ = 13.7 Hz, 1H), 3.88 (d, $J$ = 13.8 Hz, 1H), 3.76 (s, 3H), 2.64 (s, 2H), 2.31 (s, 1H), 1.70 (s, 1H), 1.65 − 1.60 (m, 1H), 1.40 (s, 4H), 1.26 (d, $J$ = 6.3 Hz, 3H); HRMS(ESI)(M + H)+, m/z: calcd for $C_{24}H_{27}N_5O_2$ 417.2165, found 418.2237.

### 2.3.52. Tert-butyl 4-((4–(4-fluorobenzyl)-5-oxo-4,5-dihydro-[1,2,4] triazolo[4,3-a] quinazolin-1-yl)methyl)piperazine-1-carboxylate (A27)

White solid; yield: 66.7%; *m.p.*: 2 0 2 ∼ 204 °C; $^1$H NMR (400 MHz, DMSO-$d_6$) $\delta$ 8.26 (t, $J$ = 8.4 Hz, 2H), 7.97 (t, $J$ = 7.5 Hz, 1H), 7.62 (t, $J$ = 7.5 Hz, 1H), 7.56 − 7.49 (m, 2H), 7.15 (t, $J$ = 8.8 Hz, 2H), 5.37 (s, 2H), 4.02 (s, 2H), 3.33 (s, 4H), 2.51 (s, 4H), 1.39 (s, 9H); HRMS(ESI)(M + H)$^+$, m/z: calcd for $C_{26}H_{29}FN_6O_3$ 492.2285, found 493.2350.

### 2.3.53. Tert-butyl 4-((4–(4-methoxybenzyl)-5-oxo-4,5-dihydro-[1,2,4] triazolo[4,3-a] quinazolin-1-yl)methyl)piperazine-1-carboxylate (A28)

White solid; yield: 73.3%; *m.p.*: 2 4 5 ∼ 247 °C; $^1$H NMR (400 MHz, DMSO-$d_6$) $\delta$ 8.26 (dd, $J$ = 13.4, 8.3 Hz, 2H), 7.96 (t, $J$ = 7.7 Hz, 1H), 7.62 (t, $J$ = 7.5 Hz, 1H), 7.43 (d, $J$ = 8.4 Hz, 2H), 6.87 (d, $J$ = 8.4 Hz, 2H), 5.32 (s, 2H), 4.02 (s, 2H), 3.71 (s, 3H), 3.28 (s, 8H), 1.39 (s, 10H); HRMS(ESI)(M + H)$^+$, m/z: calcd for $C_{27}H_{32}N_6O_4$ 504.2485, found 505.2550.

### 2.3.54. General procedure for the preparation of triazoloquinazolinone derivatives (A29–A32)

In a 250 mL single-neck flask, dissolve compounds ***A27*** or ***A28*** (20.32 mmol) in 50 mL methanol solution, add 15 mL concentrated hydrochloric acid solution, after the addition, react at room temperature for 4 h, and TLC monitors the completion of the reaction. The reaction solution was concentrated under reduced pressure to remove the solvent methanol to obtain the crude hydrochloride for use.

In a 50 mL single-neck flask, dissolve the above crude hydrochloride (0.47 mmol) in 15 mL of dichloromethane under ice bath conditions, and add triethylamine (0.1 mL, 0.72 mmol) and propyl sulphonyl chloride (0.15 mL, 1.50 mmol) or chloroacetyl chloride (0.12 mL, 1.50 mmol). After the addition, the reaction was carried out for 30 min under ice bath conditions, and the completion of the reaction was monitored by TLC. The reaction solution was concentrated under reduced pressure to remove the solvent dichloromethane to obtain a crude product. The crude product was stirred with water, filtered with suction, and the filter cake was washed twice with acetone to obtain compounds ***A29–A32***.

### 2.3.55. 1-((4-(Cyclopropylsulfonyl)piperazin-1-yl)methyl)-4–(4-fluorobenzyl)-[1,2,4] triazolo[4,3-a]quinazolin-5(4H)-one (A29)

White solid; yield: 70.0%; m.p.: 2 4 0 ∼ 242 °C; 1H NMR (400 MHz, CDCl3) $\delta$ 8.45 (d, $J$ = 7.5 Hz, 1H), 8.11 (d, $J$ = 8.2 Hz, 1H), 7.81 (t, $J$ = 7.4 Hz, 1H), 7.72 (s, 2H), 7.57 (t, $J$ = 7.3 Hz, 1H), 6.99 (t, $J$ = 8.2 Hz, 2H), 5.50 (s, 2H), 4.08 (s, 2H), 3.32 (s, 4H), 2.75 (s, 4H), 2.23 (s, 1H), 1.15 (s, 2H), 0.96 (d, $J$ = 6.3 Hz, 2H); HRMS(ESI)(M + H)$^+$, m/z: calcd for $C_{24}H_{25}N_6O_3S$ 496.1693, found 497.1760.

### 2.3.56. 1-((4-(Cyclopropylsulfonyl)piperazin-1-yl)methyl)-4–(4-methoxybenzyl)-[1,2,4] triazolo[4,3-a]quinazolin-5(4H)-one (A30)

White solid; yield: 68.8%; *m.p.*: 2 2 5 ∼ 227 °C; $^1$H NMR (400 MHz, $CDCl_3$) $\delta$ 8.46 (d, $J$ = 8.2 Hz, 1H), 8.05 (d, $J$ = 8.7 Hz, 1H), 7.81 (t, $J$ = 7.7 Hz, 1H), 7.68 (d, $J$ = 8.4 Hz, 2H), 7.57 (t, $J$ = 7.3 Hz, 1H), 6.84 (d, $J$ = 8.2 Hz, 2H), 5.49 (s, 2H), 4.19 (s, 2H), 3.76 (s, 3H), 3.40 (s, 4H), 2.90 (s, 4H), 2.25 (s, 1H), 1.15 (s, 2H), 0.98 (d, $J$ = 6.9 Hz, 2H); HRMS(ESI)(M + H)$^+$, m/z: calcd for $C_{25}H_{28}N_6O_4S$ 508.1893, found 509.1961.

### 2.3.57. 1-((4–(2-Chloroacetyl)piperazin-1-yl)methyl)-4–(4-fluorobenzyl)-[1,2,4]triazolo [4,3-a]quinazolin-5(4H)-one (A31)

White solid; yield: 71.4%; *m.p.*: 2 1 7 ∼ 219 °C; $^1$H NMR (400 MHz, DMSO-$d_6$) $\delta$ 8.31 − 8.23 (m, 2H), 7.98 (t, $J$ = 6.9 Hz, 1H), 7.63 (t, $J$ = 7.4 Hz, 1H), 7.55 − 7.51 (m, 2H), 7.15 (t, $J$ = 8.9 Hz, 2H), 5.37 (s, 2H), 4.37 (s, 2H), 4.05 (s, 2H), 3.42 (s, 4H), 2.66 (d, $J$ = 67.3 Hz, 4H); HRMS(ESI)(M + H)$^+$, m/z: calcd for $C_{23}H_{22}ClFN_6O_2$ 468.9174, found 469.9137.

### 2.3.58. 1-((4–(2-Chloroacetyl)piperazin-1-yl)methyl)-4–(4-methoxybenzyl)-[1,2,4] triazolo[4,3-a]quinazolin-5(4H)-one (A32)

White solid; yield: 74.2%; *m.p.*: 1 9 3 ∼ 195 °C; $^1$H NMR (400 MHz, DMSO-$d_6$) $\delta$ 8.26 (dd, $J$ = 14.6, 8.1 Hz, 2H), 7.98 (t, $J$ = 7.5 Hz, 1H), 7.62 (t, $J$ = 7.5 Hz, 1H), 7.43 (d, $J$ = 8.4 Hz, 2H), 6.88 (d, $J$ = 8.4 Hz, 2H), 5.32 (s, 2H), 4.38 (s, 2H), 4.05 (s, 2H), 3.71 (s, 3H), 3.42 (s, 4H), 2.58 (s, 4H); HRMS(ESI)(M + H)$^+$, m/z: calcd for $C_{24}H_{25}ClN_6O_3$ 480.1677, found 481.1741

### 2.3.59. General procedure for the preparation of triazoloquinazolinone derivatives (A33–A35)

In a 50 mL single-neck flask, compounds **A27** or **A28** (0.43 mmol) was dissolved in 15 mL of acetonitrile, and potassium carbonate (0.18 g, 1.30 mmol) and the corresponding nitrogen-containing reagent (0.46 mmol) were added under stirring. After the addition, the reaction system was heated to 50 °C for 4 h, and TLC monitored the completion of the reaction. The reaction was quenched by adding water, the solid was stirred out, filtered with suction, and the filter cake was slurried twice with acetone to obtain compounds **A33-A35**.

### 2.3.60. 4–(4-Fluorobenzyl)-1-((4–(2-morpholinoacetyl)piperazin-1-yl) methyl)-[1,2,4] triazolo[4,3-a]quinazolin-5(4H)-one (A33)

White solid; yield: 64.5%; *m.p.*: 1 9 7 ∼ 199 °C; $^1$H NMR (400 MHz, DMSO-$d_6$) $\delta$ 8.31 − 8.24 (m, 2H), 7.98 (t, $J$ = 7.6 Hz, 1H), 7.63 (t, $J$ = 7.5 Hz, 1H), 7.56 − 7.50 (m, 2H), 7.15 (t, $J$ = 8.7 Hz, 2H), 5.37 (s, 2H), 4.03 (s, 2H), 3.56 (s, 4H), 3.51 (s, 2H), 3.41 (s, 2H), 3.13 (s, 2H), 2.55 (s, 4H), 2.38 (s, 4H); HRMS(ESI)(M + H)$^+$, m/z: calcd for $C_{27}H_{30}FN_7O_3$ 519.6394, found 520.2462.

### 2.3.61. 4–(4-Methoxybenzyl)-1-((4–(2-morpholinoacetyl)piperazin-1-yl)methyl)-[1,2,4] triazolo[4,3-a]quinazolin-5(4H)-one (A34)

White solid; yield: 71.9%; *m.p.*: 2 3 5 ∼ 237 °C; $^1$H NMR (400 MHz, DMSO-$d_6$) $\delta$ 8.31 − 8.23 (m, 2H), 7.98 (t, $J$ = 7.5 Hz, 1H), 7.63 (t, $J$ = 7.6 Hz, 1H), 7.43 (d, $J$ = 8.3 Hz, 2H), 6.88 (d, $J$ = 8.5 Hz, 2H), 5.32 (s, 2H), 4.03 (s, 2H), 3.71 (s, 3H), 3.56 (s, 4H), 3.50 (s, 2H), 3.41 (s,

2H), 3.13 (s, 2H), 2.55 (s, 4H), 2.38 (s, 4H); HRMS(ESI)(M + H)$^+$, m/z: calcd for $C_{28}H_{33}N_7O_4$ 531.2594, found 532.2664.

### 2.3.62. 4–(4-Methoxybenzyl)-1-((4–(2-(pyrrolidin-1-yl)acetyl)piperazin-1-yl)methyl)-[1,2,4]triazolo[4,3-a]quinazolin-5(4H)-one (A35)

2.3.64. Yellow solid; yield: 67.7%; *m.p.*: 2 3 5 ∼ 237 °C; $^1$H NMR (400 MHz, DMSO-$d_6$) $\delta$ 8.26 (t, $J$ = 9.0 Hz, 2H), 7.97 (s, 1H), 7.62 (s, 1H), 7.43 (d, $J$ = 7.5 Hz, 2H), 6.88 (d, $J$ = 8.1 Hz, 2H), 5.32 (s, 2H), 4.02 (s, 2H), 3.71 (s, 4H), 3.48 (s, 4H), 3.23 (s, 2H), 2.45 (s, 4H), 1.67 (s, 4H); HRMS(ESI)(M + H)$^+$, m/z: calcd for $C_{28}H_{33}N_7O_3$ 515.2645, found 516.2710

### 2.3.63. 2-Chloro-3–(2-chlorobenzyl)quinazolin-4(3H)-one (10)

At room temperature, take the intermediate compound ***7c*** (30 g, 99.08 mmol) and place it in a 500 mL single-necked flask, then add phosphorus oxychloride (364.6 g, 2377.88 mmol), and add phosphorus pentachloride under stirring. (41.27 g, 198.18 mmol), the temperature was raised to 80 °C, and the reaction was carried out for 4 h. After the completion of the reaction was detected by TLC, the reaction solution was transferred to a rotary evaporator, and the reaction solvent was removed by distillation under reduced pressure. The crude product was obtained as a yellow solid. The crude product was pulverised and poured into ice water (300 mL) and stirred for 1 h. The filter cake was collected by suction filtration and dried to obtain a shallow compound ***10***. Light yellow solid; yield: 83.0%; proceed to the next step without characterisation.

### 2.3.64. 4–(2-Chlorobenzyl)imidazo[1,2-a]quinazolin-5(4H)-one (11)

At room temperature, take intermediate compound ***10*** (10 g, 32.77 mmol) into a 250 mL single-necked flask, add 100 mL of absolute ethanol, and add triethylamine (19.90 g, 196.6 mmol), aminoacetaldehyde dimethyl acetal (20.67 g, 196.60 mmol), heat up to 80 °C, and react for 1 h. After the completion of the reaction was detected by TLC, the reaction solution was transferred to a rotary evaporator, and the reaction solvent was removed by distillation under reduced pressure to obtain the residue as a white solid. Dissolve the white solid with ethyl acetate (200 mL), then wash with citric acid aqueous solution (150 mL × 2), collect the organic layer, then backwash the organic layer (100 mL) with water, collect the organic layer. The organic layer was dried over sodium sulphate, and the organic solvent was removed by distillation under reduced pressure to obtain the crude product as a white solid. The white solid was slurried with acetone (30 mL × 2) to obtain 10.55 g of a white solid. Put the white solid (10.55 g, 28.22 mmol) in a 250 mL single-necked flask, add 48% HBr (47.57 g, 282.22 mmol), raise the temperature to 100 °C, reflux for 5 h, and detect by TLC after the reaction is complete. The reaction solution was moved to room temperature. After the reaction solution was cooled, the pH of the reaction solution was adjusted to 7–8 with ammonia water, and a white solid was precipitated. Suction filtration, collecting the filter cake, and obtaining the crude product as a white solid. The white solid was slurried with acetone (20 mL × 3) to obtain compound **11**. White solid; yield: 76.2%; $^1$H NMR (400 MHz, DMSO-d$_6$) $\delta$ 8.21 (d, $J$ = 7.6 Hz, 1H), 8.15 – 8.10 (m, 2H), 7.92 (t, $J$ = 7.4 Hz, 1H), 7.54 – 7.48 (m, 2H), 7.27 (t, $J$ = 7.3 Hz, 1H), 7.16 (t, $J$ = 7.3 Hz, 1H), 7.07 (s, 1H), 6.98 (d, $J$ = 7.5 Hz, 1H), 5.42 (s, 2H).

### 2.3.65. 4–(2-Chlorobenzyl)-5-oxo-4,5-dihydroimidazo[1,2-a]quinazoline-1-carbaldehyde (12)

At room temperature, take the intermediate compound **11** (5.0 g, 16.14 mmol) and place it in a 100 mL single-necked flask, then add phosphorus oxychloride (9.9 g, 64.57 mmol), and then slowly add *N, N*-dimethylformamide (2 mL) was heated to 90 °C and reacted for 5 h. After the reaction was detected by TLC, the reaction solution was moved to room temperature. After the reaction solution was cooled, the reaction solution was slowly poured into ice water (150 mL). An off-white solid precipitated out. The filter cake was collected by suction to obtain the crude product as off-white. The solid was purified by beating the filter cake with acetone (20 mL) to obtain compound **12**. White solid; yield: 81.0%; $^1$H NMR (400 MHz, DMSO-$d_6$) $\delta$ 9.66 (s, 1H), 9.26 (d, $J$ = 8.4 Hz, 1H), 8.30 (d, $J$ = 6.3 Hz, 2H), 8.02 (t, $J$ = 7.8 Hz, 1H), 7.66 (t, $J$ = 7.5 Hz, 1H), 7.52 (d, $J$ = 7.9 Hz, 1H), 7.31 (t, $J$ = 7.1 Hz, 1H), 7.17 (q, $J$ = 7.5 Hz, 2H), 5.51 (s, 2H).

### 2.3.66. General procedure for the preparation of imidazoquinazolinone derivatives (B1–B5)

At room temperature, take the intermediate compound ***12*** (0.59 mmol) and place it in a 50 mL single-necked flask. In an ice bath, add glacial acetic acid (0.07 g, 1.18 mmol) and the corresponding nitrogen-containing reagent (1.30 mmol). After stirring for 15 min in an ice bath, add sodium triacetoxyborohydride (0.502 g, 0.98 mmol), remove the ice bath, and react at room temperature for 18 h. After the completion of the reaction was detected by TLC, the reaction solution was transferred to a rotary evaporator, and the reaction solvent was removed by distillation under reduced pressure to obtain a yellow solid. Then add water (10 mL) to the bottle, adjust the pH to 1 with concentrated hydrochloric acid, extract with dichloromethane (20 mL × 2), collect the water layer, and then adjust the pH of the water layer to alkaline with ammonia water, white The solid precipitated, filtered with suction, and the filter cake was collected as a crude product. Then it was separated and purified by column chromatography (mobile phase: petroleum ether: ethyl acetate = 1: 1) to obtain compounds **B1–B5**.

### 2.3.67. 4–(4-Fluorobenzyl)-1-((4–(2-morpholinoacetyl)piperazin-1-yl)methyl)-[1,2,4] triazolo[4,3-a]quinazolin-5(4H)-one (B1)

White solid; yield: 20.0%; *m.p.*: 174 °C; $^1$H NMR (400 MHz, $CDCl_3$) $\delta$ 8.51 (t, $J$ = 9.5 Hz, 2H), 7.84 (t, $J$ = 7.8 Hz, 1H), 7.53 (t, $J$ = 7.5 Hz, 1H), 7.45 (d, $J$ = 7.7 Hz, 1H), 7.22 (t, $J$ = 7.3 Hz, 1H), 7.15 (t, $J$ = 7.4 Hz, 1H), 7.05–6.99 (m, 2H), 5.72 (s, 2H), 3.81 (s, 2H), 2.66 (s, 4H), 2.52 (s, 4H), 2.35 (s, 3H); HRMS(ESI)(M + H)$^+$, m/z: calcd for $C_{23}H_{24}ClN_5O$ 421.1669, found 422.2328.

### 2.3.68. 4–(2-Chlorobenzyl)-1-(pyrrolidin-1-ylmethyl)imidazo[1,2-a] quinazolin-5(4H)-one (B2)

White solid; yield: 47.0%; *m.p.*: 171 °C; $^1$H NMR (400 MHz, $CDCl_3$) $\delta$ 8.54 (d, $J$ = 8.7 Hz, 1H), 8.49 (d, $J$ = 7.6 Hz, 1H), 7.84 (t, $J$ = 7.5 Hz, 1H), 7.52 (t, $J$ = 7.4 Hz, 1H), 7.45 (d, $J$ = 7.8 Hz, 1H), 7.22 (t, $J$ = 7.2 Hz, 1H), 7.15 (t, $J$ = 7.0 Hz, 1H), 7.08 – 6.94 (m, 2H), 5.72 (s, 2H), 3.92 (s, 2H), 2.67 (s, 4H), 1.87 (s, 4H); HRMS(ESI)(M-H)$^-$, m/z: calcd for $C_{22}H_{21}ClN_4O$ 392.1404, found 391.2836.

### 2.3.69. 4–(2-Chlorobenzyl)-1-(piperidin-1-ylmethyl)imidazo[1,2-a] quinazolin-5(4H)-one (B3)

White solid; yield: 29.0%; *m.p.*: 185 °C; $^1$H NMR (400 MHz, $CDCl_3$) $\delta$ 8.62 (d, $J = 8.4$ Hz, 1H), 8.48 (d, $J = 7.8$ Hz, 1H), 7.83 (t, $J = 7.7$ Hz, 1H), 7.52 (t, $J = 7.5$ Hz, 1H), 7.44 (d, $J = 7.7$ Hz, 1H), 7.21 (t, $J = 7.4$ Hz, 1H), 7.15 (t, $J = 7.2$ Hz, 1H), 7.01 (d, $J = 6.8$ Hz, 2H), 5.72 (s, 2H), 3.72 (s, 2H), 2.55 (s, 4H), 1.63 (s, 4H), 1.53 (s, 2H); HRMS(ESI)(M-H)$^-$, m/z: calcd for $C_{23}H_{23}ClN_4O$ 406.1506, found 405.1456.

### 2.3.70. 4–(2-Chlorobenzyl)-1-(morpholinomethyl)imidazo[1,2-a]quinazolin-5(4H)-one (B4)

White solid; yield: 25.0%; *m.p.*: 192 °C; $^1$H NMR (400 MHz, $CDCl_3$) $\delta$ 8.50 (d, $J = 7.1$ Hz, 2H), 7.84 (t, $J = 8.2$ Hz, 1H), 7.54 (t, $J = 7.8$ Hz, 1H), 7.45 (d, $J = 7.1$ Hz, 1H), 7.22 (t, $J = 6.9$ Hz, 1H), 7.15 (t, $J = 7.0$ Hz, 1H), 7.05 – 6.98 (m, 2H), 5.71 (s, 2H), 3.81 (s, 2H), 3.78 (s, 4H), 2.64 (s, 4H); HRMS(ESI)(M + H)$^+$, m/z: calcd for $C_{22}H_{21}ClN_4O_2$ 408.1353, found 409.1141.

### 2.3.71. 4–(2-Chlorobenzyl)-1-(thiomorpholinomethyl)imidazo[1,2-a] quinazolin-5(4H)-one (B5)

White solid; yield: 36.0%; *m.p.*: 191 °C; $^1$H NMR (400 MHz, $CDCl_3$) $\delta$ 8.50 (d, $J = 7.8$ Hz, 1H), 8.45 (d, $J = 8.4$ Hz, 1H), 7.83 (t, $J = 7.6$ Hz, 1H), 7.54 (t, $J = 7.4$ Hz, 1H), 7.45 (d, $J = 7.8$ Hz, 1H), 7.22 (t, $J = 7.4$ Hz, 1H), 7.15 (t, $J = 7.5$ Hz, 1H), 7.01 (d, $J = 8.6$ Hz, 2H), 5.72 (s, 2H), 3.82 (s, 2H), 2.91 (s, 4H), 2.74 (s, 4H); HRMS(ESI)(M + H)$^+$, m/z: calcd for $C_{22}H_{21}ClN_4OS$ 424.1125, found 425.1089.

### 2.3.72. 2-Amino-3–(2-chlorobenzyl)quinazolin-4(3H)-one (13)

At room temperature, place Intermediate compound **10** (15 g, 49.15 mmol) in a 250 mL single-necked flask, add acetonitrile (150 mL), and add *p*-methoxybenzylamine (8.09 g, 58.99 mmol), potassium carbonate (20.35 g, 147.46 mmol), heated to 80 °C, and reacted for 8 h. After the reaction is detected by TLC, the reaction solution is moved to room temperature. After the reaction solution is cooled, water is added to the reaction solution for extraction (50 mL × 2), the filtrate is collected, dried with anhydrous sodium sulphate, and then distilled under reduced pressure. The organic solvent was removed, and 17.2 g of the crude product was obtained as a yellow oil. The yellow oil was not purified, and trifluoroacetic acid (96.64 g, 847.57 mmol) was directly added to the yellow oil, the temperature was raised to 80 °C, and the reaction was carried out for 24 h. After the completion of the reaction was detected by TLC, the reaction solution was distilled under reduced pressure to remove trifluoroacetic acid. The residue was a dark green oily substance. Add water (50 mL) to the bottle. After stirring, the oily substance became a solid. The filter cake was collected, and the crude product was obtained as a green solid. The crude product was purified by beating with methyl *tert*-butyl ether (20 mL × 3), and dried to obtain compound **13**. Light green solid; yield: 80.0%; $^1$H NMR (400 MHz, $CDCl_3$) $\delta$ 8.25 (d, $J = 7.9$ Hz, 1H), 7.79 (t, $J = 7.7$ Hz, 1H), 7.61 (d, $J = 8.2$ Hz, 1H), 7.53 – 7.42 (m, 2H), 7.33 (dt, $J = 20.8$, 7.4 Hz, 2H), 7.23 (s, 1H), 5.49 (s, 2H), 3.59 (s, 2H).

### 2.3.73. 4–(2-Chlorobenzyl)-5-oxo-4,5-dihydroimidazo[1,2-a]quinazoline-2-carboxylic acid (14)

At room temperature, place the intermediate compound **13** (9.5 g, 33.25 mmol) in a 250 mL single-necked flask, add absolute ethanol (150 mL) to the flask, and then add ethyl bromopyruvate one by one while stirring Ester (12.97 g, 66.51 mmol) and sodium bicarbonate (5.59 g, 66.54 mmol) were heated to 100 °C and reacted for 7 h. After the reaction was detected by TLC, the reaction solution was moved to room temperature. After the reaction solution was cooled to room temperature, the reaction solution was suction filtered and the filter cake was collected. The crude product was obtained as an off-white solid. The solid was slurried with acetone (20 mL × 2), Purified and dried to obtain 10.0 g of white solid. Take the white solid (10.0 g, 26.19 mmol) and place it in a 100 mL single-necked flask, then add 1 mol/l sodium hydroxide aqueous solution (50 mL) to the flask, and raise the temperature to 100 °C. After the reaction was detected by TLC, the reaction solution was moved to room temperature. After the reaction solution was cooled to room temperature, the insoluble matter in the reaction solution was filtered out and discarded, and the aqueous layer was treated with 1 mol/l HCl solution (10 mL) to the pH is 1 ∼ 2, the filter cake is washed with water (50 mL), the filter cake is collected, and the filter cake is purified by beating the filter cake with acetone (20 mL × 2). After drying, compound **14**. White solid; yield: 70.0%; proceed to the next step without characterisation.

### 2.3.74. General procedure for the preparation of imidazoquinazolinone derivatives (B6–B32)

At room temperature, place the intermediate compound **14** (0.57 mmol) in a 50 mL single-necked flask, add *N,N*-dimethylformamide (15 mL) to the flask, and then continue stirring. Add the corresponding nitrogen-containing reagent (1.20 mmol), PyBOP (0.35 g, 0.67 mmol), and triethylamine (0.11 g, 1.09 mmol), and then react at room temperature for 2 h. After the completion of the reaction was detected by TLC, water (30 mL) was added to the reaction solution, stirred for 10 min, filtered with suction, and the filter cake was collected. The filter cake was slurried with acetone (10 mL × 1) for purification, and dried to obtain the compounds ***B6–B32***.

### 2.3.75. 4–(2-Chlorobenzyl)-2–(4-methylpiperazine-1-carbonyl)imidazo[1,2-a] quinazolin-5(4H)-one (B6)

White solid; yield: 61.0%; *m.p.*: 218 °C; $^1$H NMR (400 MHz, $CDCl_3$) $\delta$ 8.50 (d, $J = 7.9$ Hz, 1H), 8.18 (s, 1H), 7.88 (t, $J = 7.7$ Hz, 1H), 7.69 (d, $J = 8.2$ Hz, 1H), 7.58 (t, $J = 7.6$ Hz, 1H), 7.44 (d, $J = 7.8$ Hz, 1H), 7.24 (t, $J = 7.0$ Hz, 1H), 7.20 – 7.15 (m, 2H), 5.70 (s, 2H), 4.18 (s, 2H), 3.80 (s, 2H), 2.50 (s, 2H), 2.40 (s, 2H), 2.36 (s, 3H); HRMS(ESI)(M + H)$^+$, m/z: calcd for $C_{22}H_{23}ClN_5O_2$ 435.1462, found 436.6664.

### 2.3.76. 4–(2-Chlorobenzyl)-2-(morpholine-4-carbonyl)imidazo[1,2-a] quinazolin-5(4H) -one (B7)

White solid; yield: 59.0%; *m.p.*: 241 °C; $^1$H NMR (400 MHz, DMSO-$d_6$) $\delta$ 8.70 (s, 1H), 8.30 (d, $J = 8.1$ Hz, 2H), 7.97 (t, $J = 7.8$ Hz, 1H), 7.62 (t, $J = 7.6$ Hz, 1H), 7.54 (d, $J = 7.9$ Hz, 1H), 7.32 (t, $J = 7.3$ Hz, 1H), 7.21 (dd, $J = 12.2$, 7.2 Hz, 2H), 5.48 (s, 2H), 3.91 (s, 4H), 3.57 (s, 4H); HRMS(ESI)(M + Na)$^+$, m/z: calcd for $C_{22}H_{19}ClN_4O_3$ 422.1146, found 445.1196.

### 2.3.77. 4–(2-Chlorobenzyl)-2-(pyrrolidine-1-carbonyl)imidazo[1,2-a] quinazolin-5(4H)-one (B8)

White solid; yield: 61.0%; *m.p.*: 265 °C; $^1$H NMR (400 MHz, DMSO-$d_6$) $\delta$ 8.72 (s, 1H), 8.33 (d, $J = 8.1$ Hz, 2H), 8.01 (t, $J = 7.8$ Hz, 1H), 7.66 (t, $J = 7.7$ Hz, 1H), 7.57 (s, 1H), 7.36 (t, $J = 7.3$ Hz, 1H), 7.28 – 7.18 (m, 2H), 5.51 (s, 2H), 4.00 (s, 4H), 2.66 (d, $J = 59.5$ Hz,

4H); HRMS(ESI)(M + Na)$^+$, m/z: calcd for $C_{22}H_{19}ClN_4O_2$ 406.1197, found 429.0882.

### 2.3.78. 4–(2-Chlorobenzyl)-2-(thiomorpholine-4-carbonyl)imidazo [1,2-a]quinazolin-5(4H)-one (B9)

White solid; yield: 65.0%; *m.p.*: 227 °C; $^1$H NMR (400 MHz, DMSO-$d_6$) $\delta$ 8.72 (s, 1H), 8.33 (d, $J$ = 8.1 Hz, 2H), 8.01 (t, $J$ = 7.8 Hz, 1H), 7.69 – 7.63 (m, 1H), 7.58 (d, $J$ = 7.7 Hz, 1H), 7.36 (t, $J$ = 7.3 Hz, 1H), 7.26 (t, $J$ = 7.4 Hz, 2H), 5.51 (s, 2H), 4.01 (s, 4H), 2.57 (s, 4H); HRMS(ESI)(M-H)$^-$, m/z: calcd for $C_{23}H_{19}ClN_4O_2S$ 438.0917, found 437.9257.

### 2.3.79. 4–(2-Chlorobenzyl)-2–(4-methylpiperidine-1-carbonyl)imidazo [1,2-a] quinazolin-5(4H)-one (B10)

White solid; yield: 61.0%; *m.p.*: 208 °C; $^1$H NMR (400 MHz, $CDCl_3$) $\delta$ 8.48 (d, $J$ = 7.8 Hz, 1H), 8.14 (s, 1H), 7.87 (t, $J$ = 7.3 Hz, 1H), 7.68 (d, $J$ = 8.2 Hz, 1H), 7.57 (t, $J$ = 7.5 Hz, 1H), 7.44 (d, $J$ = 7.6 Hz, 1H), 7.23 (t, $J$ = 7.4 Hz, 1H), 7.15 (q, $J$ = 7.9 Hz, 2H), 5.70 (s, 2H), 4.81 (d, $J$ = 132.2 Hz, 2H), 2.88 (d, $J$ = 99.1 Hz, 2H), 1.69 (d, $J$ = 37.4 Hz, 3H), 1.31 – 1.15 (m, 2H), 0.99 (d, $J$ = 6.3 Hz, 3H). HRMS(ESI)(M-H)$^-$, m/z: calcd for $C_{24}H_{23}ClN_4O_2$ 434.1510, found 433.6832.

### 2.3.80. 4–(2-Chlorobenzyl)-2–(4-hydroxypiperidine-1-carbonyl)imidazo[1,2-a] quinazolin-5(4H)-one (B11)

White solid; yiled: 57.0%; *m.p.*: 249 °C; $^1$H NMR (400 MHz, $CDCl_3$) $\delta$ 8.50 (d, $J$ = 8.0 Hz, 1H), 8.18 (s, 1H), 7.89 (t, $J$ = 7.7 Hz, 1H), 7.70 (d, $J$ = 8.2 Hz, 1H), 7.59 (t, $J$ = 7.4 Hz, 1H), 7.45 (d, $J$ = 8.0 Hz, 1H), 7.24 (t, $J$ = 7.3 Hz, 1H), 7.19 – 7.11 (m, 2H), 5.70 (s, 2H), 4.43 (d, $J$ = 143.5 Hz, 2H), 3.98 (s, 1H), 3.48 (d, $J$ = 108.3 Hz, 2H), 1.95 (d, $J$ = 36.4 Hz, 2H), 1.57 – 1.28 (m, 3H); HRMS(ESI)(M-H)$^-$, m/z: calcd for $C_{23}H_{21}ClN_4O_3$ 436.1302, found 435.2382.

### 2.3.81. 4–(2-Chlorobenzyl)-2-(piperidine-1-carbonyl)imidazo[1,2-a] quinazolin-5(4H)-one (B12)

White solid; yield: 67.0%; *m.p.*: 216 °C; $^1$H NMR (400 MHz, $CDCl_3$) $\delta$ 8.49 (d, $J$ = 7.8 Hz, 1H), 8.15 (s, 1H), 7.88 (t, $J$ = 7.6 Hz, 1H), 7.69 (d, $J$ = 8.1 Hz, 1H), 7.57 (t, $J$ = 7.6 Hz, 1H), 7.44 (d, $J$ = 7.8 Hz, 1H), 7.23 (t, $J$ = 7.3 Hz, 1H), 7.15 (q, $J$ = 7.7 Hz, 2H), 5.70 (s, 2H), 4.02 (s, 2H), 3.73 (s, 2H), 1.70 (s, 4H), 1.57 (s, 2H); HRMS(ESI)(M-H)$^-$, m/z: calcd for $C_{23}H_{21}ClN_4O_2$ 420.1353, found 419.7771.

### 2.3.82. 4–(2-Chlorobenzyl)-2–(3-methylpiperidine-1-carbonyl)imidazo [1,2-a] quinazolin-5(4H)-one (B13)

White solid; yield: 65.0%; *m.p.*: 194 °C; $^1$H NMR (400 MHz, $CDCl_3$) $\delta$ 8.49 (d, $J$ = 7.9 Hz, 1H), 8.15 (s, 1H), 7.87 (t, $J$ = 7.6 Hz, 1H), 7.69 (d, $J$ = 8.2 Hz, 1H), 7.57 (t, $J$ = 7.6 Hz, 1H), 7.44 (d, $J$ = 7.8 Hz, 1H), 7.22 (t, $J$ = 7.4 Hz, 1H), 7.14 (dd, $J$ = 18.2, 11.0 Hz, 2H), 5.70 (s, 2H), 4.96 – 4.44 (m, 2H), 2.77 (dd, $J$ = 119.1, 111.7 Hz, 2H), 1.91 – 1.71 (m, 2H), 1.53 (s, 2H), 1.19 (d, $J$ = 13.1 Hz, 1H), 0.86 (d, $J$ = 95.8 Hz, 3H); HRMS(ESI)(M-H)$^-$, m/z: calcd for $C_{24}H_{23}ClN_4O_2$ 434.1510, found 433.0834.

### 2.3.83. Tert-butyl 4–(4–(2-chlorobenzyl)-5-oxo-4,5-dihydroimidazo [1,2-a]quinazoline-2-carbonyl)piperazine-1-carboxylate (B14)

White solid; yield: 61.0%; *m.p.*: 234 °C; $^1$H NMR (400 MHz, $CDCl_3$) $\delta$ 8.51 (d, $J$ = 7.9 Hz, 1H), 8.19 (s, 1H), 7.89 (t, $J$ = 7.7 Hz, 1H), 7.70 (d, $J$ = 8.2 Hz, 1H), 7.59 (t, $J$ = 7.6 Hz, 1H), 7.46 (d, $J$ = 7.8 Hz, 1H), 7.24 (t, $J$ = 7.4 Hz, 1H), 7.16 (dd, $J$ = 18.9, 7.3 Hz, 2H), 5.69 (s, 2H), 4.12 (s, 2H), 3.73 (s, 2H), 3.47 (d, $J$ = 40.1 Hz, 4H), 1.54 (s, 9H); HRMS(ESI)(M + H)$^+$, m/z: calcd for $C_{27}H_{28}ClN_5O_4$ 521.1380, found 522.3588.

### 2.3.84. 4–(2-Chlorobenzyl)-N-(3-methoxyphenyl)-5-oxo-4,5-dihydroimidazo[1,2-a] quinazoline-2-carboxamide (B15)

White solid; yield: 69.0%; *m.p.*: 291 °C; $^1$H NMR (400 MHz, DMSO-$d_6$) $\delta$ 9.71 (s, 1H), 8.93 (s, 1H), 8.34 (d, $J$ = 7.9 Hz, 1H), 8.25 (d, $J$ = 7.0 Hz, 1H), 7.98 (t, $J$ = 7.0 Hz, 1H), 7.62 (t, $J$ = 7.0 Hz, 1H), 7.55 (d, $J$ = 8.4 Hz, 1H), 7.47 (s, 1H), 7.41 (d, $J$ = 7.2 Hz, 1H), 7.32 (s, 1H), 7.28 – 7.19 (m, 3H), 6.69 (d, $J$ = 7.2 Hz, 1H), 5.59 (s, 2H), 3.77 (s, 3H); HRMS(ESI)(M + H)$^+$, m/z: calcd for $C_{25}H_{19}ClN_4O_3$ 458.1146, found 459.0456.

### 2.3.85. 4–(2-Chlorobenzyl)-5-oxo-N-(3-(trifluoromethoxy)phenyl)-4,5-dihydroimidazo [1,2-a]quinazoline-2-carboxamide (B16)

White solid; yield: 79.0%; *m.p.*: 274 °C; $^1$H NMR (400 MHz, DMSO-$d_6$) $\delta$ 10.10 (s, 1H), 8.99 (s, 1H), 8.36 (d, $J$ = 7.8 Hz, 1H), 8.26 (d, $J$ = 7.5 Hz, 1H), 8.00 (d, $J$ = 14.0 Hz, 2H), 7.86 (d, $J$ = 7.5 Hz, 1H), 7.63 (t, $J$ = 6.8 Hz, 1H), 7.56 (d, $J$ = 7.3 Hz, 1H), 7.49 (t, $J$ = 7.1 Hz, 1H), 7.33 (d, $J$ = 5.7 Hz, 1H), 7.23 (d, $J$ = 6.9 Hz, 2H), 7.10 (d, $J$ = 7.7 Hz, 1H), 5.60 (s, 2H); HRMS(ESI)(M-H)$^-$, m/z: calcd for $C_{25}H_{16}ClF_3N_4O_3$ 512.0863, found 511.4709.

### 2.3.86. 4–(2-Chlorobenzyl)-N-(4-fluorobenzyl)-5-oxo-4,5-dihydroimidazo[1,2-a] quinazoline-2-carboxamide (B17)

White solid; yield: 61.0%; *m.p.*: 265 °C; $^1$H NMR (400 MHz, DMSO-$d_6$) $\delta$ 8.80 (s, 1H), 8.60 (t, $J$ = 5.9 Hz, 1H), 8.33 (d, $J$ = 8.3 Hz, 1H), 8.25 (d, $J$ = 7.8 Hz, 1H), 7.98 (t, $J$ = 7.4 Hz, 1H), 7.61 (t, $J$ = 7.5 Hz, 1H), 7.54 (d, $J$ = 7.9 Hz, 1H), 7.34 (dd, $J$ = 14.7, 8.5 Hz, 3H), 7.23 (t, $J$ = 7.2 Hz, 1H), 7.15 (t, $J$ = 8.1 Hz, 3H), 5.51 (s, 2H), 4.43 (d, $J$ = 6.1 Hz, 2H); HRMS(ESI)(M-H)$^-$, m/z: calcd for $C_{25}H_{18}ClFN_4O_2$ 460.1102, found 459.3676.

### 2.3.87. 4–(2-Chlorobenzyl)-5-oxo-N-(pyridin-2-ylmethyl)-4,5-dihydroimidazo[1,2-a] quinazoline-2-carboxamide (B18)

White solid; yield: 68.0%; *m.p.*: 280 °C; $^1$H NMR (400 MHz, DMSO-$d_6$) $\delta$ 8.83 (s, 1H), 8.62 (t, $J$ = 5.4 Hz, 1H), 8.52 (d, $J$ = 4.1 Hz, 1H), 8.33 (d, $J$ = 8.2 Hz, 1H), 8.26 (d, $J$ = 7.9 Hz, 1H), 7.98 (t, $J$ = 7.5 Hz, 1H), 7.76 (t, $J$ = 7.7 Hz, 1H), 7.62 (t, $J$ = 7.6 Hz, 1H), 7.55 (d, $J$ = 7.8 Hz, 1H), 7.35 – 7.26 (m, 3H), 7.21 (dd, $J$ = 14.7, 7.2 Hz, 2H), 5.53 (s, 2H), 4.57 (d, $J$ = 5.8 Hz, 2H); HRMS(ESI)(M-H)$^-$, m/z: calcd for $C_{24}H_{18}ClN_5O_2$ 443.1149, found 442.2751.

### 2.3.88. 4–(2-Chlorobenzyl)-N-(furan-2-ylmethyl)-5-oxo-4,5-dihydroimidazo[1,2-a] quinazoline-2-carboxamide (B19)

White solid; yield: 61.0%; *m.p.*: 294 °C; $^1$H NMR (400 MHz, DMSO-$d_6$) $\delta$ 8.80 (s, 1H), 8.39 (t, $J$ = 5.6 Hz, 1H), 8.32 (d, $J$ = 8.3 Hz, 1H), 8.24 (d, $J$ = 7.8 Hz, 1H), 7.97 (t, $J$ = 7.6 Hz, 1H), 7.61 (t, $J$ = 7.5 Hz, 1H), 7.54 (d, $J$ = 12.3 Hz, 2H), 7.32 (t, $J$ = 7.5 Hz, 1H), 7.22 (t, $J$ = 7.5 Hz, 1H), 7.15 (d, $J$ = 7.6 Hz, 1H), 6.39 (s, 1H), 6.24 (s, 1H), 5.50 (s, 2H), 4.44 (d, $J$ = 5.8 Hz, 2H). HRMS(ESI)(M + H)$^+$, m/z: calcd for $C_{23}H_{17}ClN_4O_3$ 432.0989, found 433.6325.

### 2.3.89. (S)-4–(2-chlorobenzyl)-5-oxo-N-(1-phenylethyl)-4,5-dihydroimidazo[1,2-a] quinazoline-2-carboxamide (B20)

White solid; yield: 66.0%; *m.p.*: 220 °C; $^1$H NMR (400 MHz, DMSO-$d_6$) $\delta$ 8.77 (s, 1H), 8.30 (d, $J$ = 8.3 Hz, 1H), 8.24 (t, $J$ = 8.7 Hz, 2H),

7.97 (t, $J = 7.6$ Hz, 1H), 7.61 (t, $J = 7.6$ Hz, 1H), 7.55 (d, $J = 7.9$ Hz, 1H), 7.40 (d, $J = 7.4$ Hz, 2H), 7.34 (t, $J = 7.4$ Hz, 3H), 7.28 − 7.16 (m, 3H), 5.55 (s, 2H), 5.23 − 5.13 (m, 1H), 1.51 (d, $J = 6.9$ Hz, 3H). HRMS(ESI)(M-H)$^-$, m/z: calcd for $C_{26}H_{21}ClN_4O_2$ 456.1353, found 455.7511.

### 2.3.90. (R)-4–(2-chlorobenzyl)-5-oxo-N-(1-phenylethyl)-4,5-dihydroimidazo[1,2-a] quinazoline-2-carboxamide (B21)

White solid; yield: 74.0%; *m.p.*: 220 °C; $^1$H NMR (400 MHz, DMSO-$d_6$) $\delta$ 8.76 (s, 1H), 8.30 (d, $J = 8.3$ Hz, 1H), 8.24 (t, $J = 8.8$ Hz, 2H), 7.97 (t, $J = 7.7$ Hz, 1H), 7.61 (t, $J = 7.6$ Hz, 1H), 7.54 (d, $J = 7.8$ Hz, 1H), 7.40 (d, $J = 7.4$ Hz, 2H), 7.34 (t, $J = 7.4$ Hz, 3H), 7.26 − 7.18 (m, 3H), 5.54 (s, 2H), 5.24 − 5.16 (m, 1H), 1.51 (d, $J = 6.9$ Hz, 3H); HRMS(ESI)(M-H)$^-$, m/z: calcd for $C_{26}H_{21}ClN_4O_2$ 456.1353, found 455.7570.

### 2.3.91. 4–(2-Chlorobenzyl)-5-oxo-N-(3-(trifluoromethyl)phenyl)-4,5-dihydroimidazo [1,2-a]quinazoline-2-carboxamide (B22)

White solid; yield: 64.0%; *m.p.*: 292 °C; $^1$H NMR (400 MHz, DMSO-$d_6$) $\delta$ 10.16 (s, 1H), 8.99 (s, 1H), 8.37 (d, $J = 8.3$ Hz, 1H), 8.30 − 8.24 (m, 2H), 8.14 (d, $J = 7.9$ Hz, 1H), 8.00 (t, $J = 7.4$ Hz, 1H), 7.65 − 7.55 (m, 3H), 7.46 (d, $J = 7.2$ Hz, 1H), 7.34 (t, $J = 7.2$ Hz, 1H), 7.22 (t, $J = 7.5$ Hz, 2H), 5.61 (s, 2H); HRMS(ESI)(M-H)$^-$, m/z: calcd for $C_{25}H_{16}ClF_3N_4O_2$ 496.0914, found 495.8887.

### 2.3.92. 4–(2-Chlorobenzyl)-N-(4-methoxybenzyl)-5-oxo-4,5-dihydroimidazo[1,2-a]quinazoline-2-carboxamide (B23)

White solid; yield: 64.0%; *m.p.*: 251 °C; $^1$H NMR (400 MHz, DMSO-$d_6$) $\delta$ 8.79 (s, 1H), 8.46 (t, $J = 5.9$ Hz, 1H), 8.32 (d, $J = 8.3$ Hz, 1H), 8.25 (d, $J = 7.9$ Hz, 1H), 7.98 (t, $J = 7.7$ Hz, 1H), 7.61 (t, $J = 7.6$ Hz, 1H), 7.54 (d, $J = 7.8$ Hz, 1H), 7.33 (t, $J = 7.4$ Hz, 1H), 7.23 (dd, $J = 13.0$, 8.1 Hz, 3H), 7.15 (d, $J = 7.4$ Hz, 1H), 6.89 (d, $J = 8.4$ Hz, 2H), 5.51 (s, 2H), 4.38 (d, $J = 6.0$ Hz, 2H), 3.74 (s, 3H); HRMS(ESI)(M + H)$^+$, m/z: calcd for $C_{25}H_{19}ClN_4O_3$ 472.13, found 473.14661.

### 2.3.93. 4–(2-Chlorobenzyl)-5-oxo-N-phenyl-4,5-dihydroimidazo[1,2-a]quinazoline-2-carboxamide (B24)

White solid; yield: 62.5%; *m.p.*: 2 8 4 ~ 286 °C; $^1$H NMR (400 MHz, DMSO-$d_6$) $\delta$ (ppm) = 9.77 (s, 1H), 8.96 (s, 1H), 8.36 (d, $J = 8.1$ Hz, 1H), 8.27 (d, $J = 7.3$ Hz, 1H), 8.00 (t, $J = 7.4$ Hz, 1H), 7.81 (d, $J = 7.4$ Hz, 2H), 7.63 (t, $J = 7.3$ Hz, 1H), 7.57 (d, $J = 7.5$ Hz, 1H), 7.39 − 7.32 (m, 3H), 7.23 (s, 2H), 7.13 (d, $J = 7.0$ Hz, 1H), 5.60 (s, 2H); HRMS(ESI)(M + H)$^+$, m/z: calcd for $C_{24}H_{17}ClN_4O_2$ 428.1040, found 429.0886.

### 2.3.94. 4–(2-Chlorobenzyl)-N-(2-hydroxyethyl)-5-oxo-4,5-dihydroimidazo[1,2-a] quinazoline-2-carboxamide (B25)

White solid; yield: 71.4%; *m.p.*: 3 0 0 ~ 302 °C; $^1$H NMR (400 MHz, DMSO-$d_6$) $\delta$ (ppm) = 8.79 (s, 1H), 8.33 (d, $J = 8.2$ Hz, 1H), 8.25 (d, $J = 7.8$ Hz, 1H), 7.97 (t, $J = 7.7$ Hz, 1H), 7.91 (t, $J = 5.4$ Hz, 1H), 7.61 (t, $J = 7.6$ Hz, 1H), 7.55 (d, $J = 7.9$ Hz, 1H), 7.34 (t, $J = 7.4$ Hz, 1H), 7.23 (t, $J = 7.5$ Hz, 1H), 7.15 (d, $J = 7.5$ Hz, 1H), 5.50 (s, 2H), 4.76 (t, $J = 5.2$ Hz, 1H), 3.49 (q, $J = 5.7$ Hz, 2H), 3.33 (d, $J = 6.4$ Hz, 2H); HRMS(ESI)(M + H)$^+$, m/z: calcd for $C_{20}H_{17}ClN_4O_3$ 396.0989, found 397.1064.

### 2.3.95. 4–(2-Chlorobenzyl)-N-cyclopropyl-5-oxo-4,5-dihydroimidazo [1,2-a] quinazoline-2-carboxamide (B26)

White solid; yield: 72.0%; *m.p.*: 2 7 5 ~ 277 °C; $^1$H NMR (400 MHz, $CDCl_3$) $\delta$ (ppm) = 8.47 (d, $J = 7.9$ Hz, 1H), 8.18 (s, 1H), 7.89 (t, $J = 7.7$ Hz, 1H), 7.70 (d, $J = 8.2$ Hz, 1H), 7.58 (t, $J = 7.6$ Hz, 1H), 7.47 (d, $J = 7.8$ Hz, 1H), 7.26 (t, $J = 7.7$ Hz, 1H), 7.19 (t, $J = 7.0$ Hz, 2H), 7.11 (d, $J = 7.6$ Hz, 1H), 5.67 (s, 2H), 2.94 − 2.91 (m, 1H), 0.90 (q, $J = 6.4$ Hz, 2H), 0.70 (d, $J = 6.3$ Hz, 2H); HRMS(ESI)(M + H)$^+$, m/z: calcd for $C_{21}H_{17}ClN_4O_2$ 392.1040, found 393.2096.

### 2.3.96. 4–(2-Chlorobenzyl)-N-(2,4-dimethylphenyl)-5-oxo-4,5-dihydroimidazo[1,2-a] quinazoline-2-carboxamide (B27)

White solid; yield: 65.9%; *m.p.*: 2 8 6 ~ 288 °C; $^1$H NMR (400 MHz, DMSO-$d_6$) $\delta$ (ppm) = 9.29 (s, 1H), 8.93 (s, 1H), 8.35 (d, $J = 6.5$ Hz, 1H), 8.29 (d, $J = 8.5$ Hz, 1H), 8.02 − 7.97 (m, 1H), 7.60 (dd, $J = 21.0$, 11.3 Hz, 3H), 7.33 (s, 1H), 7.26 (s, 2H), 7.06 (dd, $J = 19.0$, 5.2 Hz, 2H), 5.57 (s, 2H), 2.29 (s, 3H), 2.18 (s, 3H); HRMS(ESI)(M + H)$^{+,}$ m/z: calcd for $C_{26}H_{21}ClN_4O_2$ 456.1353, found 457.1425.

### 2.3.97. 4–(2-Chlorobenzyl)-N-cyclohexyl-5-oxo-4,5-dihydroimidazo [1,2-a]quinazoline-2-carboxamide (B28)

White solid; yield: 73.2%; *m.p.*: 2 5 0 ~ 252 °C; $^1$H NMR (400 MHz, $CDCl_3$) $\delta$ (ppm) = 8.48 (d, $J = 7.8$ Hz, 1H), 8.15 (s, 1H), 7.88 (t, $J = 7.6$ Hz, 1H), 7.69 (d, $J = 8.2$ Hz, 1H), 7.57 (t, $J = 7.6$ Hz, 1H), 7.48 (d, $J = 7.8$ Hz, 1H), 7.25 (d, $J = 8.2$ Hz, 1H), 7.22 − 7.15 (m, 2H), 7.06 (d, $J = 8.1$ Hz, 1H), 5.70 (s, 2H), 2.04 (d, $J = 10.7$ Hz, 2H), 1.80 (d, $J = 13.2$ Hz, 2H), 1.67 (s, 1H), 1.59 (s, 1H), 1.47 (q, $J = 12.0$, 9.7 Hz, 2H), 1.32 (dt, $J = 23.0$, 12.2 Hz, 3H); HRMS(ESI)(M + H)$^+$, m/z: calcd for $C_{24}H_{23}ClN_4O_2$ 434.1510, found 435.1583.

### 2.3.98. 4–(2-Chlorobenzyl)-N-(2-methoxyphenyl)-5-oxo-4,5-dihydroimidazo[1,2-a] quinazoline-2-carboxamide (B29)

White solid; yield: 61.8%; *m.p.*: 2 6 2 ~ 264 °C; $^1$H NMR (400 MHz, DMSO-$d_6$) $\delta$ (ppm) = 9.47 (s, 1H), 8.97 (s, 1H), 8.37 (d, $J = 8.3$ Hz, 2H), 8.33 (d, $J = 7.8$ Hz, 1H), 8.01 (t, $J = 7.8$ Hz, 1H), 7.66 (t, $J = 7.6$ Hz, 1H), 7.60 (d, $J = 7.9$ Hz, 1H), 7.35 (t, $J = 7.6$ Hz, 1H), 7.32 − 7.25 (m, 2H), 7.11 (d, $J = 3.8$ Hz, 2H), 7.00 (dt, $J = 9.0$, 4.6 Hz, 1H), 5.56 (s, 2H), 3.90 (s, 3H); HRMS(ESI)(M + H)$^+$, m/z: calcd for $C_{25}H_{19}ClN_4O_3$ 458.1146, found 459.1221.

### 2.3.99. 4–(2-Chlorobenzyl)-N-((2R,6S)-2,6-dimethylcyclohexyl)-5-oxo-4,5-dihydroimidazo[1,2-a]quinazoline-2-carboxamide (A30)

White solid; yield: 61.1%; *m.p.*: 2 0 5 ~ 207 °C; $^1$H NMR (400 MHz, $CDCl_3$) $\delta$ (ppm) = 8.49 (d, $J = 7.9$ Hz, 1H), 8.13 (s, 1H), 7.88 (t, $J = 7.7$ Hz, 1H), 7.69 (d, $J = 8.1$ Hz, 1H), 7.57 (t, $J = 7.6$ Hz, 1H), 7.44 (d, $J = 7.8$ Hz, 1H), 7.23 (t, $J = 7.5$ Hz, 1H), 7.15 (t, $J = 7.4$ Hz, 1H), 7.08 (d, $J = 7.6$ Hz, 1H), 5.70 (s, 2H), 1.88 (d, $J = 13.1$ Hz, 1H), 1.72 (s, 4H), 1.64 (s, 3H), 1.55 (s, 1H), 1.25 (d, $J = 37.6$ Hz, 4H); HRMS(ESI)(M + H)$^+$, m/z: calcd for $C_{22}H_{27}ClN_4O_2$ 462.1863, found 463.3052.

### 2.3.100. N-Benzyl-4-(2-chlorobenzyl)-5-oxo-4,5-dihydroimidazo[1,2-a] quinazoline-2-carboxamide (B31)

White solid; yield: 63.2%; *m.p.*: 2 7 0 ~ 272 °C; $^1$H NMR (400 MHz, DMSO-$d_6$) $\delta$ (ppm) = 8.85 (s, 1H), 8.60 (s, 1H), 8.34 (d, $J = 7.8$ Hz, 1H), 8.25 (d, $J = 6.8$ Hz, 1H), 7.98 (t, $J = 7.2$ Hz, 1H), 7.62 (t, $J = 7.5$ Hz, 1H), 7.54 (d, $J = 7.5$ Hz, 1H), 7.46 (d, $J = 6.7$ Hz, 1H), 7.26 (dd, $J = 34.6$, 11.2 Hz, 6H), 5.53 (s, 2H), 4.52 (s, 2H);

HRMS(ESI)$(M+H)^+$, m/z: calcd for $C_{25}H_{19}ClN_4O_2$ 442.1197, found 443.1271.

#### 2.3.101. Tert-butyl (1–(4-(2-chlorobenzyl)-5-oxo-4,5-dihydroimidazo[1,2-a] quinazoline-2-carbonyl)piperidin-4-yl)carbamate (B32)

White solid; yield: 66.0%; *m.p.*: 2 6 8 ~ 270 °C; $^1$H NMR (400 MHz, $CDCl_3$) $\delta$ (ppm) = 8.45 (d, $J$ = 7.6 Hz, 1H), 8.11 (s, 1H), 7.82 (d, $J$ = 7.7 Hz, 1H), 7.65 (d, $J$ = 8.0 Hz, 1H), 7.54 (t, $J$ = 7.3 Hz, 1H), 7.40 (d, $J$ = 7.4 Hz, 1H), 7.19 (t, $J$ = 8.6 Hz, 2H), 7.10 (d, $J$ = 11.8 Hz, 2H), 5.65 (s, 2H), 5.30 (s, 1H), 2.92 (d, $J$ = 29.0 Hz, 4H), 1.55 (s, 9H), 1.37 (s, 4H); HRMS(ESI)$(M+H)^+$, m/z: calcd for $C_{28}H_{30}ClN_5O_4$ 535.1986, found 536.2062.

### 2.4. Pharmacology

#### 2.4.1. In vitro *enzyme inhibition assay*

Self-isolated and purified SHP2 was used as the target protein, and SHP244 was used as a positive control to evaluate the enzyme activity of the target compound. Add the test compound solution and the positive control solution to the test plate at the prepared concentrations of 100 μM and 200 μM, 5 μL to each volume, add 2 Wells to each solution of each concentration, then add 5 μL of SHP2 enzyme solution (0.5 nM), gently shake the test plate to mix the solution, incubate at room temperature for 10 min. Add 5 μL 2 P-IRS-1 peptide solution (0.5 μM) and react for 30 min. Add 5 μL metabolic substrate desizing solution (0.5 nM) and incubate for 30 min. Add 5 μL BPV solution (160 μM) to quench the reaction. Measure the absorbance value (a value) of each well with a microplate reader at the excitation wavelength of 340 nm and emission wavelength of 450 nm, and calculate the enzyme activity inhibition rate (%) based on the average absorbance of each group of 2 replicate wells:

$$\%\,\text{Inhibition} = (\text{OD value of normal hole} - \text{OD value of dosing hole}) / \text{OD value of normal hole} \times 100$$

#### 2.4.2. *Mtt assay* in vitro

Human melanoma cell line A375 was used as the test cell line, and SHP244 and sorafenib were used as positive controls. The MTT assay was used to determine the *in vitro* inhibition rate of the compound at a concentration of 100 μM and 72 h. The cells in the logarithmic growth phase were uniformly inoculated into a 96-well plate ($10^5$ cells/well), placed in a 5% $CO_2$ incubator, and cultured at 37 °C for 12 h. After the cells adhered to the wall, they were applied to 3 replication Wells in each set, placed in a 5% $CO_2$ incubator and incubated at 37 °C for 72 h. After inhaling and discarding all Wells, 100 μL DMEM high glucose medium was added to each well, then 20 μL MTT solution (5 mg/mL) was added to the dark, again placed in a 5% $CO_2$ incubator, and incubated in darkness at 37 °C for 4 h. Measure the absorbance value (a value) of each well with a microplate reader at a wavelength of 490 nm, and calculate the cell proliferation inhibition rate based on the average absorbance of each group of 3 replicate wells:

$$\text{Inhibition rate of cell proliferation}(\%) = 1 - \text{OD value of dosing hole}/\text{OD value of normal hole} \times 100\%\ \textit{Metabolic}$$

#### 2.4.3. *Metabolic stability of liver microsomes assay of compound A22* in vitro

**SHP244** was used as a positive control to evaluate the metabolic stability of the target product **A22** in human and rat liver microsome. Standard stock solution (100 μM, 99% ACN) of test compounds were prepared in dimethyl sulfoxide (DMSO) and acetonitrile (ACN). The Standard stock solution (2 μL) were preincubated with phosphate buffer (100 μL) of microsomes (human microsome: HLM, Corning, lot No. 38296; Rat microsome: RLM, Xenotech, Lot No. 1910100) for 10 min at 37 °C. The incubation system was then activated by initiation factors (98 μL, 10 mM) (NADPH, Chem-Impex International, Cat. No. 00616). After incubation for different time points (0, 5, 15, 30, 45, and 60 min) at 37 °C, cold (4 °C) ACN (containing 200 ng/mL tolbutamide and 200 ng/mL labetalol) was added to quench the reaction. All sampling plates were shaken for 10 min, then centrifuged at 4000 rpm for 20 min at 4 °C, and the supernatants (300 μL) were analysed using LC-MS/MS.

$T_{1/2}$ (min): half-life

CL (μL/min/mg): intrinsic clearance = 0.693/$T_{1/2}$/microsome protein per mL (mg).

## 3. Results and discussion

### 3.1. Design of inhibitors

Design of "Latch" site Inhibitors of SHP2 (Figure 2) (1): (a) According to the interaction between compound SHP244 and protein, it can be seen that the triazole ring interacts with the main chain, side chain and protein residues, so the triazole ring is retained to ensure the activity of the compound; (b) Replace the benzene ring structure with a nitrogen-containing side chain in order to enhance the binding effect with the receptor; (c) The chlorobenzene ring is replaced with an aromatic ring containing

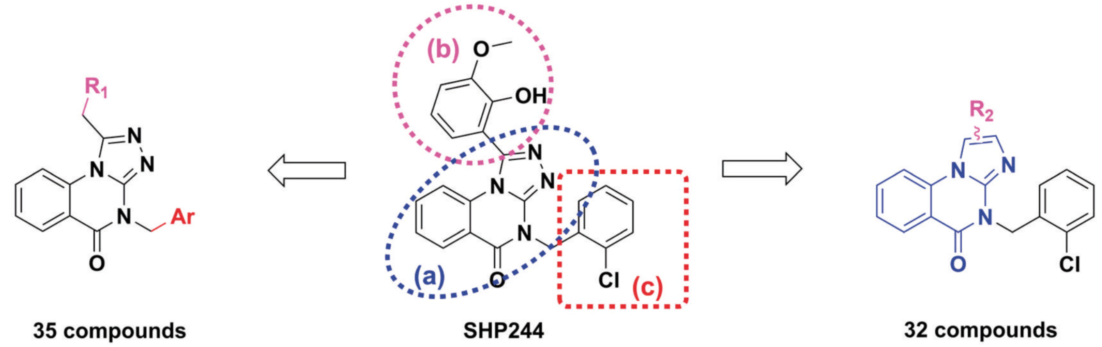

Figure 2. Structure of target compounds.

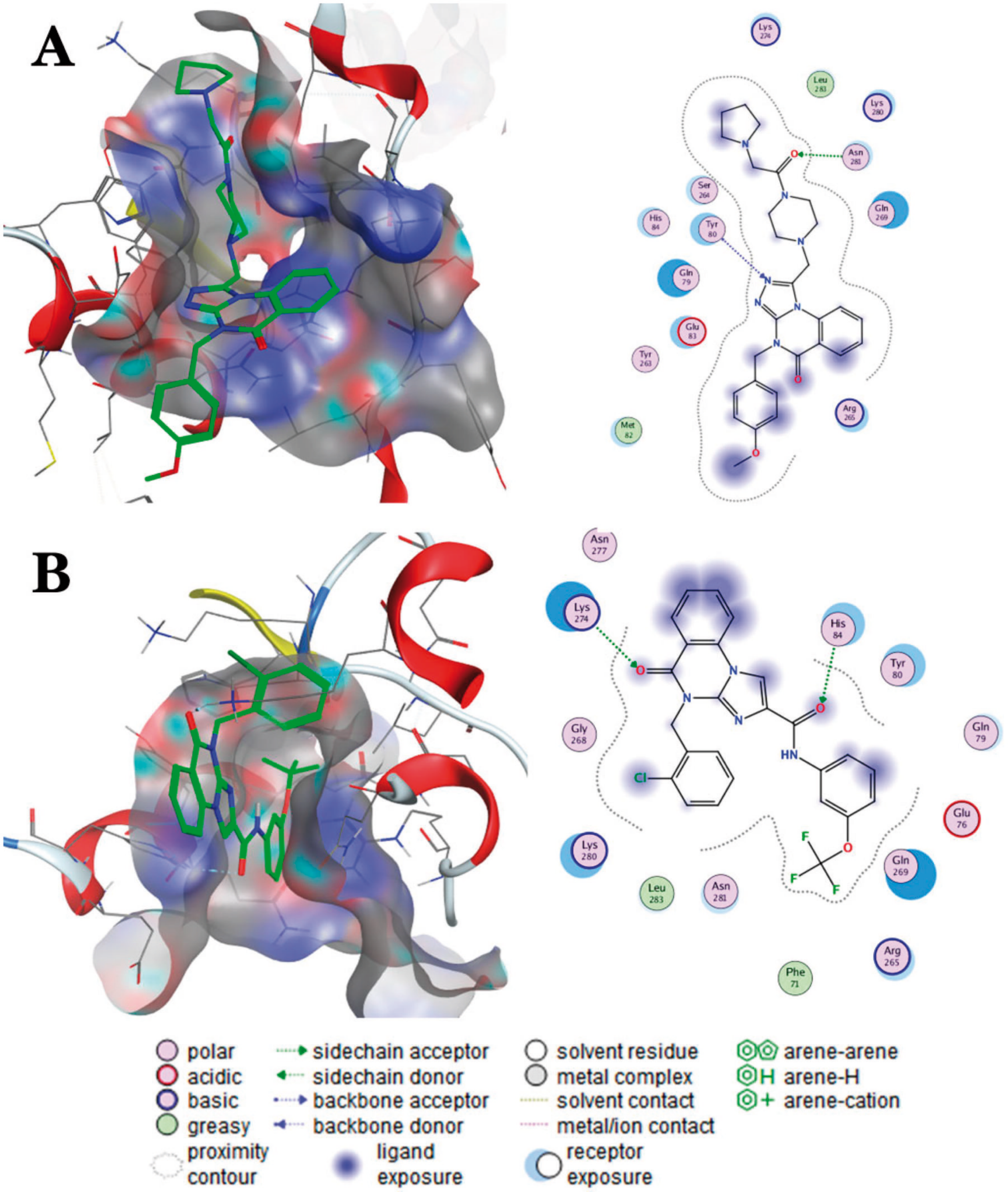

**Figure 3.** Pose and interations of compounds **A35** (**A**) and **B16** (**B**) with SHP2 protein.

different substituents to investigate the effect of this part of the structure on the activity. (2): (a) Under the premise of ensuring its similarity in the spatial structure, replace its triazoloquinazolinone ring with an imidazoquinazolinone ring; (b) The introduction of nitrogen-containing side chains at different positions of the imidazole ring in order to enhance the binding to the receptor; (c) retain the chlorophenyl ring that can interact with amino acid residues Q269 and Q79 to ensure that it can interact with the protein through hydrogen bonds.

### 3.2. Docking studies

As shown in Figure 3, the **A35** of the triazoloquinazolinone forms hydrogen with the amino acid residues Asn281 and Tyr80, respectively. **B16** of the imidazoquinazolinones forms hydrogen bond interactions with Lys274 and His84, respectively. The docking results show that the imidazoquinazolinone core has a certain affinity for SHP2 protein at the same time, the branch chain design of the two types of target compound structures is reasonable and worthy of further synthetic research.

### 3.3. Chemistry

Schemes 1 and 2 summarise the synthetic routes of target compounds **A1–A35** and **B1–B32**. In Scheme 1, the nucleophilic substitution reaction between isatoic anhydride and the corresponding arylmethyl primary amine yields compounds **6a–6e**, and then the disulphide bond is introduced via carbon

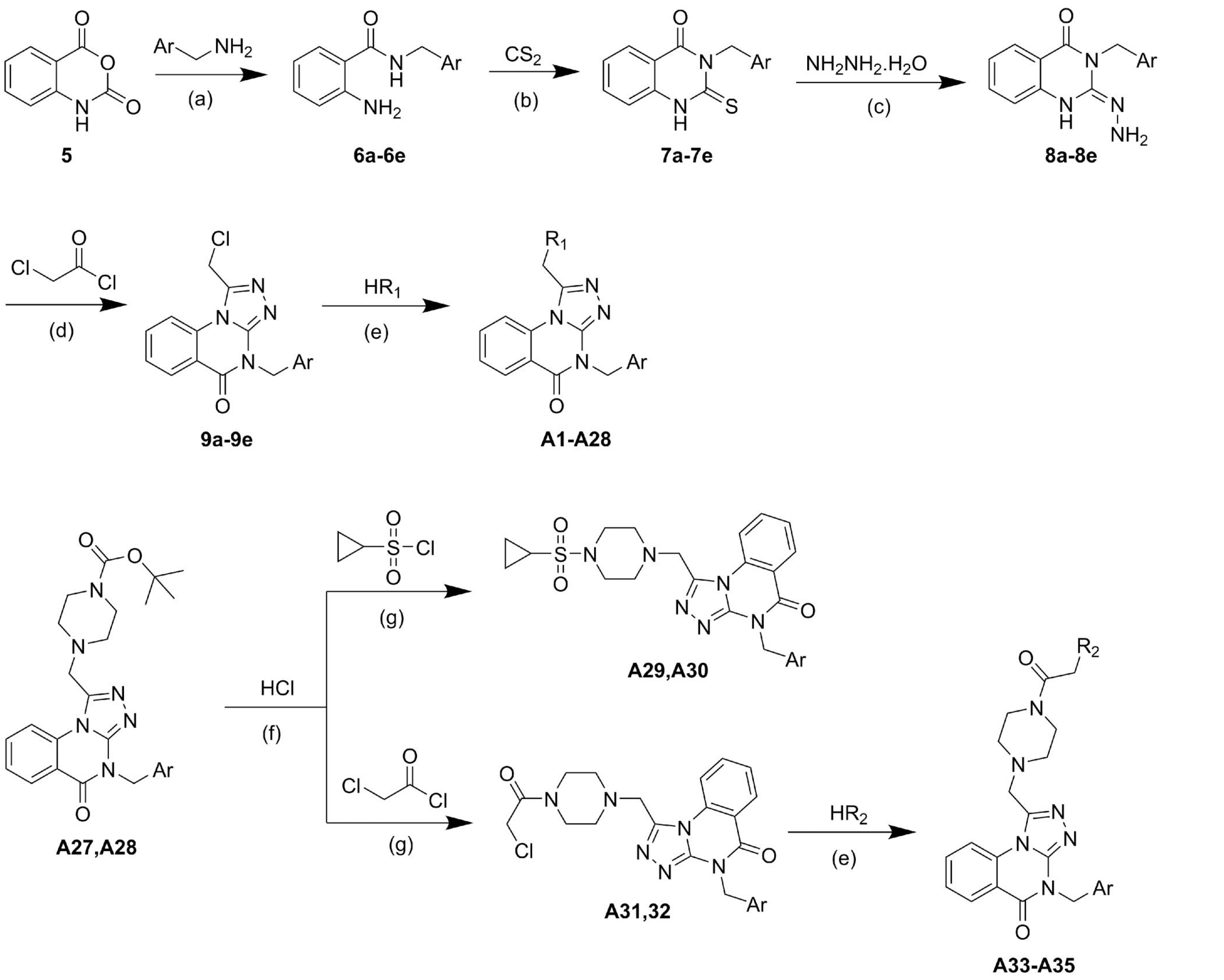

6a,7a, 8a, 9a,A1-A6: Ar= furan-2-ylmethyl
6b, 7b, 8b, 9b, A7-A10: Ar= thiophen-2-ylmethyl
6c, 7c, 8c, 9c, A11: Ar= 2-chlorobenzyl
6d, 7d, 8d, 9d, A12-A16, A27, A29, A31, A33: Ar= 4-fluorobenzyl
6e, 7e, 8e, 9e, A17-A26, A28, A30, A32, A34, A35: Ar=4-methoxybenzyl

**Scheme 1.** Synthetic route of target compounds: (a) EA, 40 °C, 2 h (b) KOH, EtOH, 55 °C, 16 h (c) *i*-PrOH, 90 °C, 8 h (d) DMF, 100 °C, 5 h (e) $K_2CO_3$, $CH_3CN$, 50 °C, 4 h (f) $H_2O$, rt, 4 h (g) TEA, DCM, 0 °C, 0.5 h disulphide under potassium hydroxide conditions to yield compounds **7a–7e**[32–36]. Compounds **7a–7e** and hydrazine hydrate are refluxed in isopropanol, undergo nucleophilic addition to obtain compounds **8a–8e**, and then cyclize with chloroacetyl chloride to obtain compounds **9a-9e**[37]. Compounds **9a-9e** undergo nucleophilic substitution with different secondary amine reagents under basic conditions to obtain compounds **A1–A35**. Next, the Boc group of compounds **A27** and **A28** were removed by hydrochloric acid to obtain their hydrochloride, and they were substituted with chloroacetyl chloride and cyclopropylsulfonyl chloride to obtain compounds **A29–A32**, respectively. Among them, compounds **A31** and **A32** are further substituted to obtain compounds **A33-A35**. In Scheme 2, compound **10** is prepared by halogenation reaction between compound **7c** and $PCl_5$[38]. Next, the imidazoquinazolinone structure in compound **11** is obtained by cyclisation of compound **10** with aminoacetal dimethanol. Subsequently, compound **12** was prepared by the Vilsmeier-Hacck reaction of compound **11** with DMF and $POCl_3$, and then reductive amination with a secondary amine reagent to obtain compounds **B1–B5**[39]. Compounds **B6–B32** are obtained by substituting compound **10** with *p*-methoxybenzylamine and removing the benzyl group, then cyclizing with ethyl bromopyruvate and condensing with amine compounds[40].

### 3.4. Biological activity

#### 3.4.1. In vitro enzymatic assays

The inhibitory activities of all synthetic triazoloquinolinone derivatives and imidazoquinazolone derivatives against SHP2 protease at 100 μM and 200 μM were evaluated. SHP244 was used as a positive control, the results are expressed as inhibition values and are summarised in Tables 1 and 2. These values are the average of at least three independent experiments.

As shown in Tables 1 and 2, most of the compounds have certain inhibitory activity against SHP2 protease. Compared with

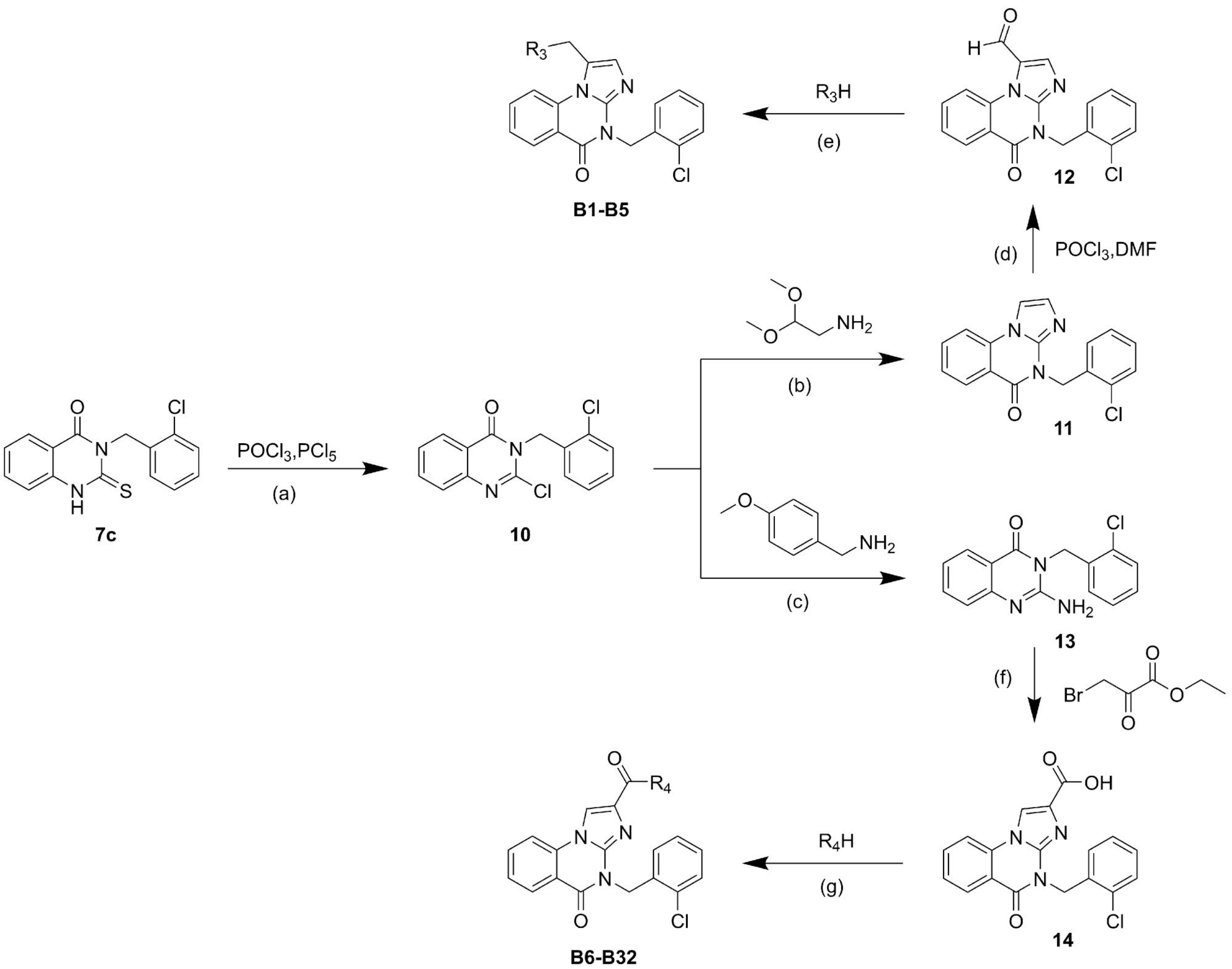

**Scheme 2.** Synthetic route of target compounds: (a) 80 °C, 4 h (b) TEA, EtOH, 80 °C, 1 h (c) $K_2CO_3$, $CH_3CN$, 80 °C, 8 h (d) DMF, 90 °C, 5 h (e) (i) AcOH, DCM, 0 °C, 15 min; ii. $NaBH(OAc)_3$, rt, 18 h (f) i. $Na_2CO_3$, EtOH, 85 °C, 7 h; (ii) NaOH, $H_2O$, 100 °C, 1 h (g) TEA, PyBOP, DMF, rt, 2 h.

SHP244, some compounds show similar or higher sensitivity to SHP2, which indicates that the modification of the three parts of SHP244 has a profound influence on the activity. The enzyme activity analysis showed that for the triazoloquinazolinone derivatives, the inhibitory activity of the compound against SHP2 protease is generally higher than that of the aromatic heterocycle when part (c) is substituted for the benzene ring. It is noteworthy that when part (c) is *p*-methoxyphenyl, the activity is significantly better than *p*-fluorophenyl. Moreover, when the side chain of part (b) is extended, the inhibitory activity of this type of compound is significantly improved. For example, compound **A30** (28.20% inhibition) and compound **A34** (26.38% inhibition) showed better SHP2 protein inhibitory activity than comparable structures at 100 μM, and better than SHP244 (23.52% inhibition).

For imidazoquinazolinone derivatives, although part of the core structure of SHP244 (a) is replaced, these compounds still show good SHP2 protease inhibitory activity, such as compound **B1** (22.14% inhibition) and compound **B11** (29.81% inhibition) showed better inhibition of SHP2 protein activity than SHP244 (14.95% inhibition) at 100 μM. The enzyme activity analysis showed that although the introduction of nitrogen-containing side chains at different positions of the imidazole ring in part (b) showed good inhibitory activity, the side chain structure has a momentous influence on the activity. When (b) part of the side chain is aniline amide, the *meta*-substituent on the benzene ring is an electron-donating group, which is beneficial to inhibit the activity, and when the substituent is a methoxy group, the order of activity is *meta* > *para* > unsubstituted > *ortho*, namely **B15** (24.27% inhibition) > **B23** (20.34% inhibition) > **B24** (12.46% inhibition) > **B29** (4.42% inhibition).

Compared with SHP2 catalytic site inhibitors, the enzyme inhibitory activity of the target compounds is relatively low. However, SHP2 allosteric inhibitors, as an effective strategy to overcome drug resistance caused by SHP2 mutations, are usually used in combination with orthosteric inhibitors to delay the emergence of drug resistance mutations in target proteins. Therefore, even with low enzymatic activity, it has the potential to work synergistically with orthosteric drugs to overcome drug resistance and improve therapeutic efficacy by fine-tuning orthosteric sites or modulating pathways associated with drug resistance[41–45]. Furthermore, the combination of "Latch" site inhibitors with "Tunnel" site inhibitors also enhanced $IC_{50}$, and dual occupancy of both allosteric sites was observed through the X-ray structure[28].

Above these, the structure-activity (SARs) relationship can be obtained through the kinase inhibitory activity of two types of target compounds: (I) Substituting imidazoquinazolinone core for triazoloquinazolinone core also has good SHP2 protein kinase Inhibitory activity; (II) For triazoloquinazolinone derivatives, when part (c) is a substituted benzene ring, the inhibitory activity of the compound against SHP2 protease is higher than that of aromatic heterocyclic ring, and the p-methoxyphenyl group is better than p-fluorophenyl group; (III) For triazoloquinazolinone derivatives,

**Table 1.** *In vitro* enzyme inhibitory activities of target compounds on SHP2 protein at 100 μM and 200 μM.

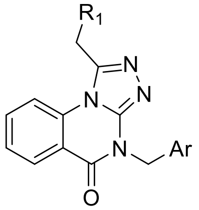

| Compd. | $R_1$ | Ar | % Inhibition[a] 100 μM | % Inhibition[a] 200 μM |
|---|---|---|---|---|
| **A1** |  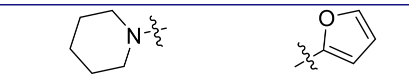 | | −19.08 | −54.89 |
| **A2** |  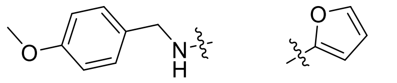 | | −31.33 | −40.46 |
| **A3** |  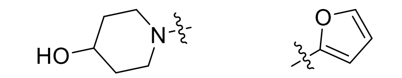 | | 22.60 | −2.61 |
| **A4** |  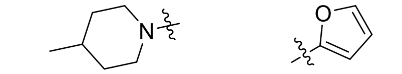 | | 7.69 | −63.63 |
| **A5** |  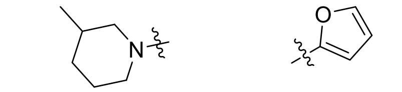 | | 3.77 | −64.31 |
| **A6** |  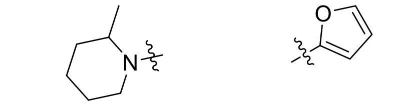 | | 10.61 | 4.67 |
| **A7** |  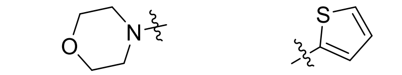 | | 9.36 | 10.33 |
| **A8** |  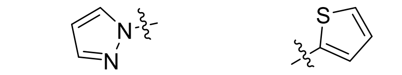 | | 14.40 | 16.30 |
| **A9** |  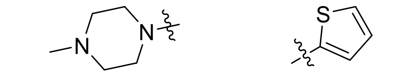 | | 12.15 | 17.95 |
| **A10** |  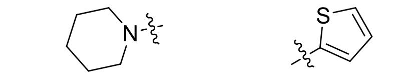 | | 5.92 | 7.95 |
| **A11** |  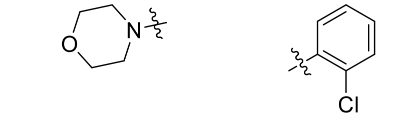 | | 18.40 | 19.91 |
| **A12** |  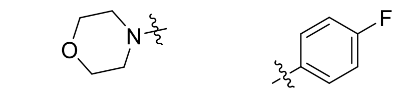 | | 18.23 | 20.59 |
| **A13** |  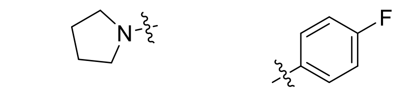 | | 12.43 | 13.39 |
| **A14** |  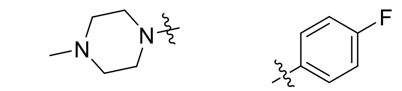 | | 15.02 | 18.01 |
| **A15** |  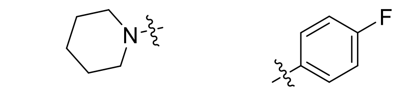 | | 8.77 | −1.08 |
| **A16** |  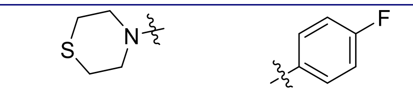 | | 15.58 | 18.20 |
| **A17** |  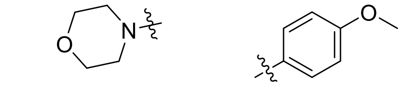 | | 11.11 | – |
| **A18** |  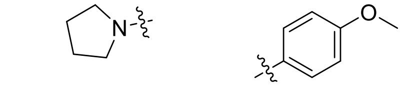 | | 15.39 | 22.83 |
| **A19** |  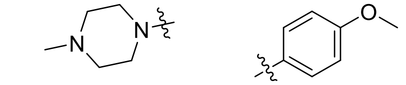 | | 20.40 | 27.37 |
| **A20** |  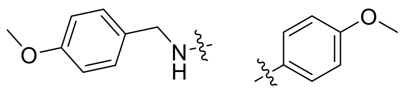 | | 6.94 | 10.58 |
| **A21** |  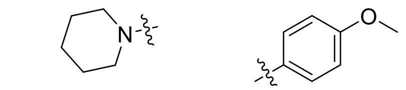 | | 0.59 | −7.39 |
| **A22** |  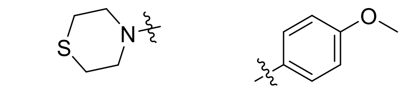 | | 4.96 | −11.19 |
| **A23** |  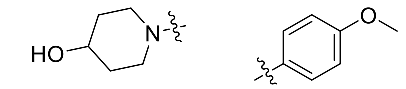 | | 14.40 | 2.69 |
| **A24** |  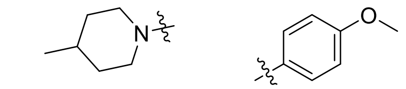 | | 11.27 | −4.83 |
| **A25** |  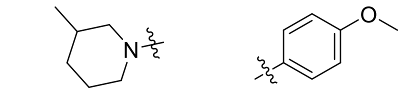 | | 19.41 | 7.08 |
| **A26** |  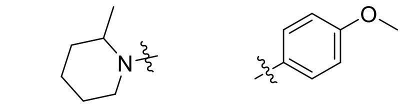 | | 12.58 | −1.00 |
| **A27** |  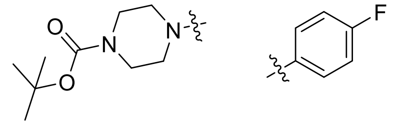 | | 11.69 | 14.16 |
| **A28** |  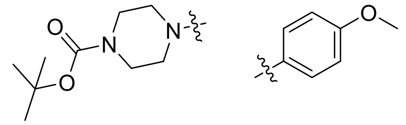 | | 13.88 | 18.82 |
| **A29** |  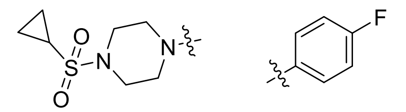 | | 0.16 | 5.73 |
| **A30** |  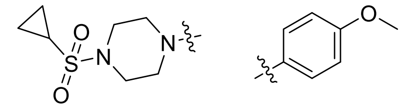 | | 28.20 | 32.79 |
| **A31** |  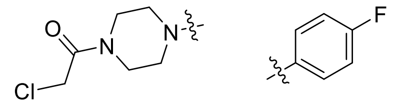 | | 14.04 | 16.04 |

(*continued*)

**Table 1.** Continued.

(*continued*)

**Table 1.** Continued.

| Compd. | $R_1$ | Ar | % Inhibition[a] 100 μM | 200 μM |
|---|---|---|---|---|
| **A32** |  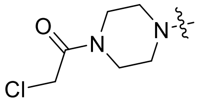 |  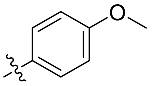 | 18.79 | 25.02 |
| **A33** |  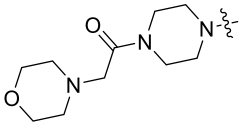 |  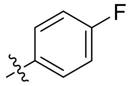 | 19.22 | 20.23 |
| **A34** |  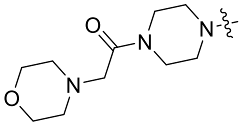 |  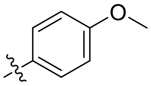 | 26.38 | 28.74 |
| **A35** |  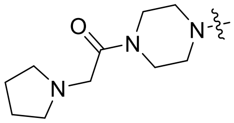 |  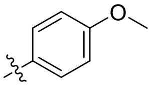 | 15.29 | 30.47 |
| **SHP244**[b] | | | 23.52 | 75.31 |

[a]Data presented is the mean ± SD value of three independent determinations.
[b]Used as positive control.

part (b) of the side chain extension can increase the activity; (IV) part (b) of the introduction of different side chains at different positions of the imidazole ring has an important effect on the activity; (V) For imidazoquinazolinone derivatives, when part of the side chain of (b) is aniline amide, the meta-substituent on the benzene ring is an electron-donating group, which is beneficial to inhibit the activity. And when the substituent is a methoxy group, the order of activity is *meta* > *para* > unsubstituted > *ortho* (Table 2)

### *3.4.2.* In vitro *cytotoxicity*

In order to further study the anti-tumour activity of the target compound, SHP244 and sorafenib were used as positive controls, and the MTT method was used to evaluate the *in vitro* cytotoxicity of the target compounds 100 μM on the melanoma cell line A375. As shown in Table 3, compared with SHP244 and sorafenib, most of the compounds showed significant cytotoxicity to A375, such as compound **A5** (27.02% inhibition), **A21** (29.60% inhibition), **B1** (76.15% inhibition) and **B22** (27.93% inhibition) showed effective activity. For this reason, the $IC_{50}$ values of some compounds were further determined, as shown in Table 4. Among them, the $IC_{50}$ values of compounds **B21**, **B23** and **B30** were determined to be 96.35 μM, 35.89 μM and 109.24 μM, respectively. These data once again show the further research value of this class of compounds. It is worth noting that compared with SHP244, the tumour cell activity of target compounds is significantly better than enzyme activity, so it is speculated that the target compound may have other targeting effects besides SHP2.

**Table 2.** *In vitro* enzyme inhibitory activities of target compounds on SHP2 protein at 100 μM and 200 μM.

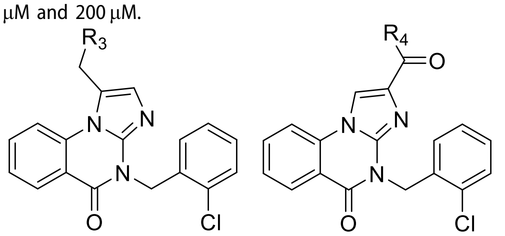

| Compd. | $R_3$ | $R_4$ | % Inhibition[a] 100 μM | 200 μM |
|---|---|---|---|---|
| **B1** |  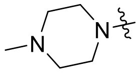 | | 22.14 | – |
| **B2** |  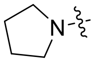 | | 16.12 | 1.91 |
| **B3** |  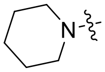 | | 12.02 | 8.14 |
| **B4** |  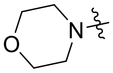 | | 12.23 | 6.61 |
| **B5** |  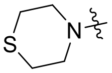 | | 7.56 | – |
| **B6** | |  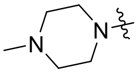 | 10.79 | – |
| **B7** | |  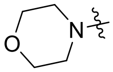 | 1.80 | 3.23 |
| **B8** | |  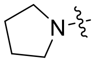 | 5.47 | 9.93 |
| **B9** | |  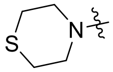 | 15.29 | 4.96 |
| **B10** | |  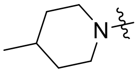 | 20.42 | – |
| **B11** | |  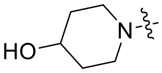 | 29.81 | 10.56 |
| **B12** | |  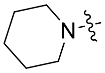 | 19.59 | 11.28 |
| **B13** | |  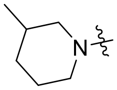 | 11.82 | – |

(*continued*)

Table 2. Continued.

| Compd. | $R_3$ | $R_4$ | % Inhibition[a] 100 μM | 200 μM |
|---|---|---|---|---|
| **B14** | |  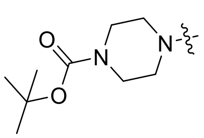 | 6.19 | – |
| **B15** | |  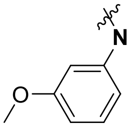 | 24.27 | 2.43 |
| **B16** | |  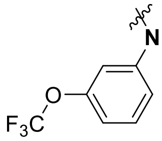 | 13.62 | 7.86 |
| **B17** | |  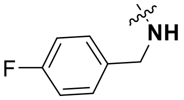 | 23.23 | 6.83 |
| **B18** | |  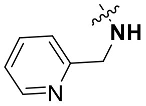 | 19.26 | – |
| **B19** | |  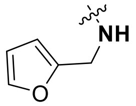 | 13.46 | 20.71 |
| **B20** | |  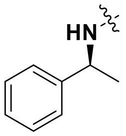 | 14.16 | 4.50 |
| **B21** | |  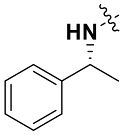 | 1.85 | 1.52 |
| **B22** | |  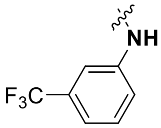 | 9.64 | 3.96 |
| **B23** | |  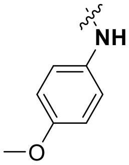 | 20.34 | 4.68 |

(*continued*)

Table 2. Continued.

| Compd. | $R_3$ | $R_4$ | % Inhibition[a] 100 μM | 200 μM |
|---|---|---|---|---|
| **B24** | |  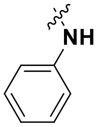 | 12.46 | 20.35 |
| **B25** | |  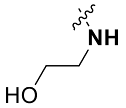 | 12.92 | 0.50 |
| **B26** | |  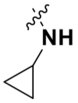 | 12.23 | −1.59 |
| **B27** | |  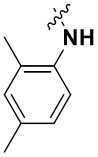 | 12.92 | 0.35 |
| **B28** | |  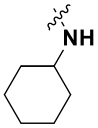 | 8.91 | 0.54 |
| **B29** | |  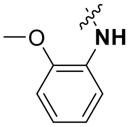 | 4.42 | 14.74 |
| **B30** | |  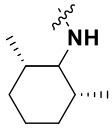 | 12.22 | −21.41 |
| **B31** | |  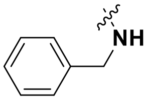 | 16.43 | −9.62 |
| **B32** | |  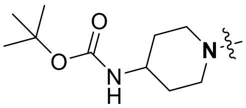 | 10.36 | −12.47 |
| **SHP244**[b] | | | 23.52 | 75.31 |

[a]Data presented is the mean ± SD value of three independent determinations.
[b]Used as positive control.

Table 3. Inhibition rate of target compounds on melanoma cells A375 *in vitro* at 100 μM.

| Compd. | % Inhibition[a] | Compd. | % Inhibition[a] |
|---|---|---|---|
| **A1** | 10.90 | **B1** | 76.15 |
| **A2** | 19.45 | **B2** | – |
| **A3** | 11.13 | **B3** | – |
| **A4** | 3.94 | **B4** | – |
| **A5** | 27.02 | **B5** | – |
| **A6** | 16.96 | **B6** | 17.60 |
| **A7** | 10.68 | **B7** | 16.81 |
| **A8** | – | **B8** | 15.04 |
| **A9** | 15.50 | **B9** | 15.92 |
| **A10** | 11.21 | **B10** | – |
| **A11** | – | **B11** | – |
| **A12** | – | **B12** | – |
| **A13** | – | **B13** | – |
| **A14** | – | **B14** | – |
| **A15** | 5.45 | **B15** | 24.42 |
| **A16** | −0.30 | **B16** | 24.08 |
| **A17** | – | **B17** | 20.14 |
| **A18** | 24.34 | **B18** | 11.23 |
| **A19** | – | **B19** | 20.61 |
| **A20** | 7.53 | **B20** | 21.89 |
| **A21** | 29.60 | **B21** | 18.13 |
| **A22** | 24.15 | **B22** | 27.93 |
| **A23** | 18.70 | **B23** | 20.14 |
| **A24** | 16.05 | **B24** | 16.82 |
| **A25** | 7.65 | **B25** | 14.98 |
| **A26** | 0.30 | **B26** | 15.23 |
| **A27** | 7.36 | **B27** | 12.47 |
| **A28** | – | **B28** | 5.02 |
| **A29** | – | **B29** | 22.09 |
| **A30** | 8.58 | **B30** | 24.77 |
| **A31** | – | **B31** | 12.22 |
| **A32** | 16.90 | **B32** | 20.59 |
| **A33** | 17.95 | | |
| **A34** | – | **SHP244b** | 13.81 |
| **A35** | 3.24 | **sorafenibb** | 14.79 |

[a]Data presented is the mean ± SD value of three independent determinations.
[b]Used as positive control.

Table 4. *In vitro* cytotoxic activities of some target compounds and **SHP244** against the melanoma cells A375.

| Compd. | B1 | B15 | B16 | B17 | B20 | B21 | B22 | B23 | B30 | SHP244[b] |
|---|---|---|---|---|---|---|---|---|---|---|
| $IC_{50}$/μM[a] | 117.97 | >200 | >200 | 151.33 | >200 | 96.35 | 197.6 | 35.89 | 109.24 | >200 |

[a]$IC_{50}$ values shown are the mean of duplicate measurements.
[b]Used as positive control.

Table 5. *In vitro* metabolic stability of liver microsomes for compounds **A22** and **SHP244**.

| | | Liver microsome stability | | |
|---|---|---|---|---|
| compd. | Species | $T_{1/2}$(min) | CL (μL/min/mg) | Remaining (T = 60min) |
| **A22** | HLM | >145 | <9.6 | 82.0% |
| | RLM | >145 | <9.6 | 78.9% |
| **SHP244**[a] | HLM | 14.2 | 97.9 | 4.7% |
| | RLM | 12.2 | 114.0 | 2.9% |

[a]Used as positive control.

#### 3.4.3. *Metabolic stability of liver microsomes for compound A22 in vitro*

As shown in Table 5, compound **A22** showed considerable stability in human and rat liver microsomes, and the clearance rate for both human and rat liver microsomes was less than 9.6 (μL/min/mg) with similar remaining (82.0% and 78.9%, T = 60 min). At the same time, $t_{1/2}$ are both greater than 145 min, which is much greater than the $t_{1/2}$ of SHP244 for human and rat liver microsomes (14.2 and 12.2 min).

## 4. Conclusions

In conclusion, a series of new triazoloquinazolinone and imidazoquinazolinone derivatives were designed and synthesised, and their biological activities were evaluated. Preliminary studies have shown that, compared with SHP244, the most promising compound **B1** has an effective inhibitory effect on SHP2 protein (22.14% inhibition) and melanoma cell line A357 (76.15% inhibition) at 100 μM. In particular, compared with SHP244, the target compound has better stability and has more potential to become a drug. SAR analysis showed that substituting imidazoquinazolinone core for triazoloquinazolinone core also had good SHP2 protein kinase and melanoma cell line A357 inhibitory activity. In summary, triazoloquinazolinone and imidazoquinazolinone SHP2 phosphatase allosteric inhibitors, as novel potential anticancer agents for the treatment of human cancer, are worthy of further study. However, compared with SHP244, the enzymatic inhibitory activities of the two types of target compounds against SHP2 were not significantly enhanced, so the design of "latch" site inhibitors requires more consideration. In future studies, the design of inhibitors will be optimised on the basis of the existing SARs in order to obtain more active SHP2 inhibitors.

## Author contributions

W.Y. Writing—original draft. W.Y., Y.L., Q.R. designed and synthesised the compounds, performed the biological evaluation. T.H., Y.C., D.C., S.W., L.Y., Y.J., C.Z., Z.Z. interpreted the data, reviewed and supervised the manuscript. All authors have read and agreed to the published version of the manuscript.

## Disclosure statement

No potential conflict of interest was reported by the author(s).

## Funding

This work was supported by Guizhou Provincial Natural Science Foundation ([2020]1Y393).
