# Supplementary material for: Design, synthesis and biological evaluation of novel triazoloquinazolinone and imidazoquinazolinone derivatives as allosteric inhibitors of SHP2 phosphatase
Source: J Enzyme Inhib Med Chem. 2022 May 29;37(1):1495–513. doi: 10.1080/14756366.2022.2078968 (PMC9176666; doi:10.1080/14756366.2022.2078968)
Supplement: Supplemental Material [file IENZ_A_2078968_SM5814.pdf]

Supplementary Material

Design, synthesis and biological evaluation of novel triazoloquinazolinone and imidazoquinazolinone derivatives as allosteric inhibitors of SHP2 phosphatase

Wenjun Ye[a,b], Ye Liu[a,b], Qian Ren[a,b], Tianhui Liao[a,b], Yumei Chen[a,b], Dongmei Chen[a,b], Sisi Wang[a,b], Lihong Yao[a,b], Yihe Jia[c], Chunshen Zhao[a,b], Zhixu Zhou[a,b]

a. School of Pharmaceutical Sciences, Guizhou University, Guiyang, 550025, China
b. Guizhou Engineering Laboratory for Synthetic Drugs, Guiyang, 550025, China
c. Department of Medicinal Chemistry, China Pharmaceutical University, Nanjing, 211198, China

Contents:

Table S1. Summary of target compounds.

Figure S1. ^{1}H-NMR spectrum of **6c.**

Figure S2. MS spectrum of **6c.**

Figure S3. ^{1}H-NMR spectrum of **6e.**

Figure S4. ^{1}H-NMR spectrum of **7c.**

Figure S5. MS spectrum of **7c.**

Figure S6. ^{1}H-NMR spectrum of **7e.**

Figure S7. ^{1}H-NMR spectrum of **8c.**

Figure S8. MS spectrum of **8c.**

Figure S9. ^{1}H-NMR spectrum of **8e.**

Figure S10. ^{1}H-NMR spectrum of **10.**

Figure S11. ^{1}H-NMR spectrum of **11.**

Figure S12. ^{1}H-NMR spectrum of **12.**

Figure S13. ^{1}H-NMR spectrum of **13.**

Figure S14. ^{1}H-NMR spectrum of **A1.**

Figure S15. MS spectrum of **A1.**

Figure S16. ^{1}H-NMR spectrum of **A2.**

Figure S17. MS spectrum of **A2.**

Figure S18. ^{1}H-NMR spectrum of **A3.**

Figure S19. MS spectrum of **A3.**

Figure S20. ^{1}H-NMR spectrum of **A4.**

Figure S21. MS spectrum of **A4.**

Figure S22. ^{1}H-NMR spectrum of **A5.**

Figure S23. MS spectrum of **A5.**

Figure S24. ^{1}H-NMR spectrum of **A6.**

Figure S25. MS spectrum of **A6.**

Figure S26. ^{1}H-NMR spectrum of **A7.**

Figure S27. MS spectrum of **A7.**

Figure S28. ^{1}H-NMR spectrum of **A8.**

Figure S29. MS spectrum of **A8.**

Figure S30. ^{1}H-NMR spectrum of **A9.**

Figure S31. MS spectrum of **A9.**

Figure S32. ^{1}H-NMR spectrum of **A10.**

Figure S33. MS spectrum of **A10.**

Figure S34. ^{1}H-NMR spectrum of **A11.**

Figure S35. MS spectrum of **A11.**

Figure S36. ^{1}H-NMR spectrum of **A12.**

Figure S37. MS spectrum of **A12.**

Figure S38. ^{1}H-NMR spectrum of **A13.**

Figure S39. MS spectrum of **A13.**

Figure S40. ^{1}H-NMR spectrum of **A14.**

Figure S41. MS spectrum of **A14.**

Figure S42. ^{1}H-NMR spectrum of **A15.**

Figure S43. MS spectrum of **A15.**

Figure S44. ^{1}H-NMR spectrum of **A16.**

Figure S45. MS spectrum of **A16.**

Figure S46. ^{1}H-NMR spectrum of **A17.**

Figure S47. MS spectrum of **A17.**

Figure S48. ^{1}H-NMR spectrum of **A18.**

Figure S49. MS spectrum of **A18.**

Figure S50. ^{1}H-NMR spectrum of **A19.**

Figure S51. MS spectrum of **A19.**

Figure S52. ^{1}H-NMR spectrum of **A20.**

Figure S53. MS spectrum of **A20.**

Figure S54. ^{1}H-NMR spectrum of A**21.**

Figure S55. MS spectrum of A**21.**

Figure S56. ^{1}H-NMR spectrum of **A22.**

Figure S57. MS spectrum of **A22.**

Figure S58. ^{1}H-NMR spectrum of **A23.**

Figure S59. MS spectrum of **A23.**

Figure S60. ^{1}H-NMR spectrum of **A24.**

Figure S61. MS spectrum of **A24.**

Figure S62. ^{1}H-NMR spectrum of **A25.**

Figure S63. MS spectrum of **A25.**

Figure S64. ^{1}H-NMR spectrum of **A26.**

Figure S65. MS spectrum of **A26.**

Figure S66. ^{1}H-NMR spectrum of **A27.**

Figure S67. MS spectrum of **A27.**

Figure S68. ^{1}H-NMR spectrum of **A28.**

Figure S69. MS spectrum of **A28.**

Figure S70. ^{1}H-NMR spectrum of **A29.**

Figure S71. MS spectrum of **A29.**

Figure S72. ^{1}H-NMR spectrum of **A30.**

Figure S73. MS spectrum of **A30.**

Figure S74. ^{1}H-NMR spectrum of **A31.**

Figure S75. MS spectrum of **A31.**

Figure S76. ^{1}H-NMR spectrum of **A32.**

Figure S77. MS spectrum of **A32.**

Figure S78. ^{1}H-NMR spectrum of **A33.**

Figure S79. MS spectrum of **A33.**

Figure S80. ^{1}H-NMR spectrum of **A34.**

Figure S81. MS spectrum of **A34.**

Figure S82. ^{1}H-NMR spectrum of **A35.**

Figure S83. MS spectrum of **A35.**

Figure S84. ^{1}H-NMR spectrum of **B1.**

Figure S85. MS spectrum of **B1.**

Figure S86. ^{1}H-NMR spectrum of **B2.**

Figure S87. MS spectrum of **B2.**

Figure S88. ^{1}H-NMR spectrum of **B3.**

Figure S89. MS spectrum of **B3.**

Figure S90. ^{1}H-NMR spectrum of **B4.**

Figure S91. MS spectrum of **B4.**

Figure S92. ^{1}H-NMR spectrum of **B5.**

Figure S93. MS spectrum of **B5.**

Figure S94. ^{1}H-NMR spectrum of **B6.**

Figure S95. MS spectrum of **B6.**

Figure S96. ^{1}H-NMR spectrum of **B7.**

Figure S97. MS spectrum of **B7.**

Figure S98. ^{1}H-NMR spectrum of **B8.**

Figure S99. MS spectrum of **B8.**

Figure S100. ^{1}H-NMR spectrum of **B9.**

Figure S101. MS spectrum of **B9.**

Figure S102. ^{1}H-NMR spectrum of **B10.**

Figure S103. MS spectrum of **B10.**

Figure S104. ^{1}H-NMR spectrum of **B11.**

Figure S105. MS spectrum of **B11.**

Figure S106. ^{1}H-NMR spectrum of **B12.**

Figure S107. MS spectrum of **B12.**

Figure S108. ^{1}H-NMR spectrum of **B13.**

Figure S109. MS spectrum of **B13.**

Figure S110. ^{1}H-NMR spectrum of **B14.**

Figure S111. MS spectrum of **B14.**

Figure S112. ^{1}H-NMR spectrum of **B15.**

Figure S113. MS spectrum of **B15.**

Figure S114. ^{1}H-NMR spectrum of **B16.**

Figure S115. MS spectrum of **B16.**

Figure S116. ^{1}H-NMR spectrum of **B17.**

Figure S117. MS spectrum of **B17.**

Figure S118. ^{1}H-NMR spectrum of **B18.**

Figure S119. MS spectrum of **B18.**

Figure S120. ^{1}H-NMR spectrum of **B19.**

Figure S121. MS spectrum of **B19.**

Figure S122. ^{1}H-NMR spectrum of **B20.**

Figure S123. MS spectrum of **B20.**

Figure S124. ^{1}H-NMR spectrum of B**21.**

Figure S125. MS spectrum of B**21.**

Figure S126. ^{1}H-NMR spectrum of **B22.**

Figure S127. MS spectrum of **B22.**

Figure S128. ^{1}H-NMR spectrum of **B23.**

Figure S129. MS spectrum of **B23.**

Figure S130. ^{1}H-NMR spectrum of **B24.**

Figure S131. MS spectrum of **B24.**

Figure S132. ^{1}H-NMR spectrum of **B25.**

Figure S133. MS spectrum of **B25.**

Figure S134. ^{1}H-NMR spectrum of **B26.**

Figure S135. MS spectrum of **B26.**

Figure S136. ^{1}H-NMR spectrum of **B27**.

Figure S137. MS spectrum of **B27**.

Figure S138. ^{1}H-NMR spectrum of **B28**.

Figure S139. MS spectrum of **B28**.

Figure S140. ^{1}H-NMR spectrum of **B29.**

Figure S141. MS spectrum of **B29.**

Figure S142. ^{1}H-NMR spectrum of **B30.**

Figure S143. MS spectrum of **B30.**

Figure S144. ^{1}H-NMR spectrum of **B31.**

Figure S145. MS spectrum of **B31.**

Figure S146. ^{1}H-NMR spectrum of **B32**.

Figure S147. MS spectrum of **B32**.

Corresponding author. Tel./fax: +86-13639093442

E-mail Address: zhixuzhou@126.com

Table S1. Summary of target compounds

Compd	Structural formula	Compd	Structural formula
**A1**	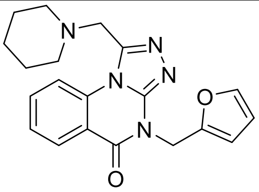	**A35**	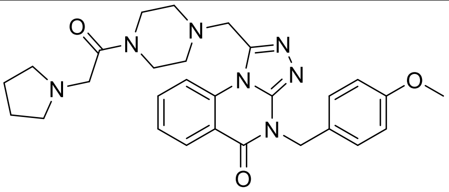
**A2**	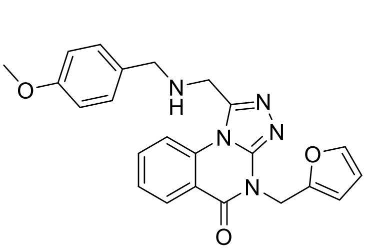	**B1**	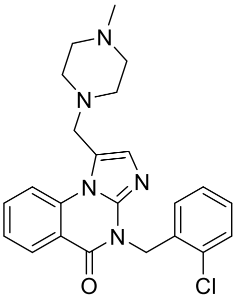
**A3**	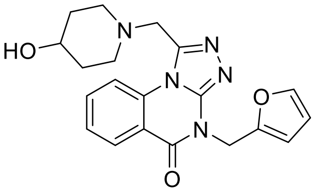	**B2**	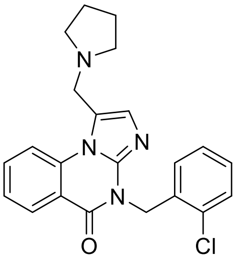
**A4**	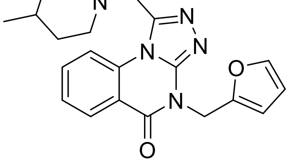	**B3**	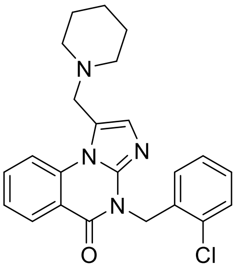
**A5**	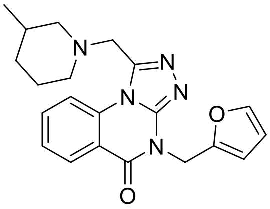	**B4**	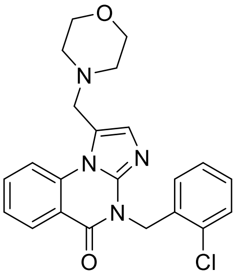
**A6**	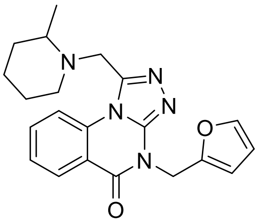	**B5**	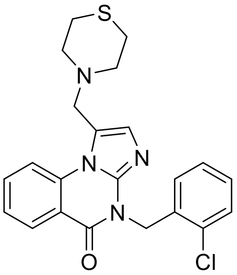

**A7**	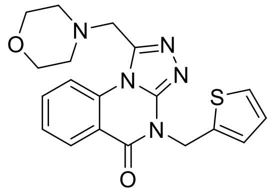	**B6**	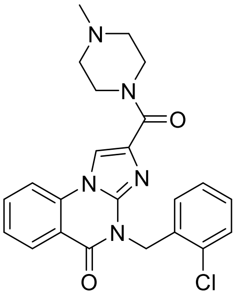
**A8**	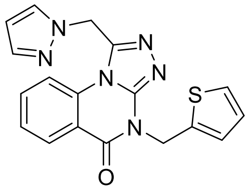	**B7**	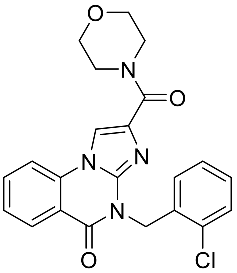
**A9**	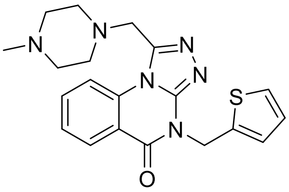	**B8**	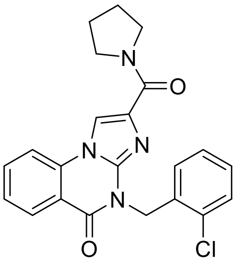
**A10**	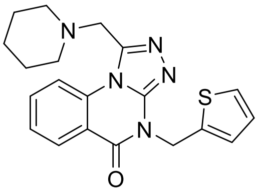	**B9**	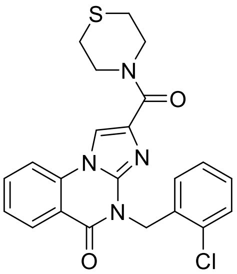
**A11**	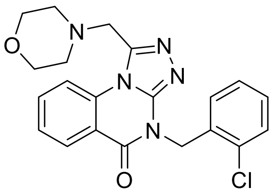	**B10**	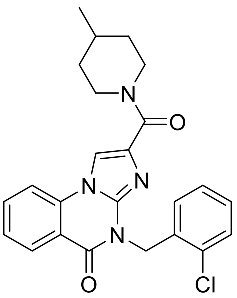
**A12**	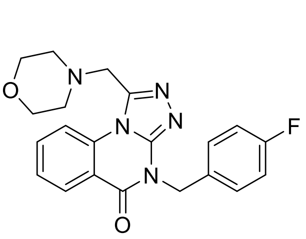	**B11**	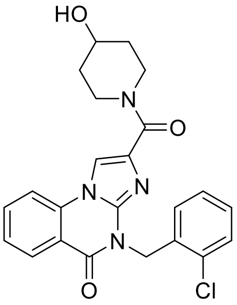

**A13**	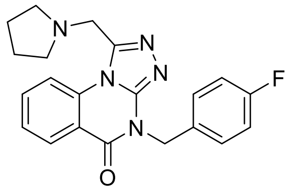	**B12**	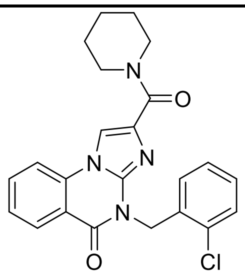
**A14**	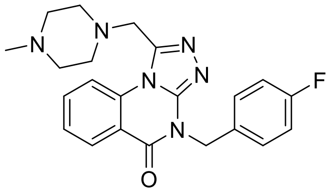	**B13**	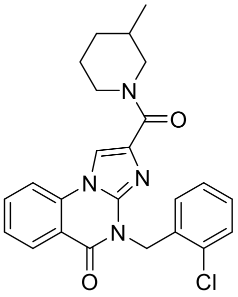
**A15**	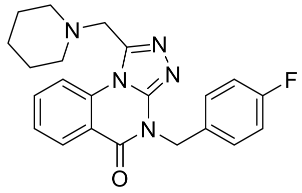	**B14**	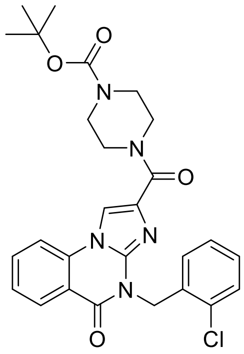
**A16**	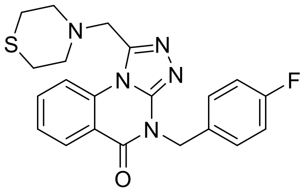	**B15**	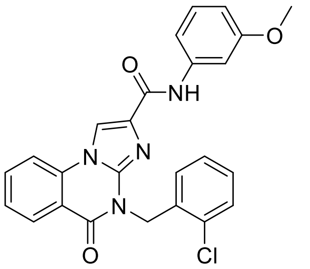
**A17**	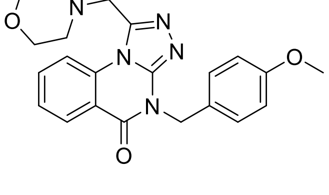	**B16**	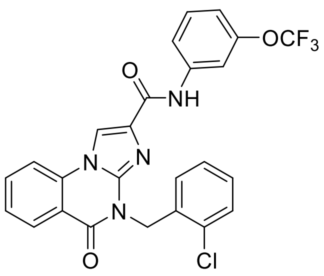
**A18**	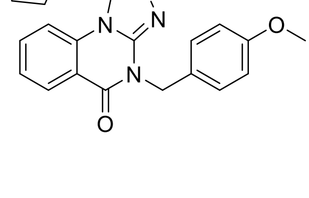	**B17**	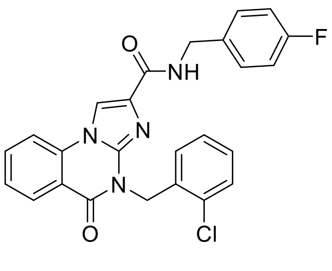

A19 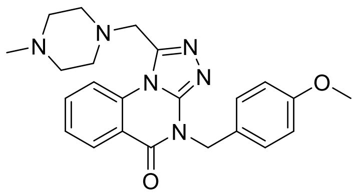

B18 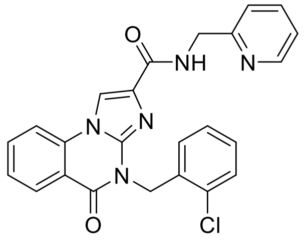

A20 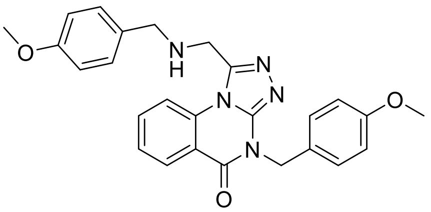

B19 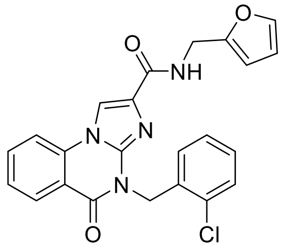

A21 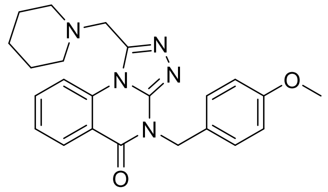

B20 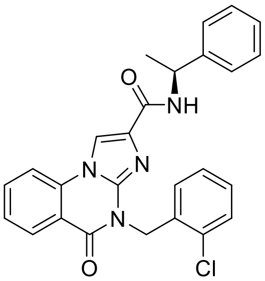

A22 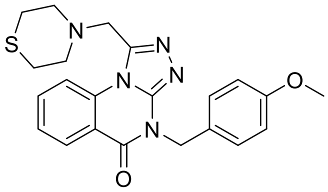

B21 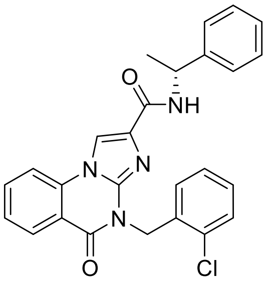

A23 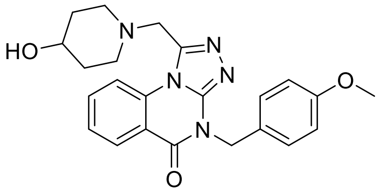

B22 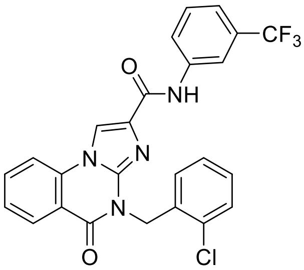

A24 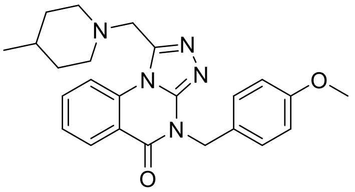

B23 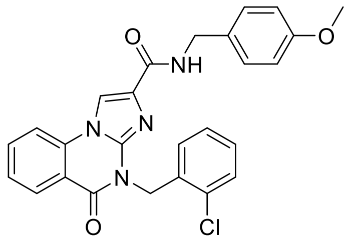

A25 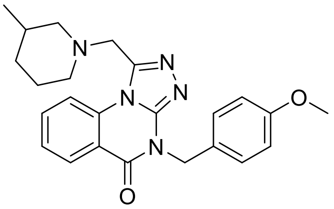

B24 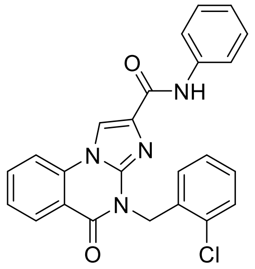

A26

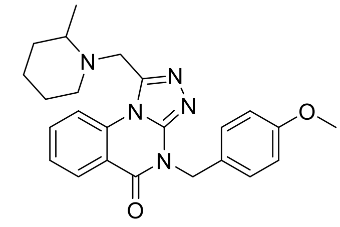

B25

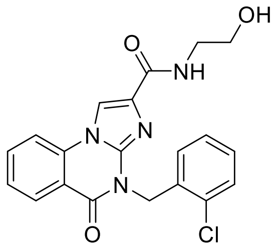

A27

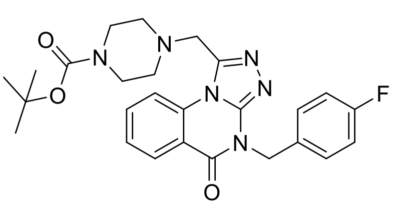

B26

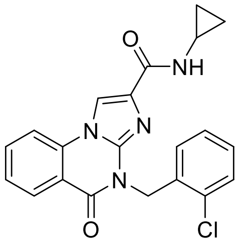

A28

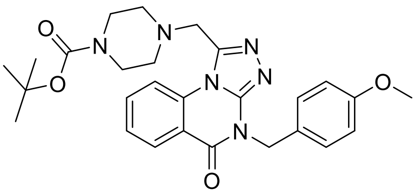

B27

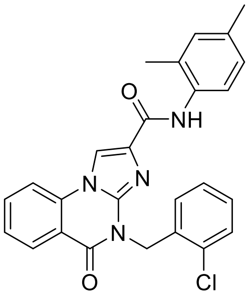

A29

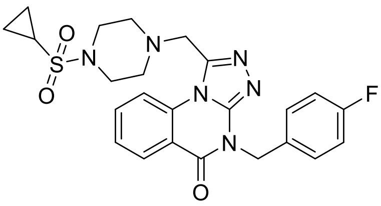

B28

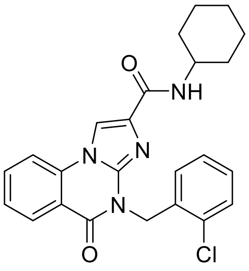

A30

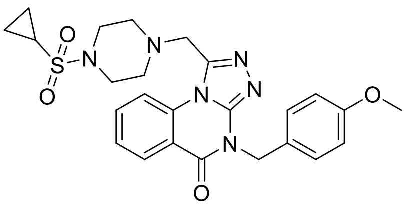

B29

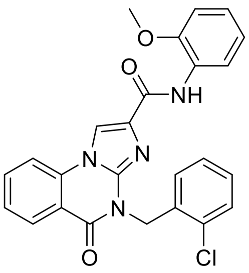

A31

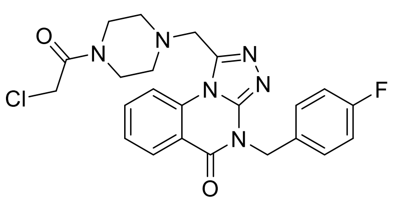

B30

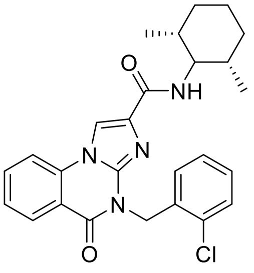

A32

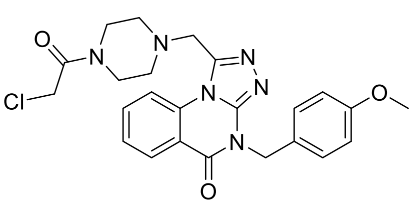

B31

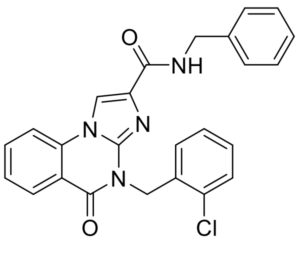

A33

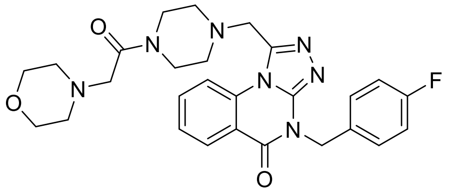

B32

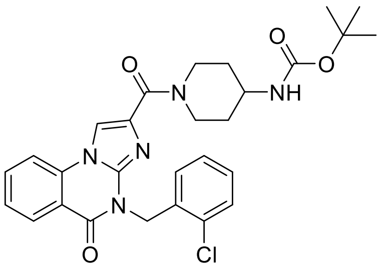

A34

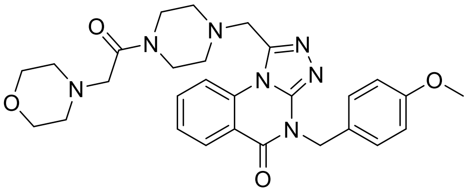

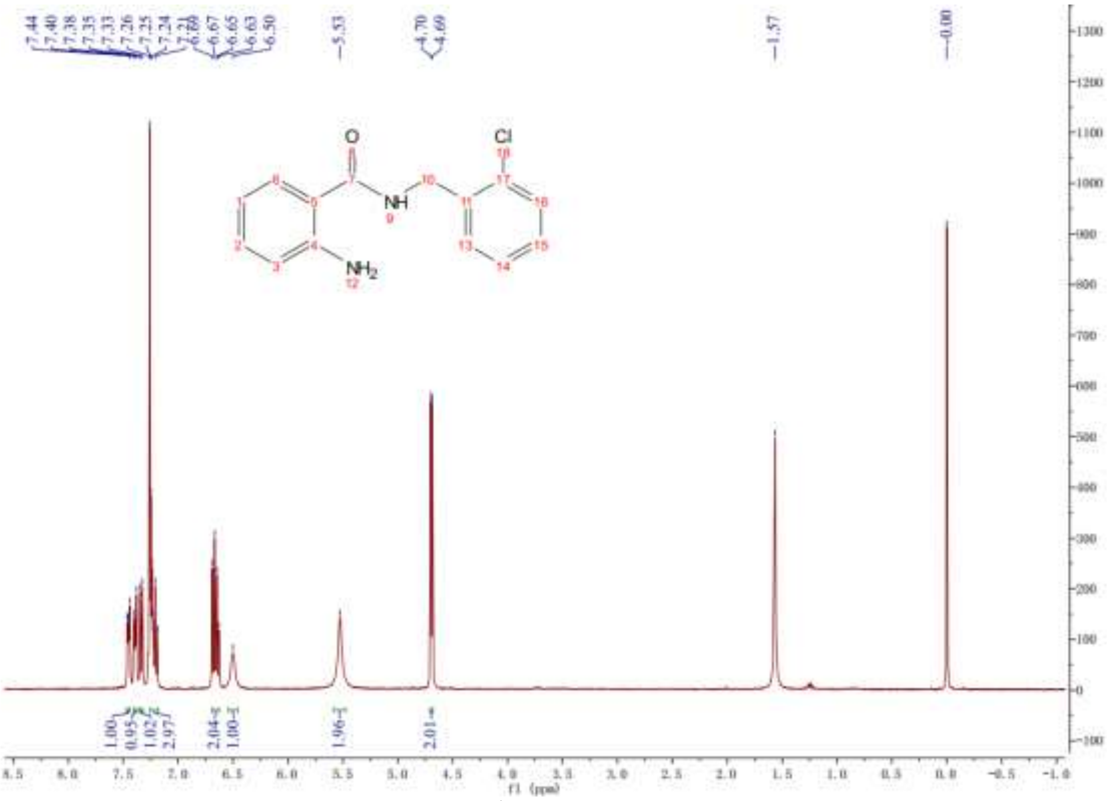

Figure S1. ^{1}H-NMR spectrum of **6c.**

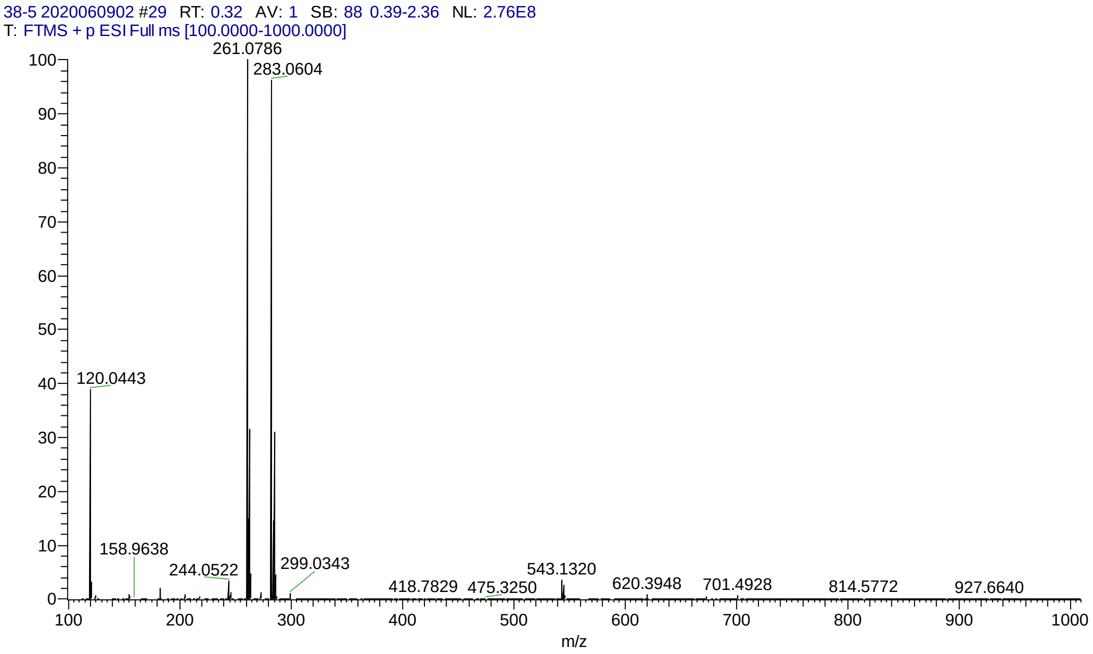

Figure S2. MS spectrum of **6c**

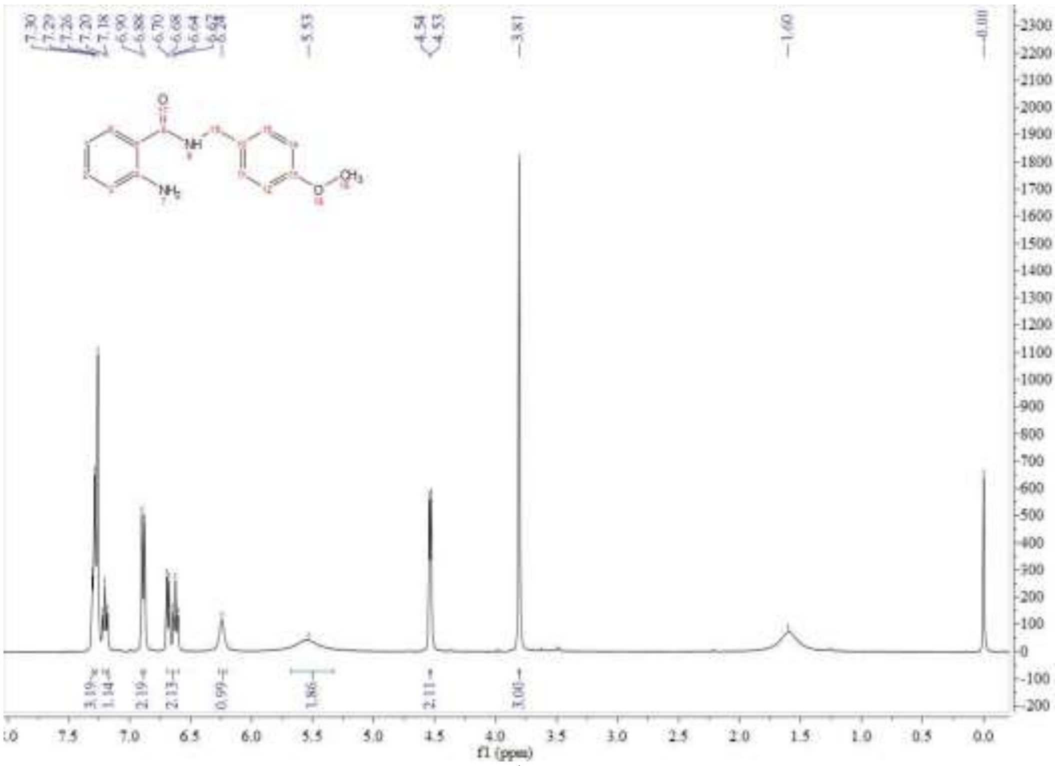

Figure S3. ^{1}H-NMR spectrum of **6e.**

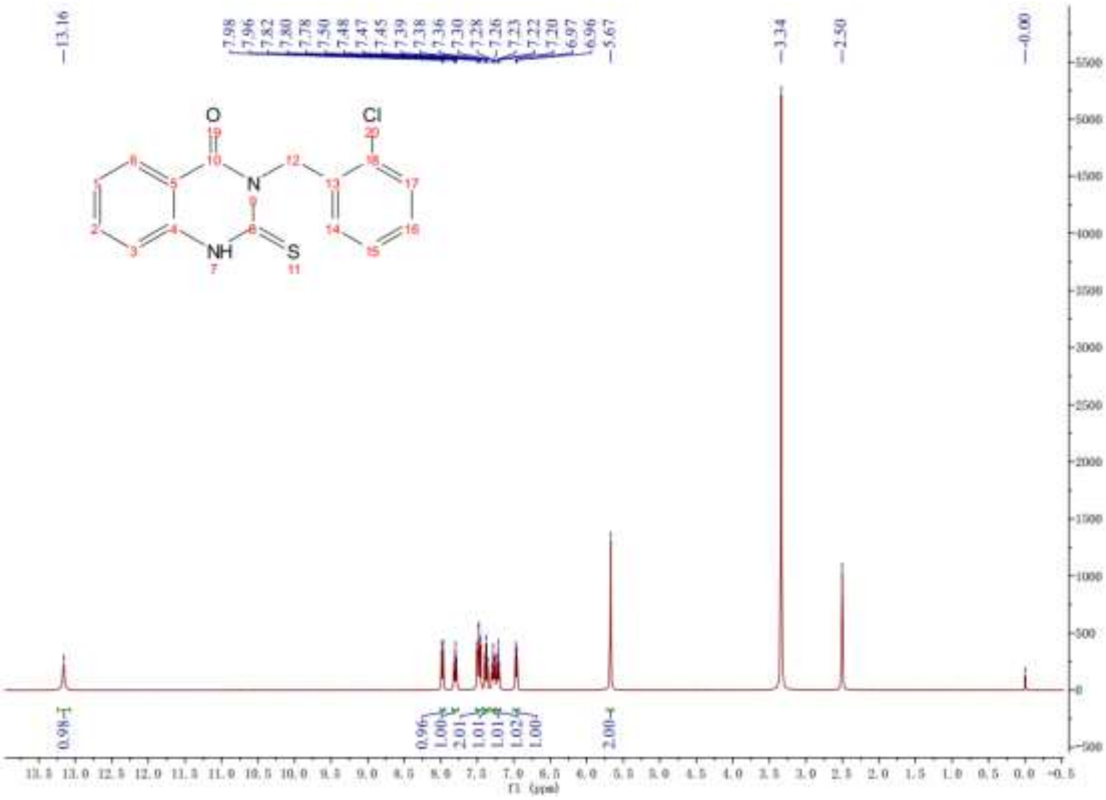

Figure S4. ^{1}H-NMR spectrum of **7c.**

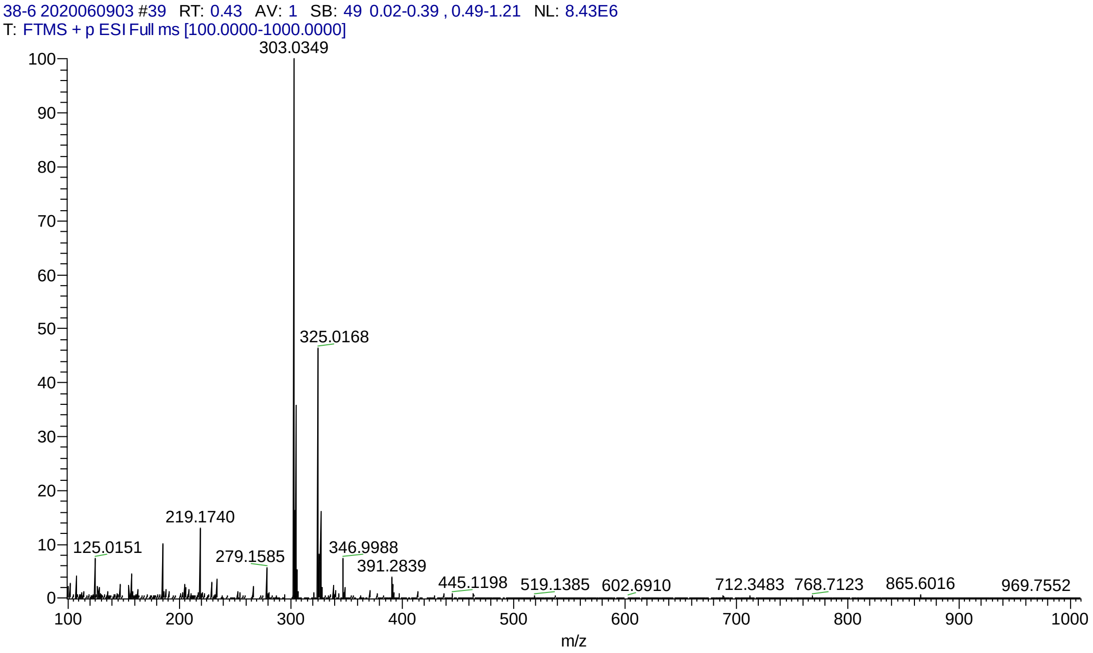

Figure S5. MS spectrum of **7c.**

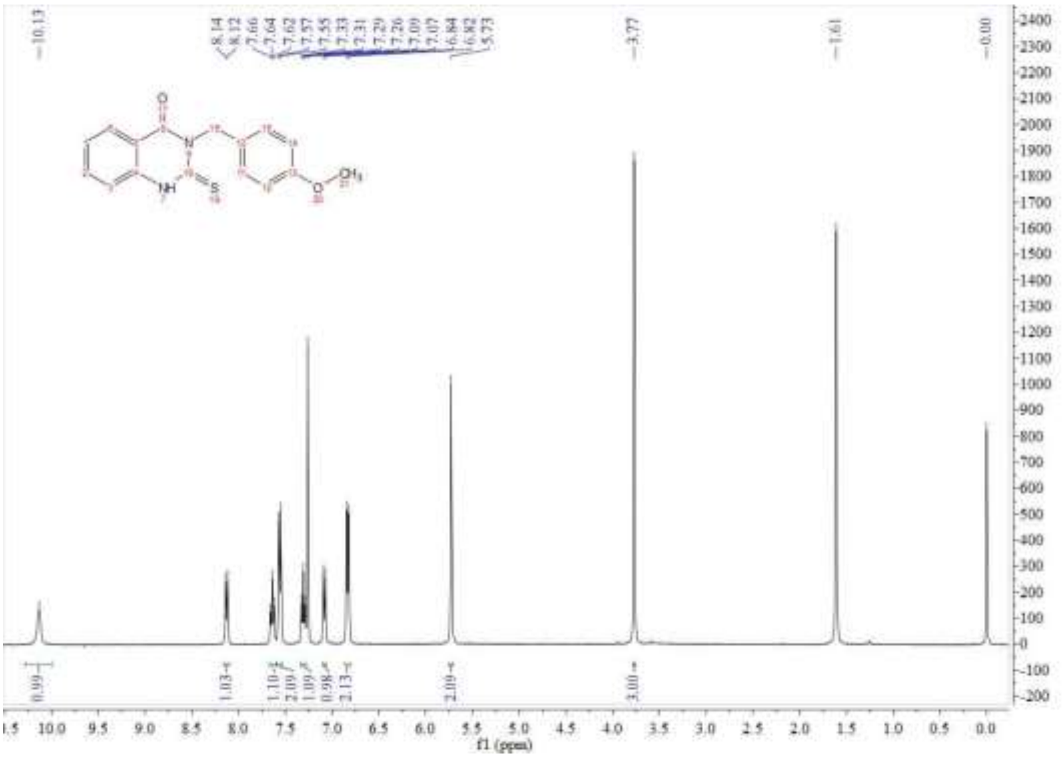

Figure S6. ^{1}H-NMR spectrum of **7e.**

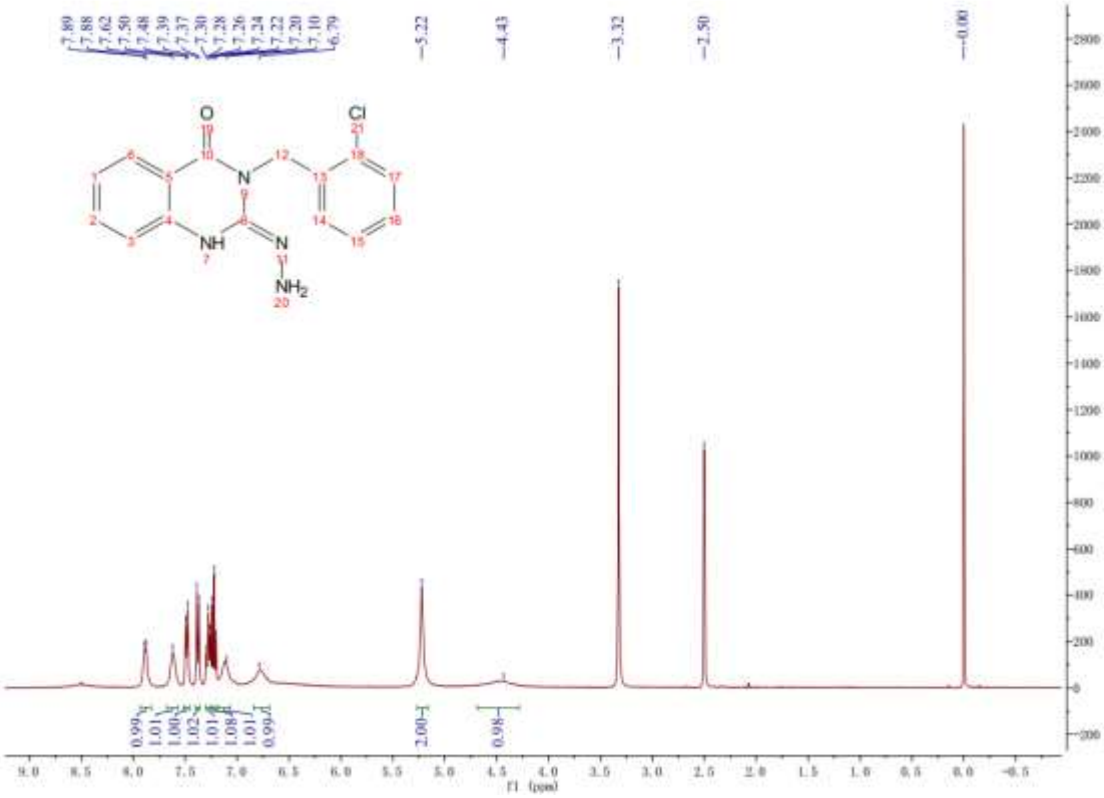

Figure S7. ^{1}H-NMR spectrum of **8c.**

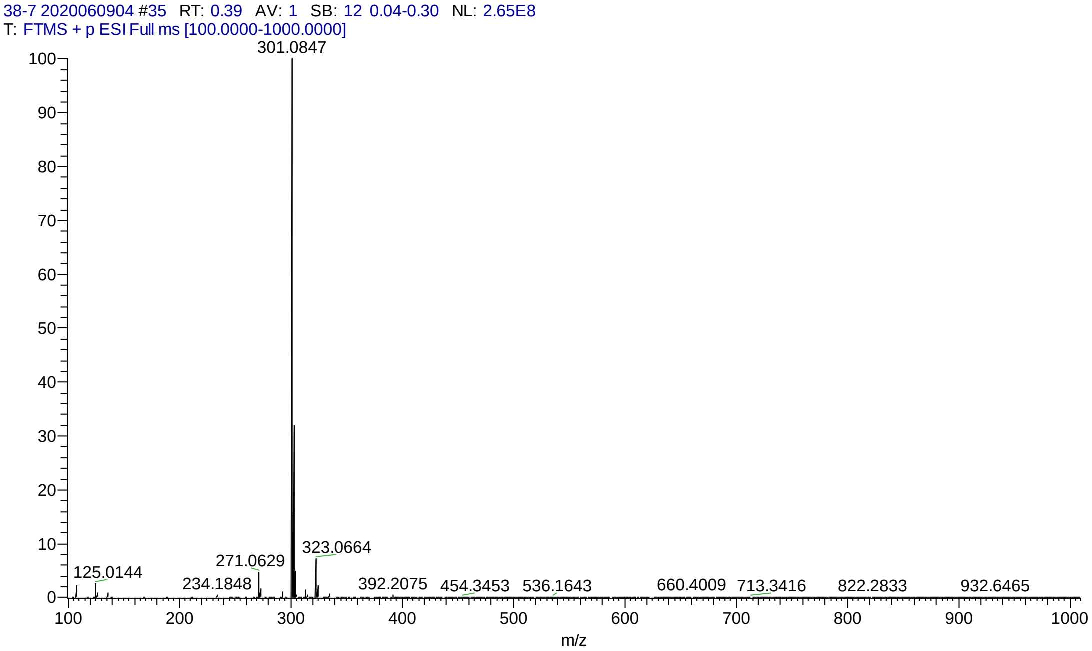

Figure S8. MS spectrum of **8c.**

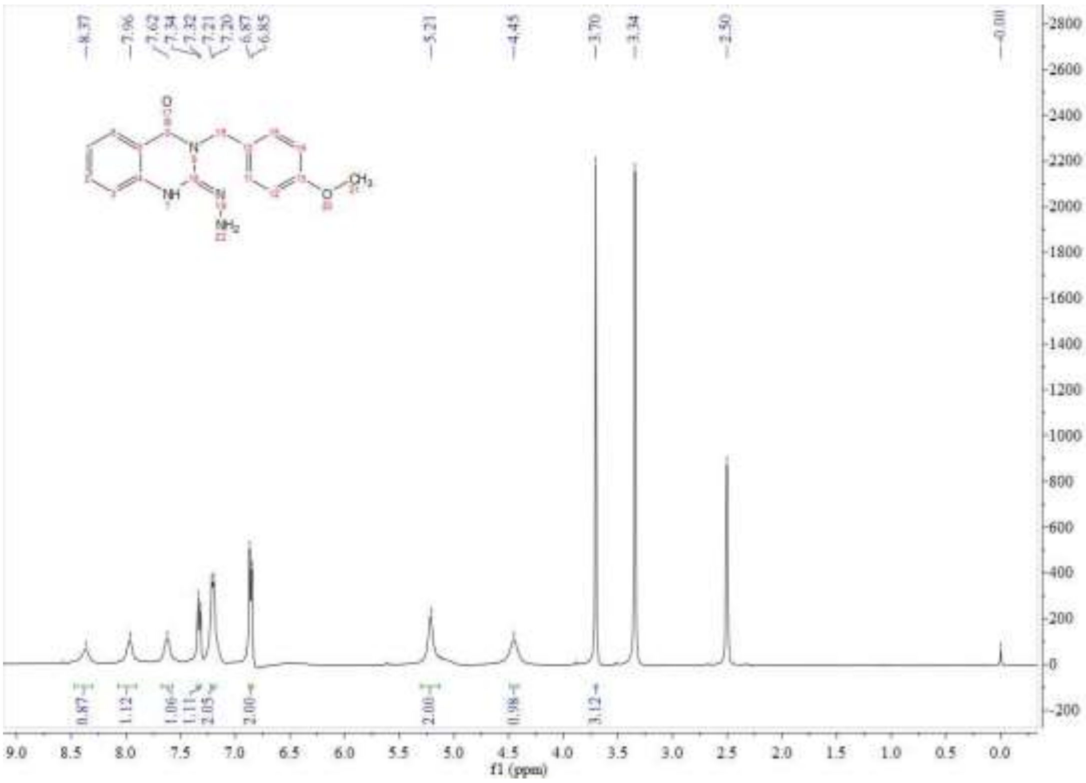

Figure S9. ^{1}H-NMR spectrum of **8e.**

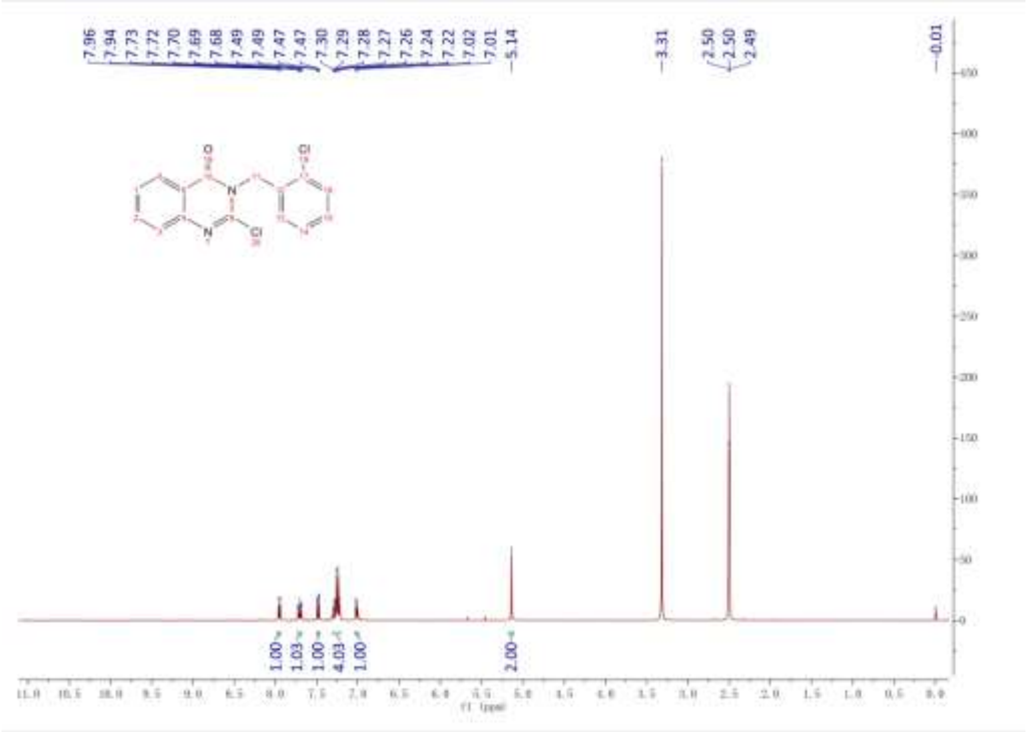

Figure S10. ^{1}H-NMR spectrum of **10.**

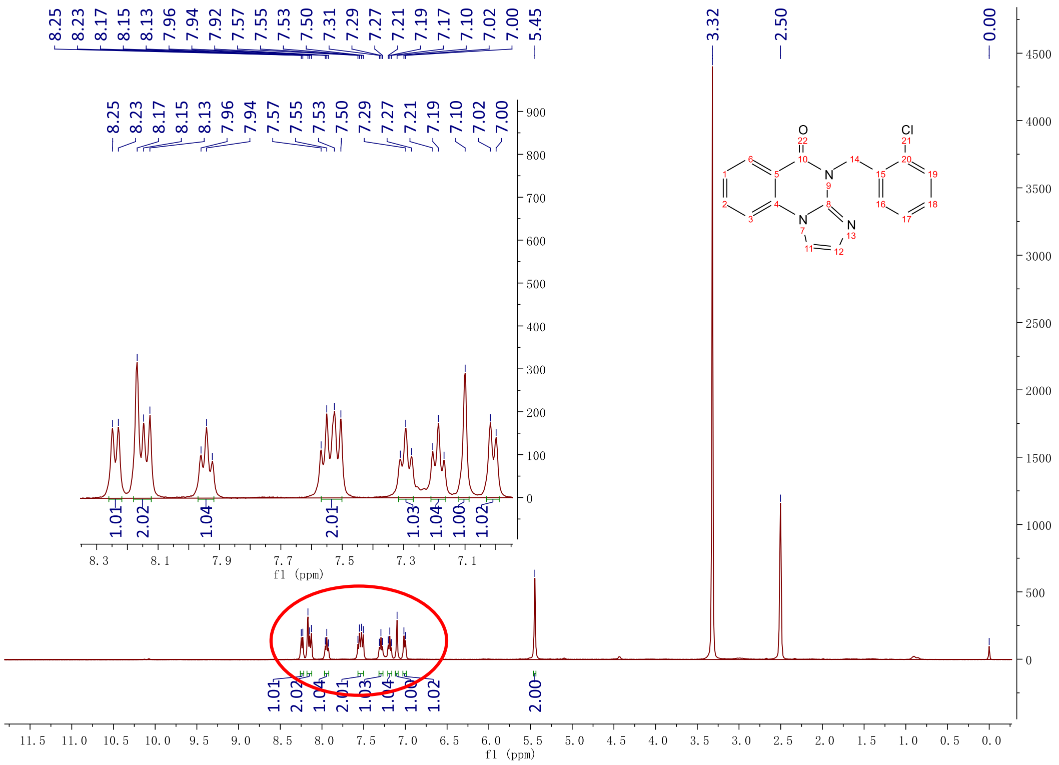

Figure S11. ^{1}H-NMR spectrum of **11.**

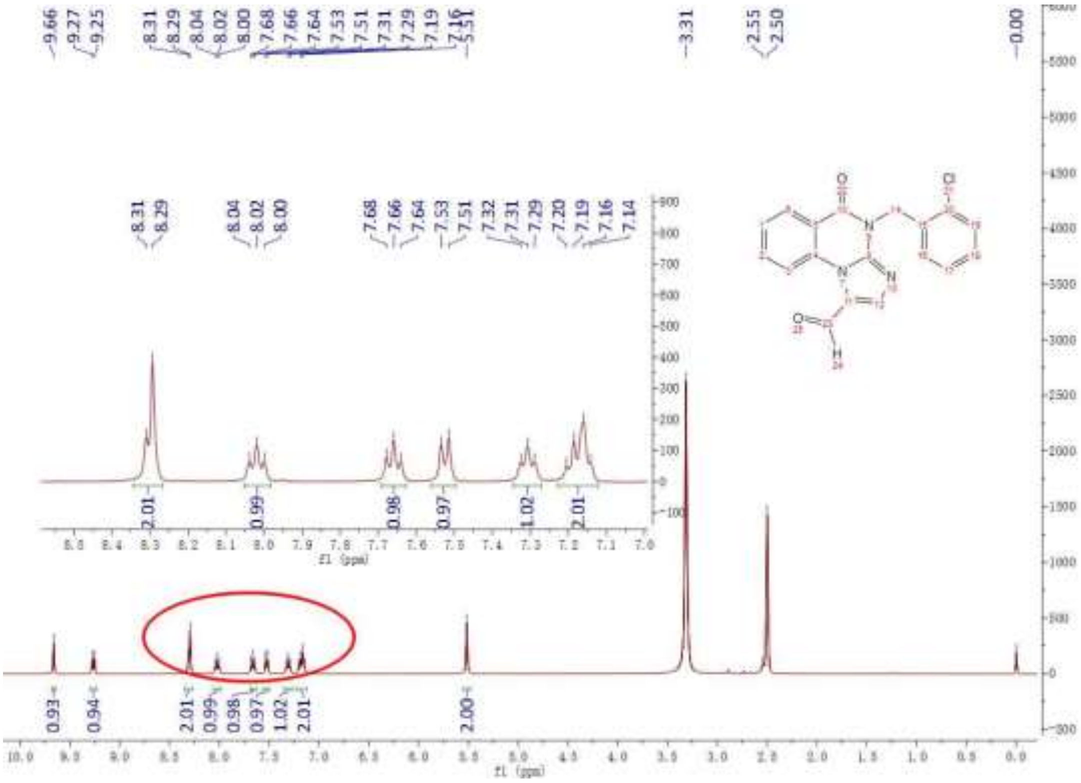

Figure S12. ^{1}H-NMR spectrum of **12.**

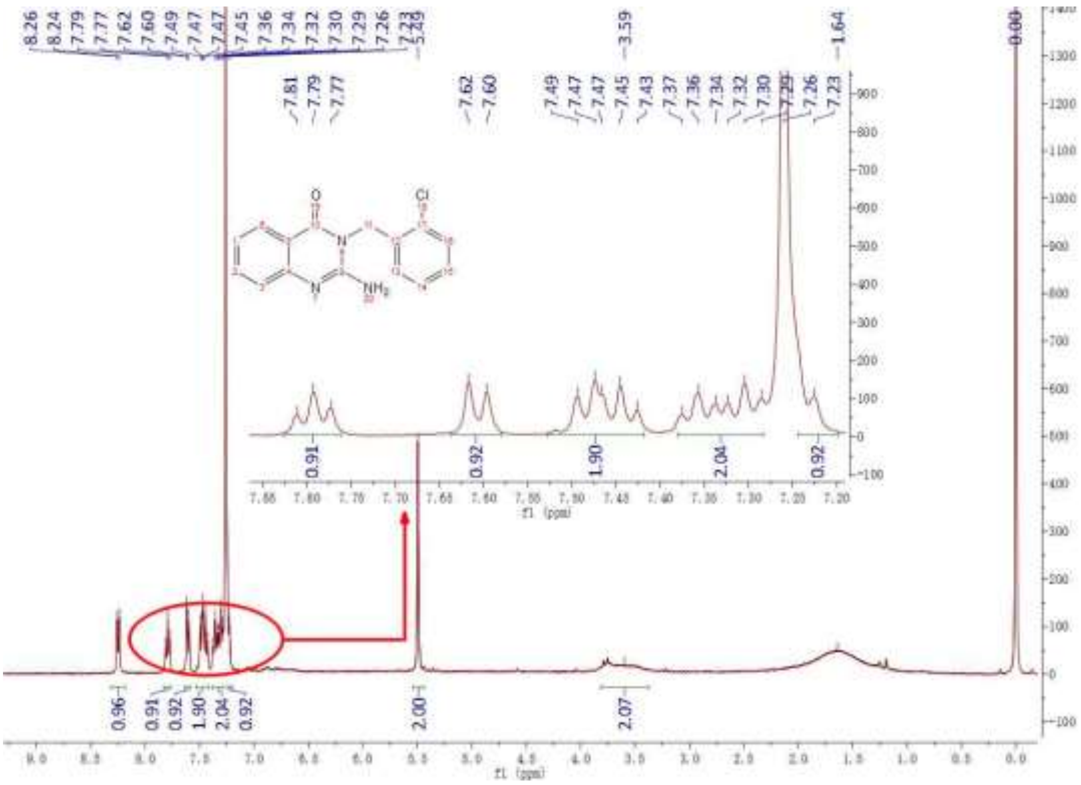

Figure S13. ^{1}H-NMR spectrum of **13.**

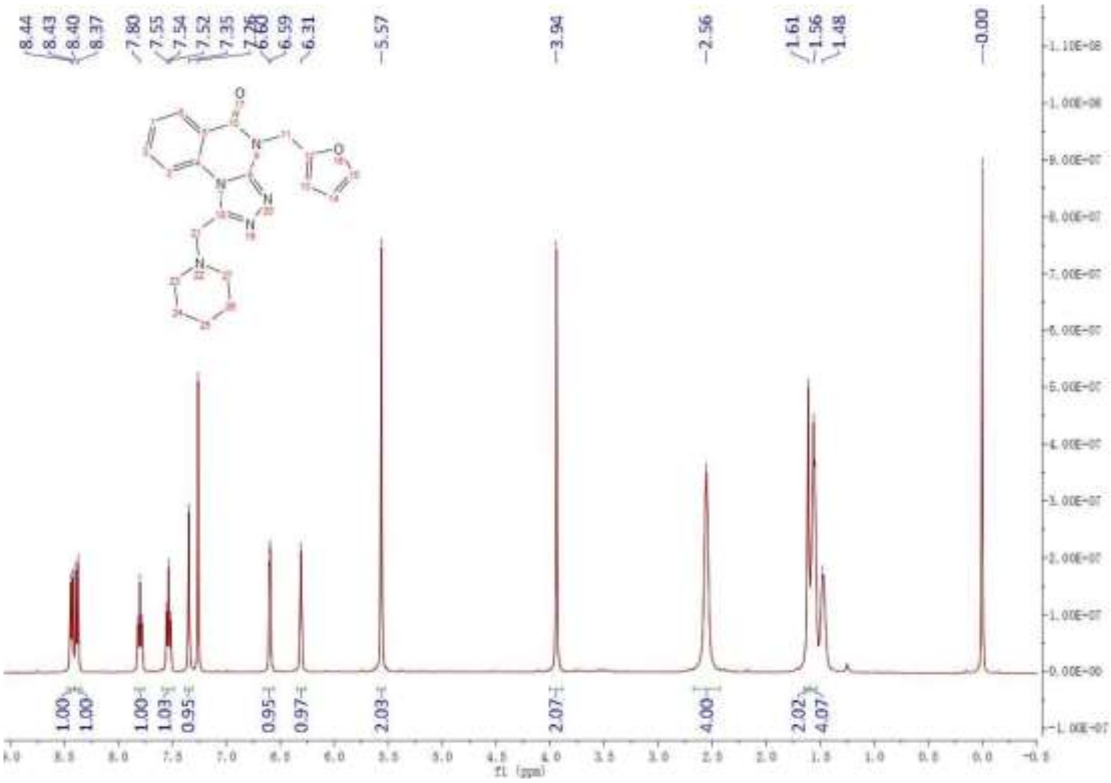

Figure S14. ^{1}H-NMR spectrum of **A1.**

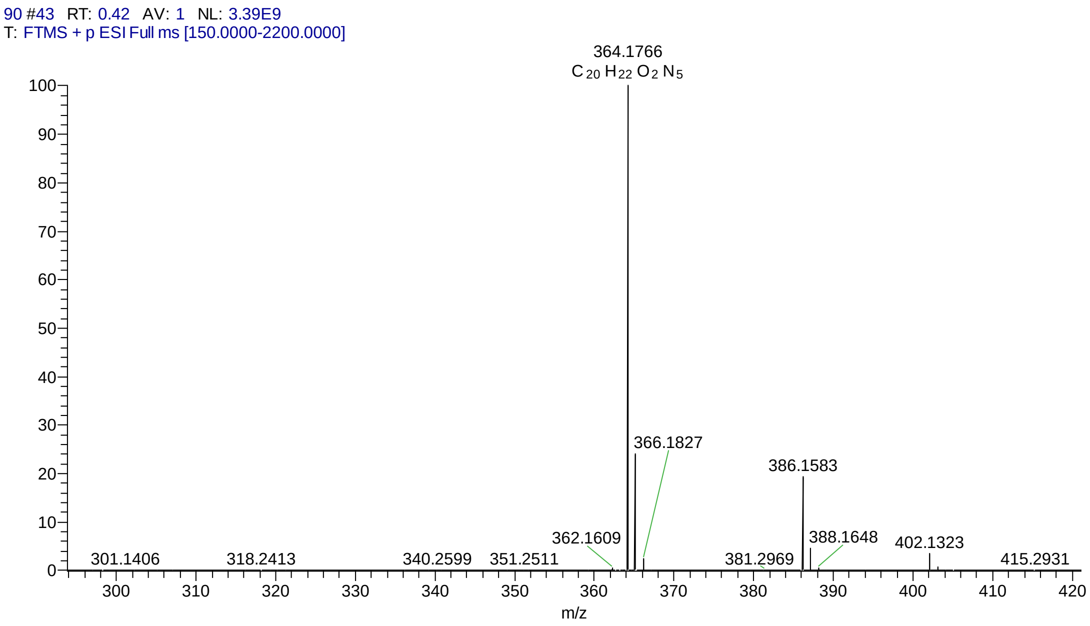

Figure S15. MS spectrum of **A1.**

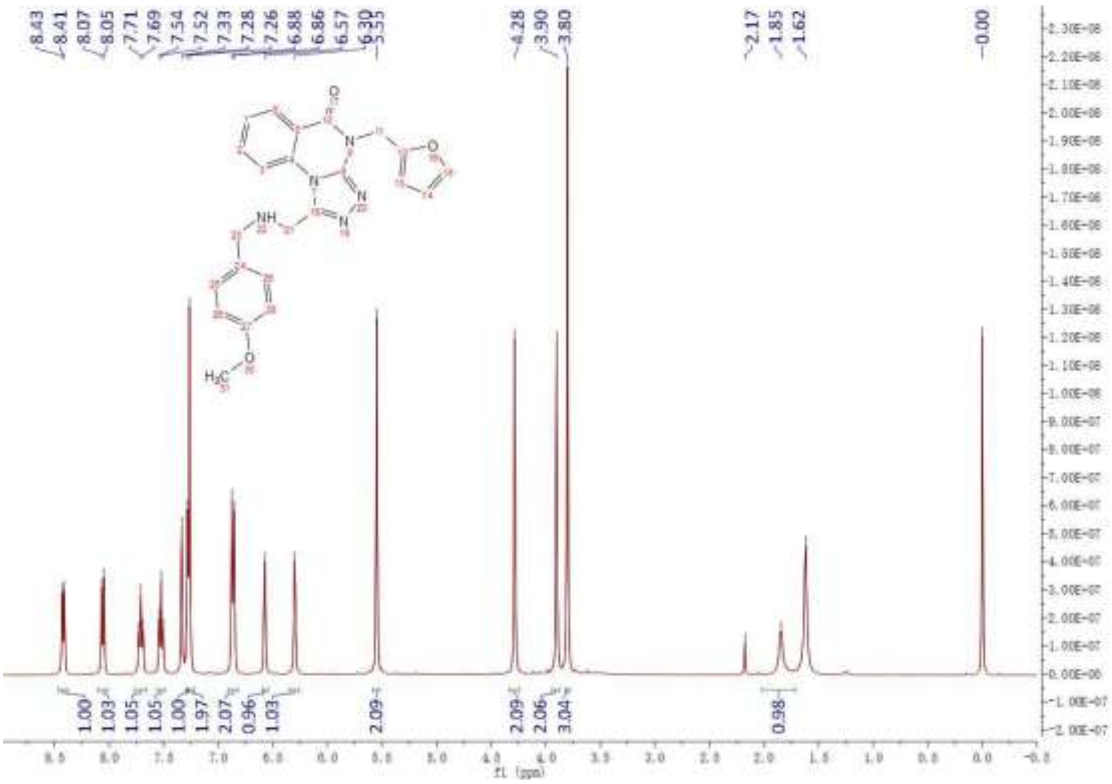

Figure S16. ^{1}H-NMR spectrum of **A2.**

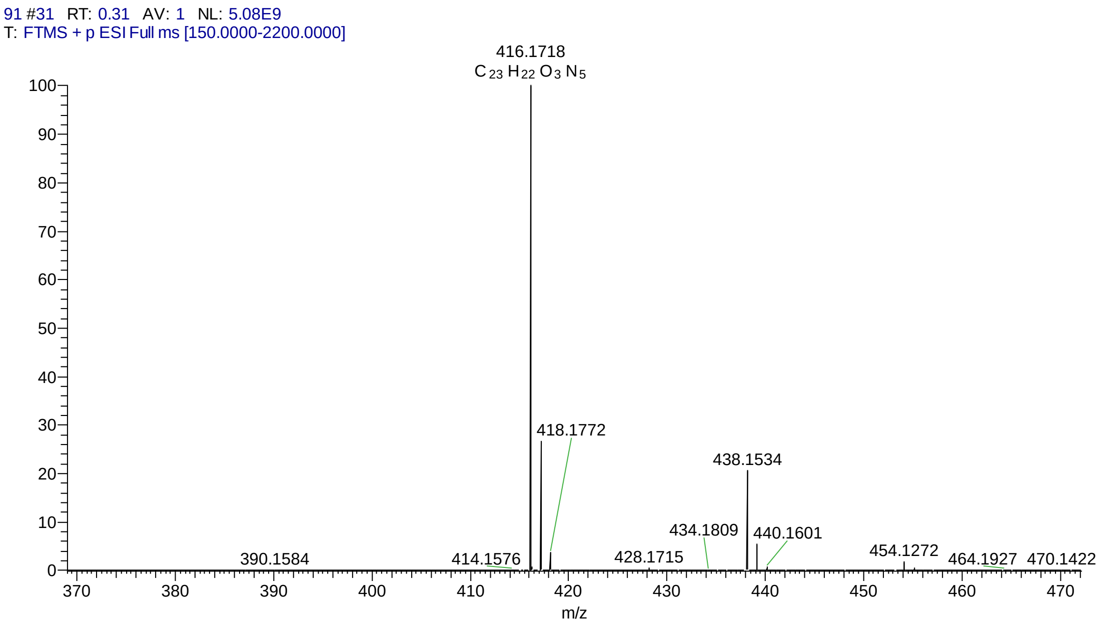

Figure S17. MS spectrum of **A2.**

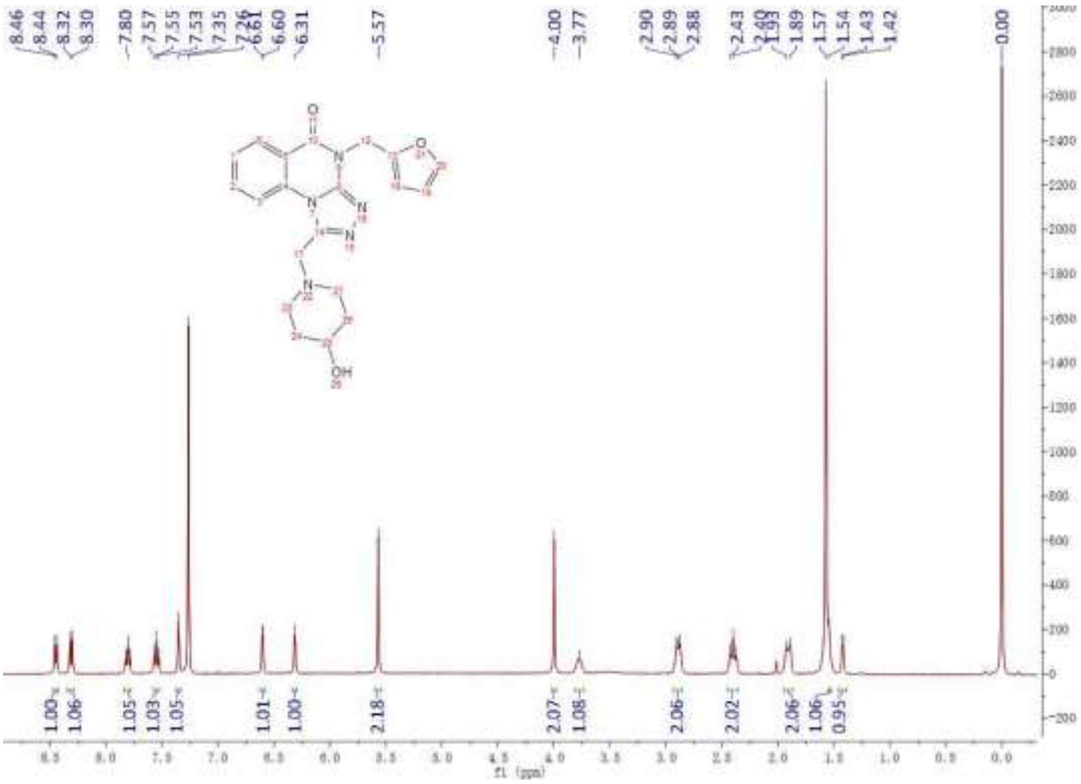

Figure S18. ^{1}H-NMR spectrum of **A3.**

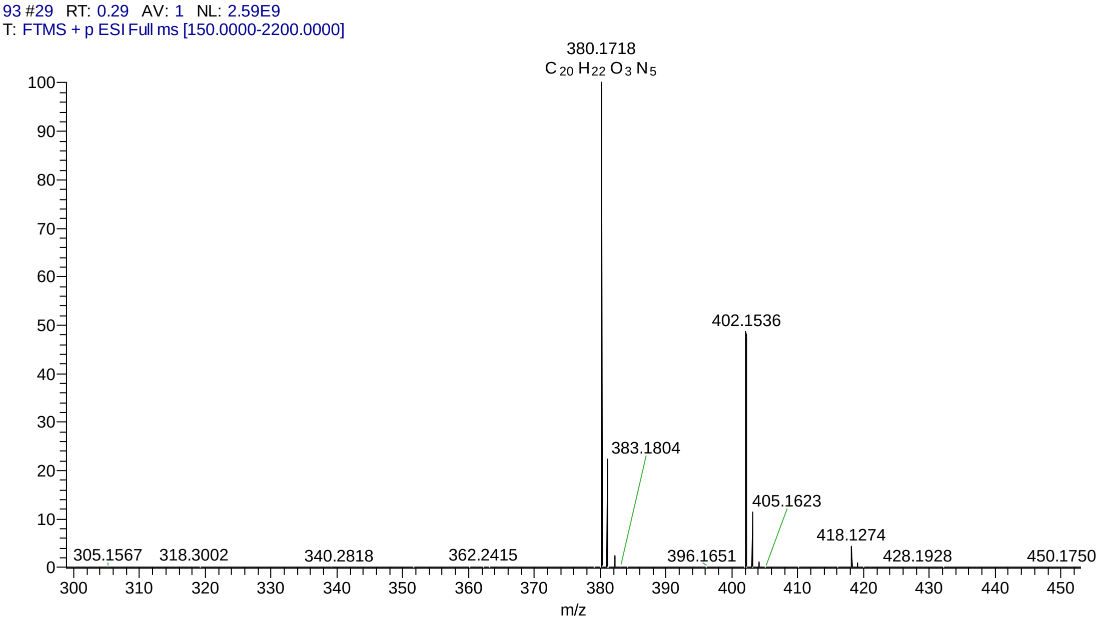

Figure S19. MS spectrum of **A3.**

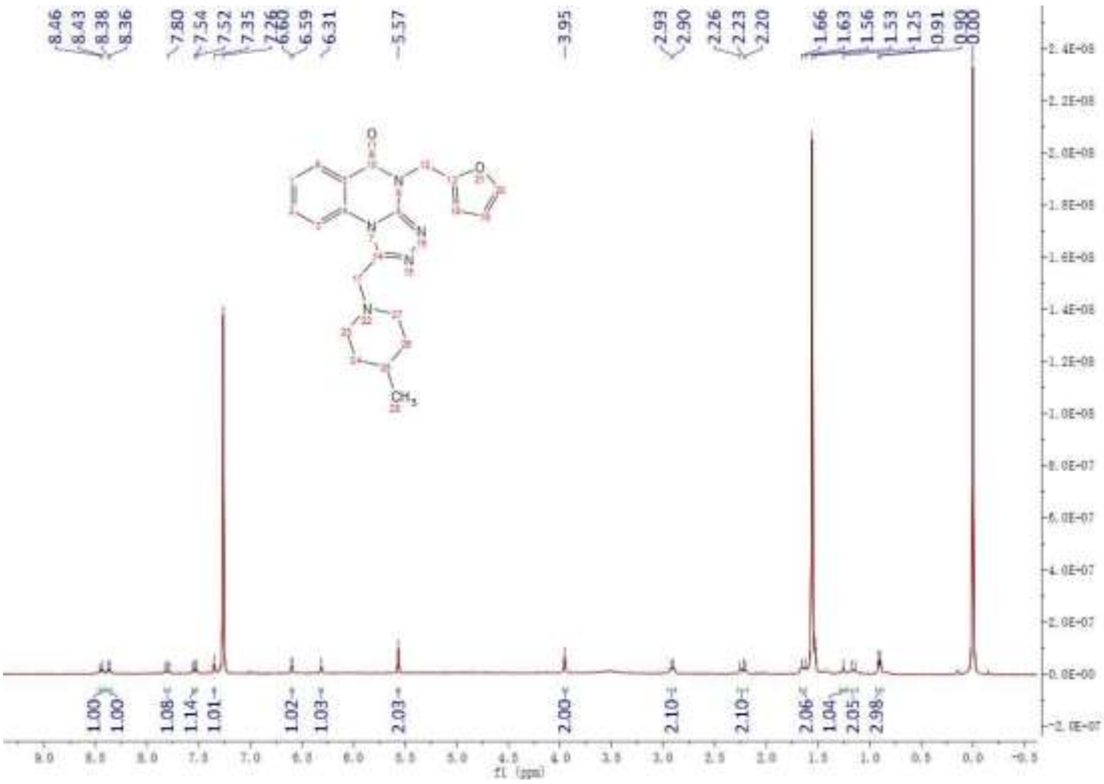

Figure S20. ^{1}H-NMR spectrum of **A4.**

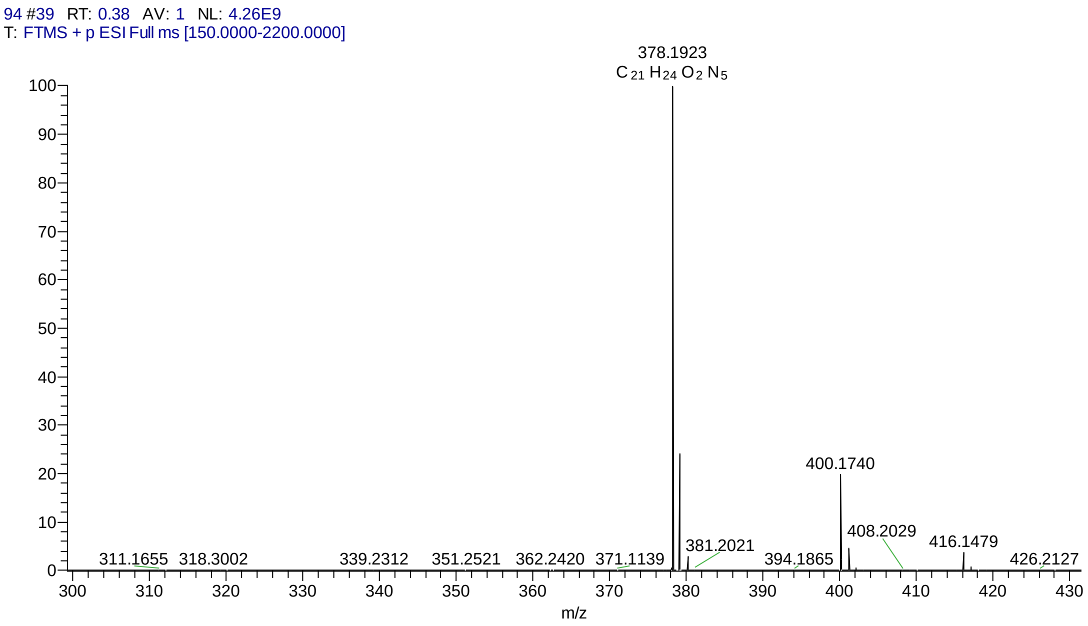

Figure S21. MS spectrum of **A4.**

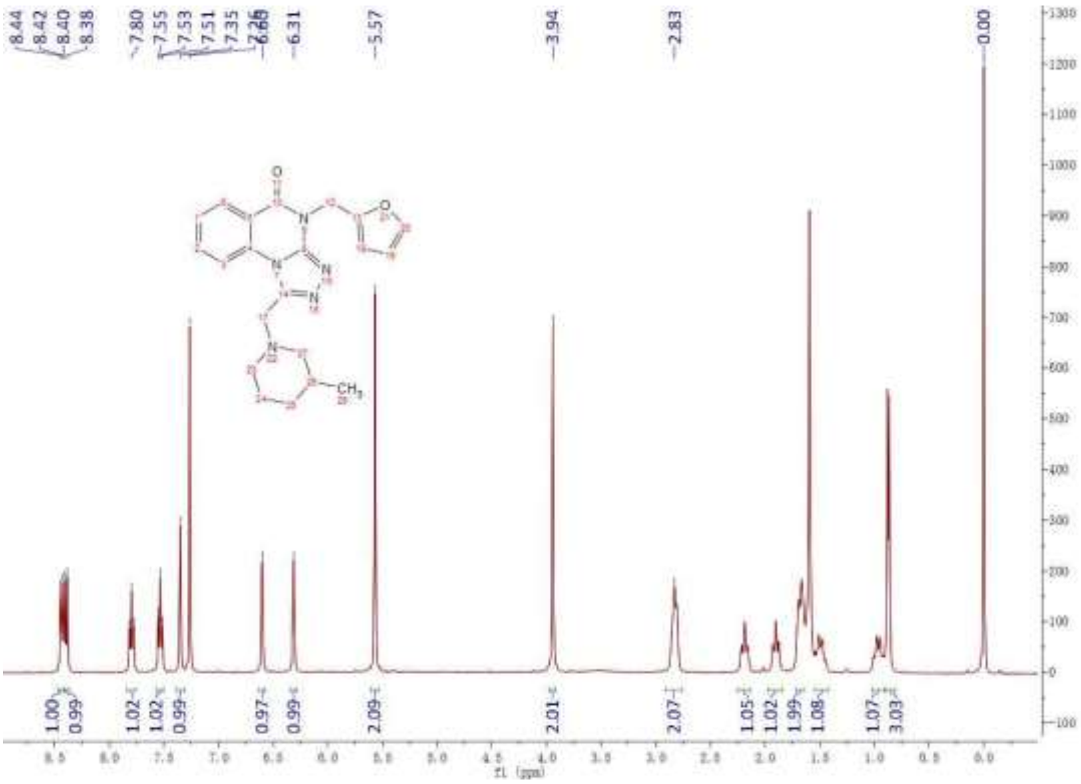

Figure S22. ^{1}H-NMR spectrum of **A5.**

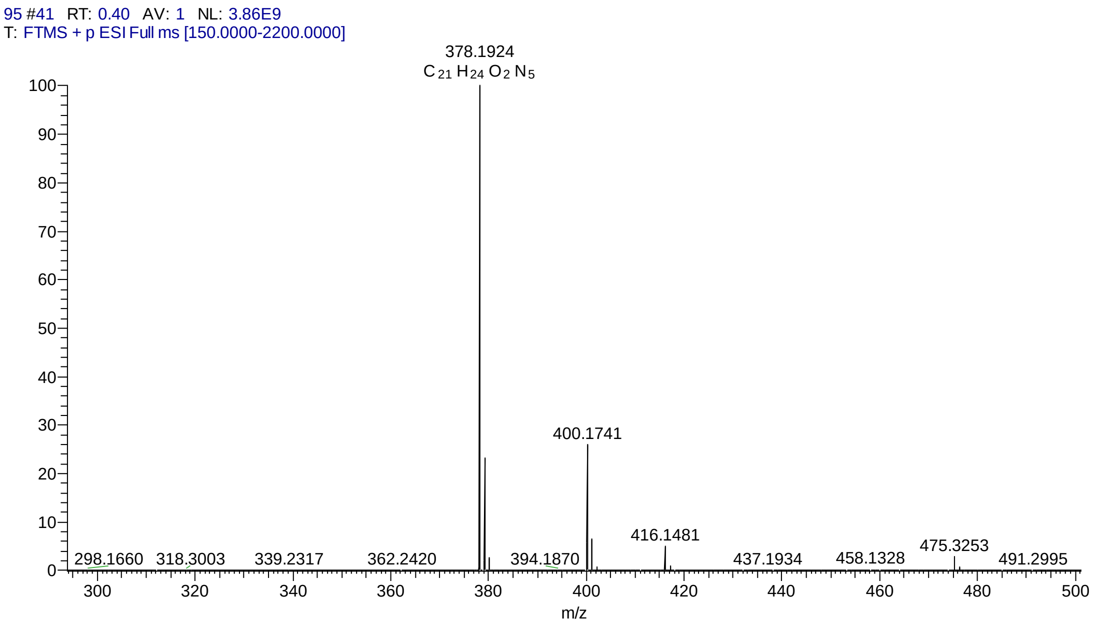

Figure S23. MS spectrum of **A5.**

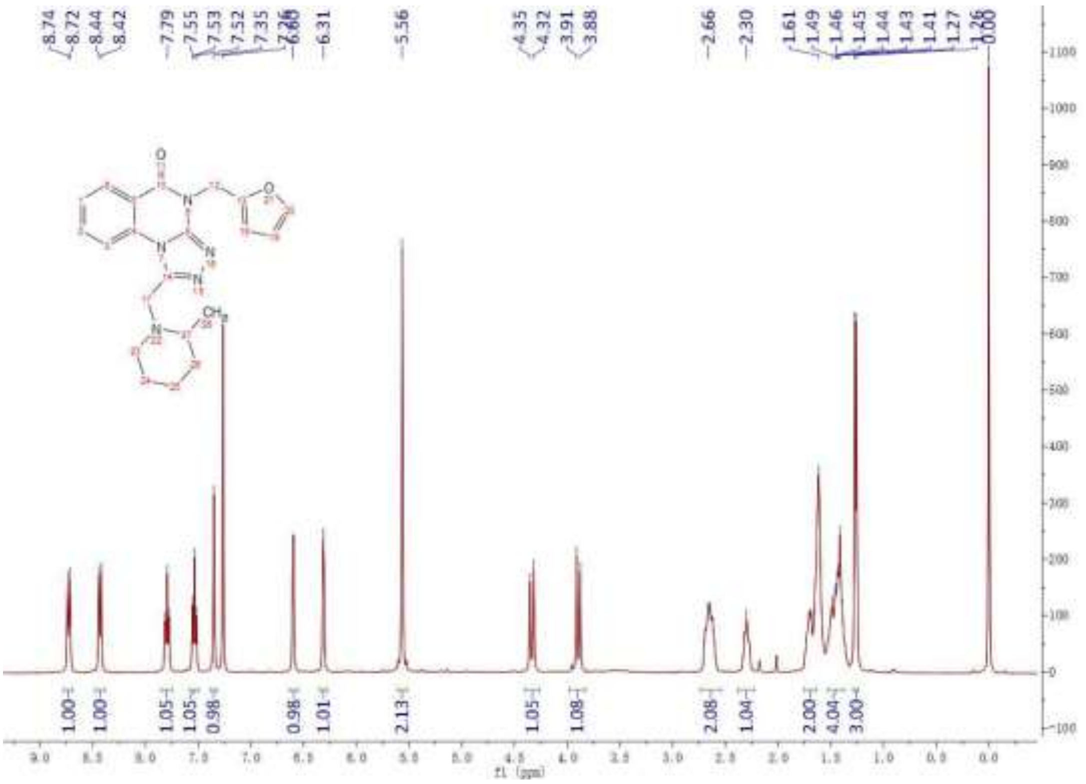

Figure S24. ^{1}H-NMR spectrum of **A6.**

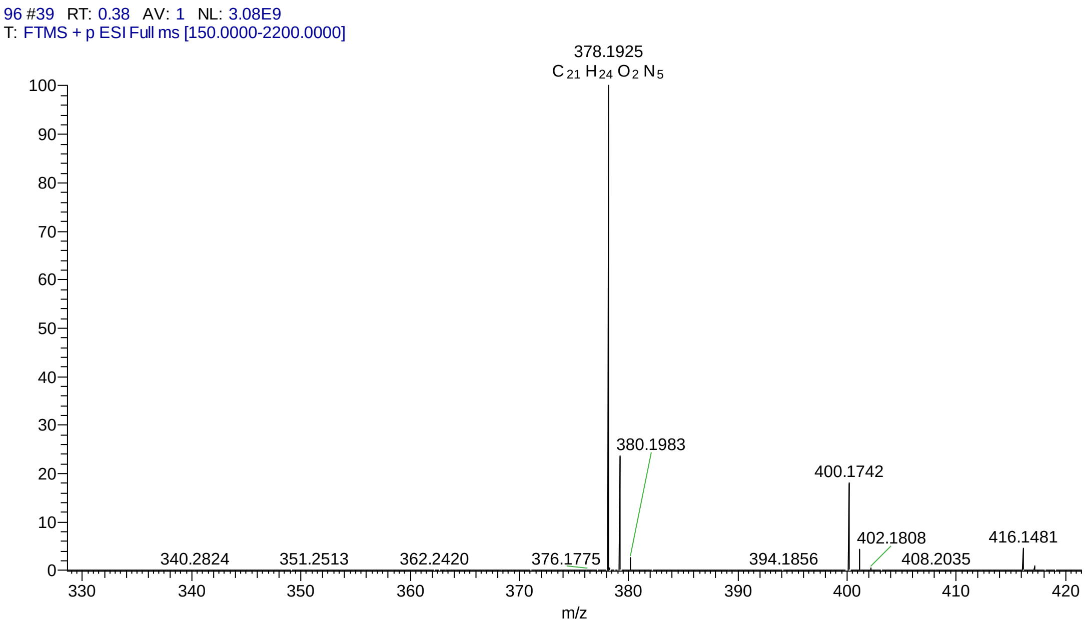

Figure S25. MS spectrum of **A6.**

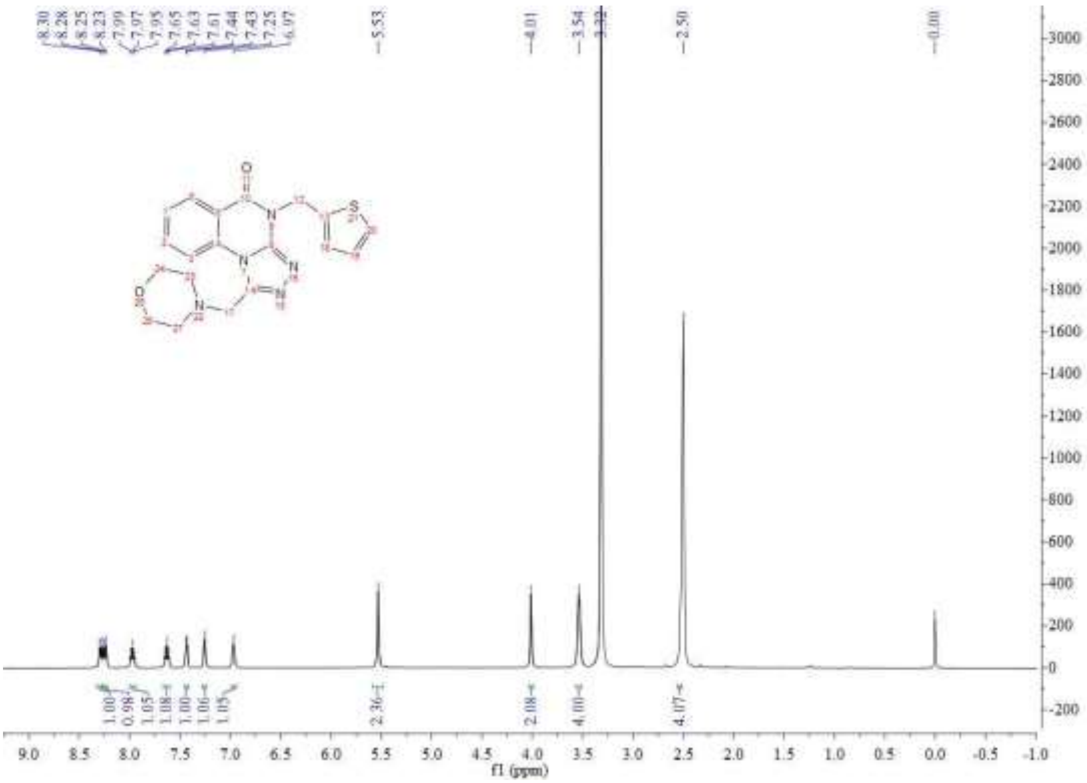

Figure S26. ^{1}H-NMR spectrum of **A7.**

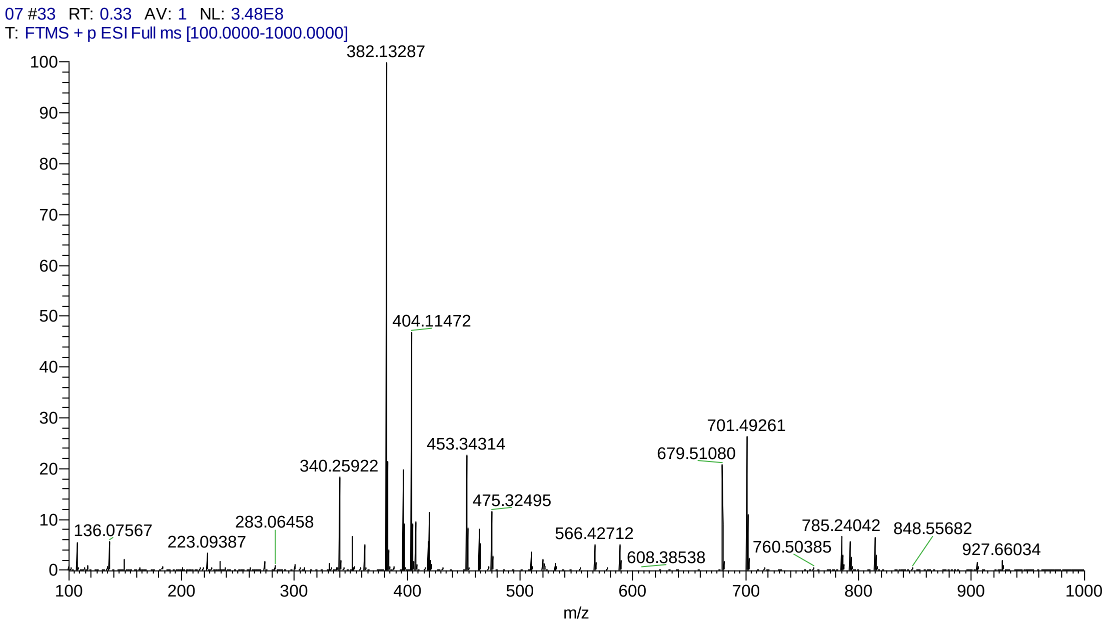

Figure S27. MS spectrum of **A7.**

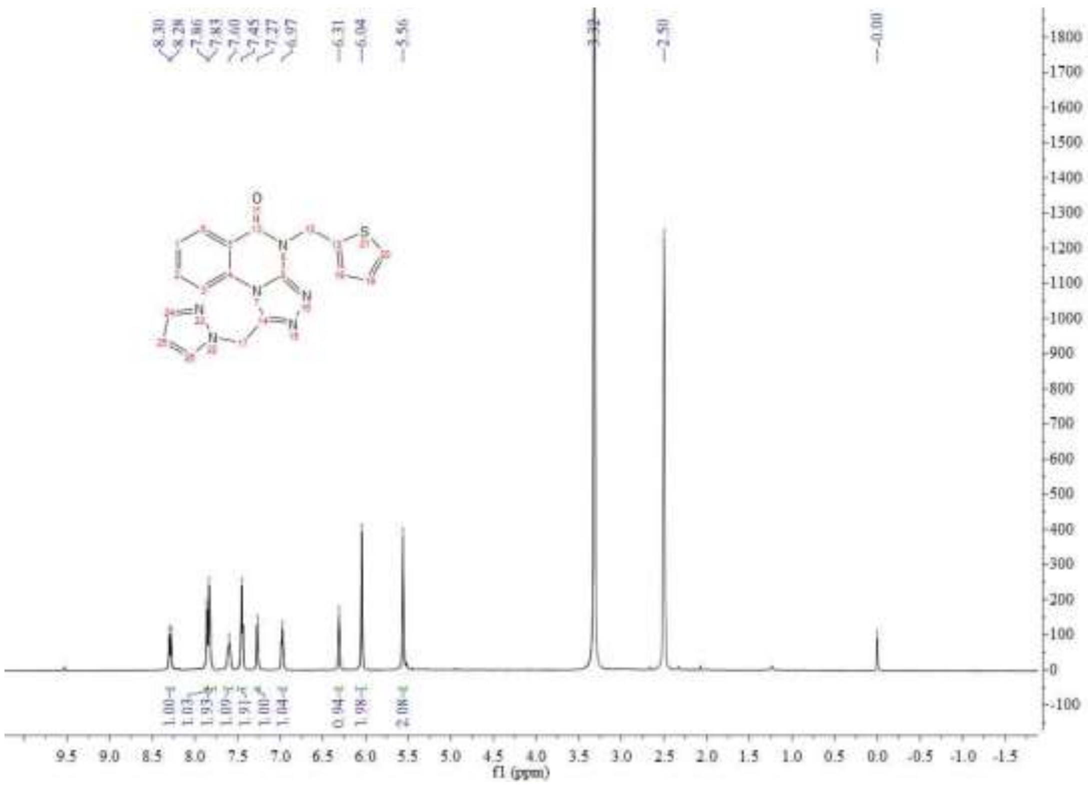

Figure S28. ^{1}H-NMR spectrum of **A8.**

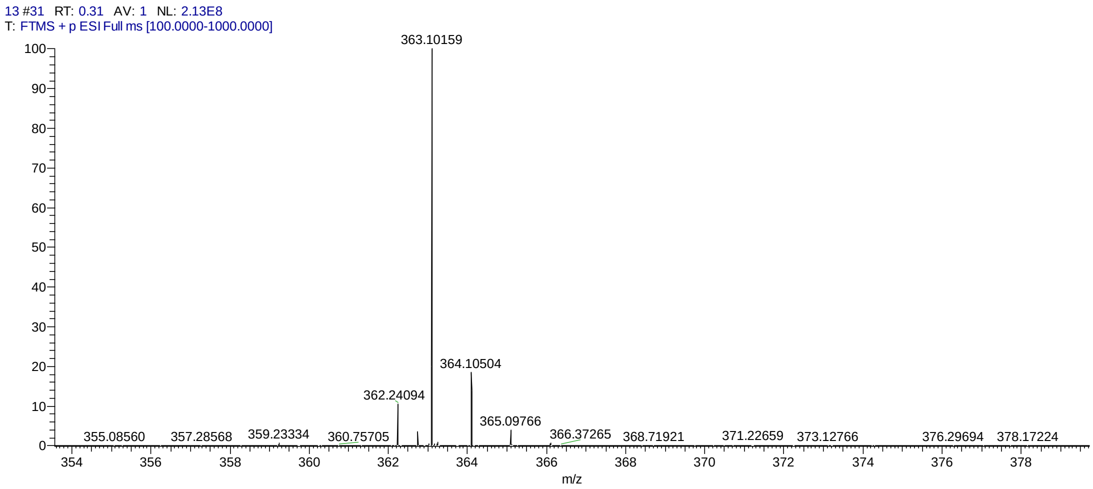

Figure S29. MS spectrum of **A8.**

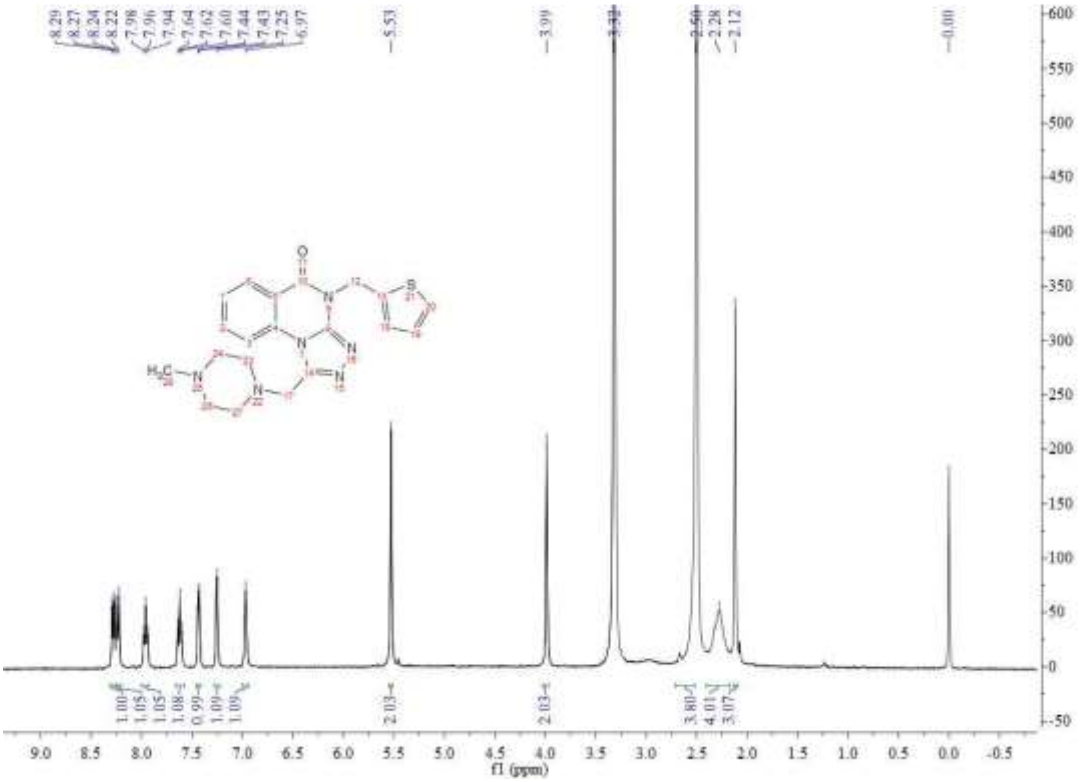

Figure S30. ^{1}H-NMR spectrum of **A9.**

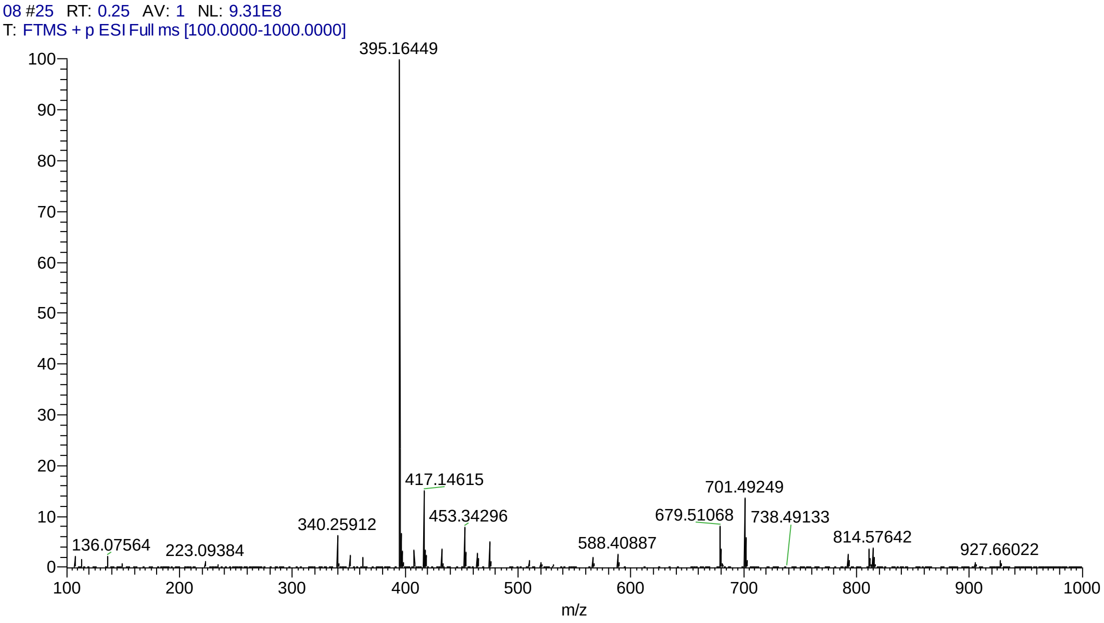

Figure S31. MS spectrum of **A9.**

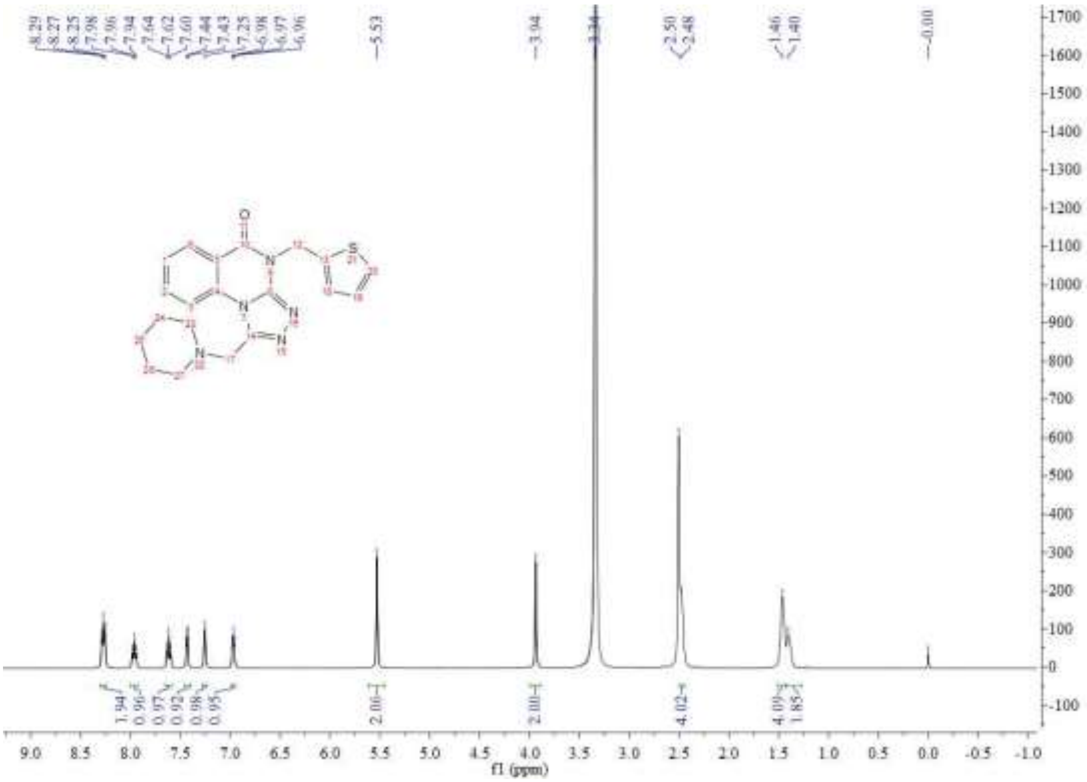

Figure S32. ^{1}H-NMR spectrum of **A10.**

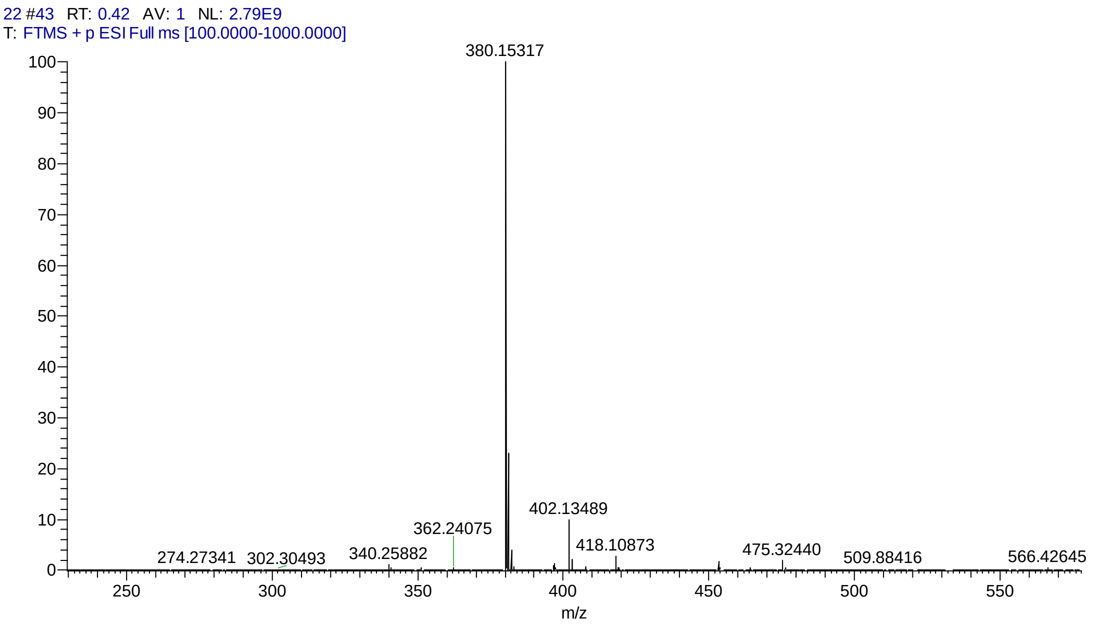

Figure S33. MS spectrum of **A10.**

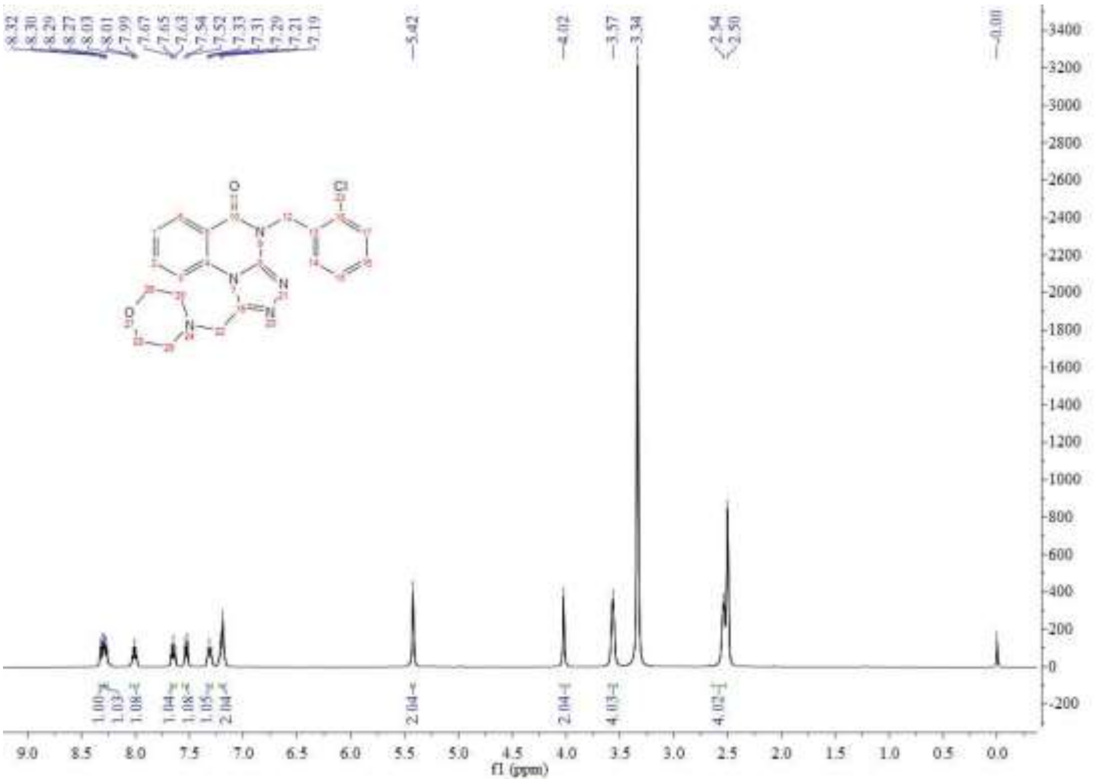

Figure S34. ^{1}H-NMR spectrum of **A11.**

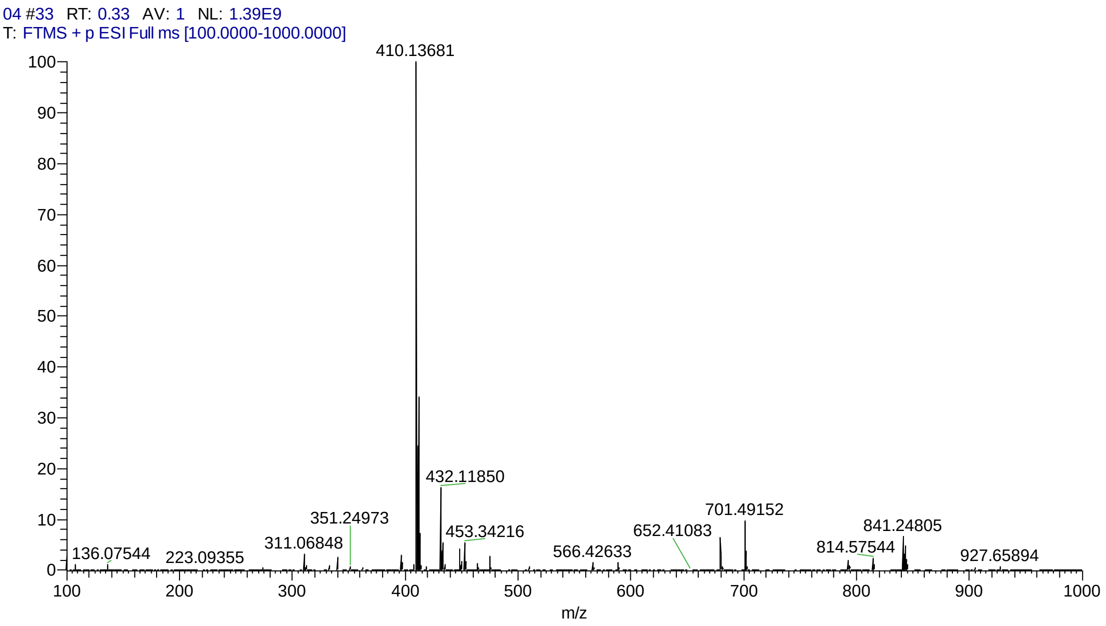

Figure S35. MS spectrum of **A11.**

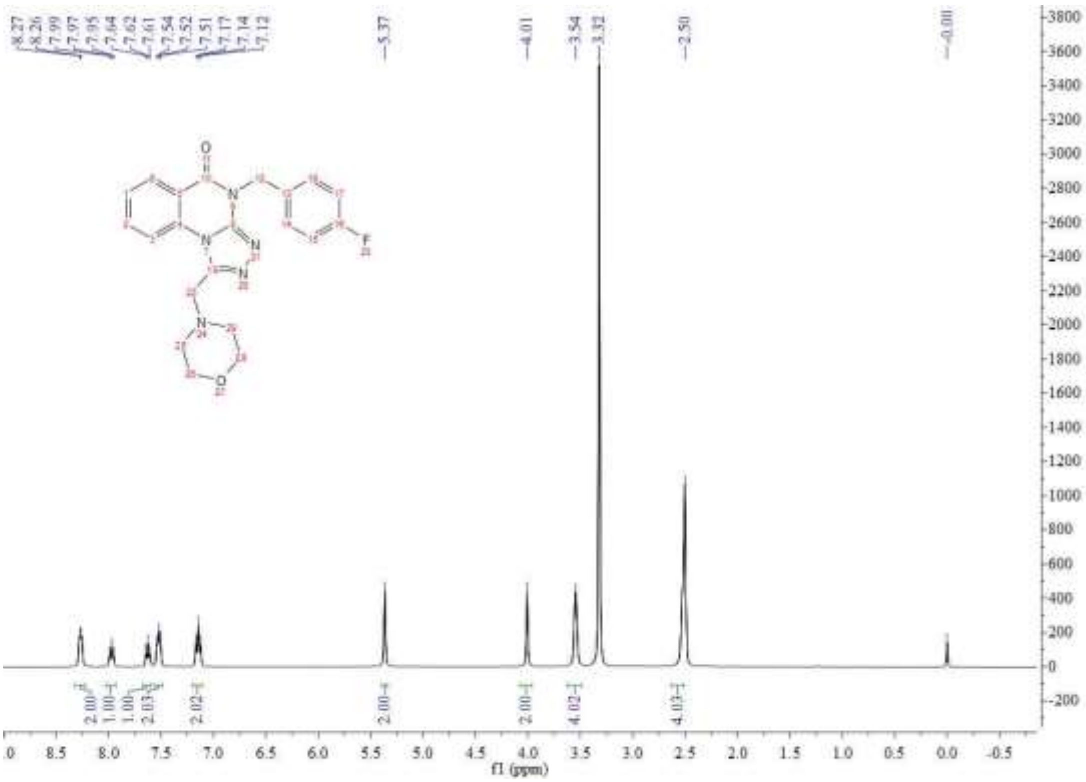

Figure S36. ^{1}H-NMR spectrum of **A12.**

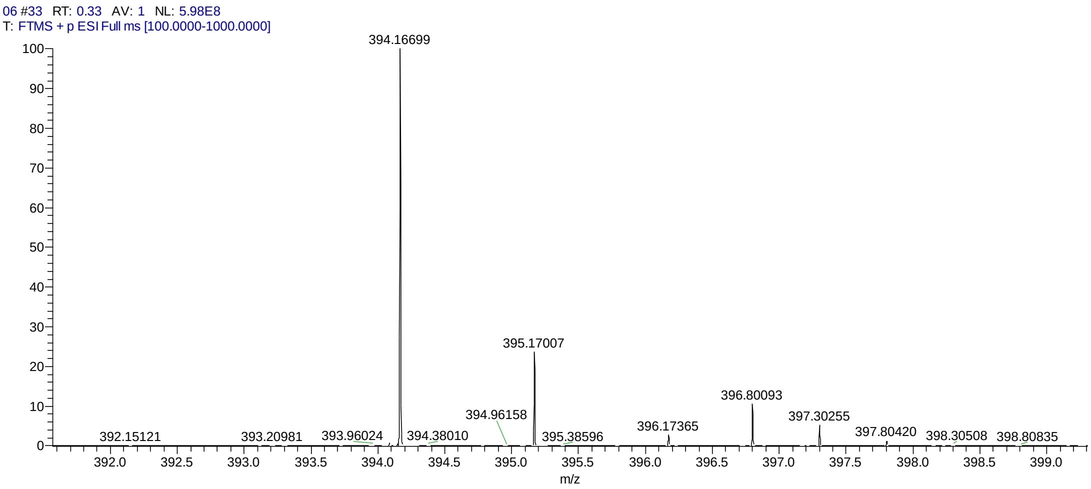

Figure S37. MS spectrum of **A12.**

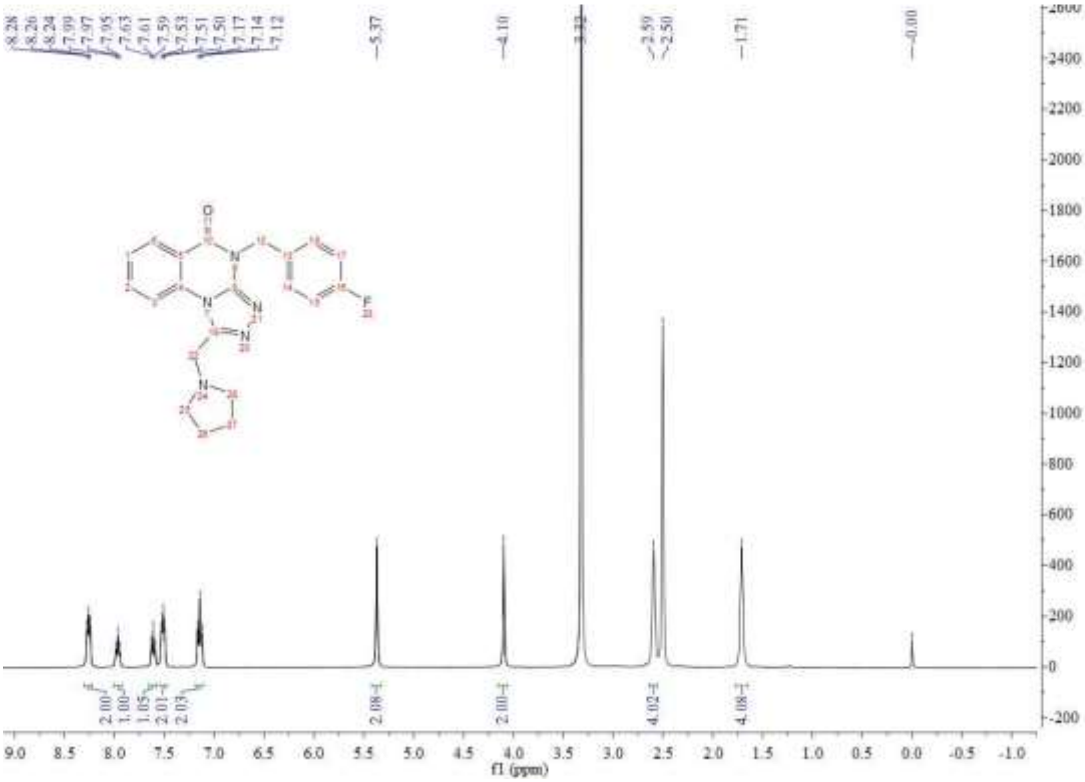

Figure S38. ^{1}H-NMR spectrum of **A13.**

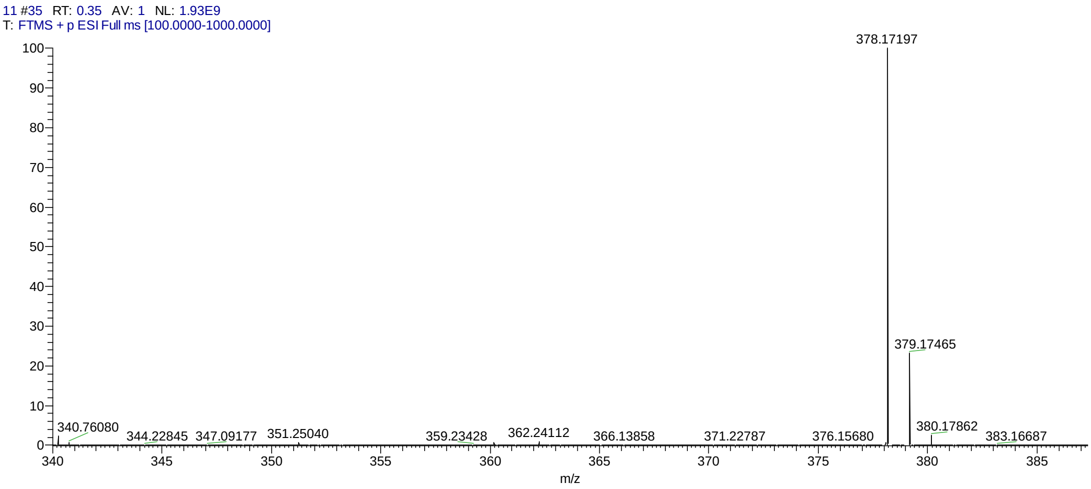

Figure S39. MS spectrum of **A13.**

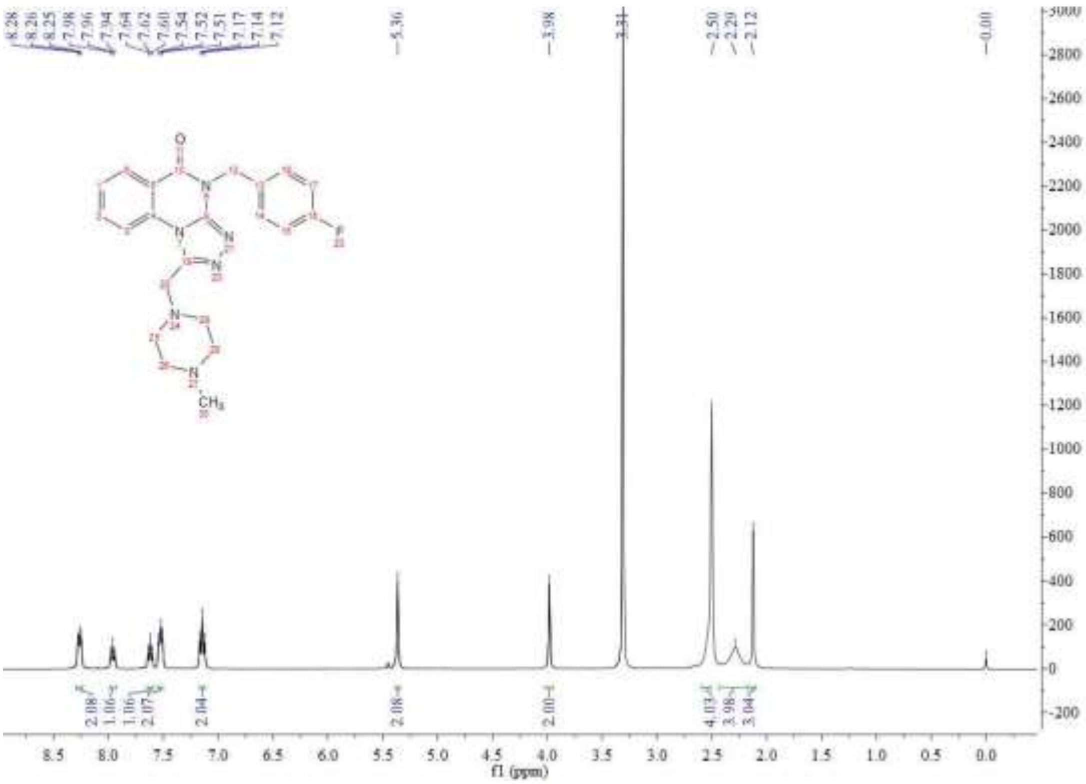

Figure S40. ^{1}H-NMR spectrum of **A14.**

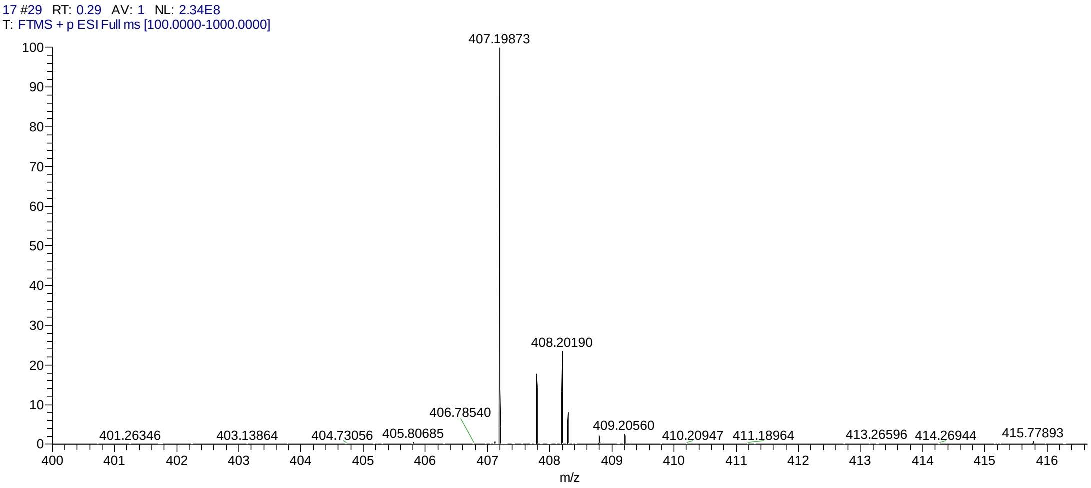

Figure S41. MS spectrum of **A14.**

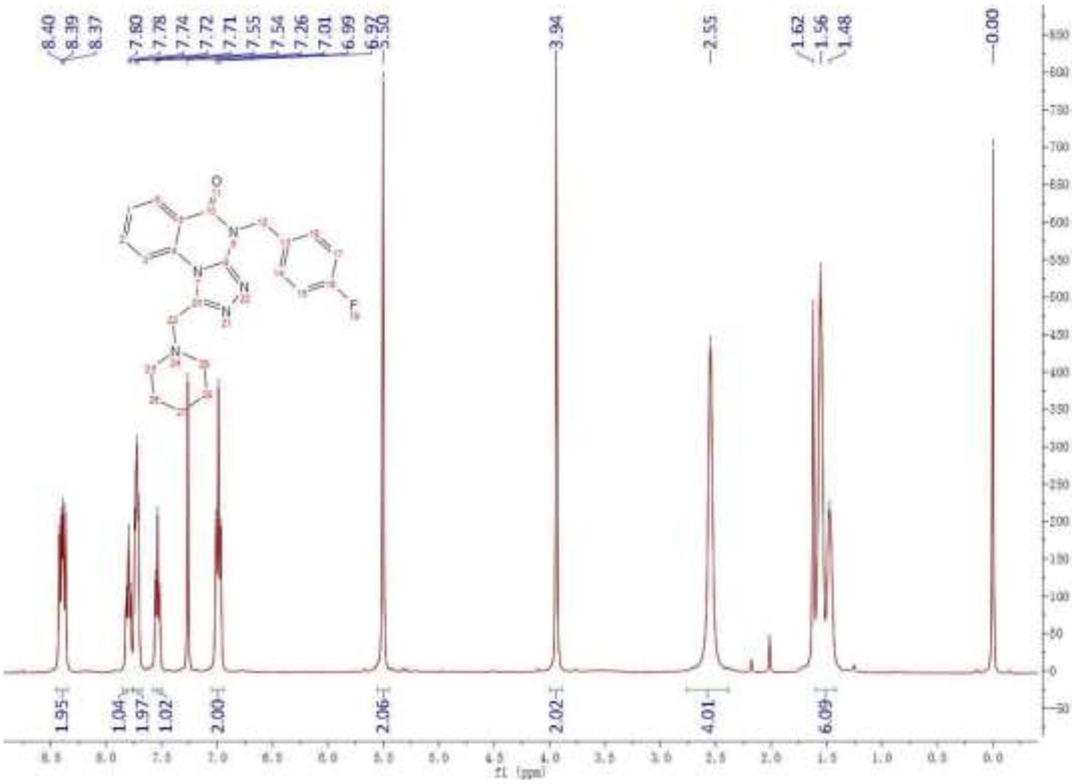

Figure S42. ^{1}H-NMR spectrum of **A15.**

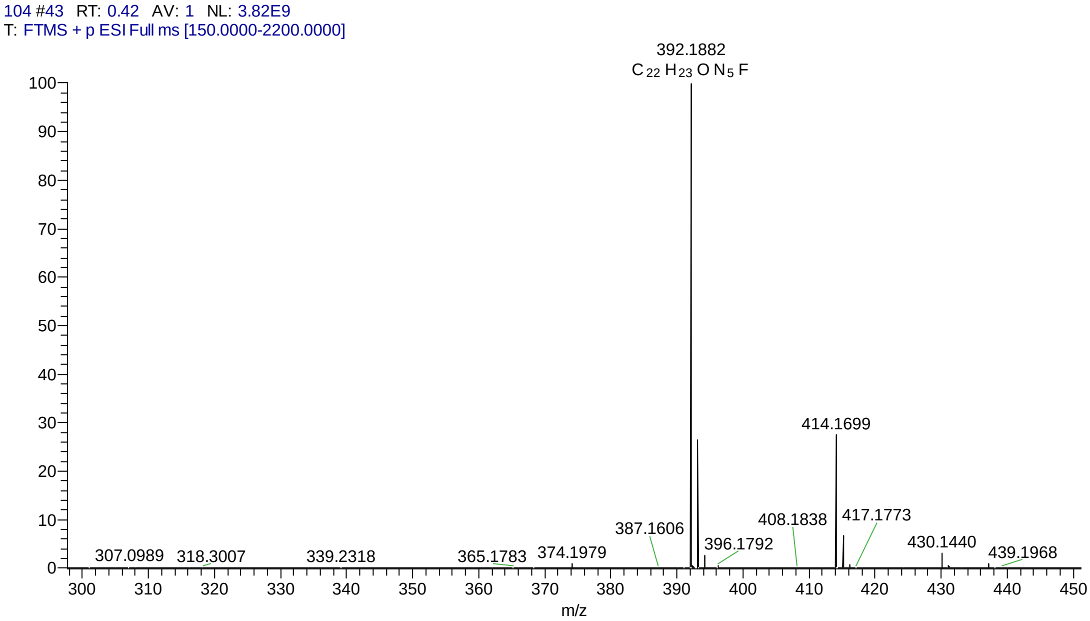

Figure S43. MS spectrum of **A15.**

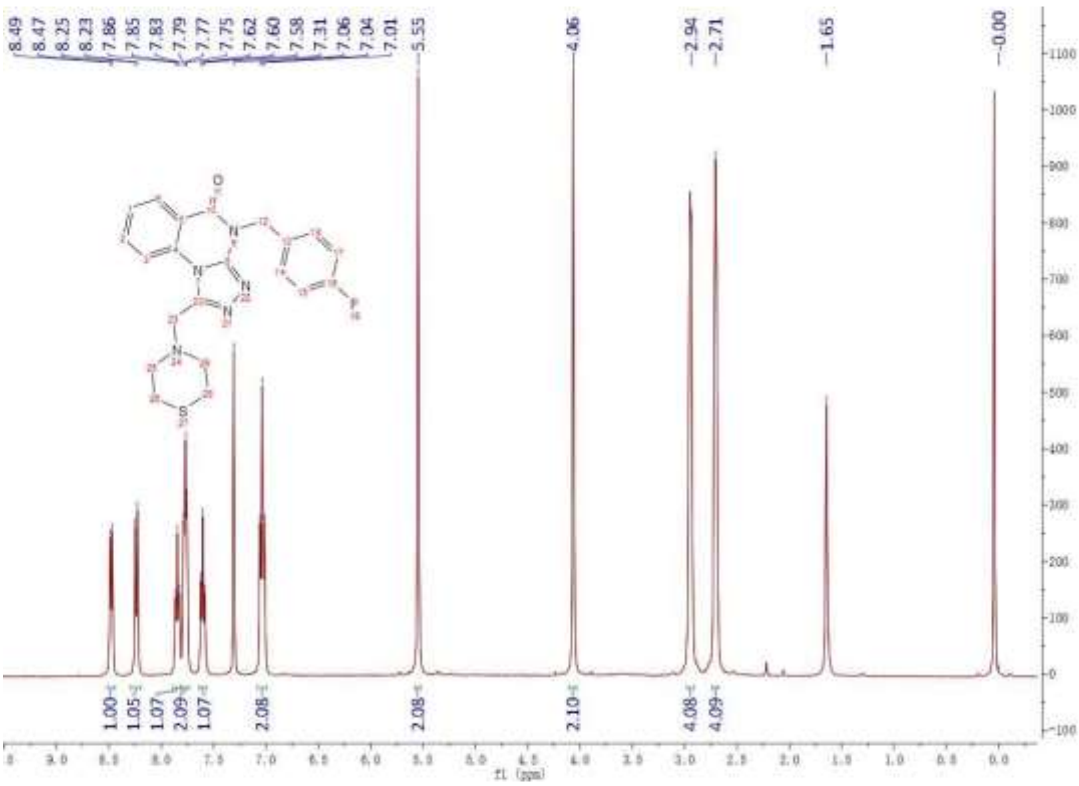

Figure S44. ^{1}H-NMR spectrum of **A16.**

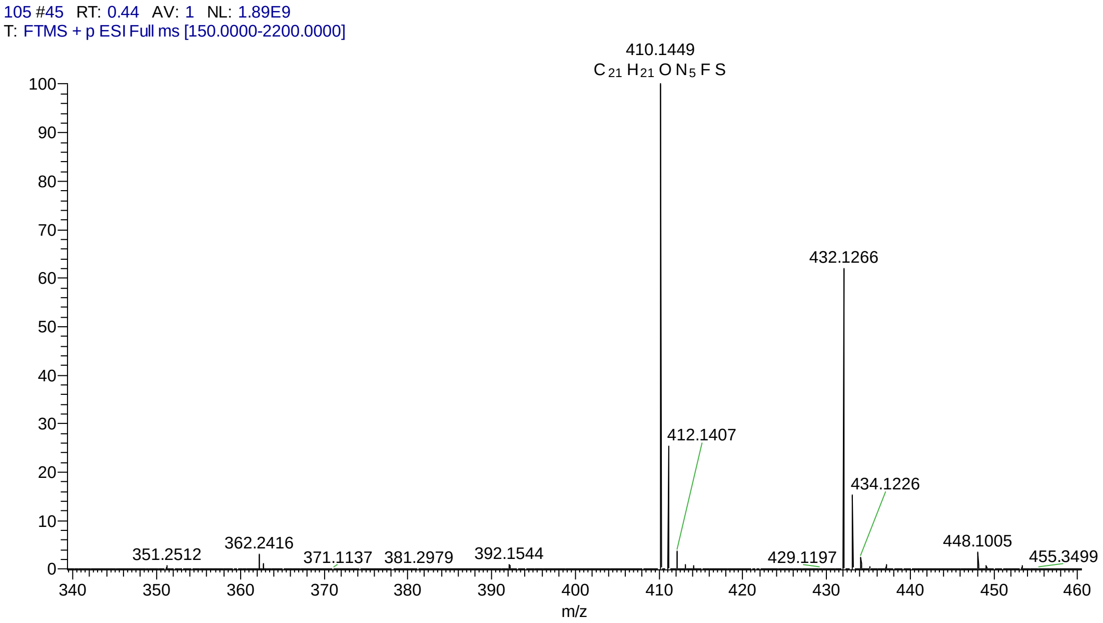

Figure S45. MS spectrum of **A16.**

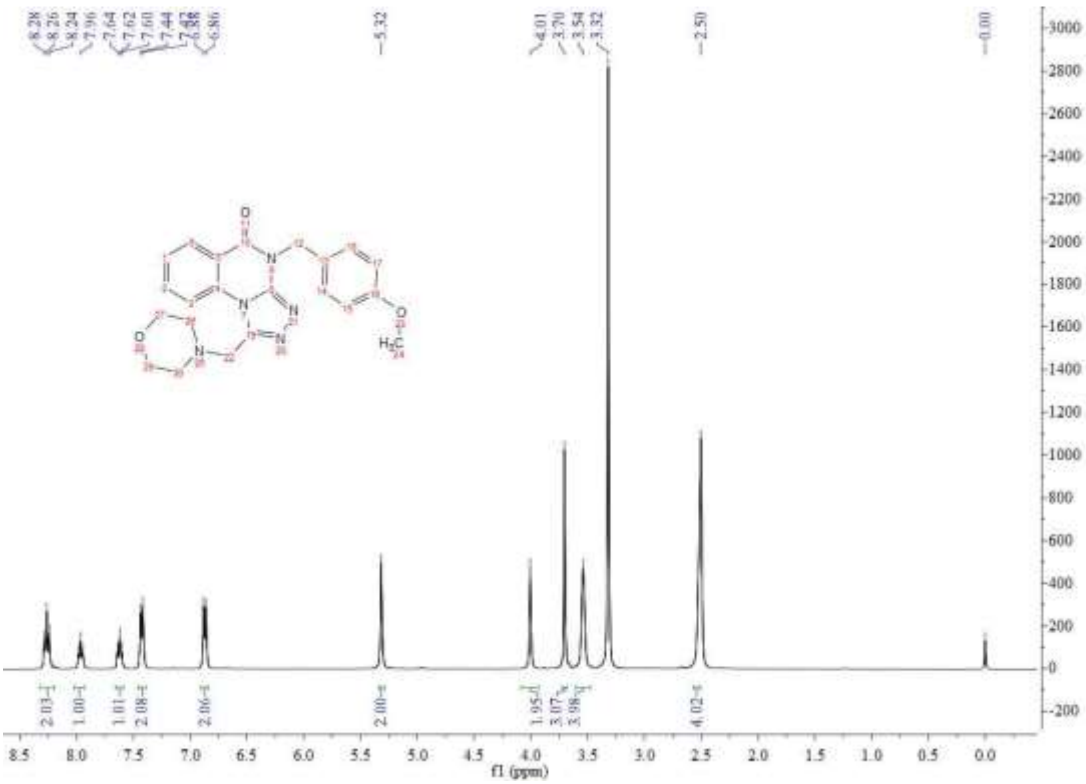

Figure S46. ^{1}H-NMR spectrum of **A17.**

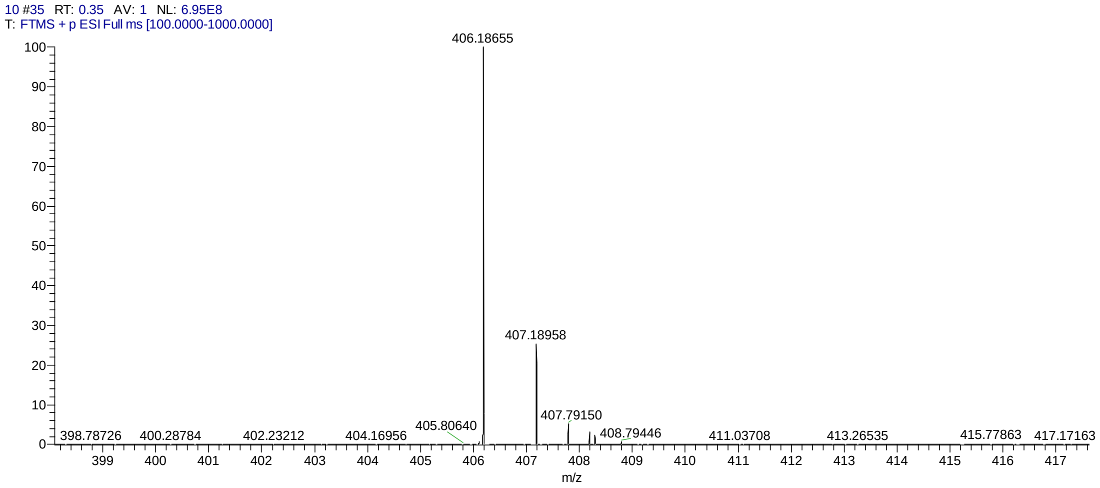

Figure S47. MS spectrum of **A17.**

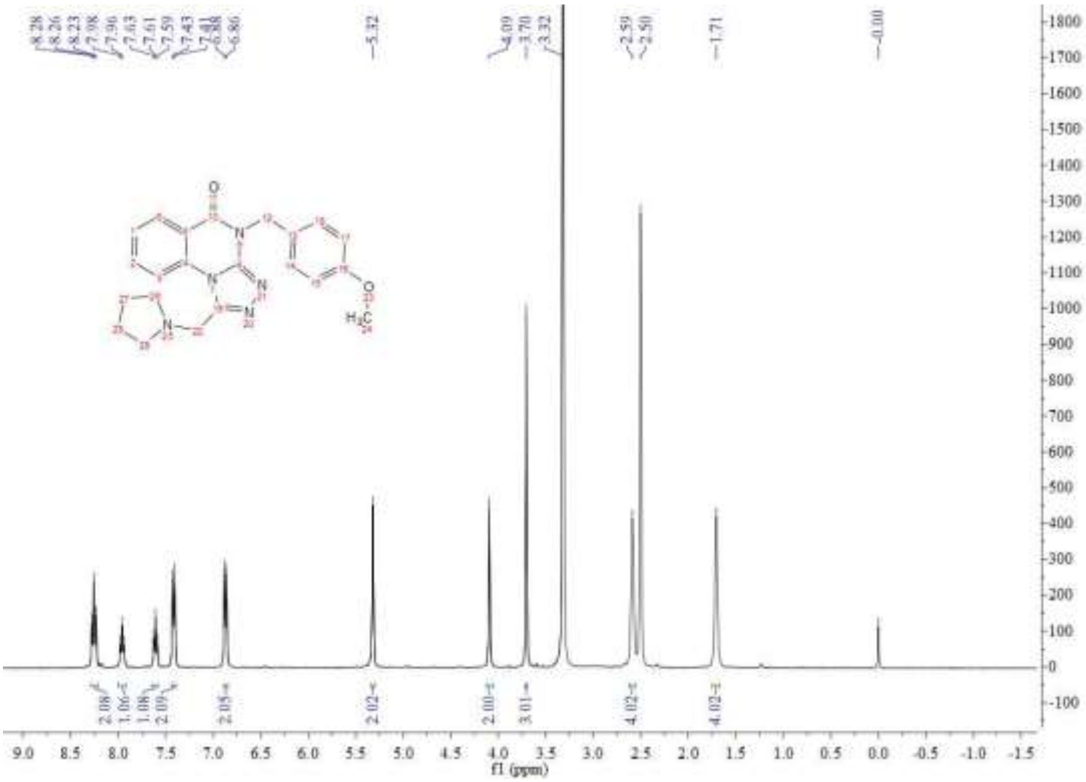

Figure S48. ^{1}H-NMR spectrum of **A18.**

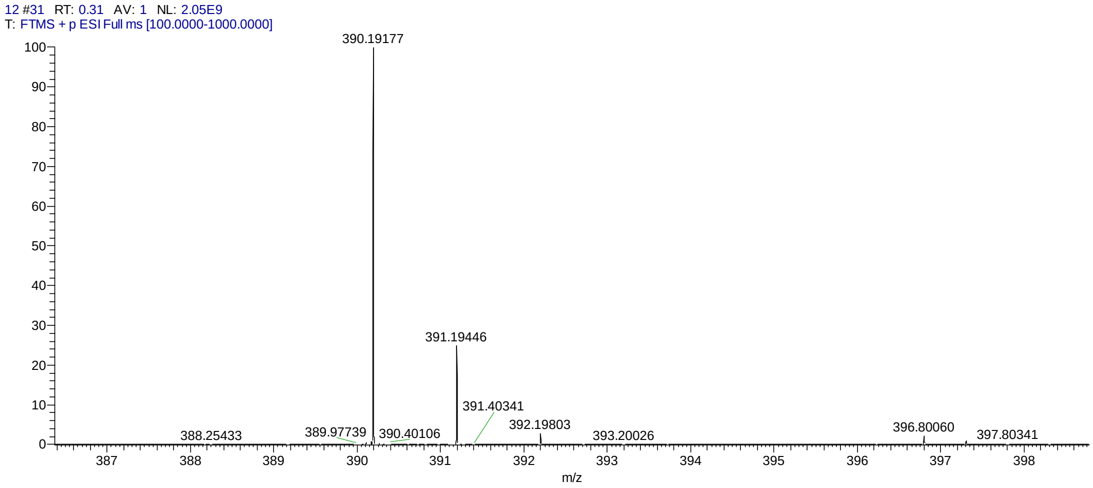

Figure S49. MS spectrum of **A18.**

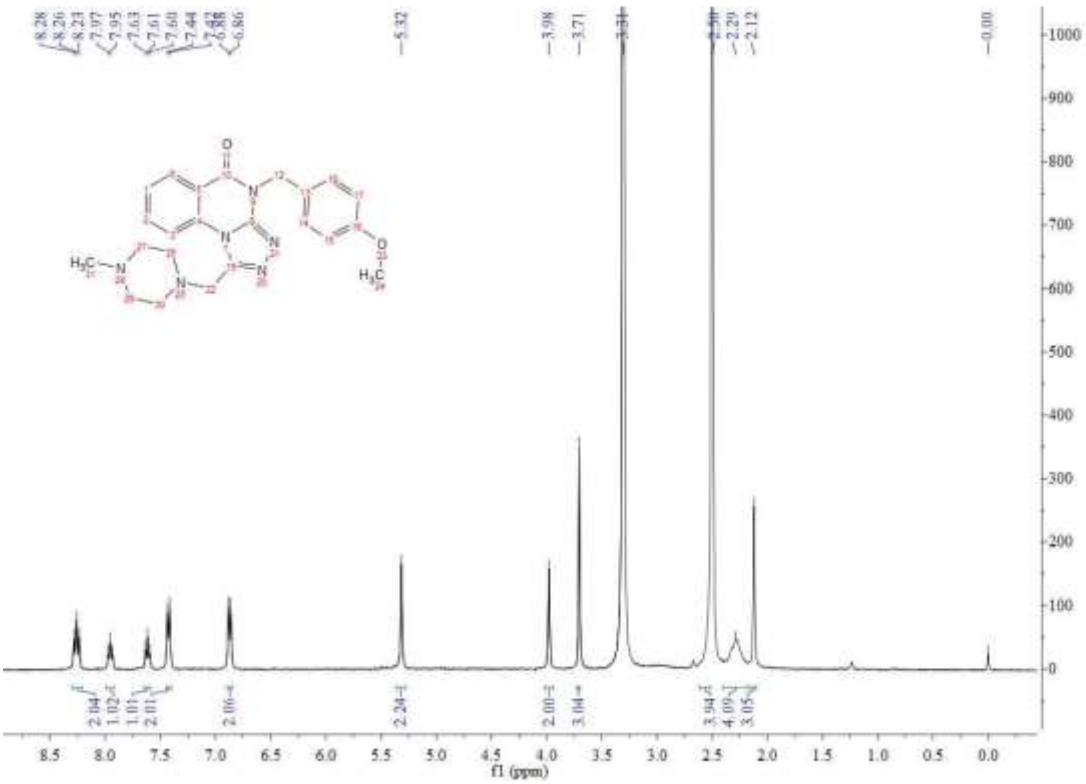

Figure S50. ^{1}H-NMR spectrum of **A19.**

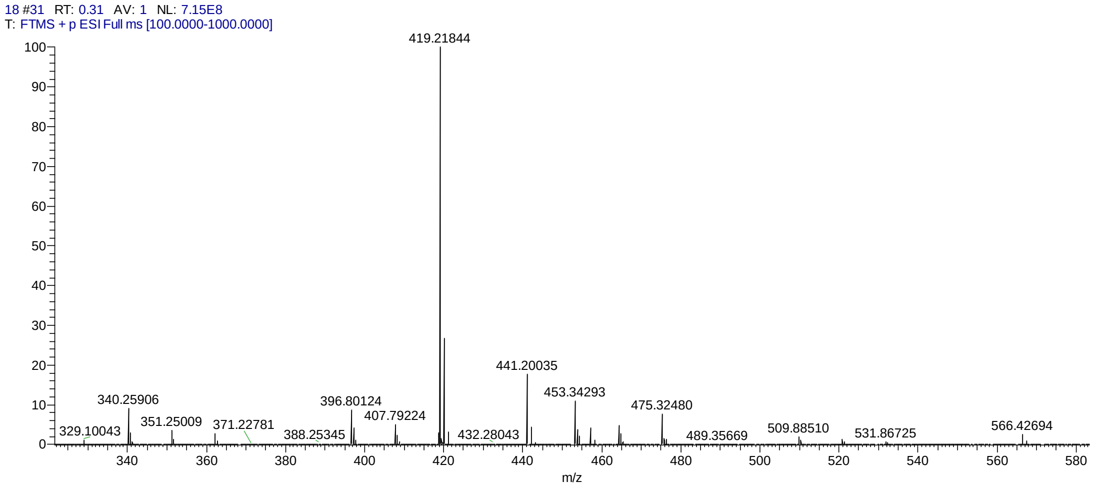

Figure S51. MS spectrum of **A19.**

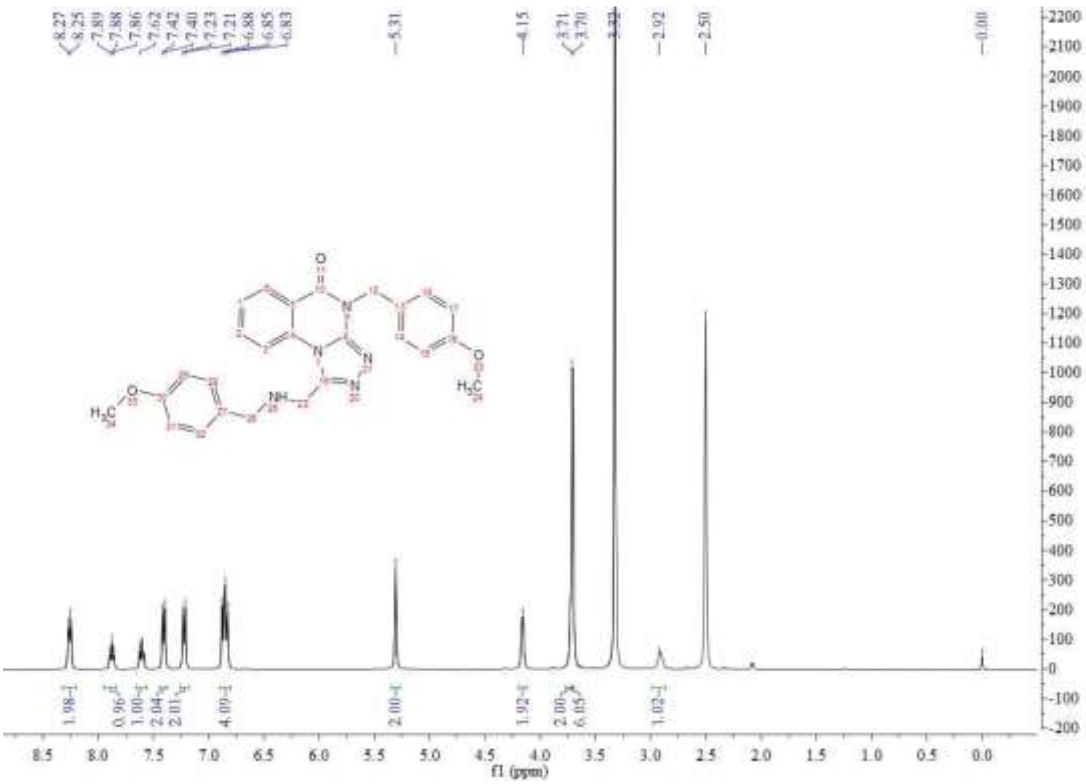

Figure S52. ^{1}H-NMR spectrum of **A20.**

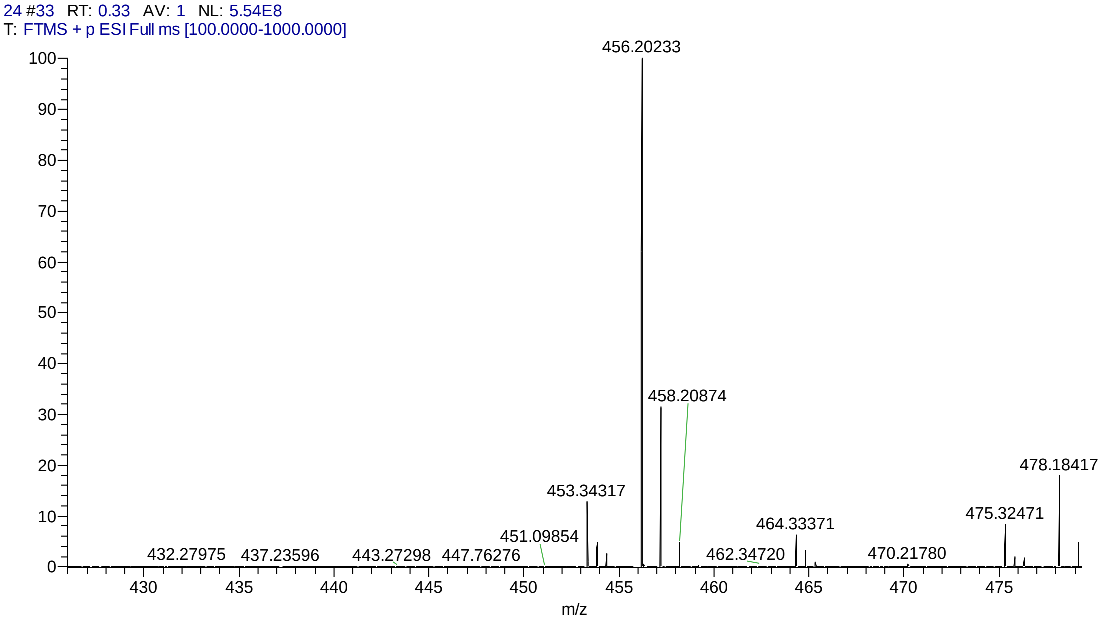

Figure S53. MS spectrum of **A20.**

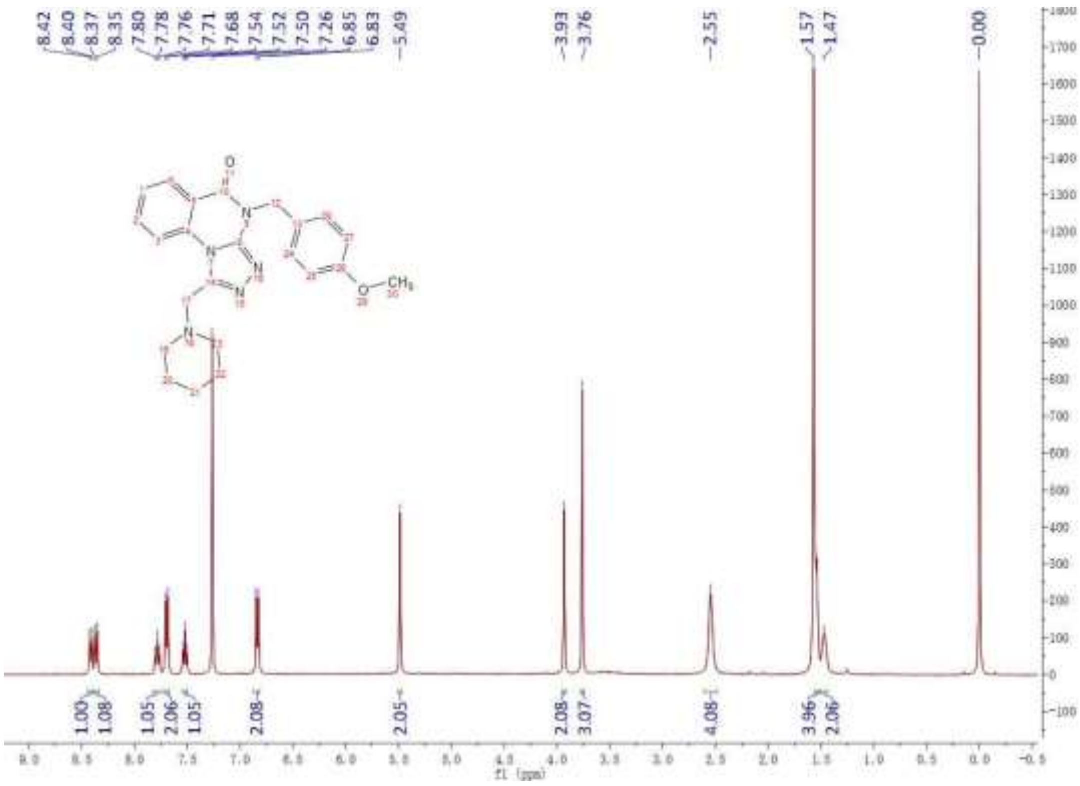

Figure S54. ^{1}H-NMR spectrum of **A21.**

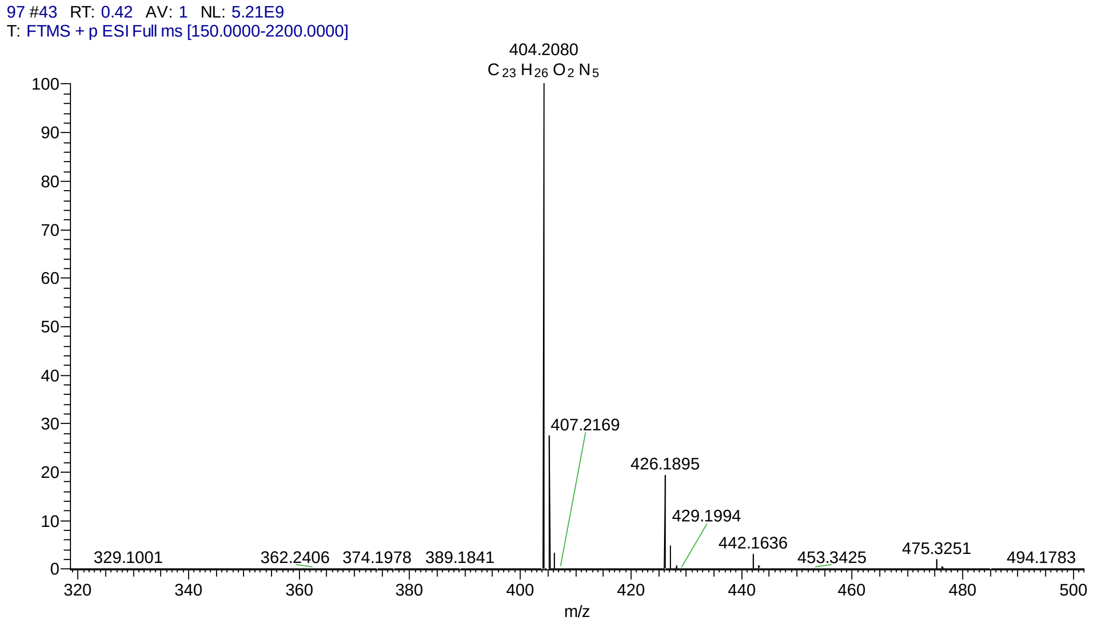

Figure S55. MS spectrum of **A21.**

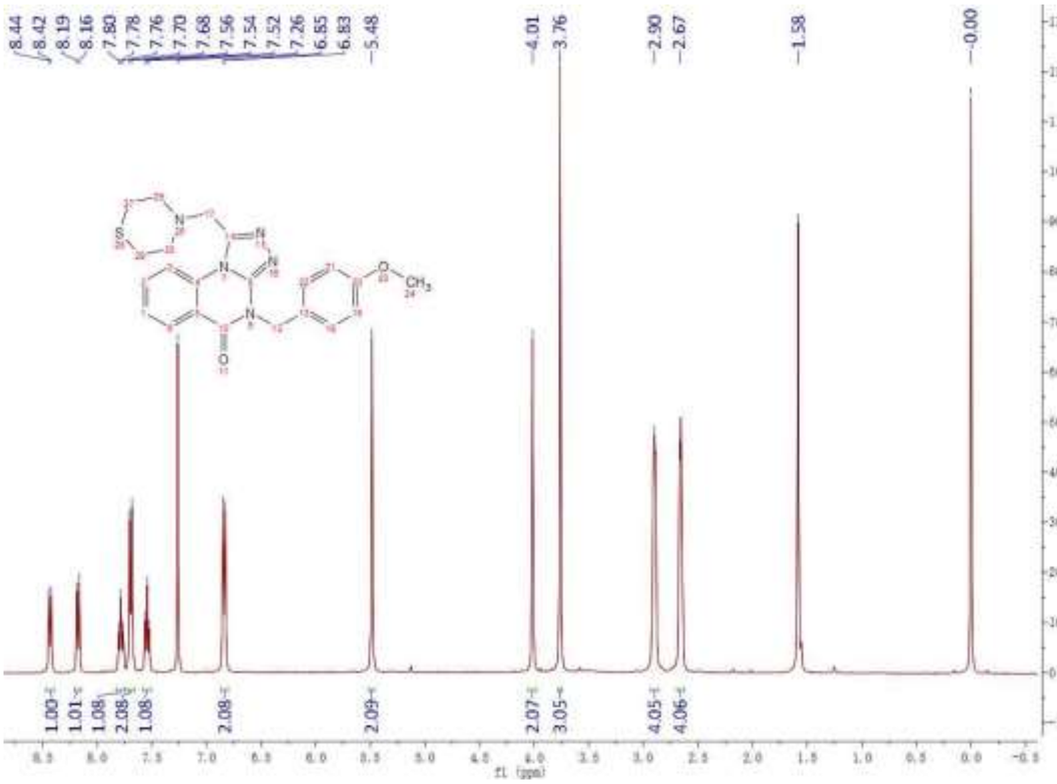

Figure S56. ^{1}H-NMR spectrum of **A22.**

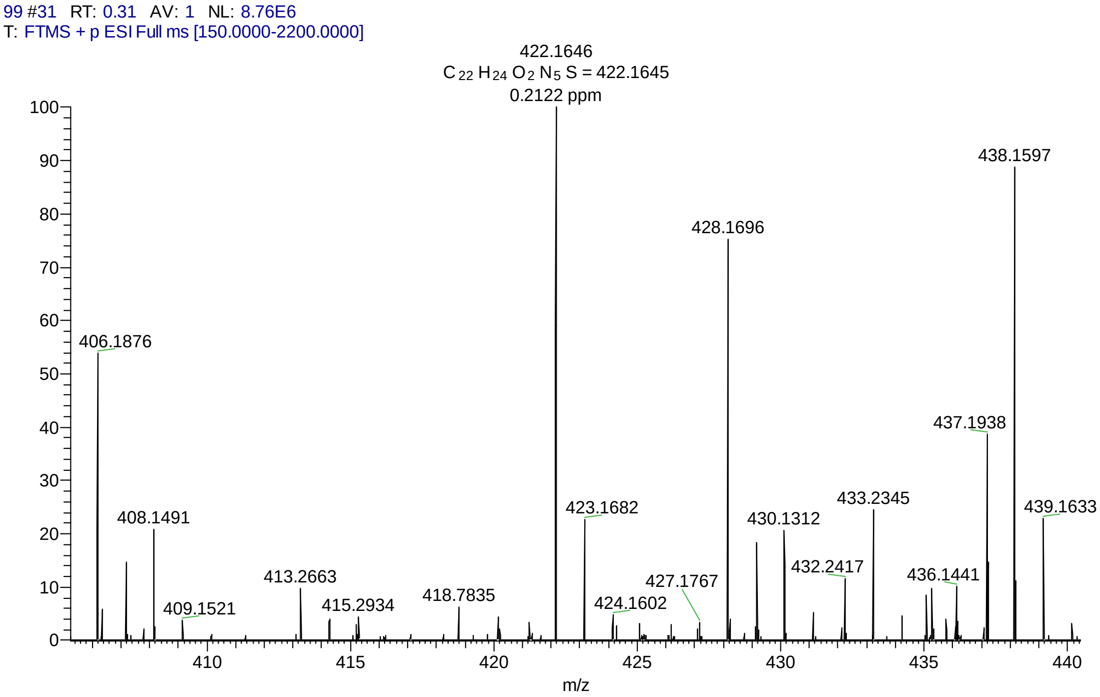

Figure S57. MS spectrum of **A22.**

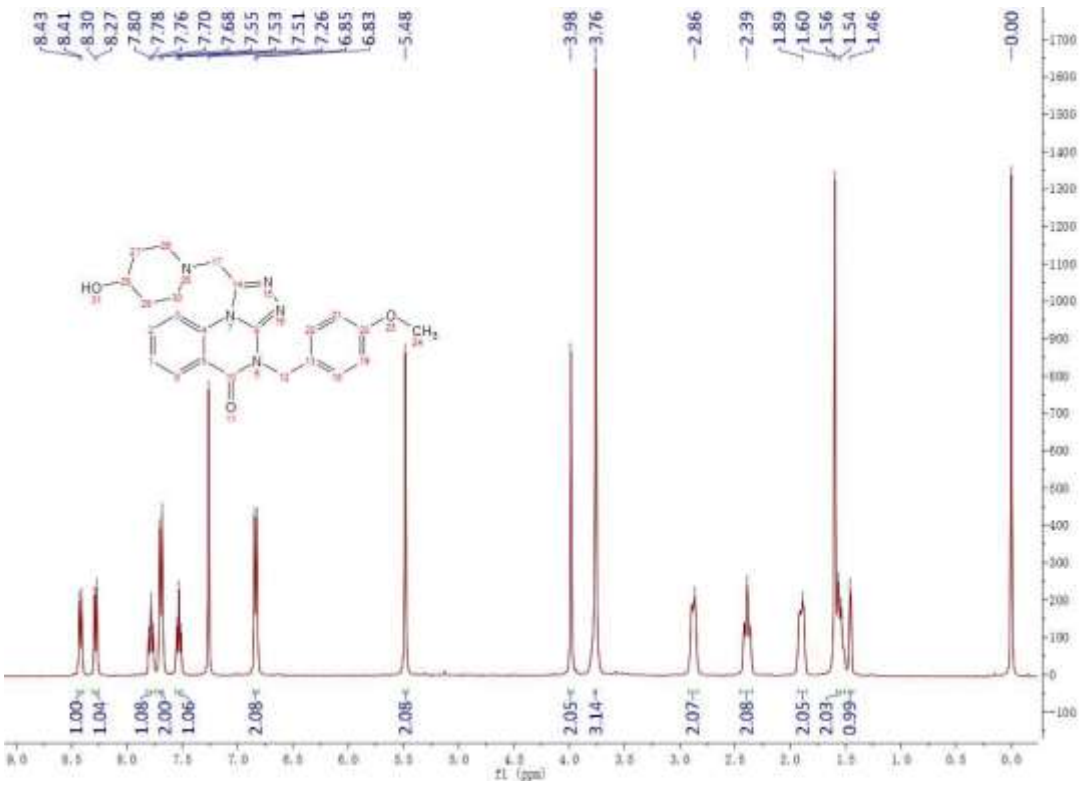

Figure S58. ^{1}H-NMR spectrum of **A23.**

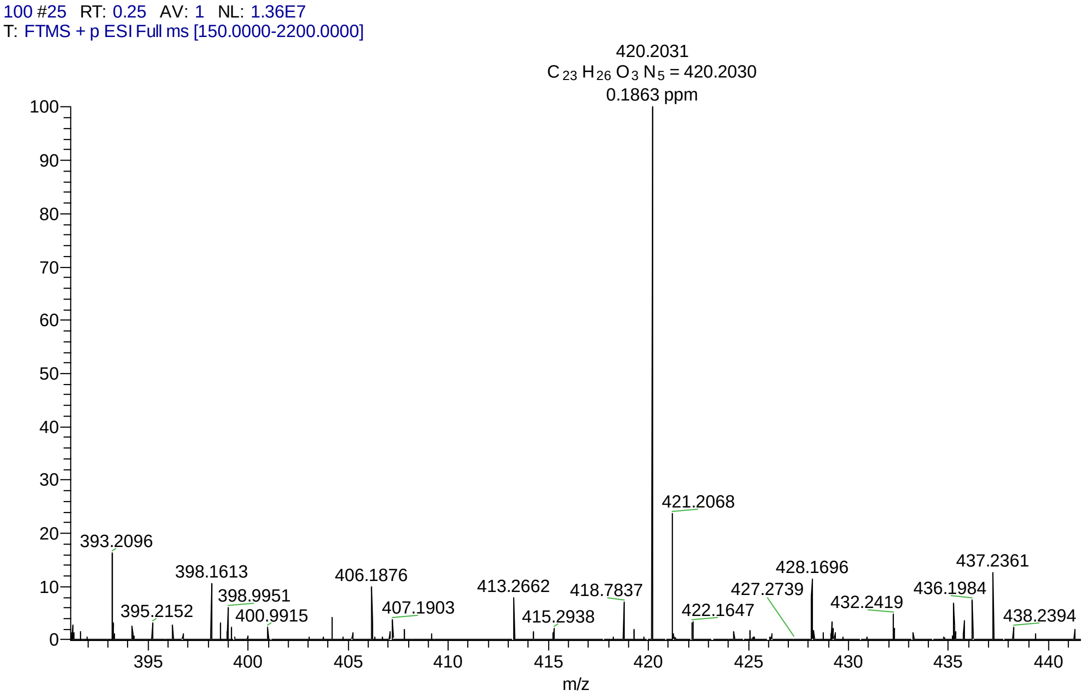

Figure S59. MS spectrum of **A23.**

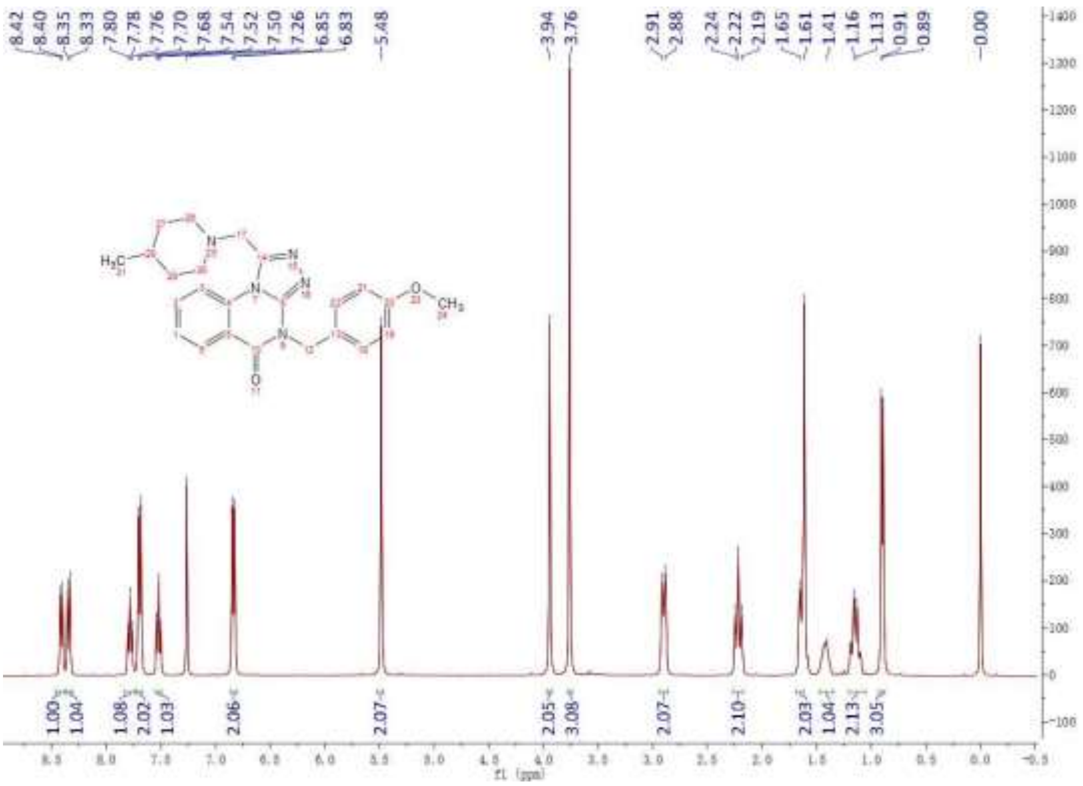

Figure S60. ^{1}H-NMR spectrum of **A24.**

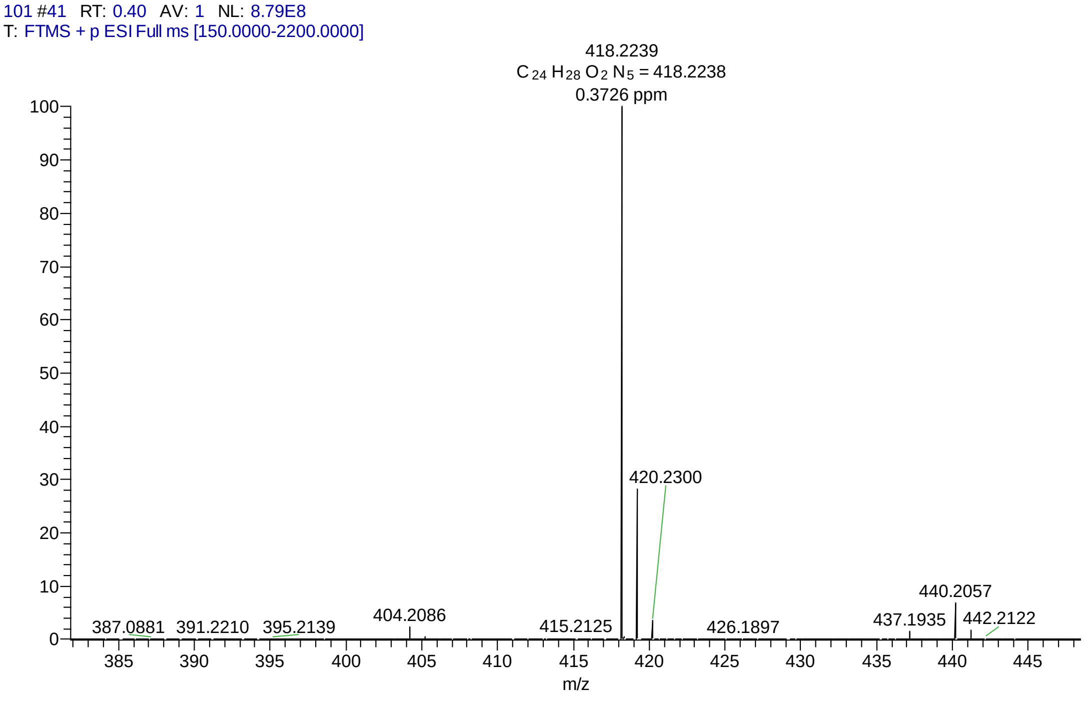

Figure S61. MS spectrum of **A24.**

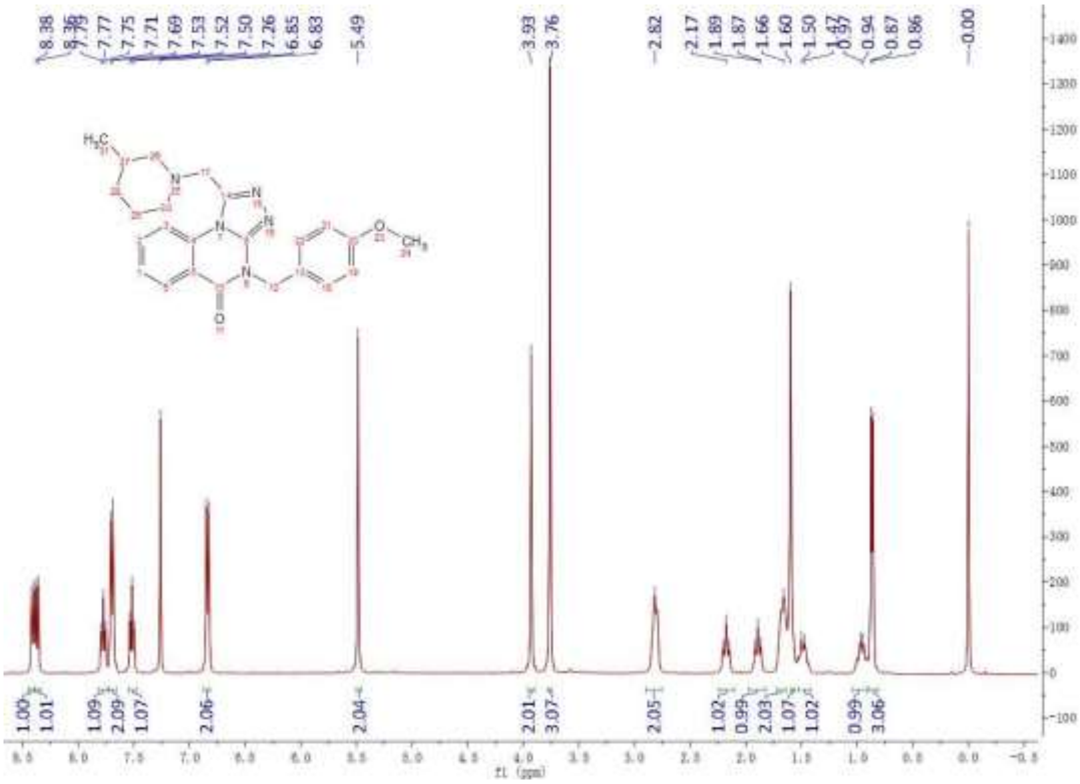

Figure S62. ^{1}H-NMR spectrum of **A25.**

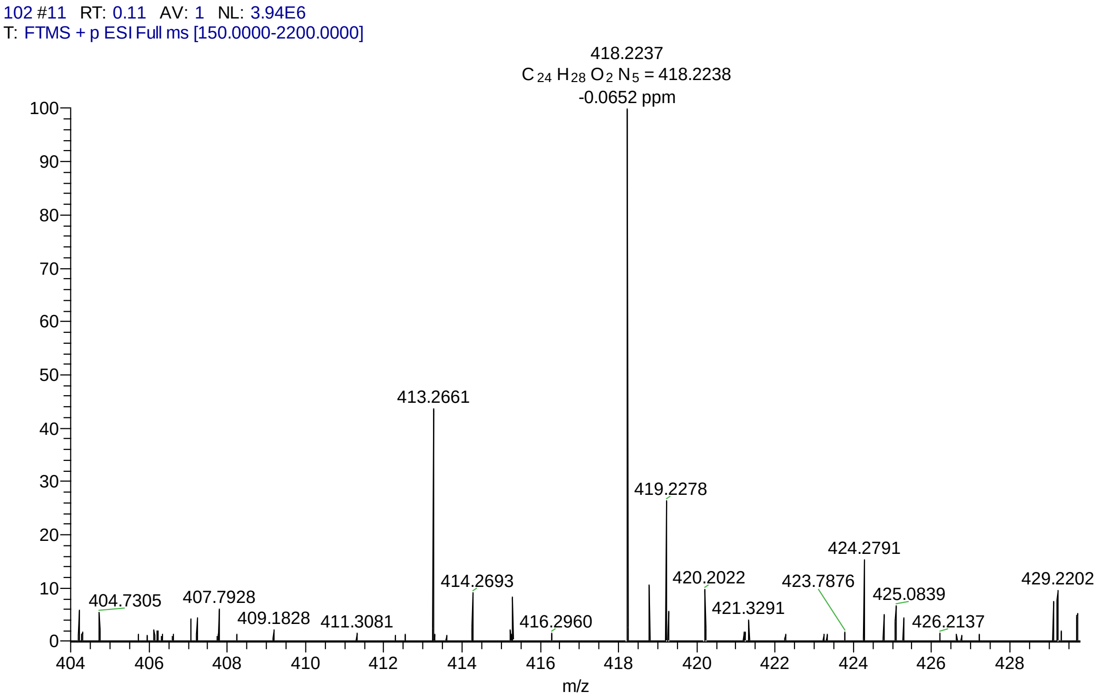

Figure S63. MS spectrum of **A25.**

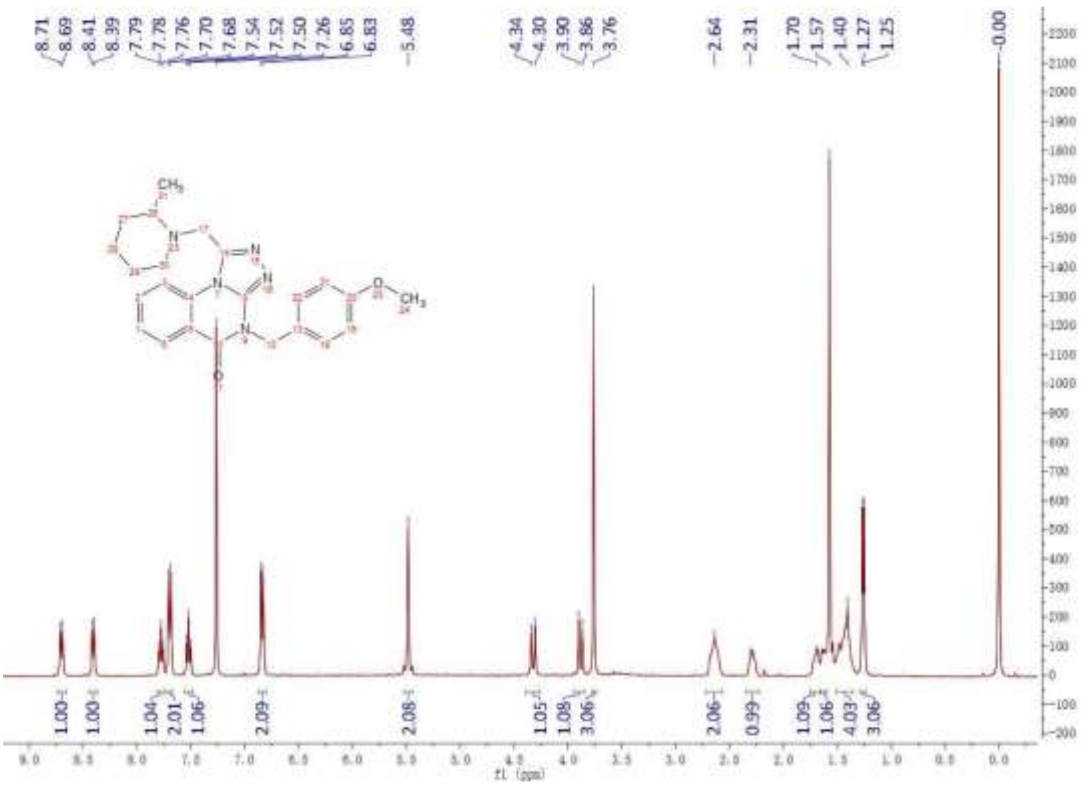

Figure S64. ^{1}H-NMR spectrum of **A26.**

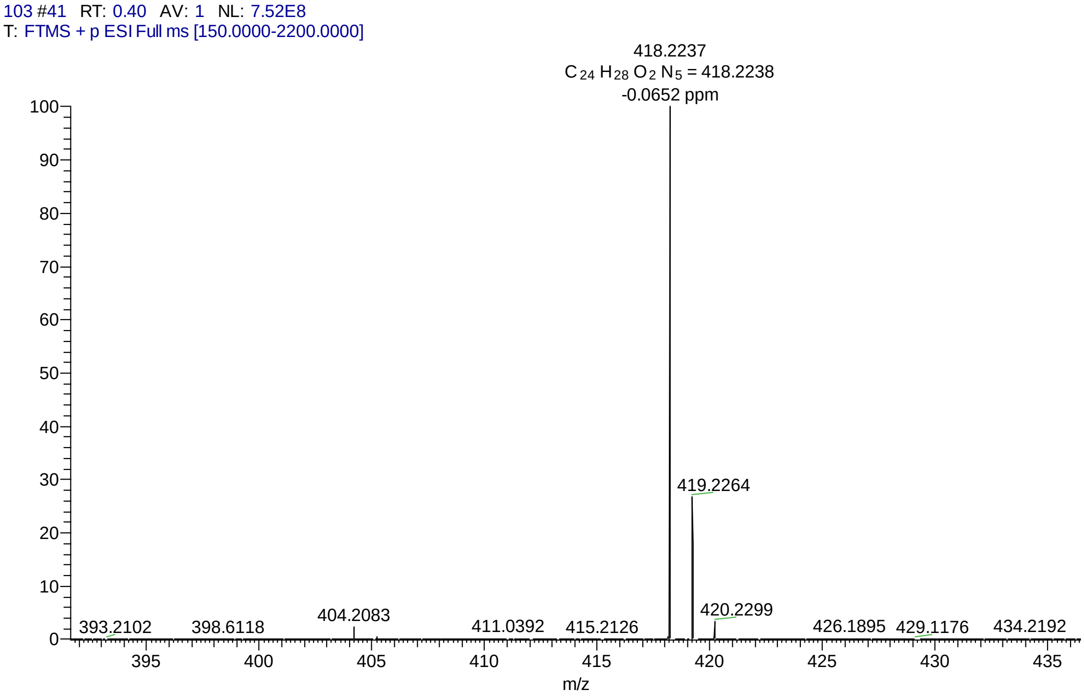

Figure S65. MS spectrum of **A26.**

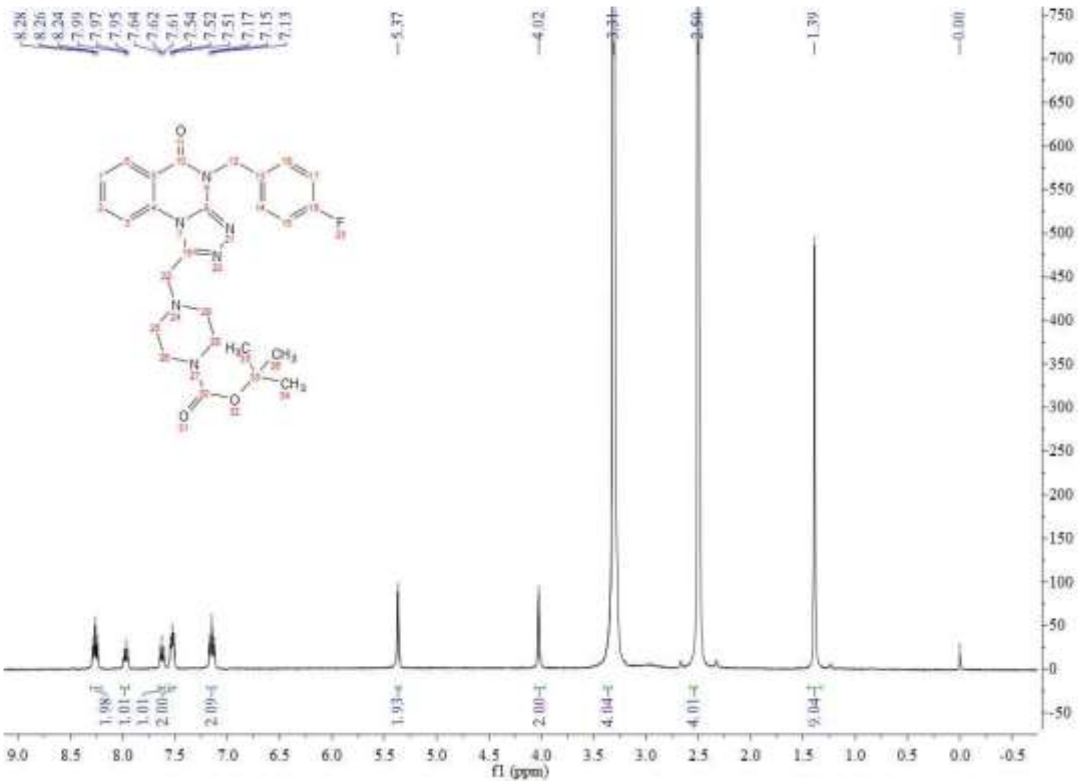

Figure S66. ^{1}H-NMR spectrum of **A27.**

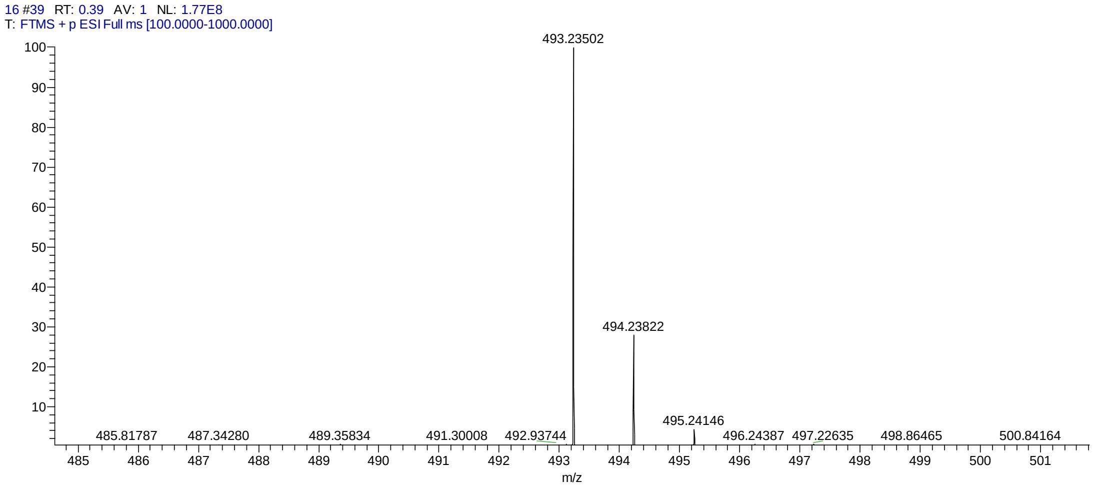

Figure S67. MS spectrum of **A27.**

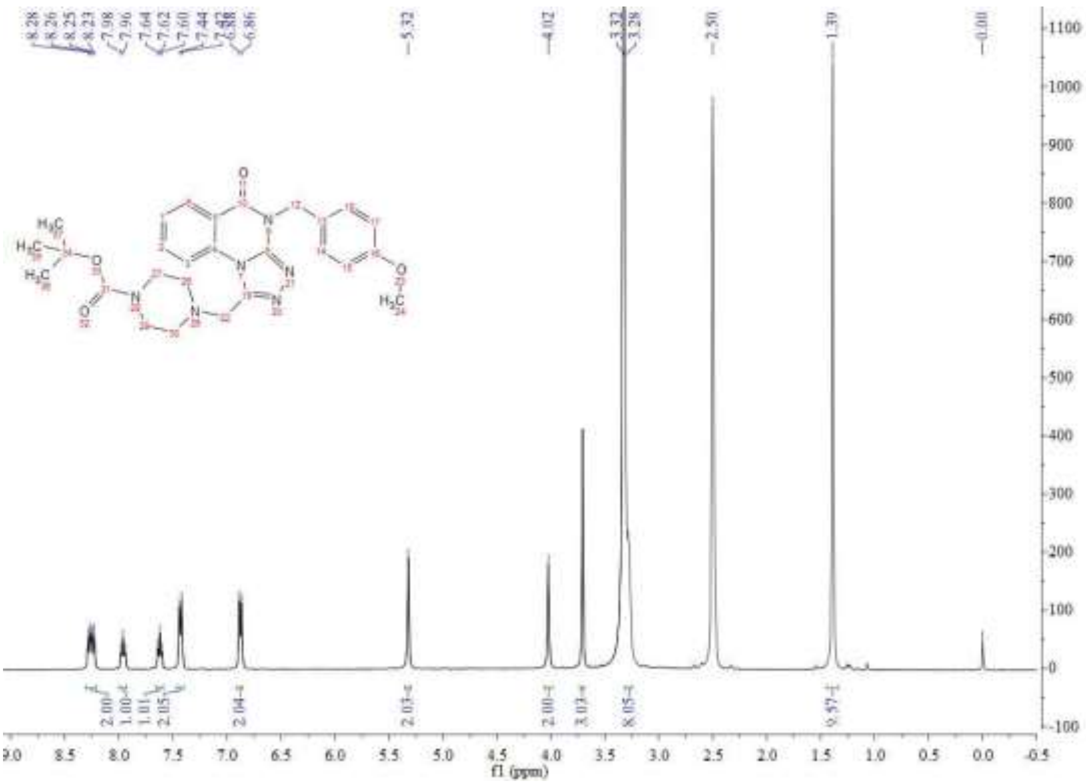

Figure S68. ^{1}H-NMR spectrum of **A28.**

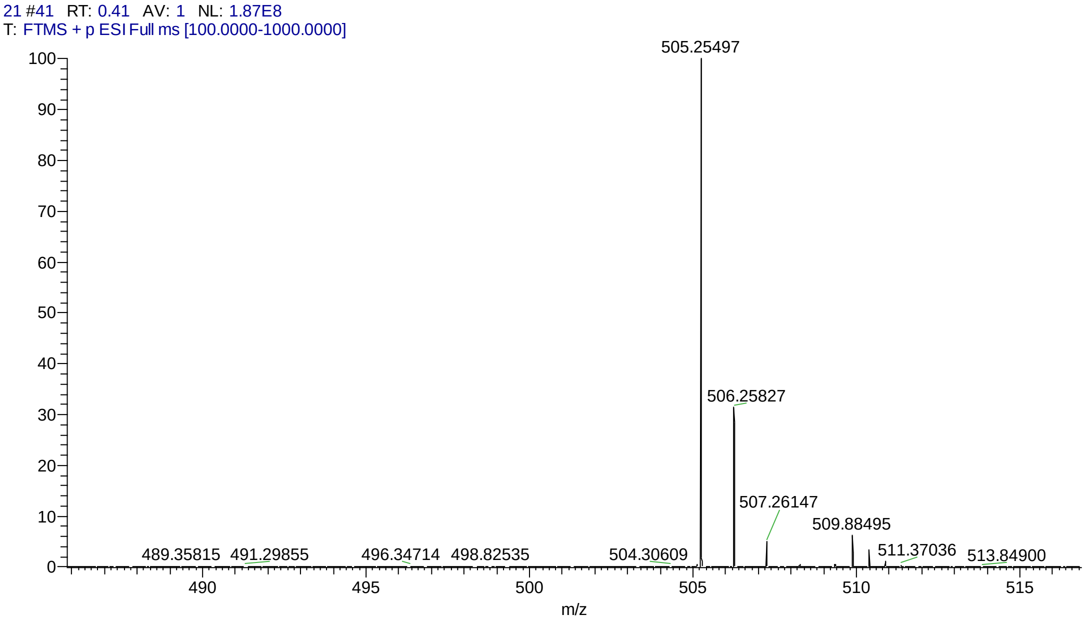

Figure S69. MS spectrum of **A28.**

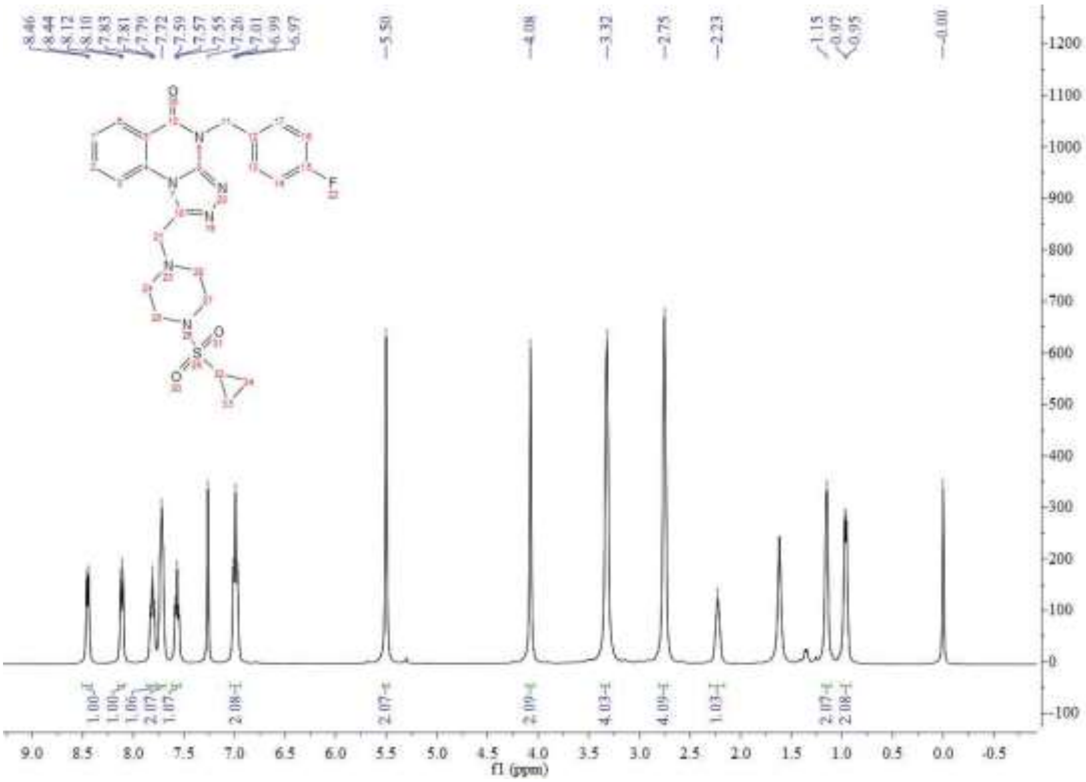

Figure S70. ^{1}H-NMR spectrum of **A29.**

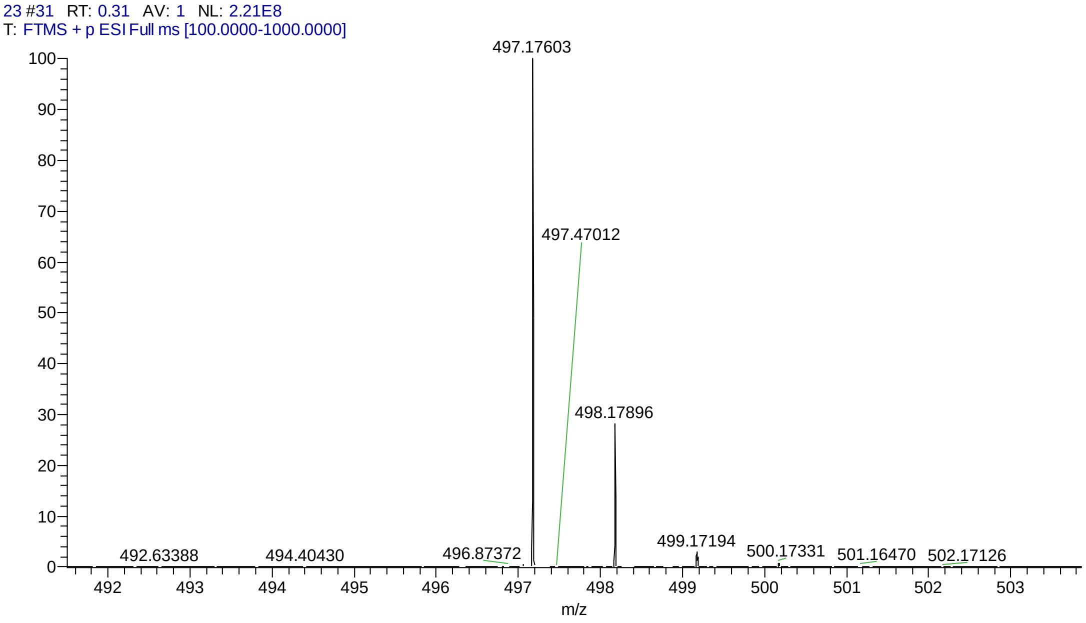

Figure S71. MS spectrum of **A29.**

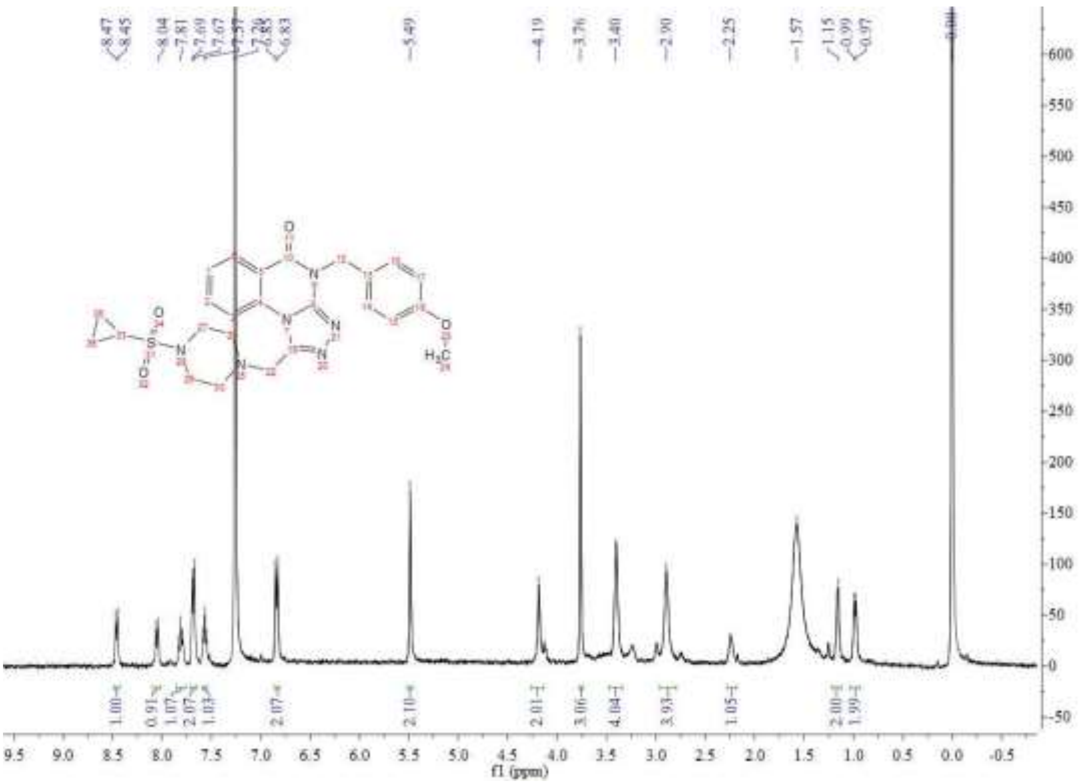

Figure S72. ^{1}H-NMR spectrum of **A30.**

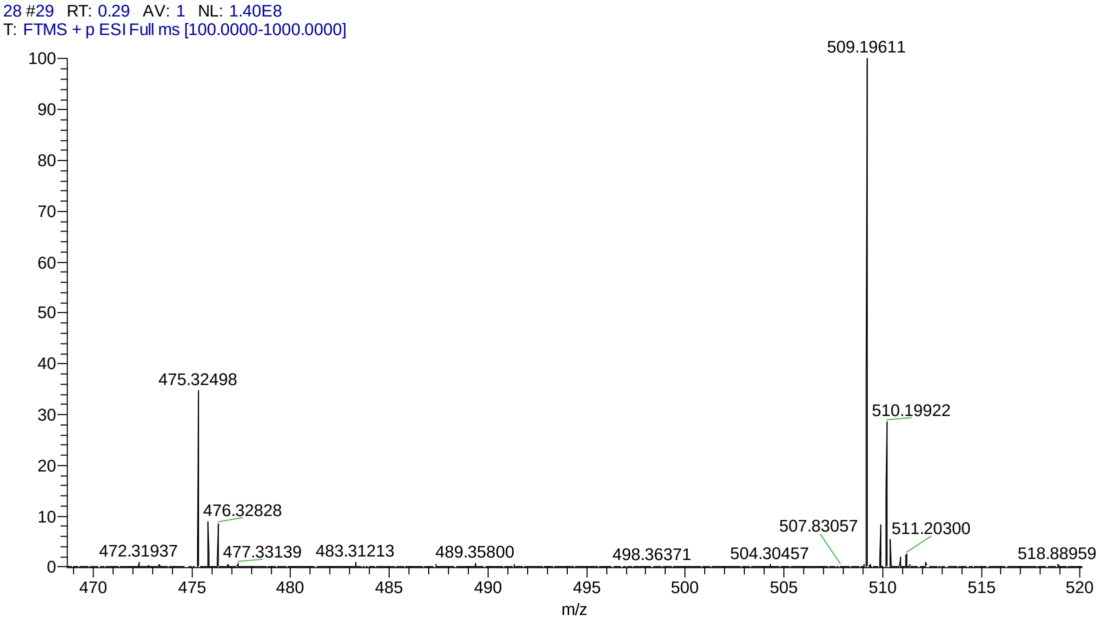

Figure S73. MS spectrum of **A30.**

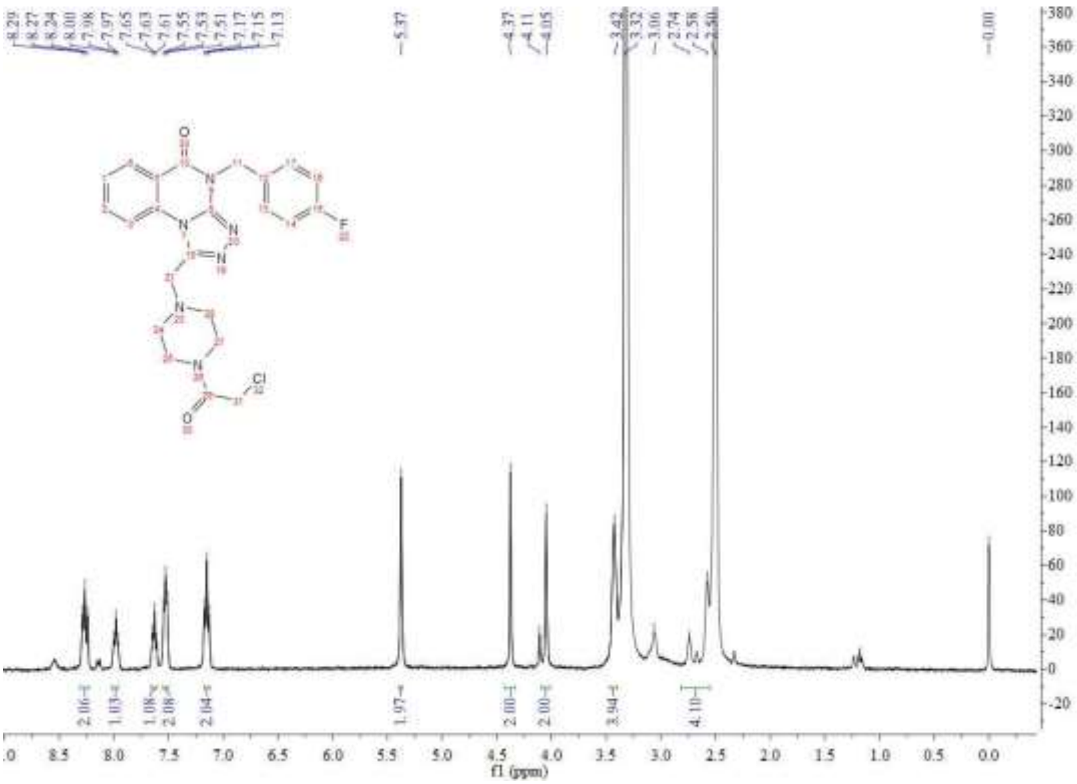

Figure S74. ^{1}H-NMR spectrum of **A31.**

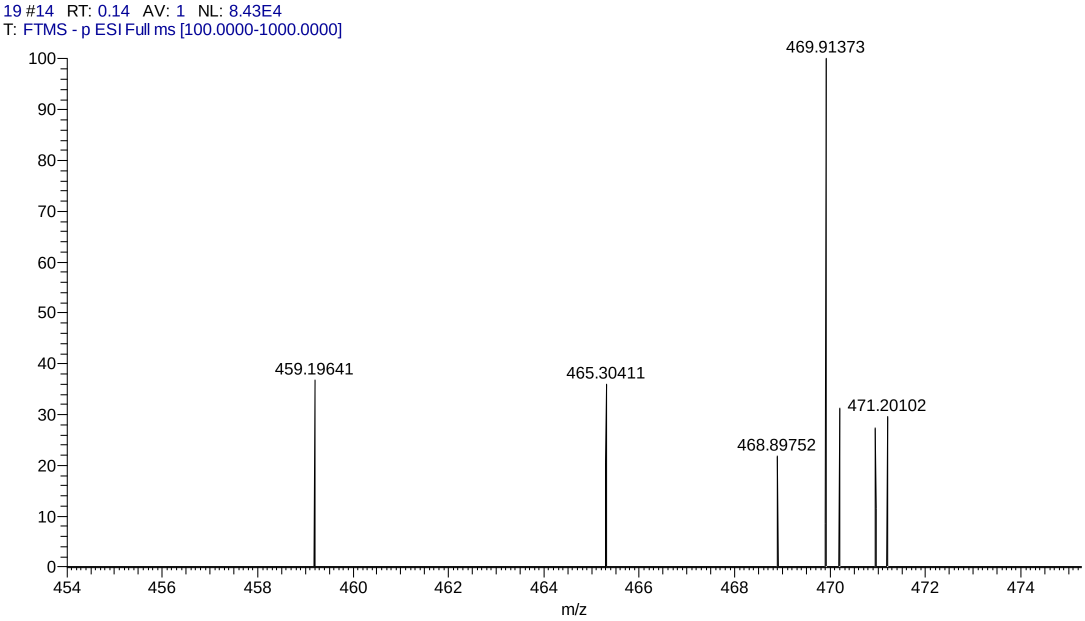

Figure S75. MS spectrum of **A31.**

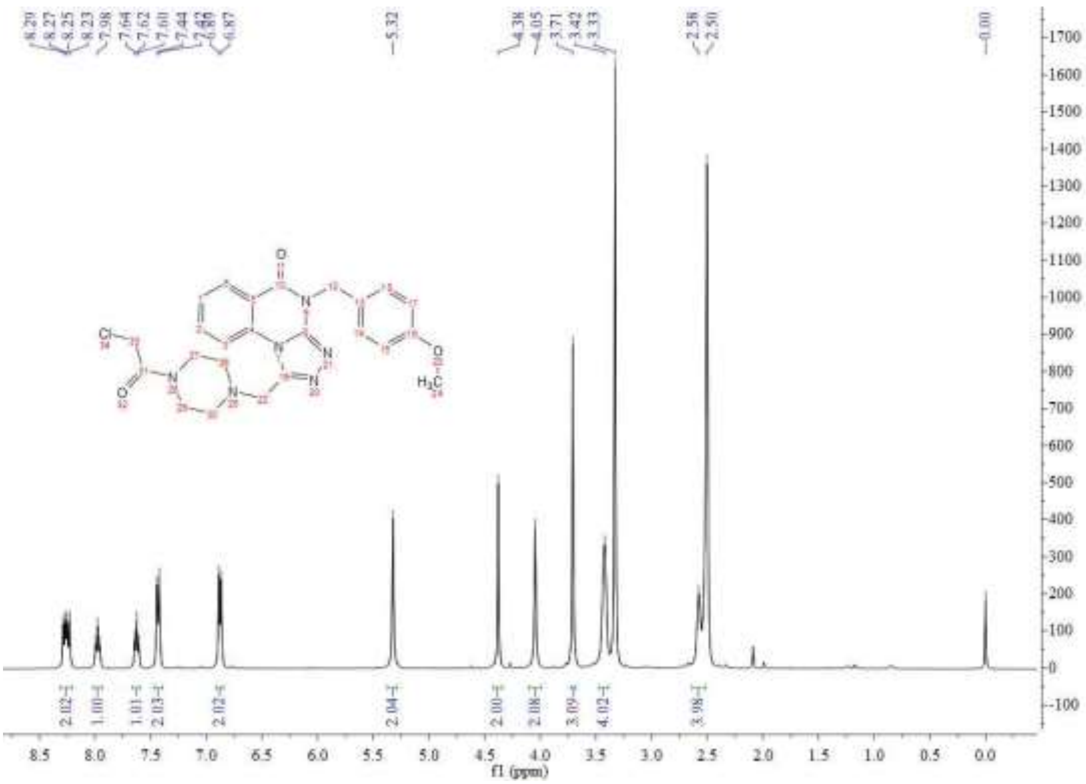

Figure S76. ^{1}H-NMR spectrum of **A32.**

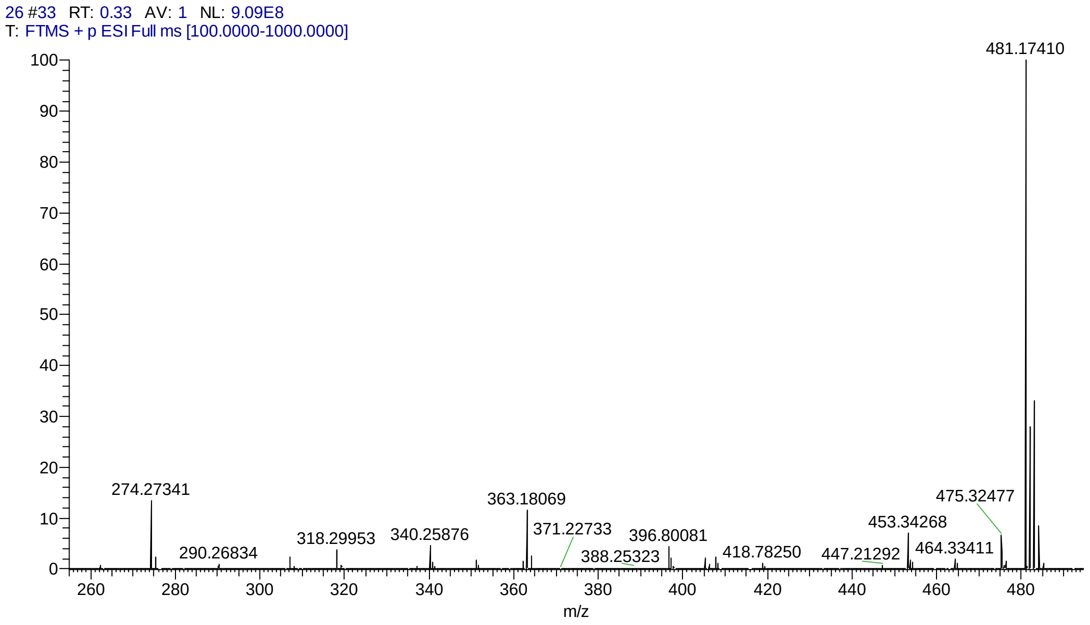

Figure S77. MS spectrum of **A32.**

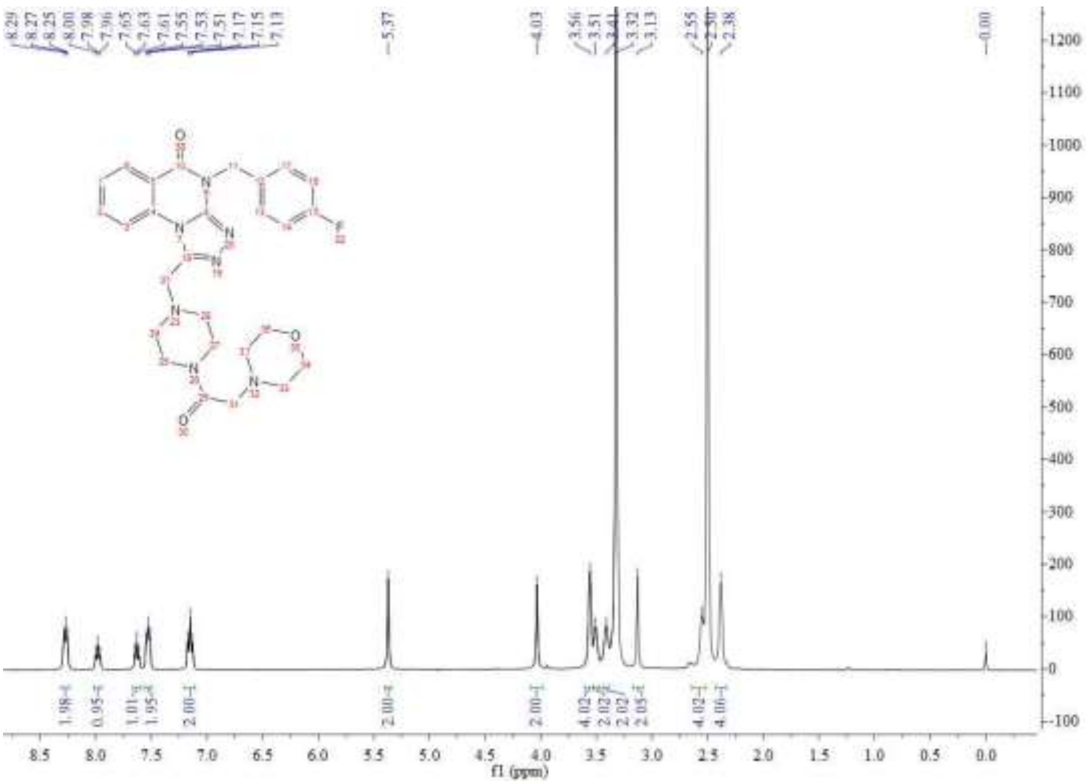

Figure S78. ^{1}H-NMR spectrum of **A33.**

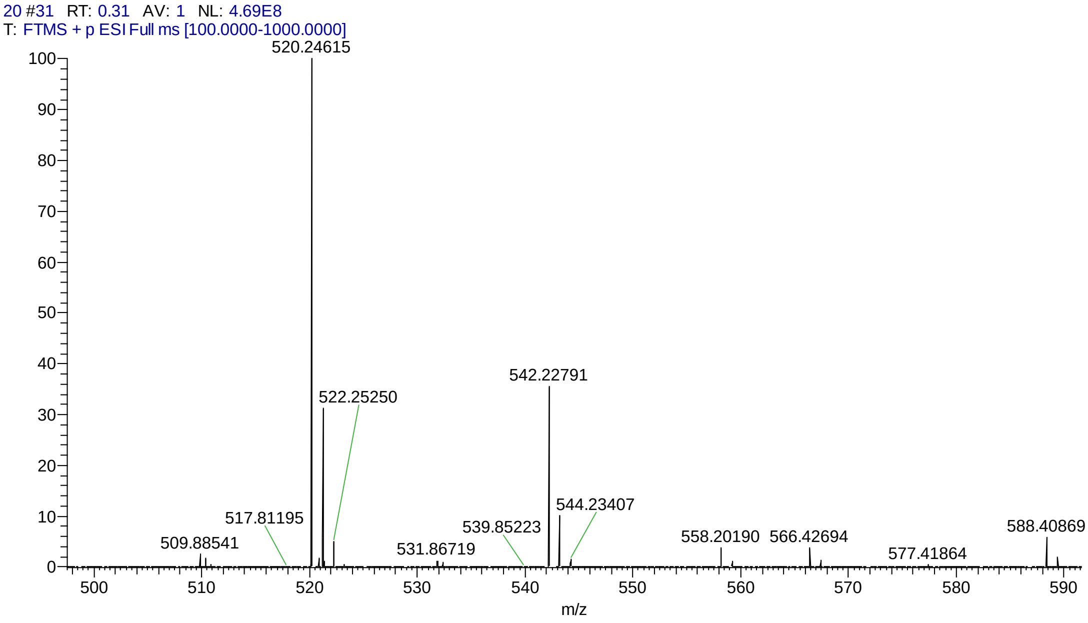

Figure S79. MS spectrum of **A33.**

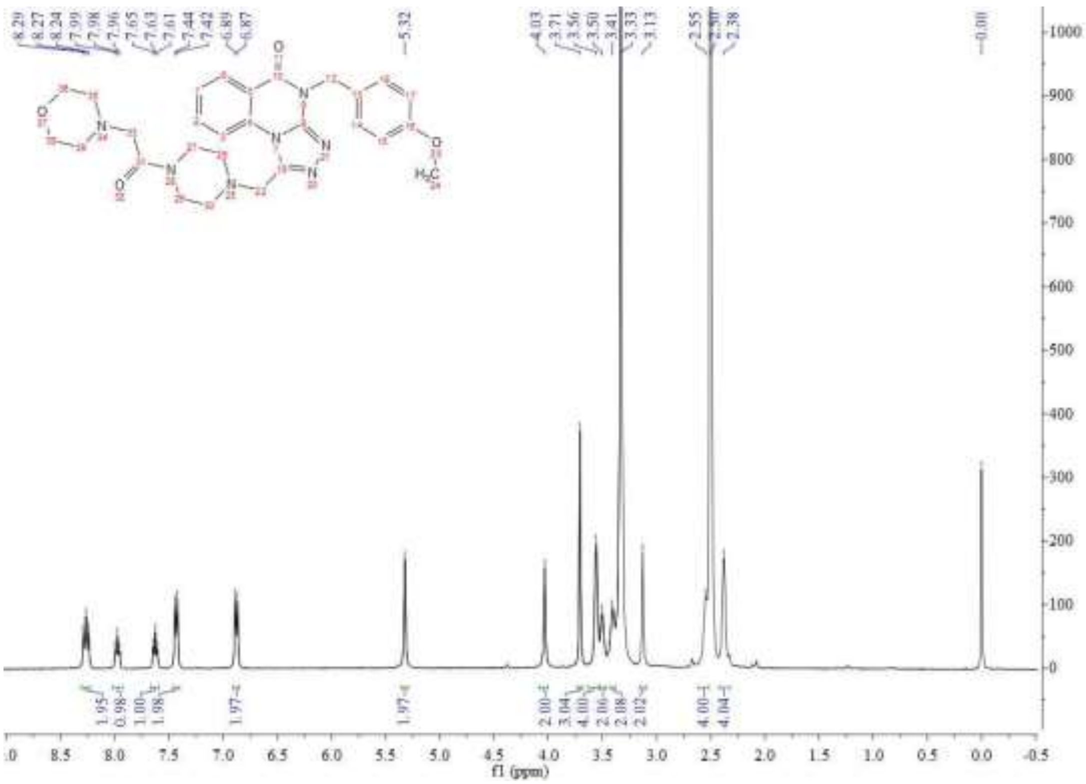

Figure S80. ^{1}H-NMR spectrum of **A34.**

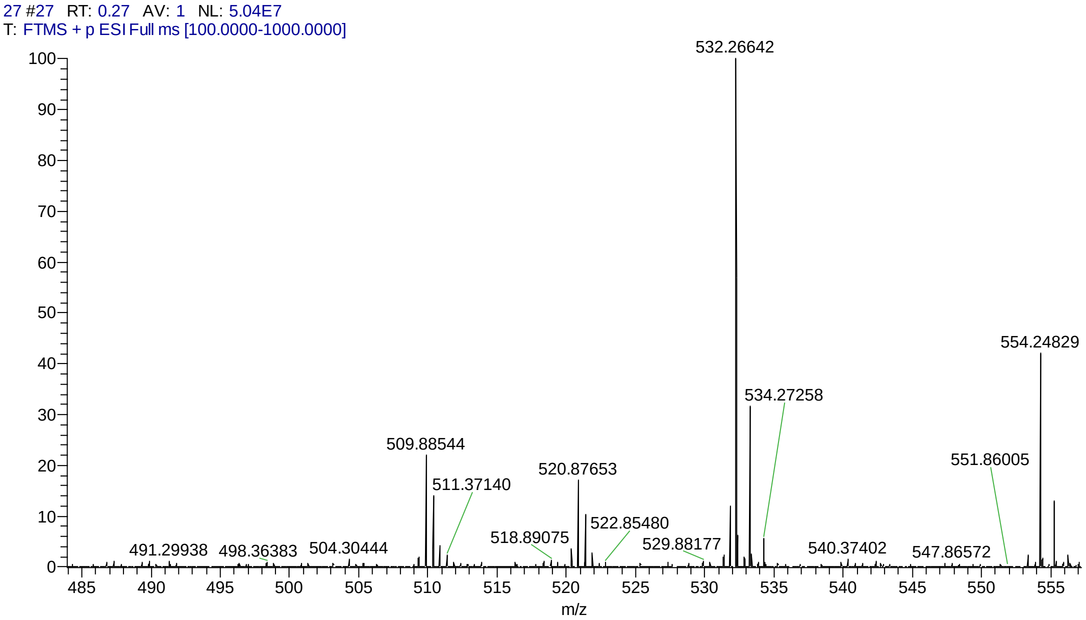

Figure S81. MS spectrum of **A34.**

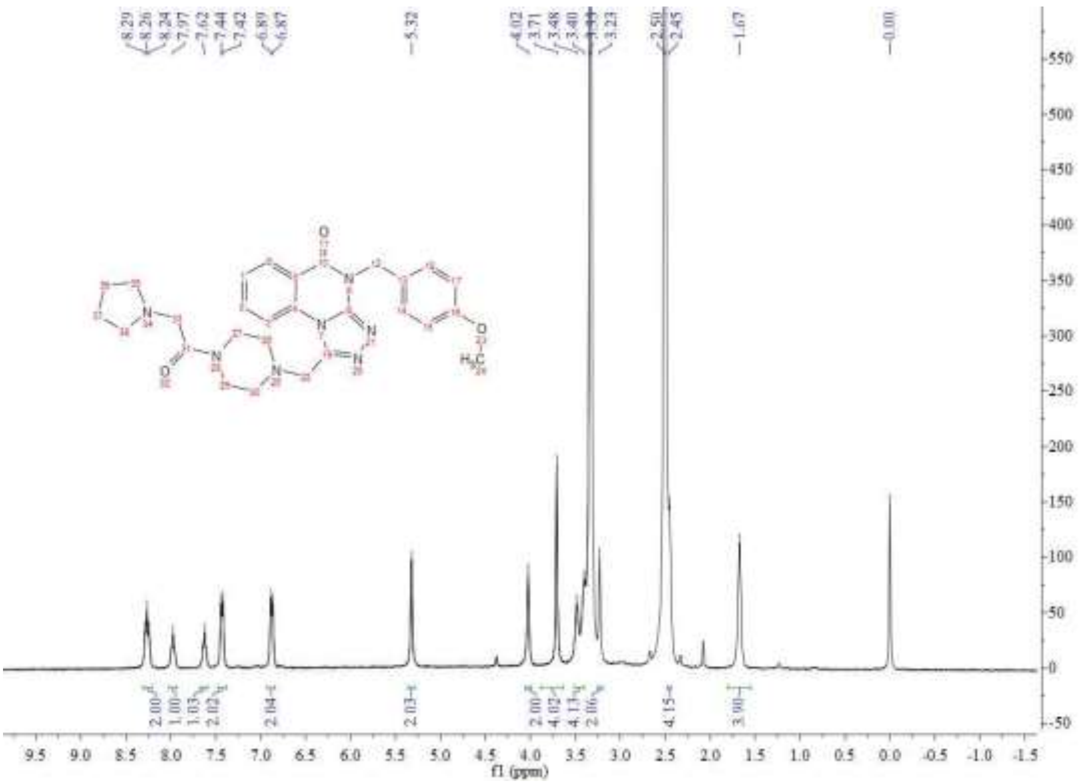

Figure S82. ^{1}H-NMR spectrum of **A35.**

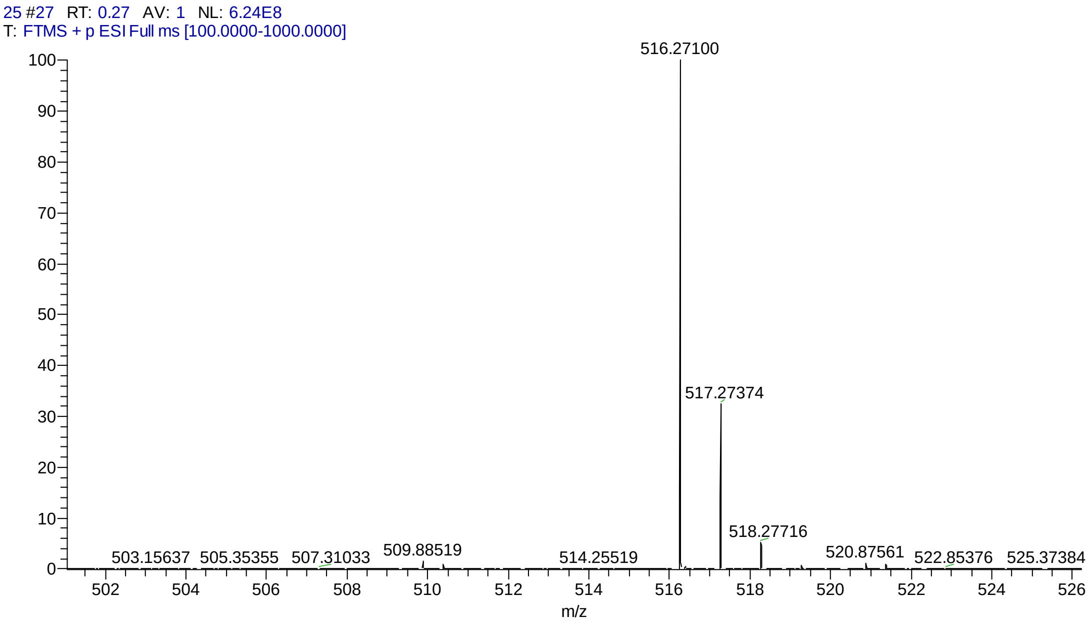

Figure S83. MS spectrum of **A35.**

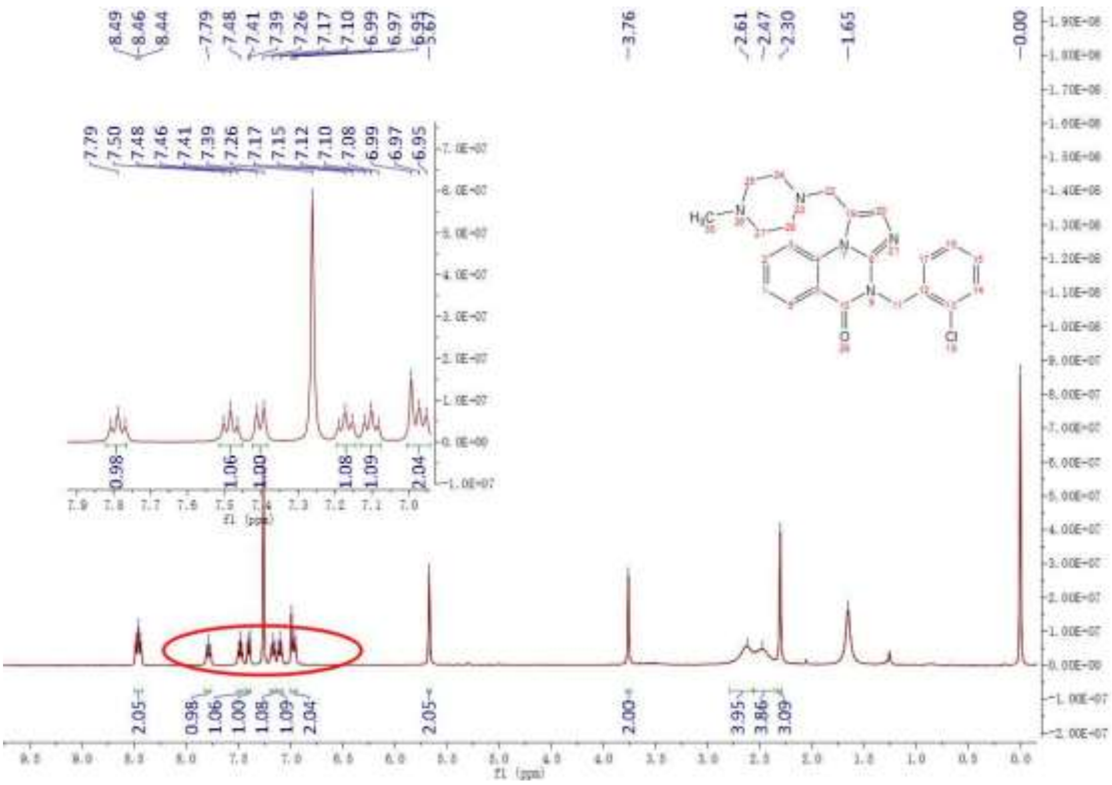

Figure S84. ^{1}H-NMR spectrum of **B1.**

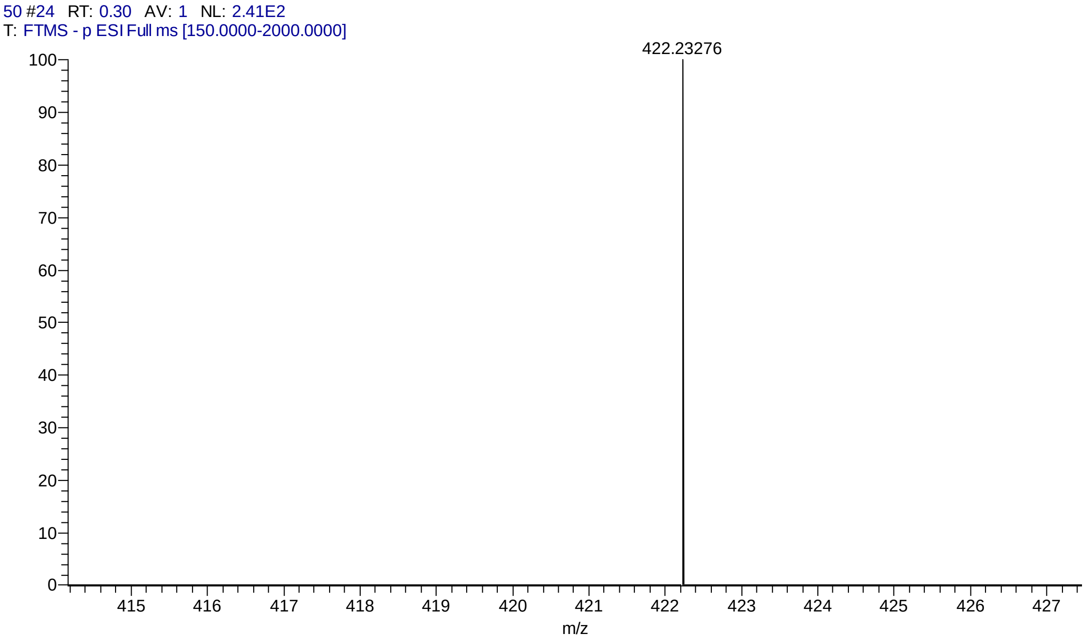

Figure S85. MS spectrum of **B1.**

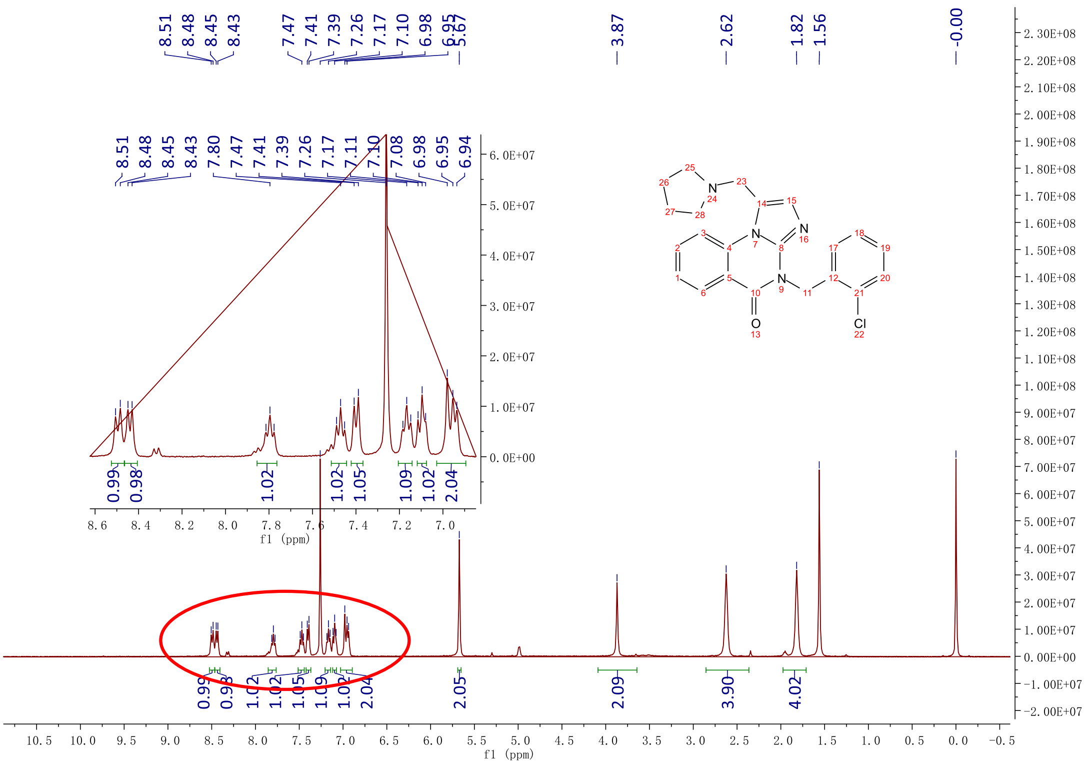

Figure S86. ^{1}H-NMR spectrum of **B2.**

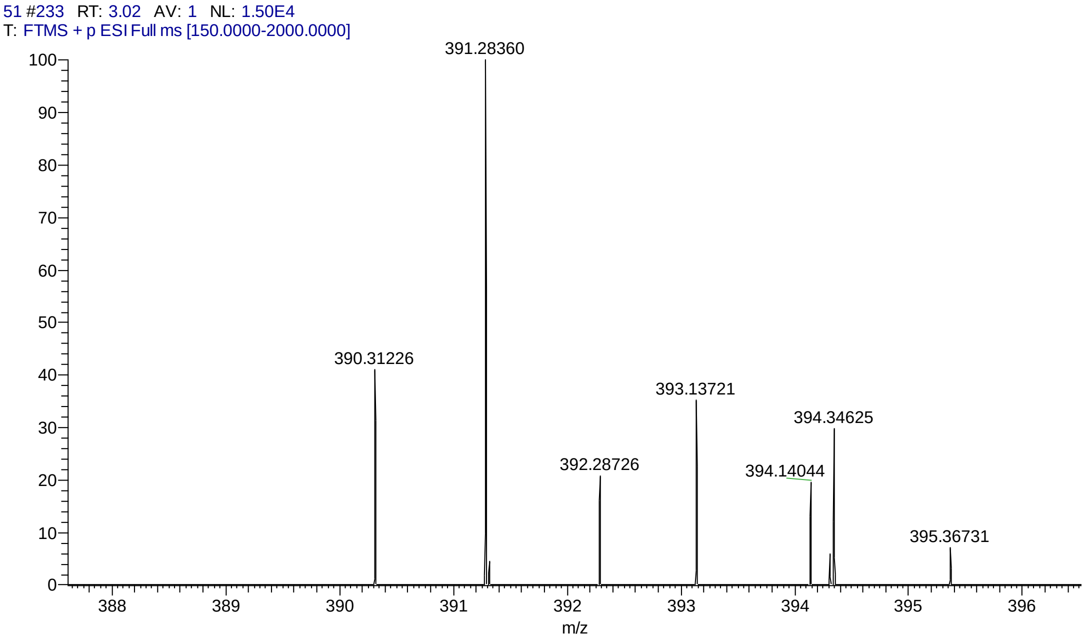

Figure S87. MS spectrum of **B2.**

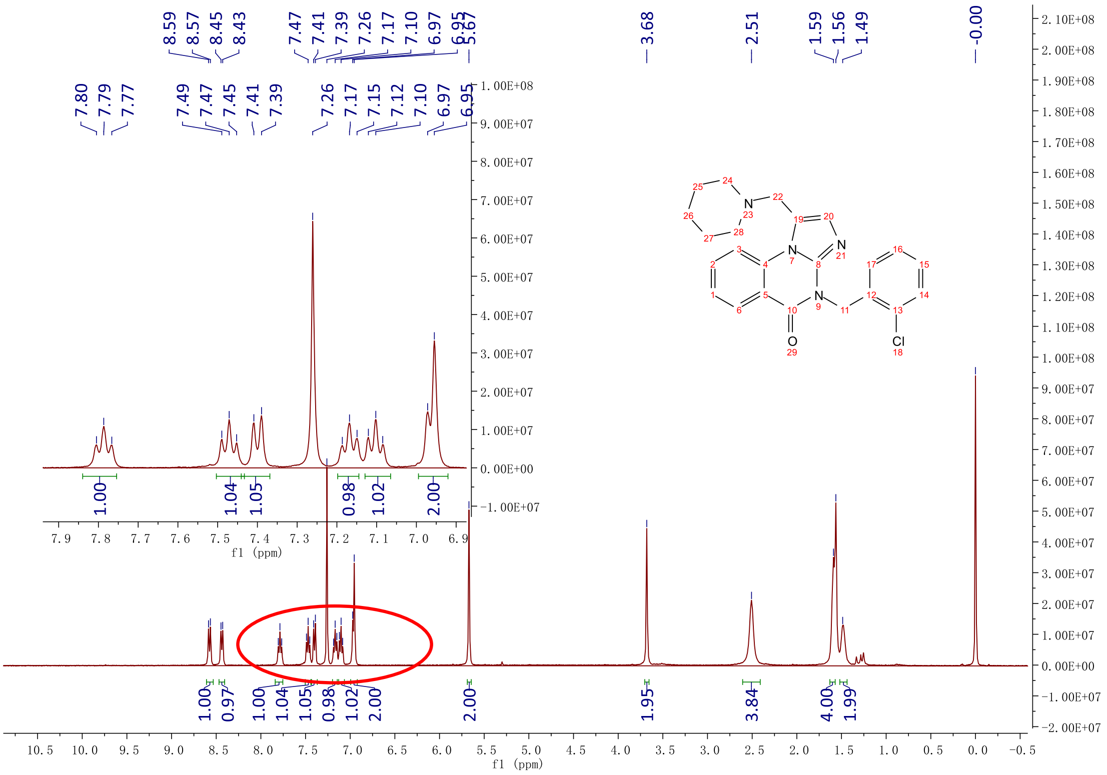

Figure S88. ^{1}H-NMR spectrum of **B3.**

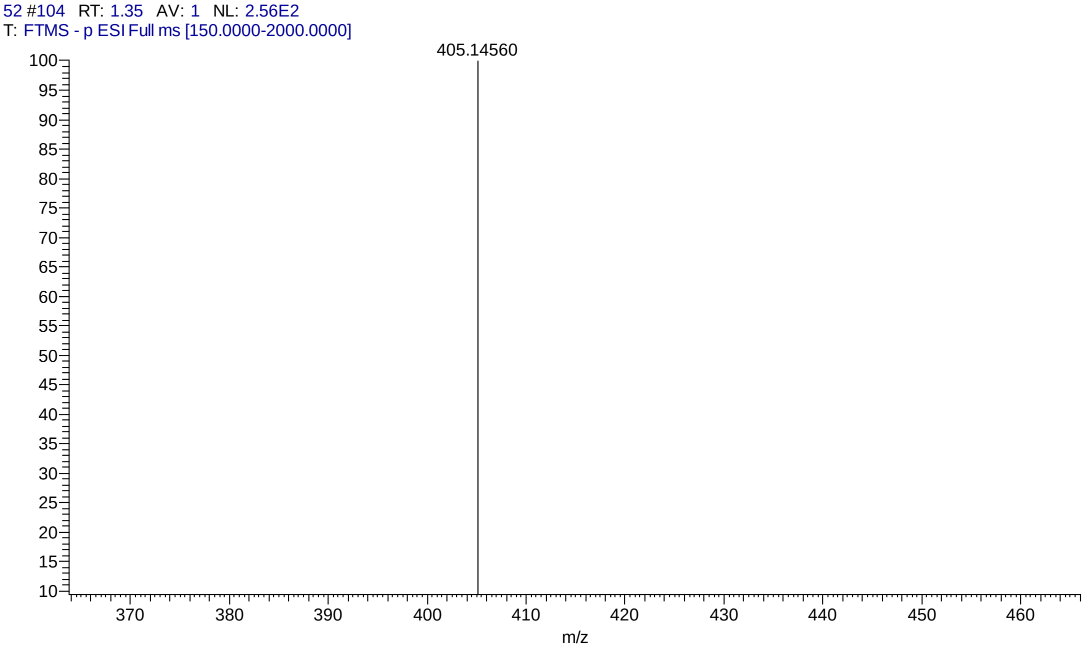

Figure S89. MS spectrum of **B3.**

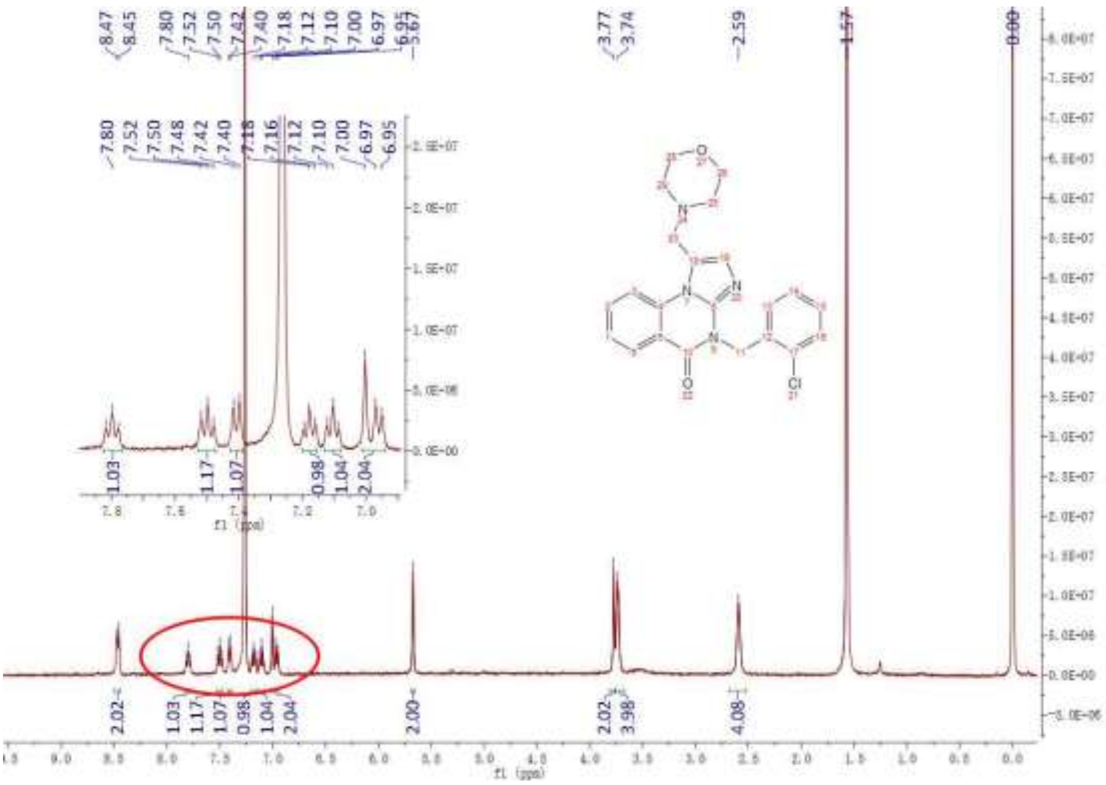

Figure S90. ^{1}H-NMR spectrum of **B4.**

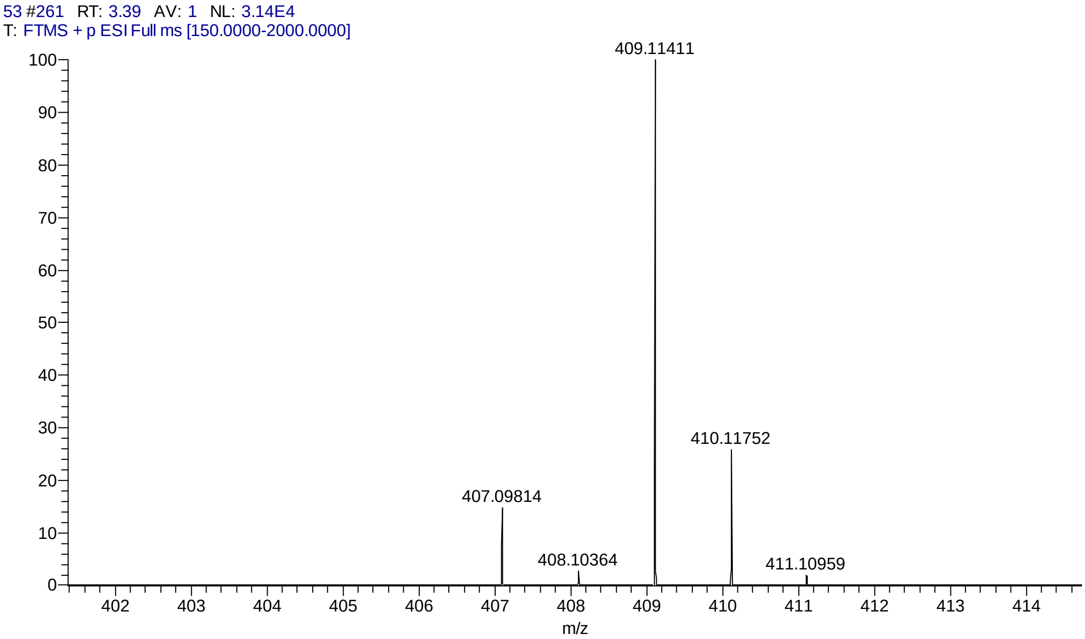

Figure S91. MS spectrum of **B4.**

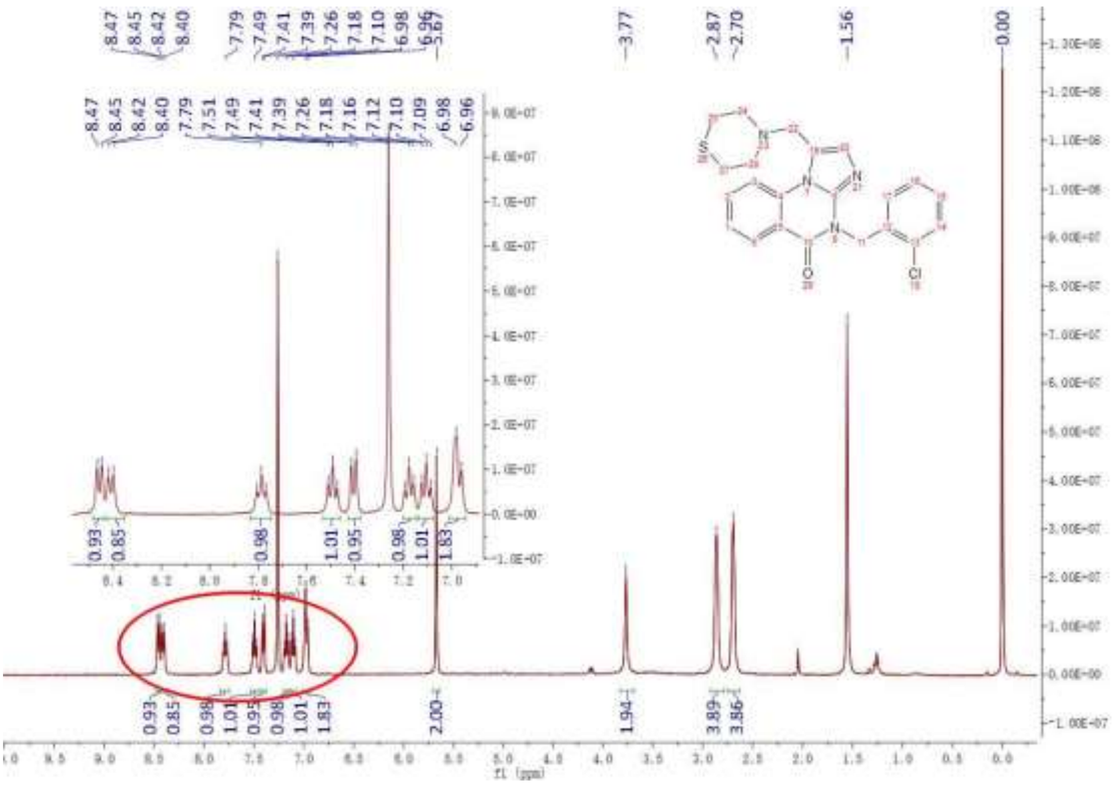

Figure S92. ^{1}H-NMR spectrum of **B5.**

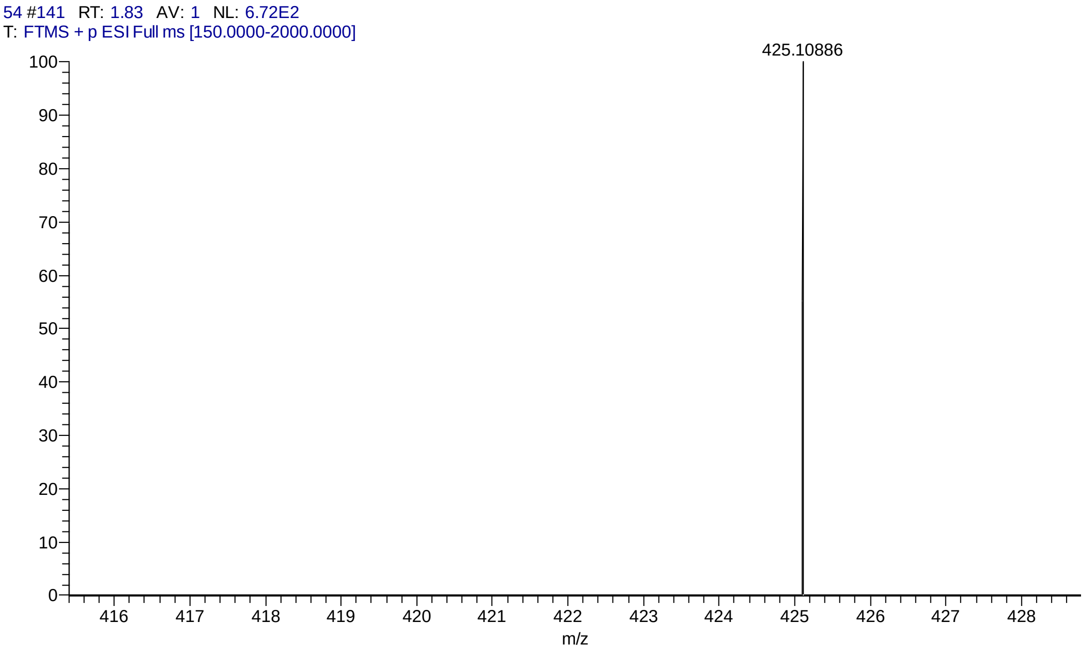

Figure S93. MS spectrum of **B5.**

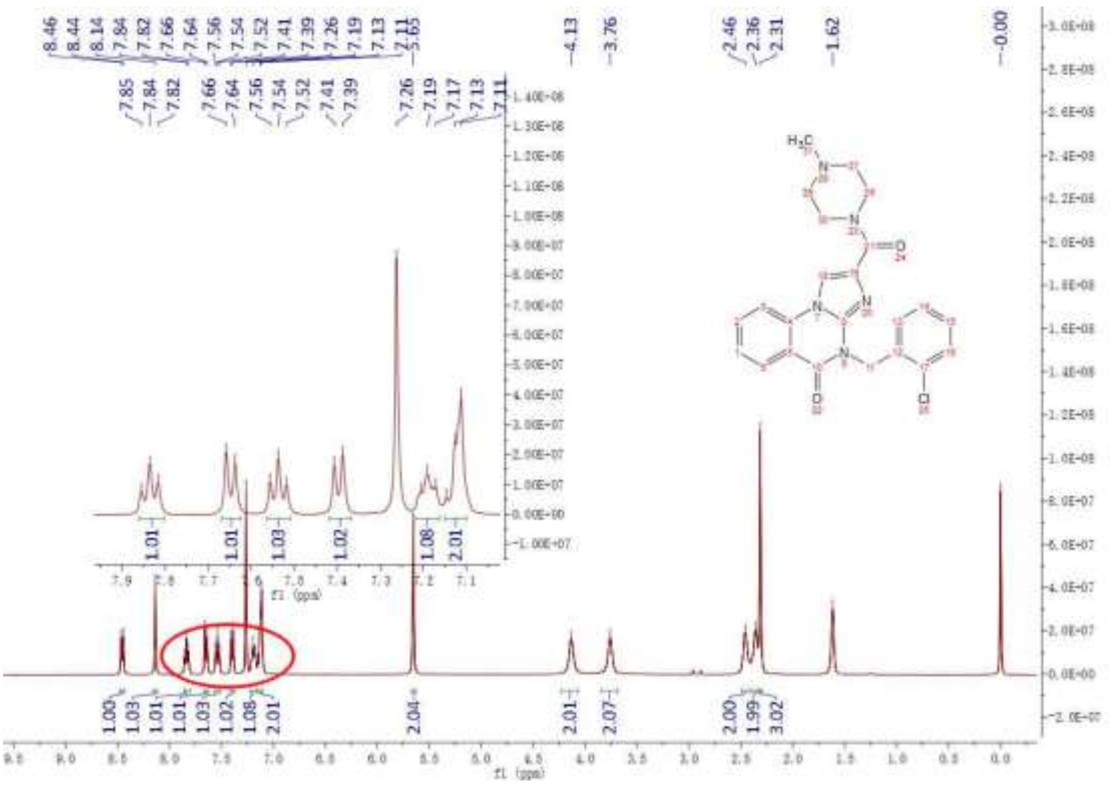

Figure S94. ¹H-NMR spectrum of **B6.**

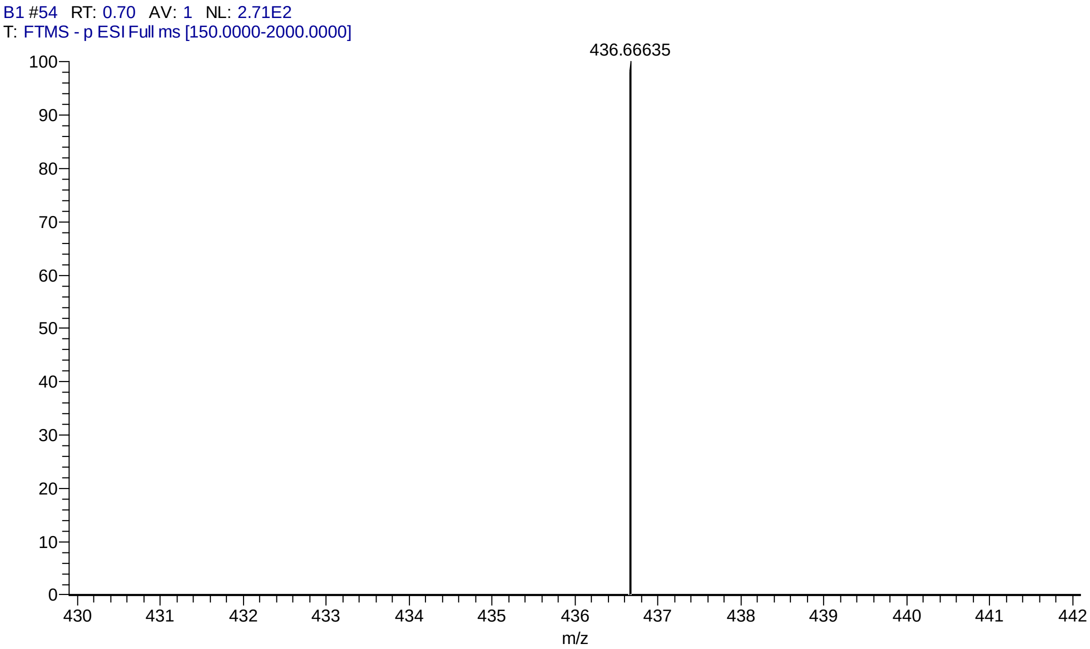

Figure S95. MS spectrum of **B6.**

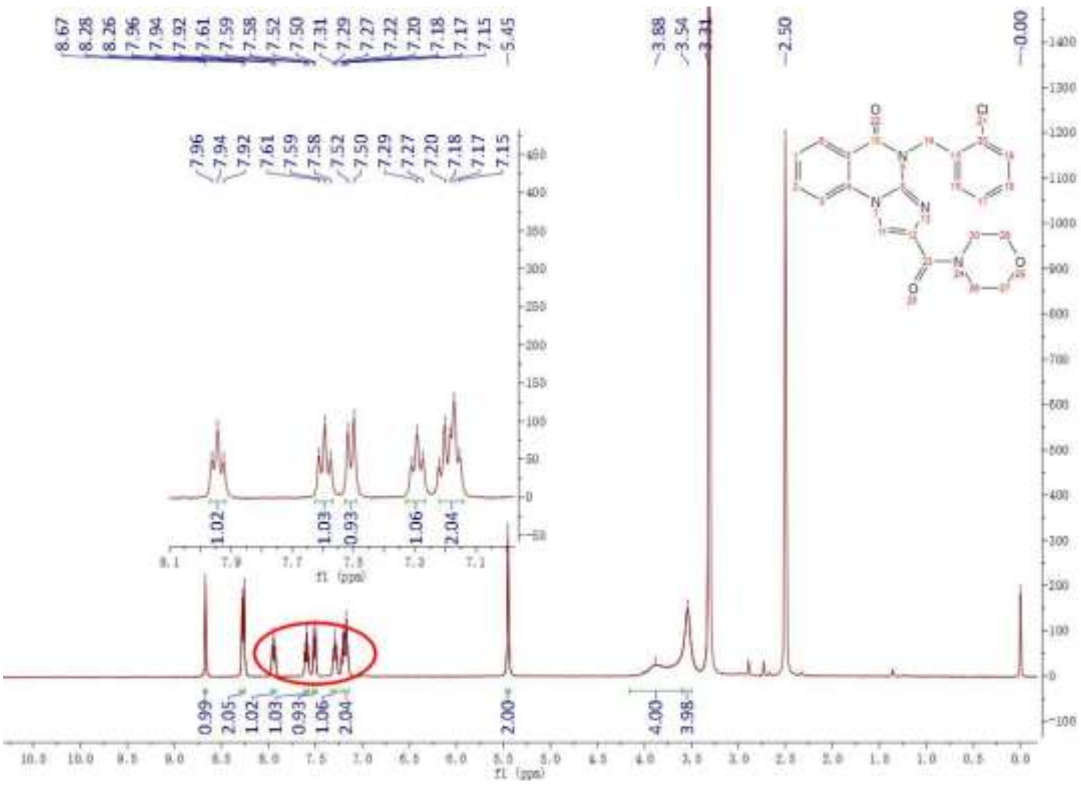

Figure S96. ^{1}H-NMR spectrum of **B7.**

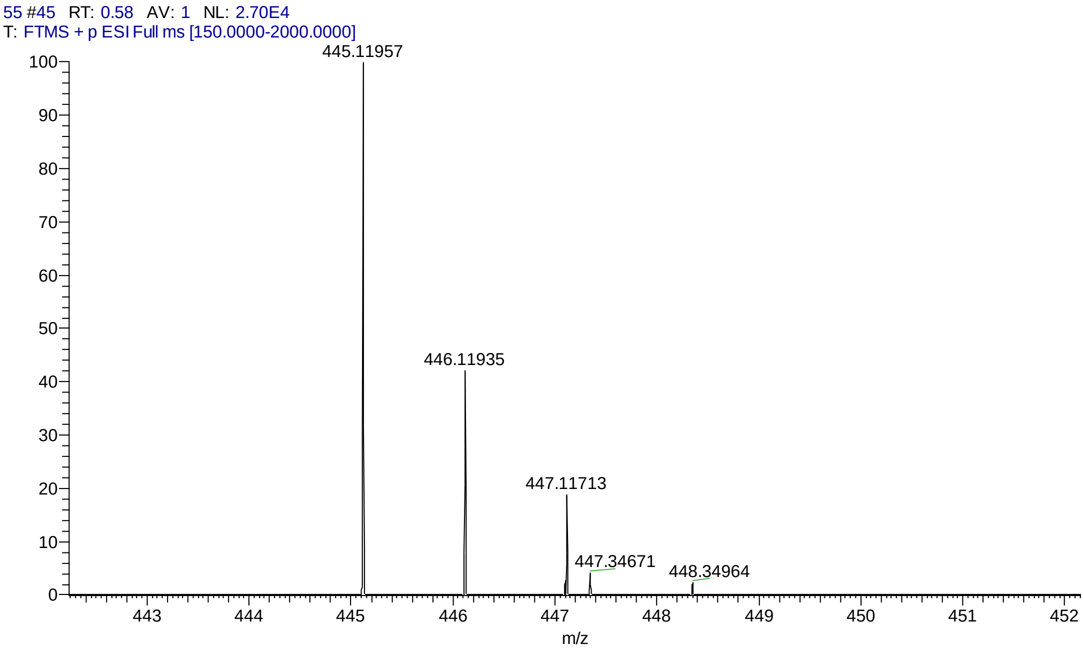

Figure S97. MS spectrum of **B7.**

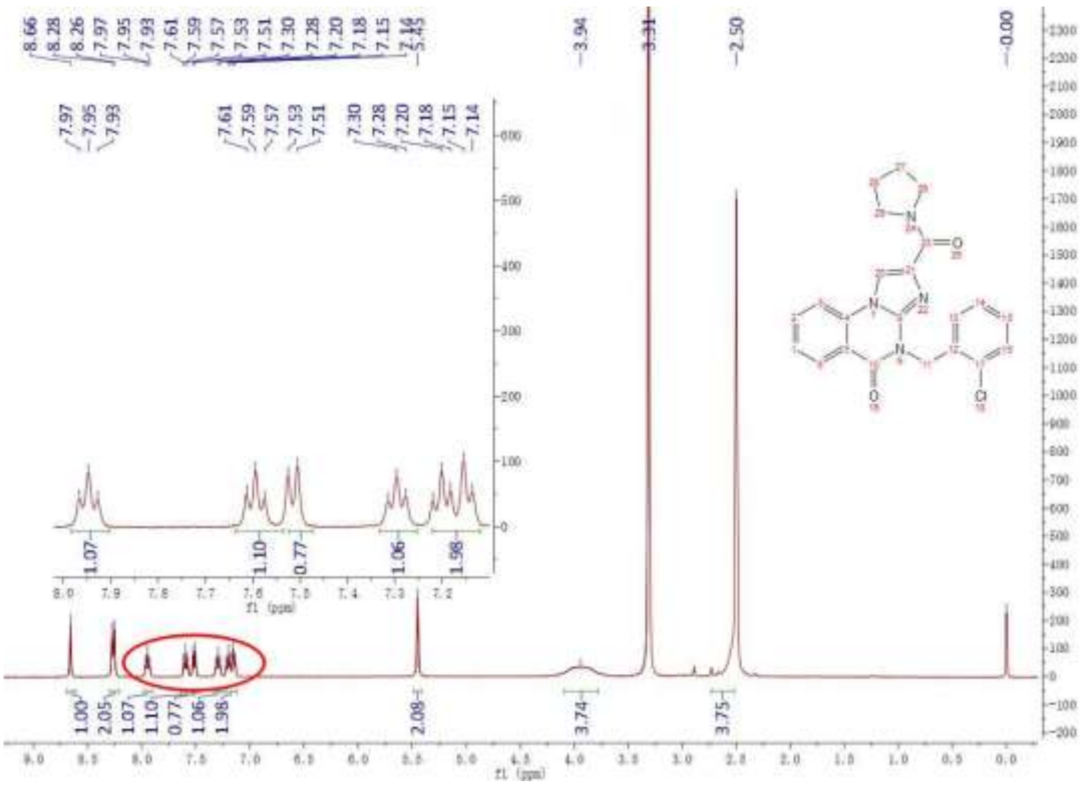

Figure S98. ^{1}H-NMR spectrum of **B8.**

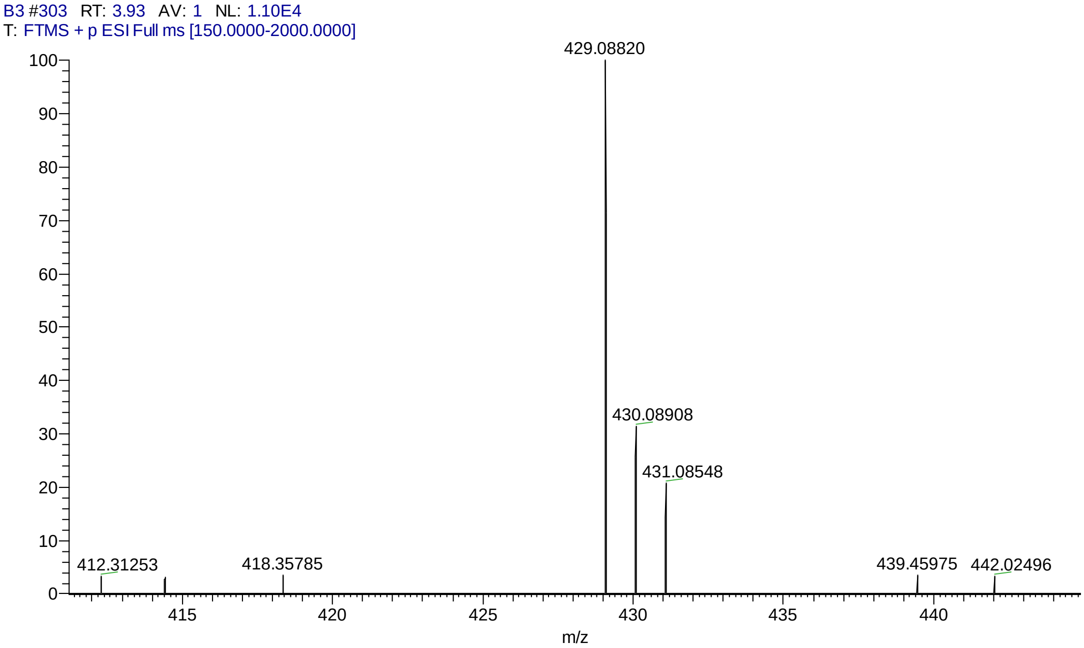

Figure S99. MS spectrum of **B8.**

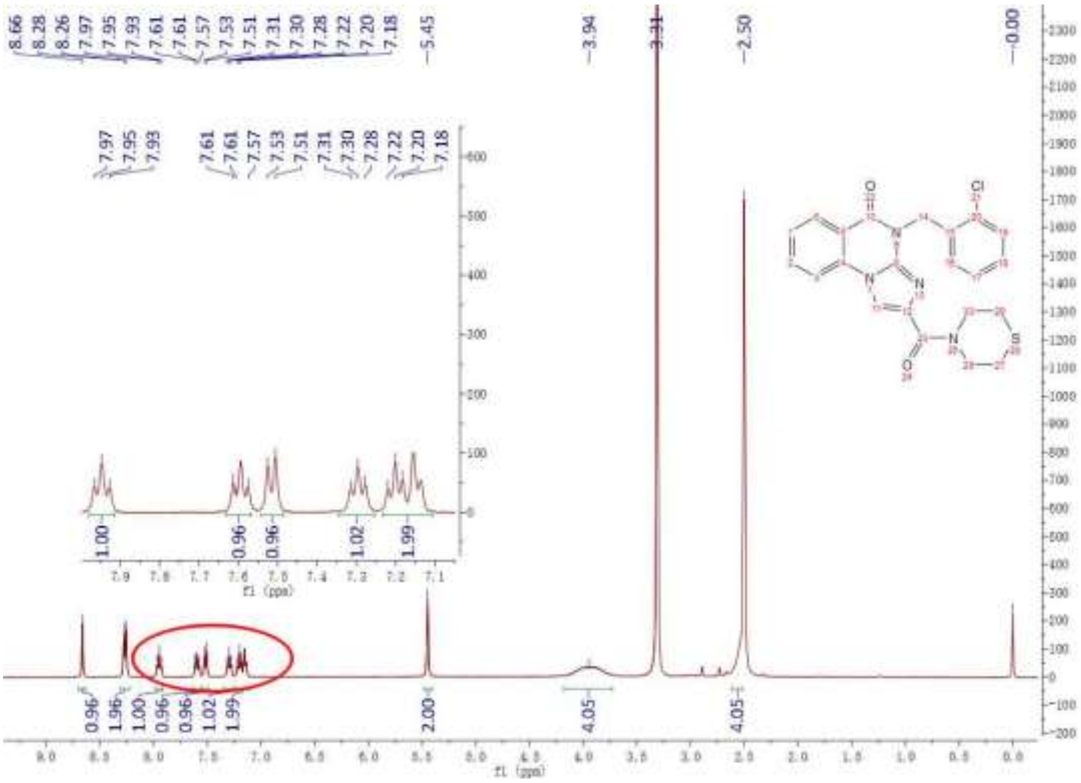

Figure S100. ^{1}H-NMR spectrum of **B9.**

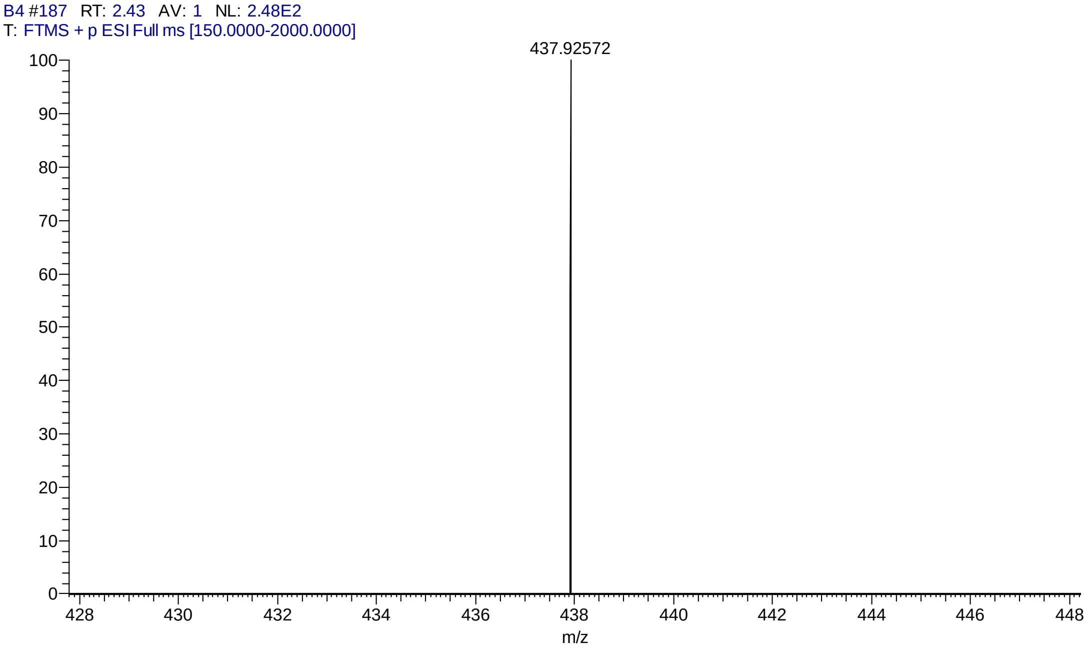

Figure S101. MS spectrum of **B9.**

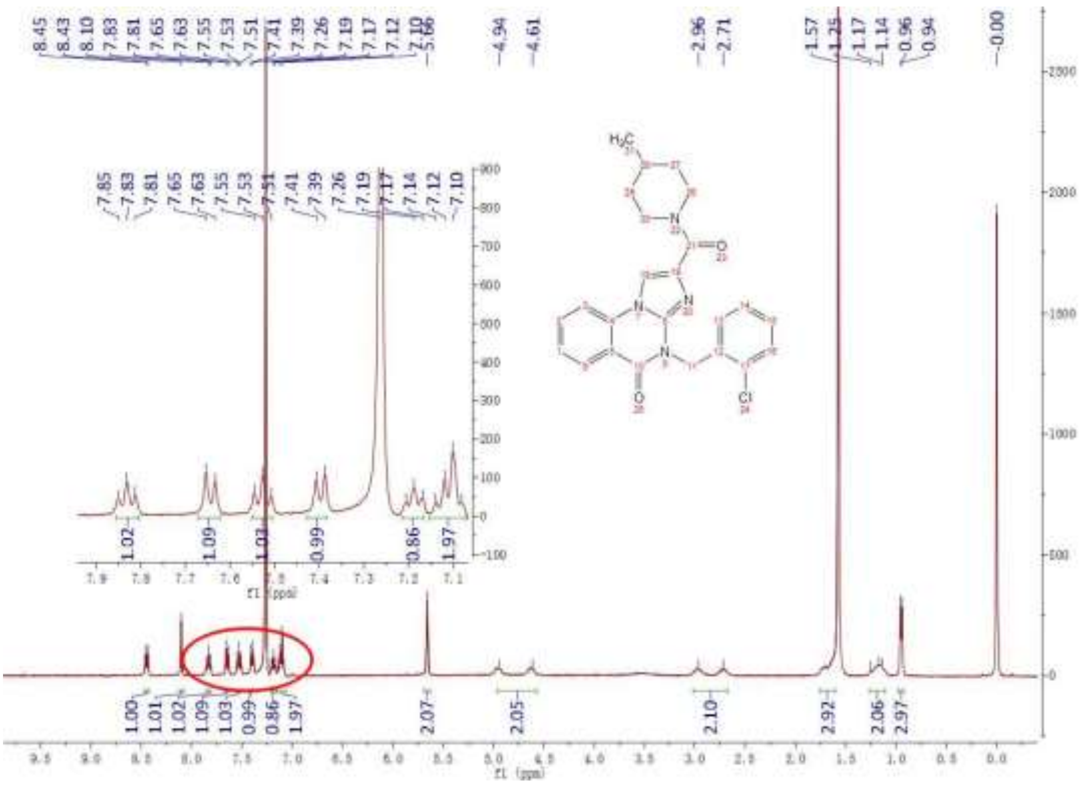

Figure S102. ^{1}H-NMR spectrum of **B10.**

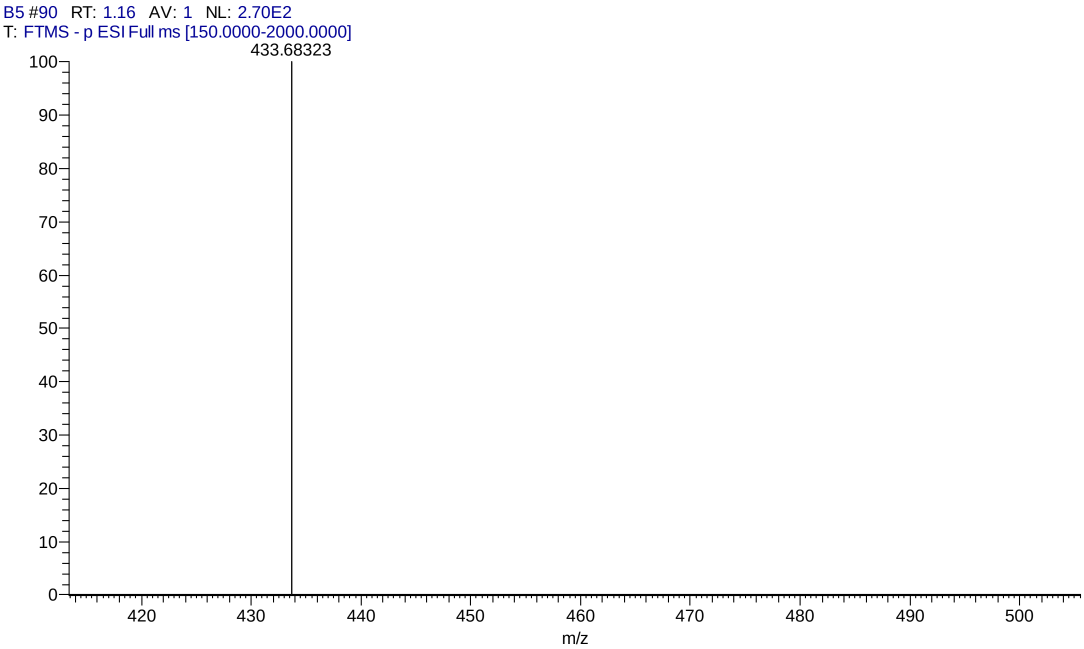

Figure S103. MS spectrum of **B10.**

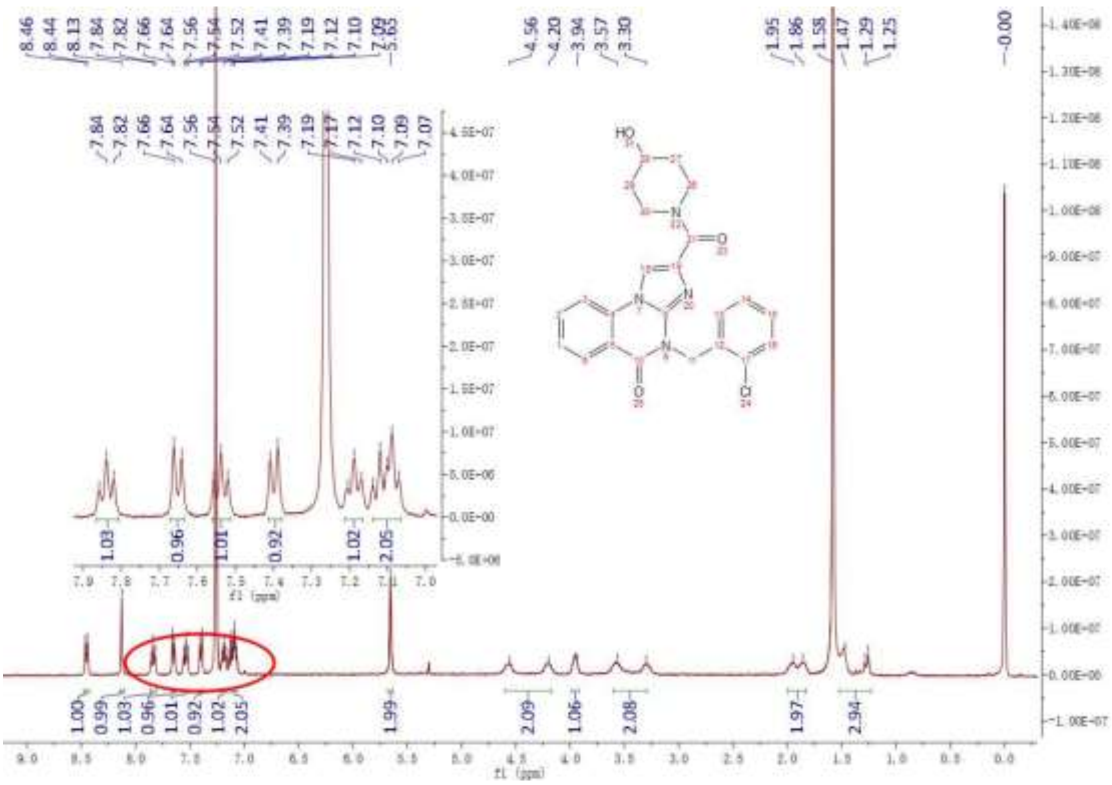

Figure S104. ^{1}H-NMR spectrum of **B11.**

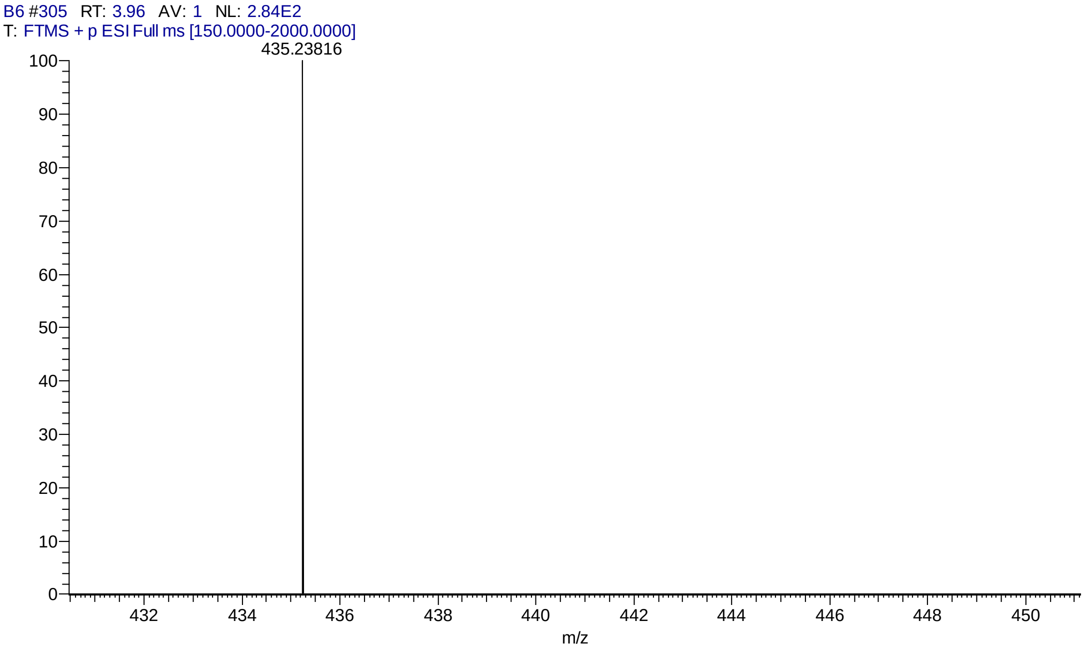

Figure S105. MS spectrum of **B11.**

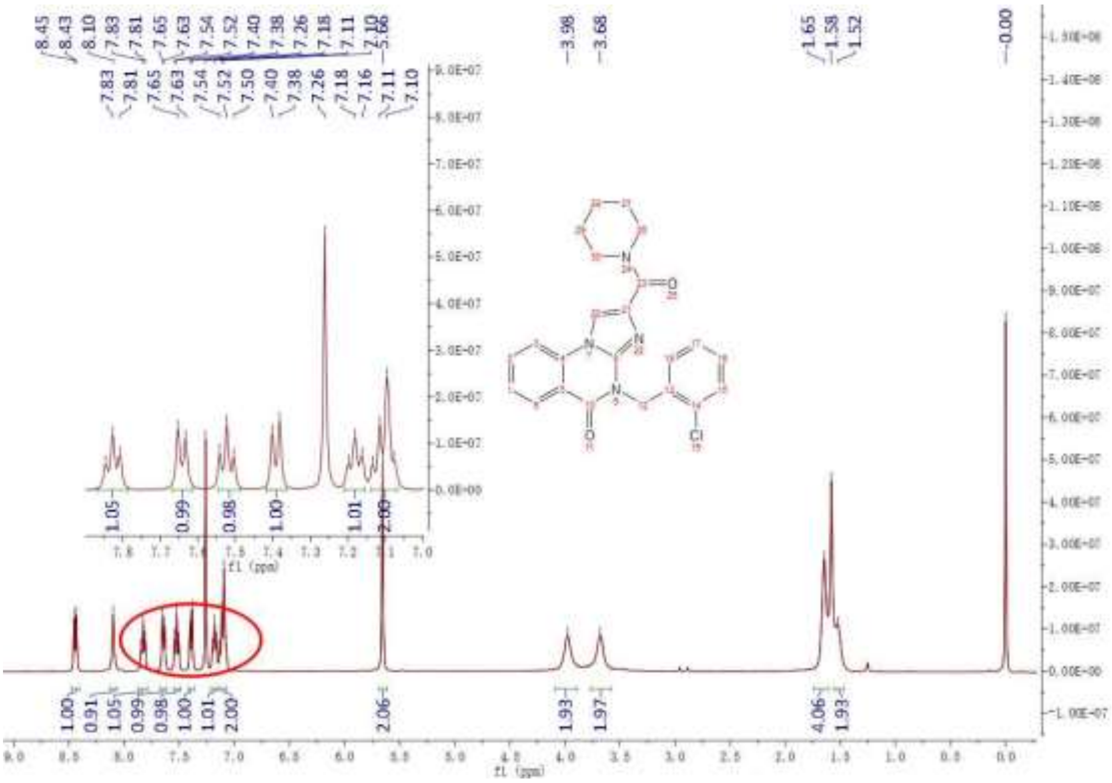

Figure S106. ^{1}H-NMR spectrum of **B12.**

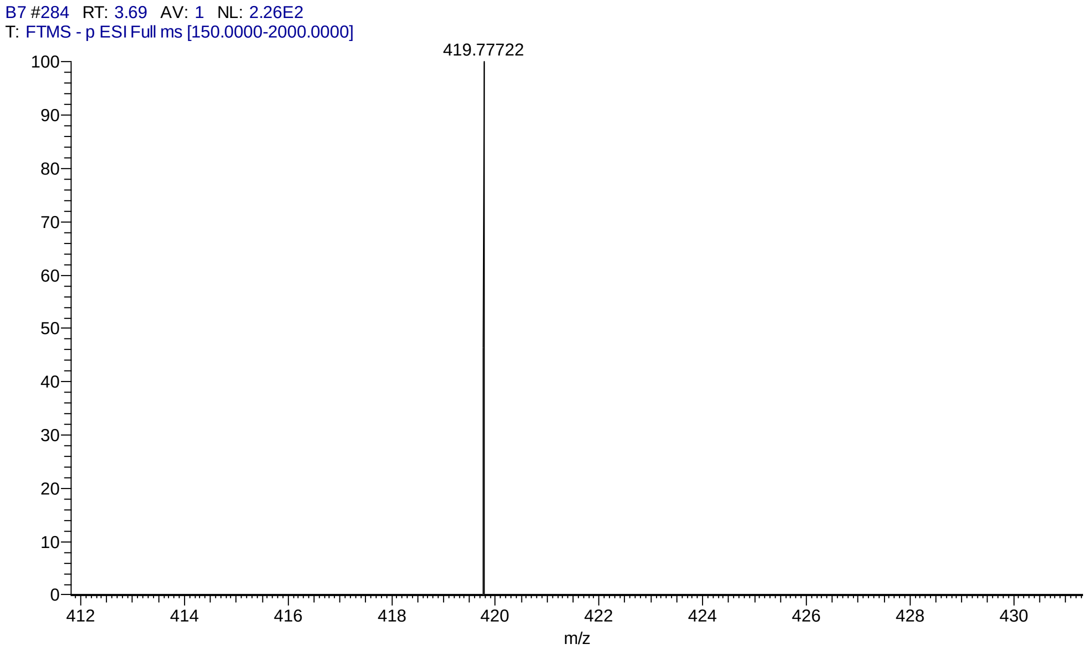

Figure S107. MS spectrum of **B12.**

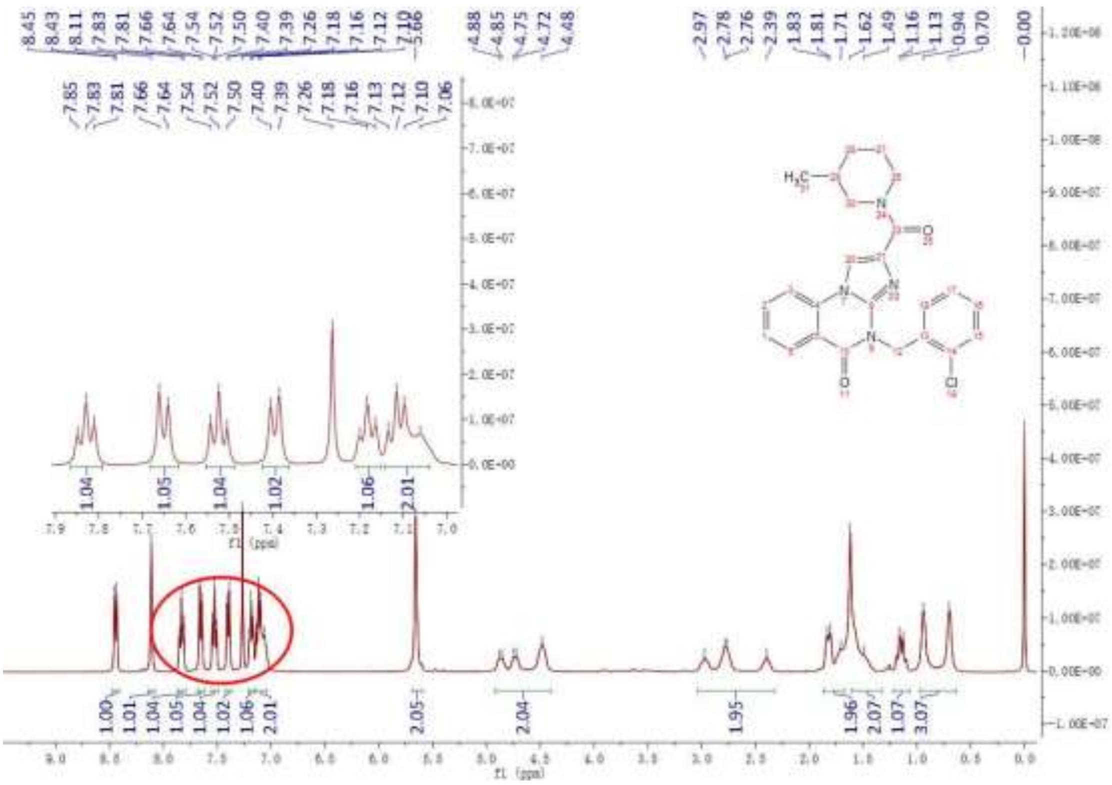

Figure S108. ^{1}H-NMR spectrum of **B13.**

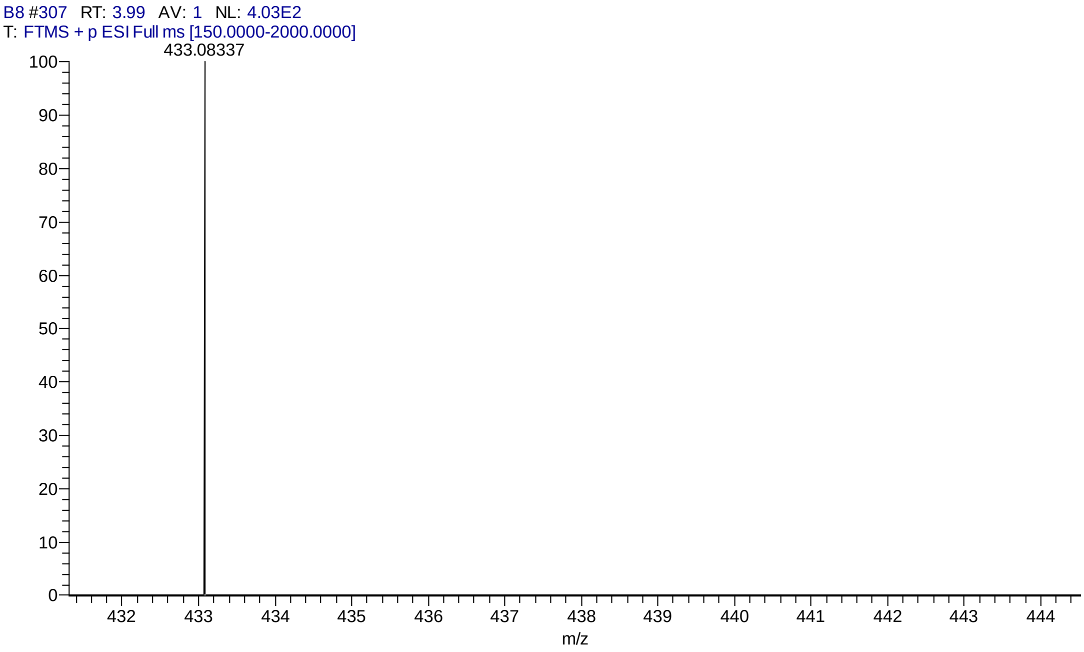

Figure S109. MS spectrum of **B13.**

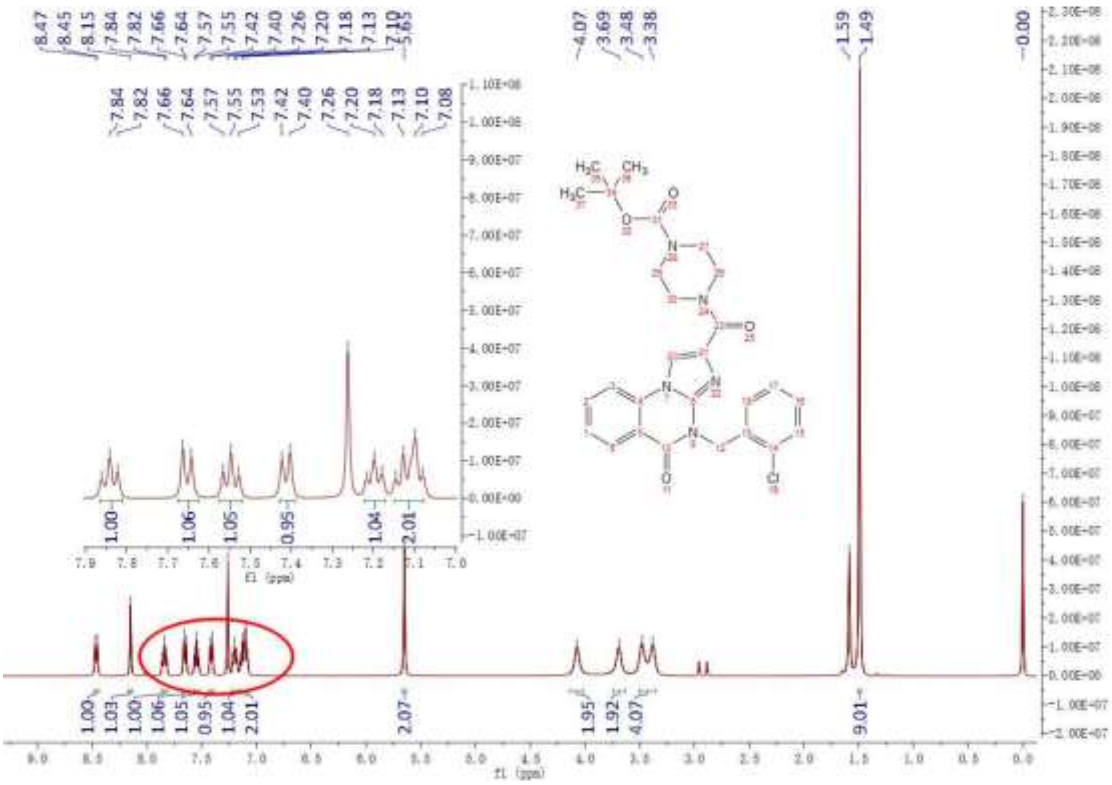

Figure S110. ^{1}H-NMR spectrum of **B14.**

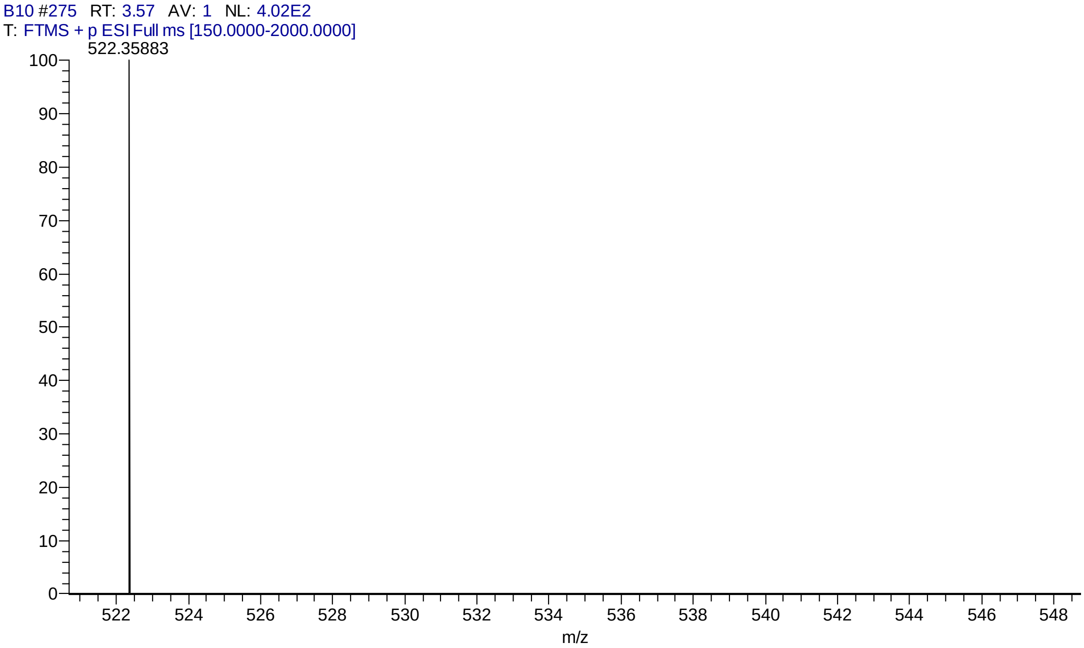

Figure S111. MS spectrum of **B14.**

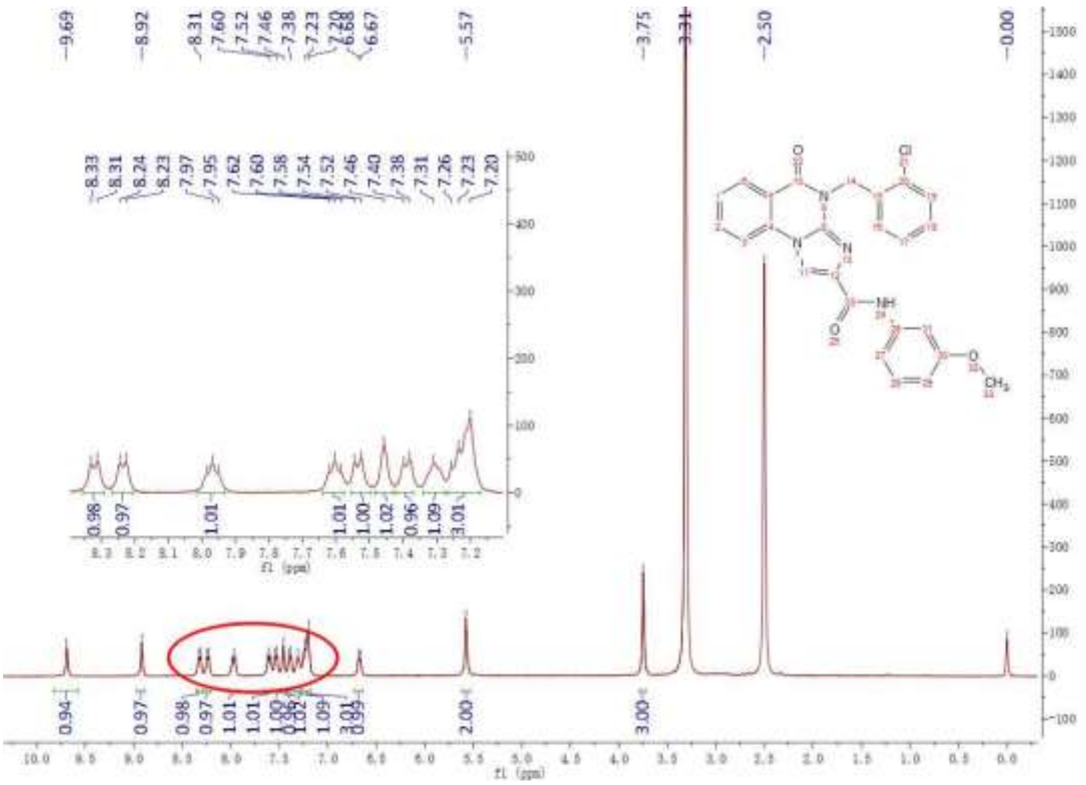

Figure S112. ^{1}H-NMR spectrum of **B15.**

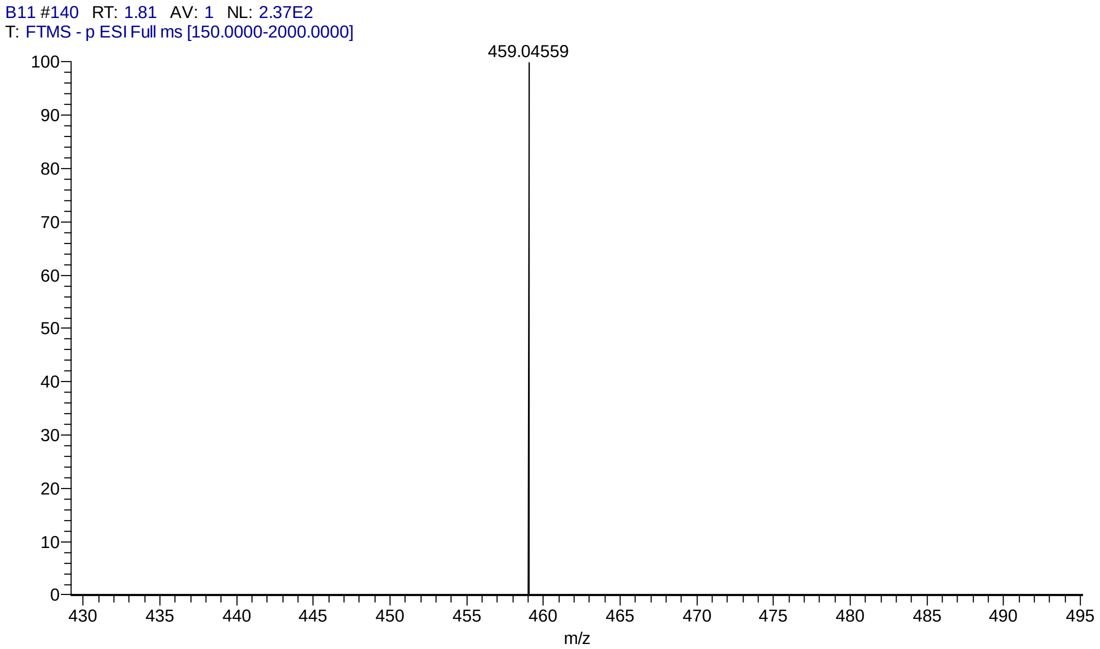

Figure S113. MS spectrum of **B15.**

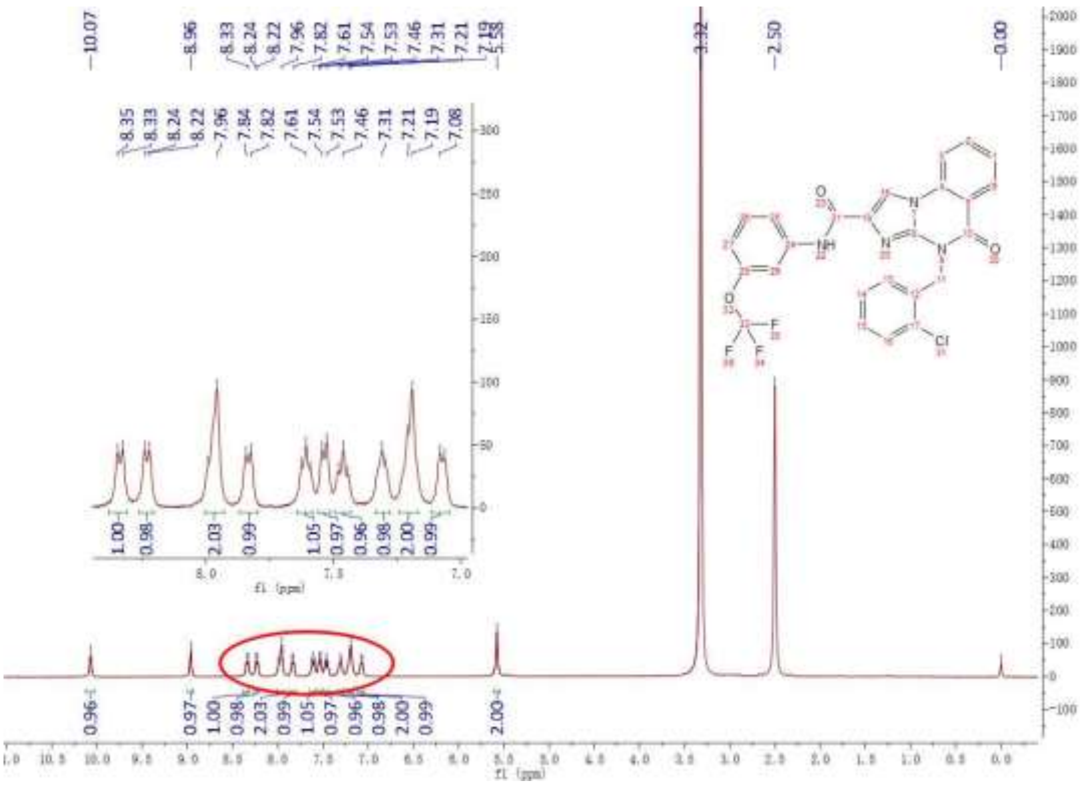

Figure S114. ^{1}H-NMR spectrum of **B16.**

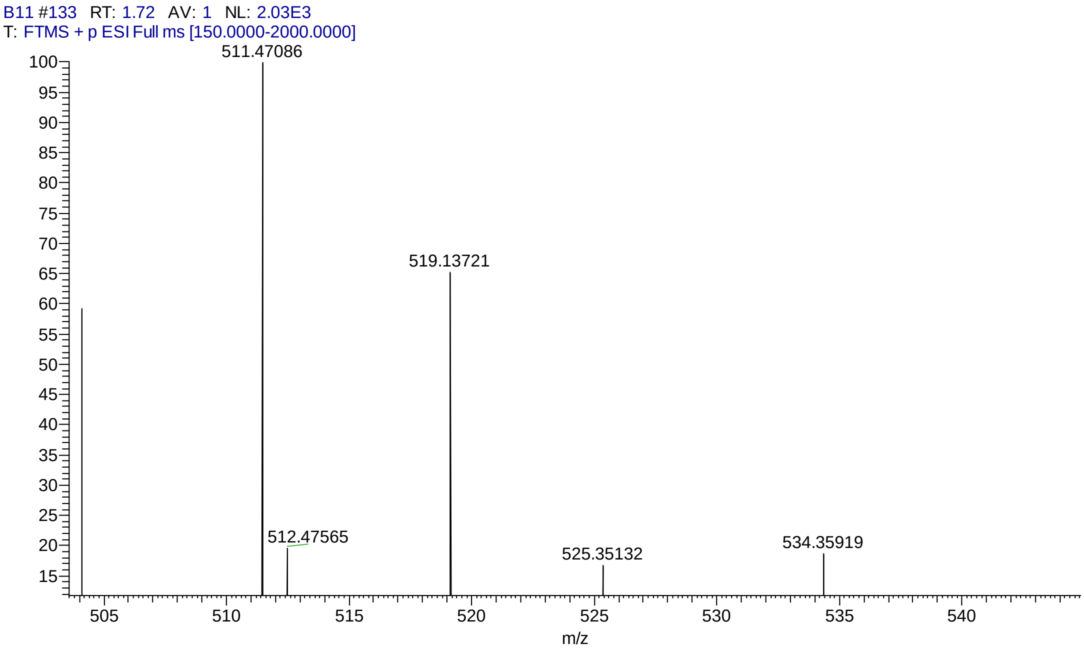

Figure S115. MS spectrum of **B16.**

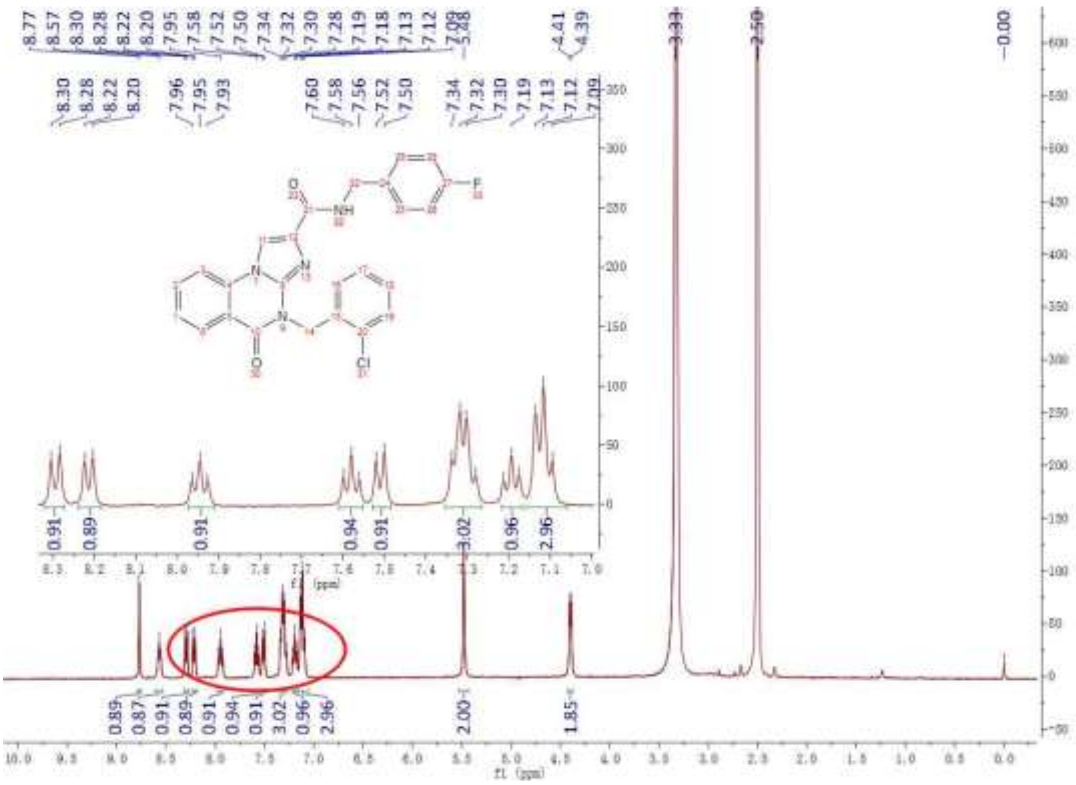

Figure S116. ^{1}H-NMR spectrum of **B17.**

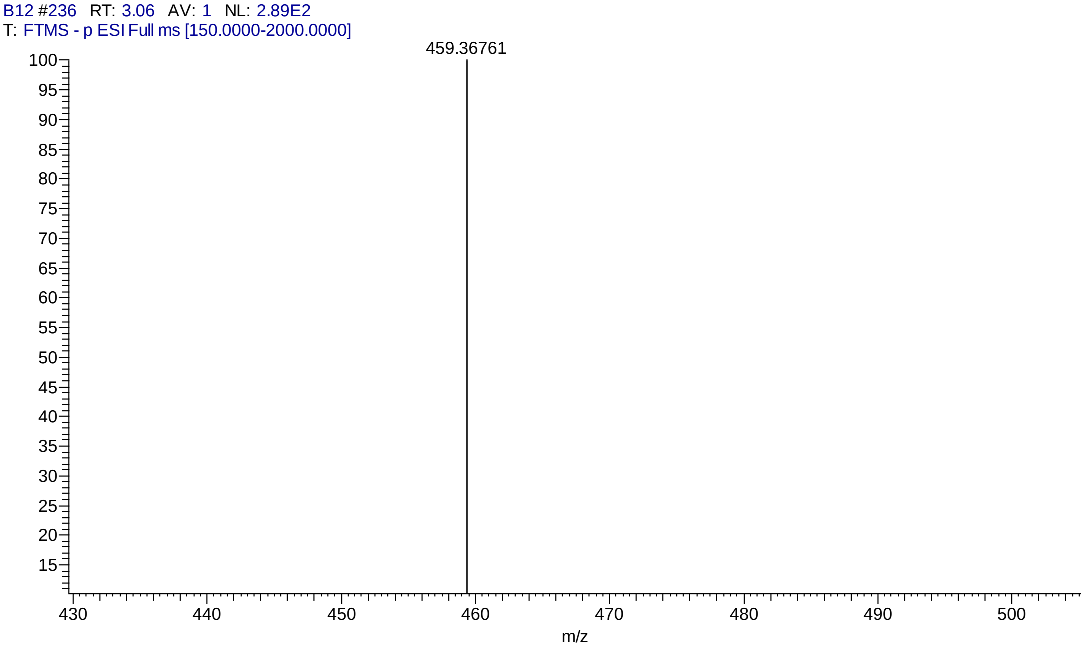

Figure S117. MS spectrum of **B17.**

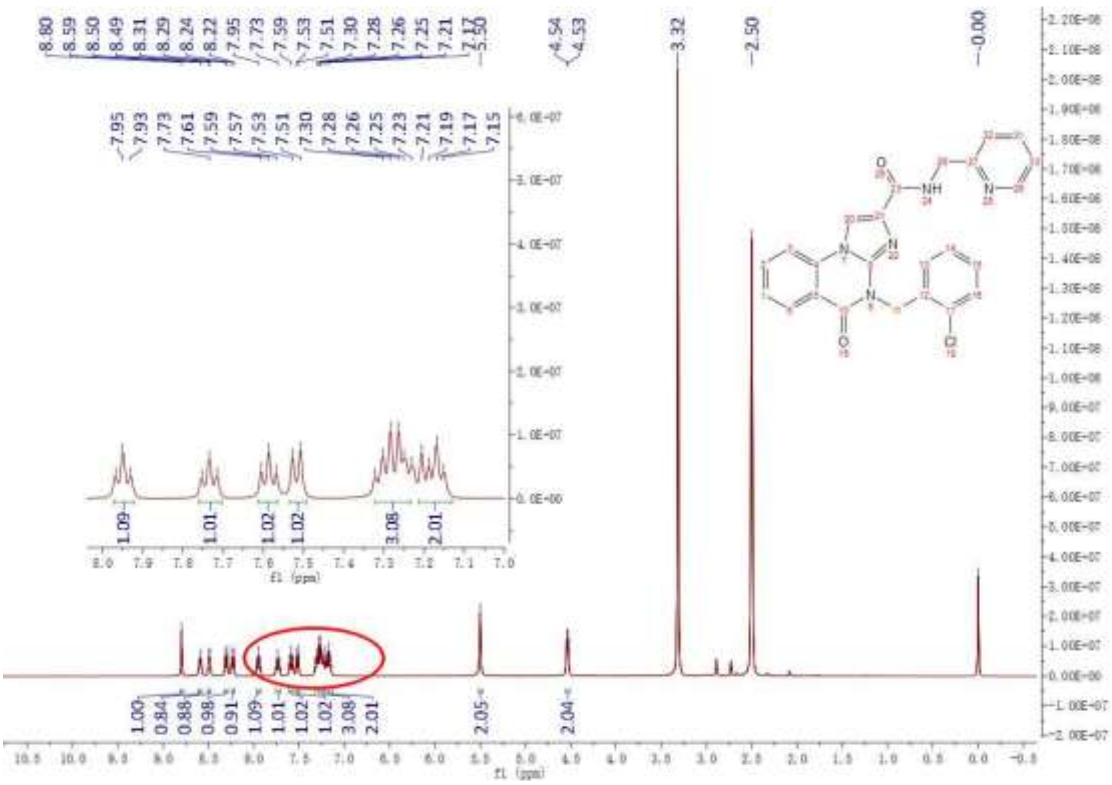

Figure S118. ^{1}H-NMR spectrum of **B18.**

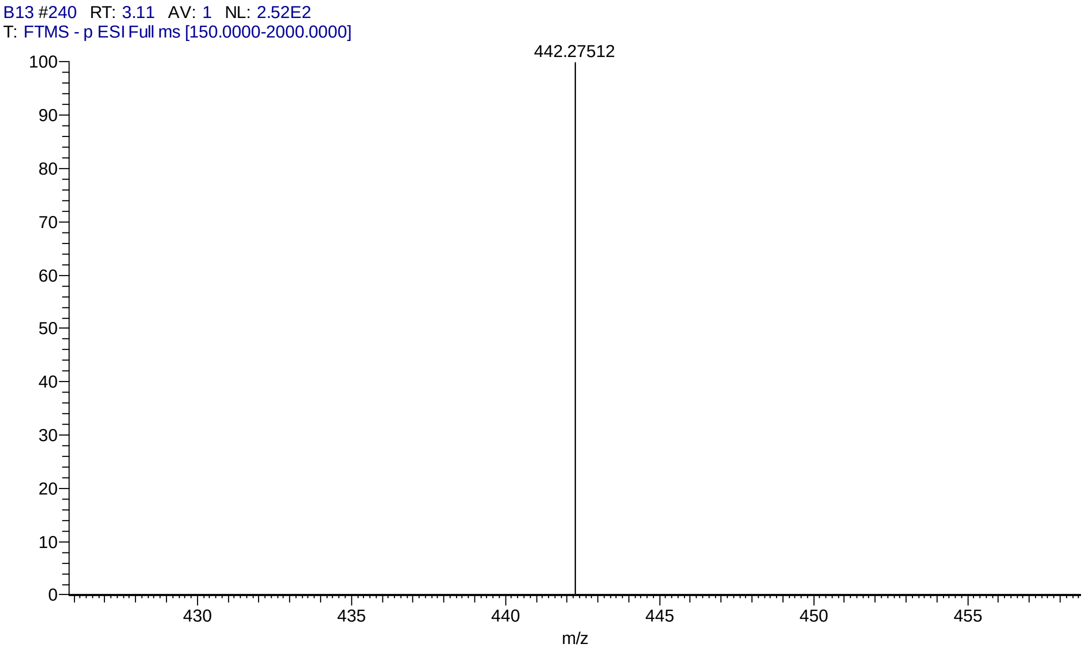

Figure S119. MS spectrum of **B18.**

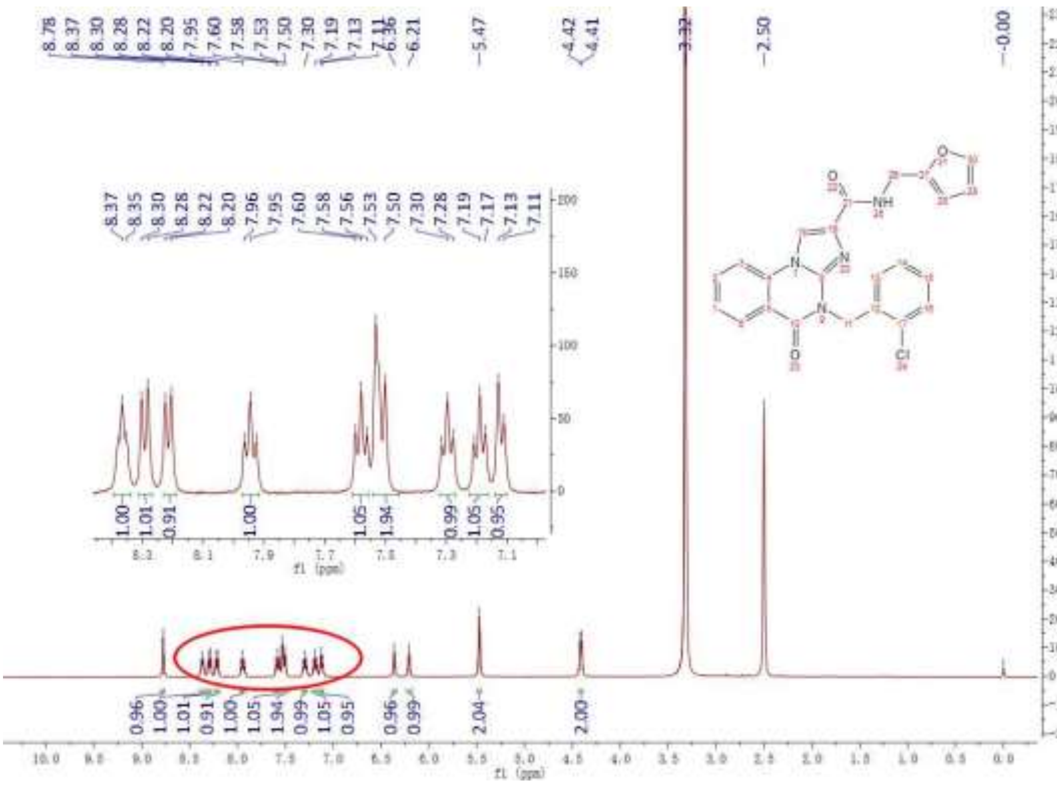

Figure S120. ^{1}H-NMR spectrum of **B19.**

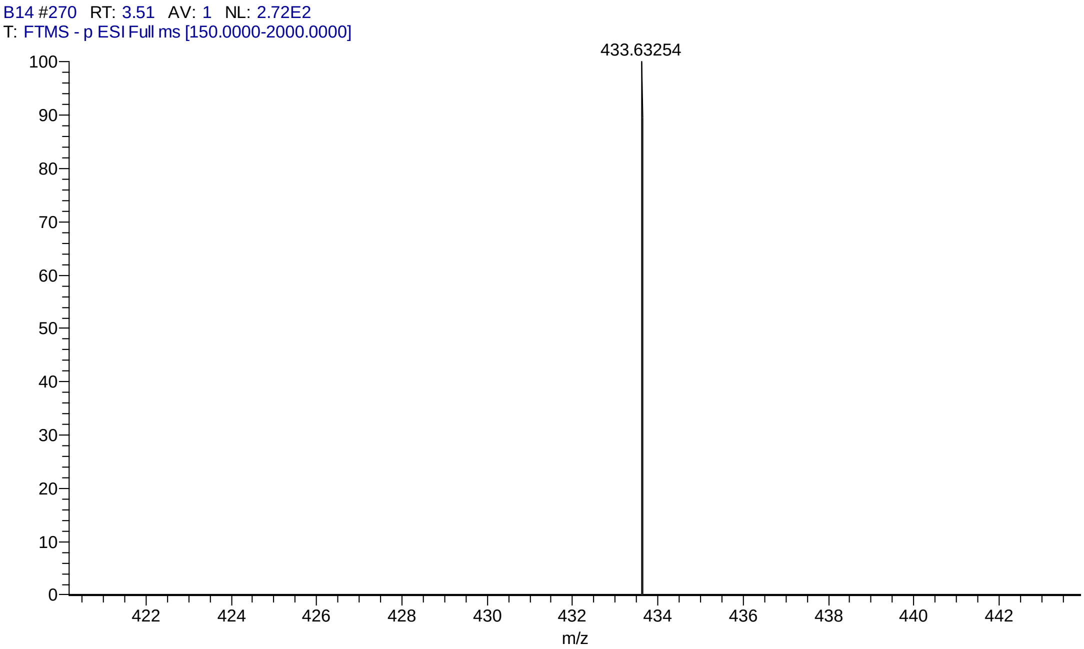

Figure S121. MS spectrum of **B19.**

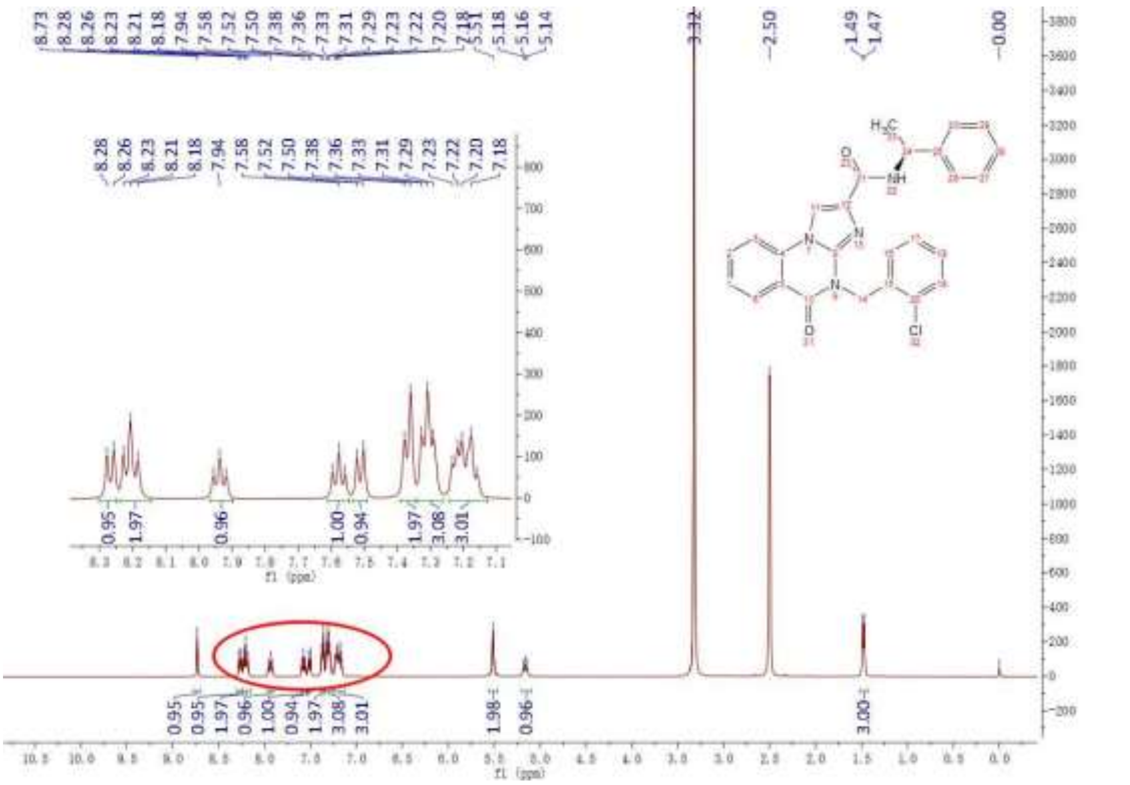

Figure S122. ^{1}H-NMR spectrum of **B20.**

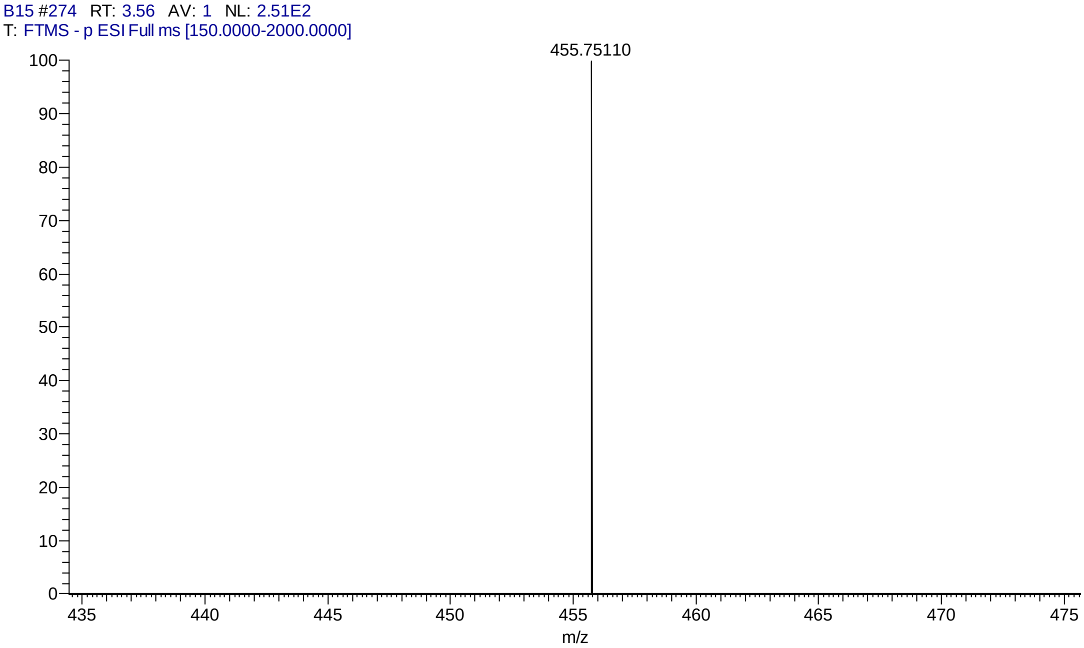

Figure S123. MS spectrum of **B20.**

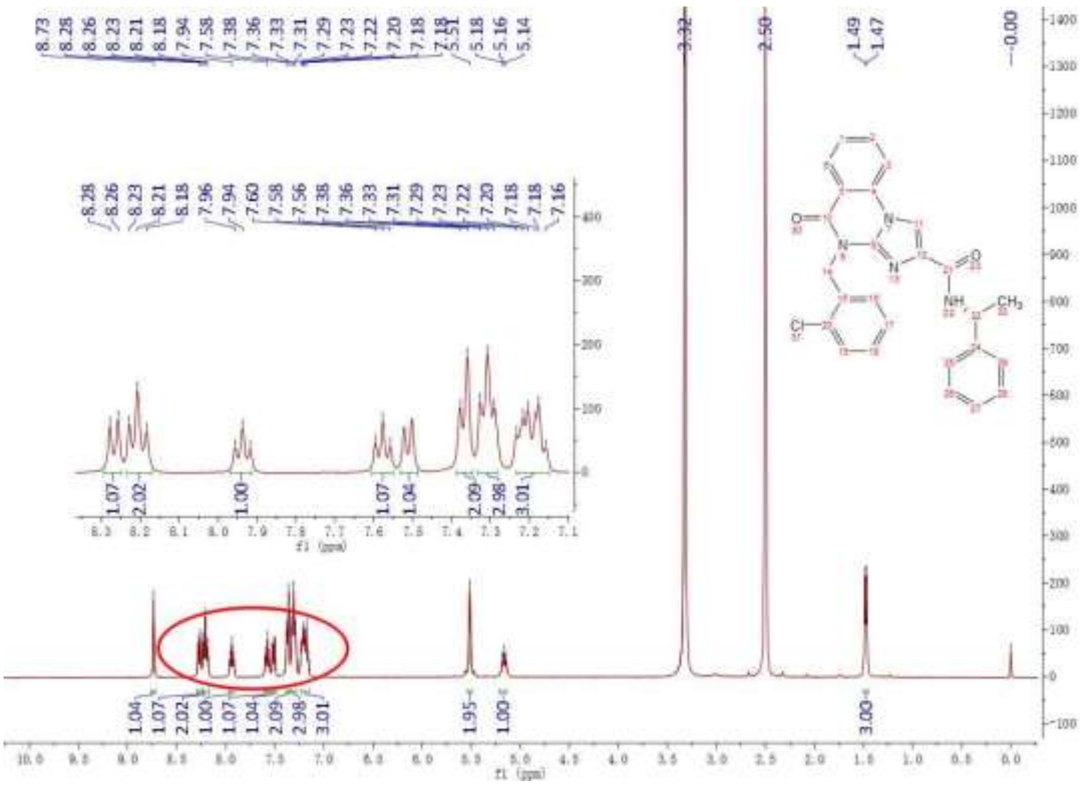

Figure S124. ^{1}H-NMR spectrum of **B21.**

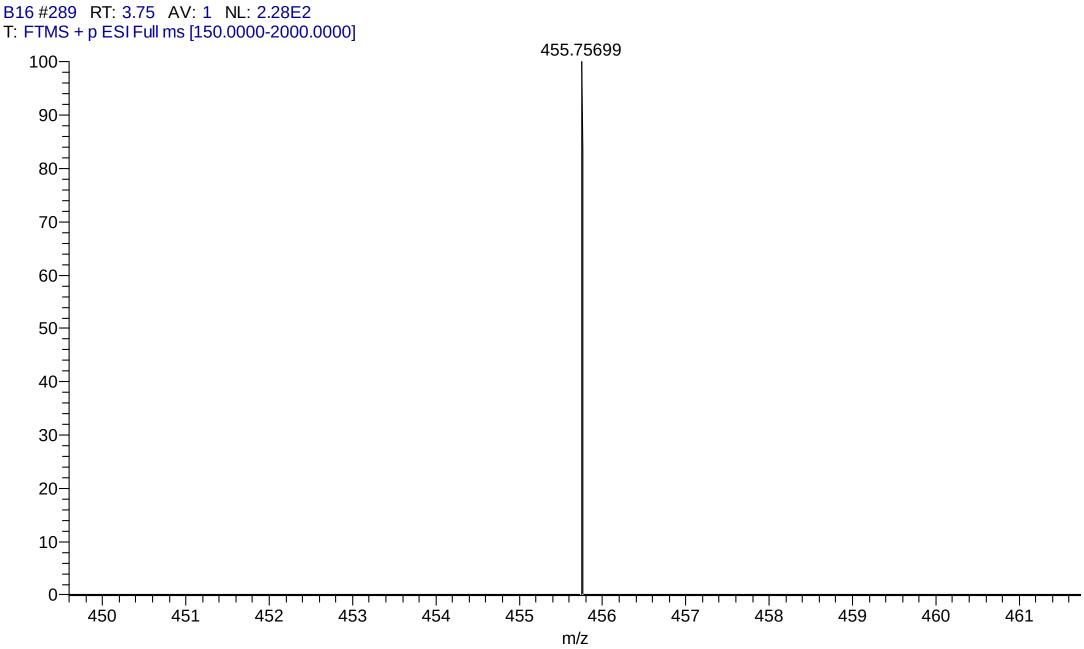

Figure S125. MS spectrum of **B21.**

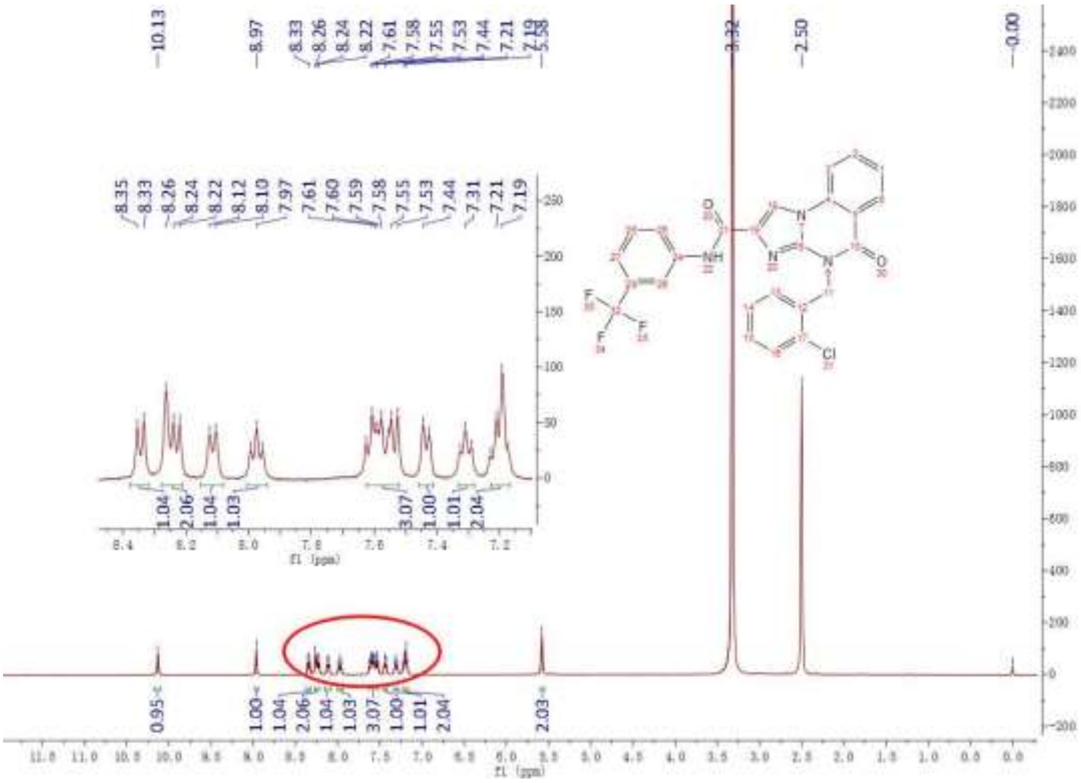

Figure S126. ^{1}H-NMR spectrum of **B22.**

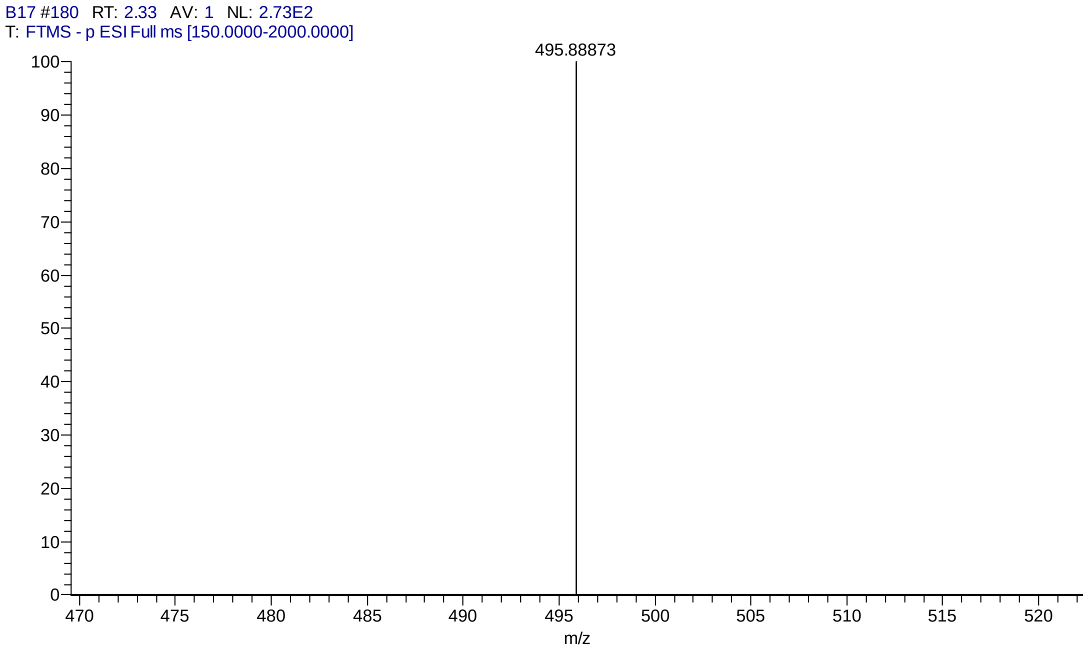

Figure S127. MS spectrum of **B22.**

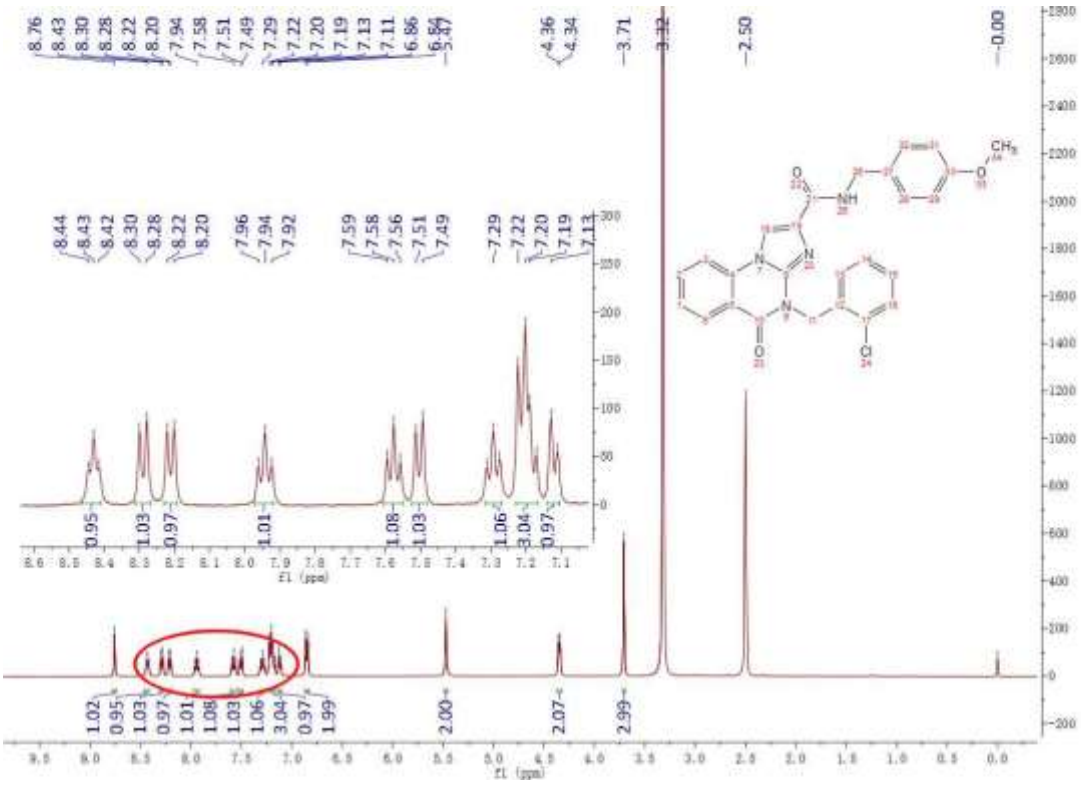

Figure S128. ^{1}H-NMR spectrum of **B23.**

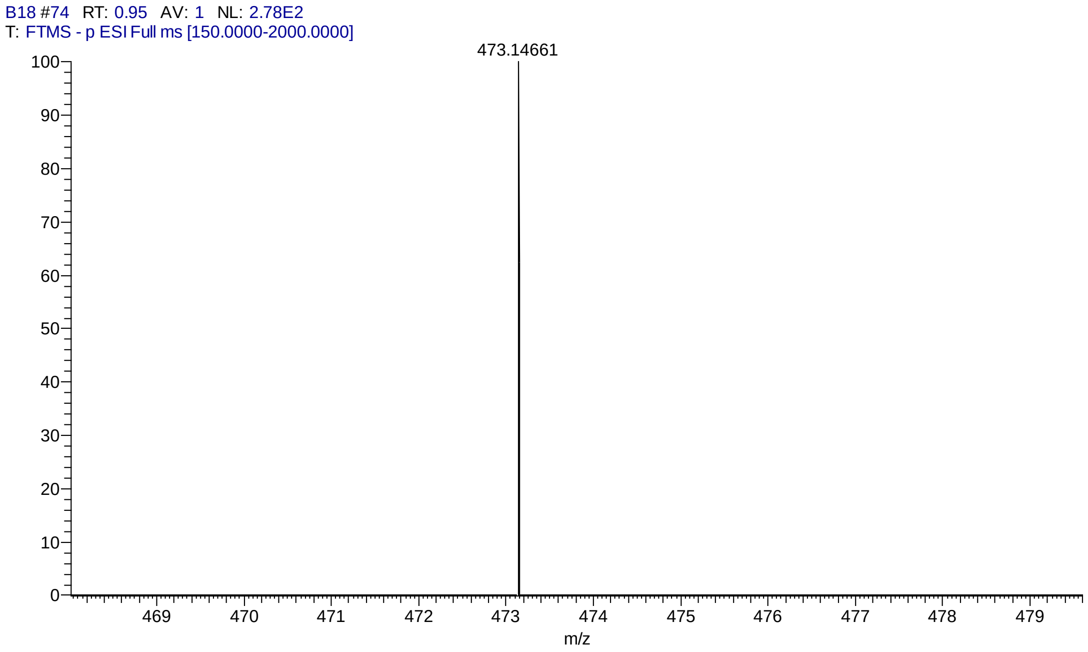

Figure S129. MS spectrum of **B23.**

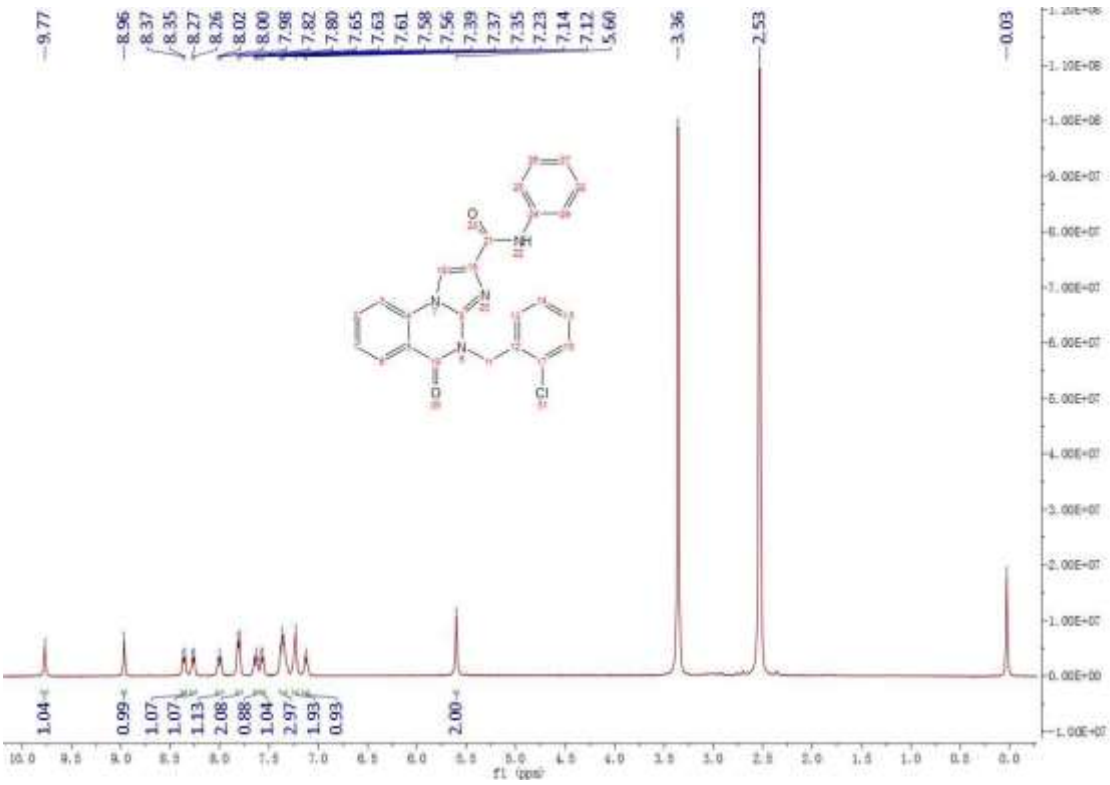

Figure S130. ^{1}H-NMR spectrum of **B24.**

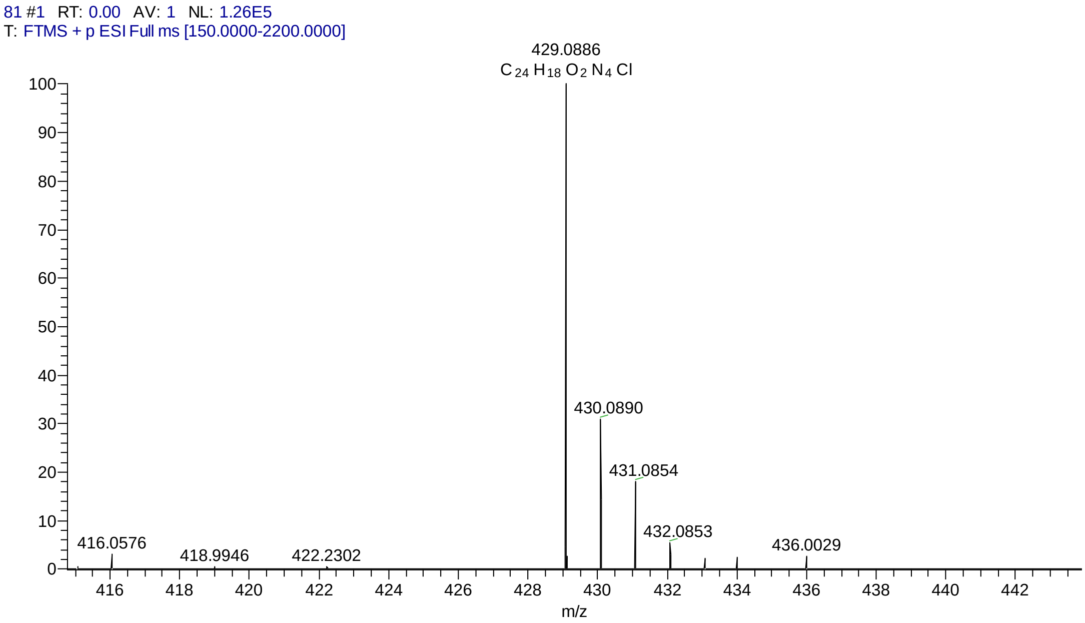

Figure S131. MS spectrum of **B24.**

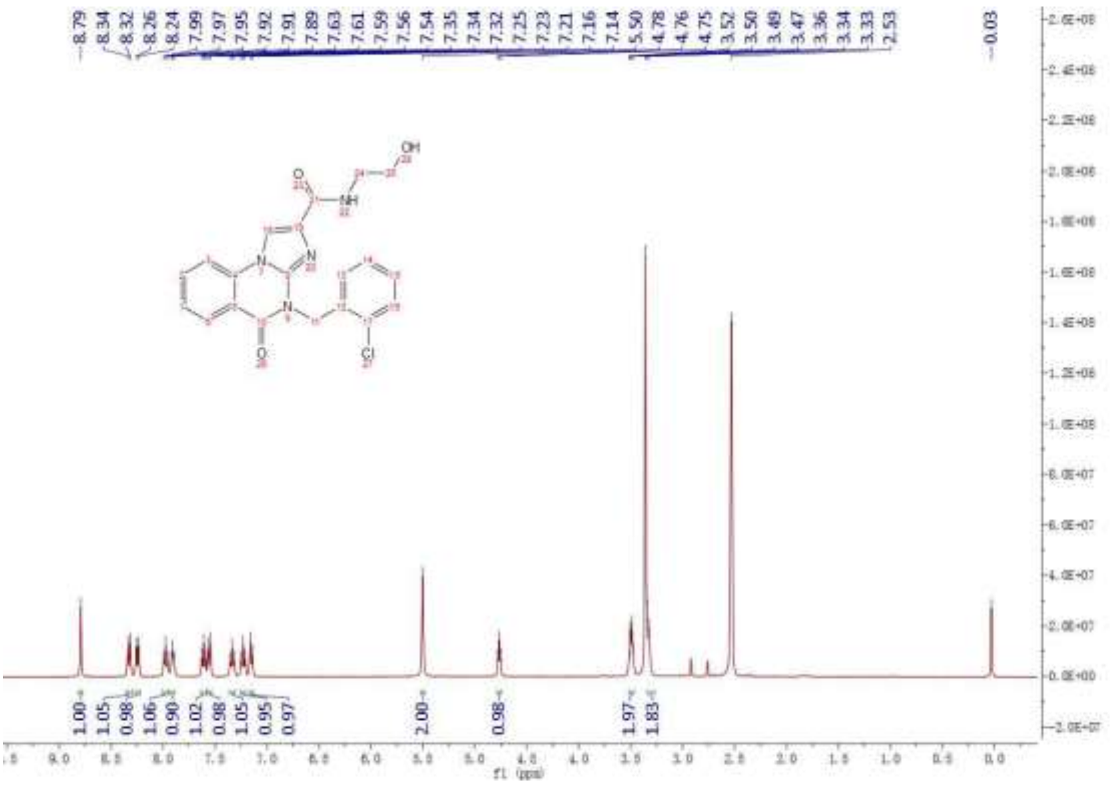

Figure S132. ^{1}H-NMR spectrum of **B25.**

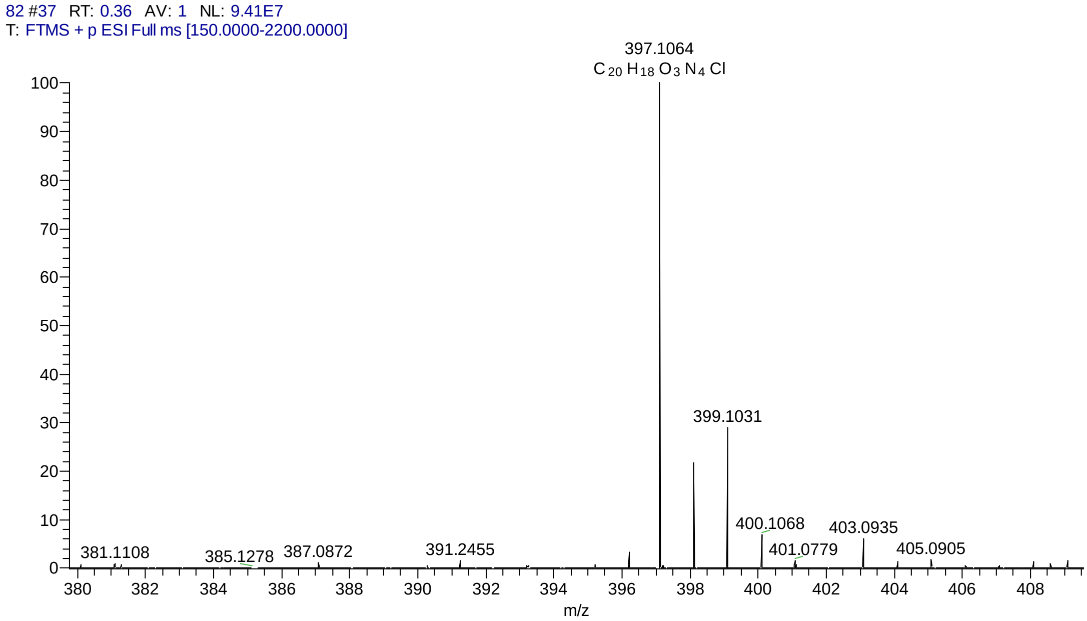

Figure S133. MS spectrum of **B25.**

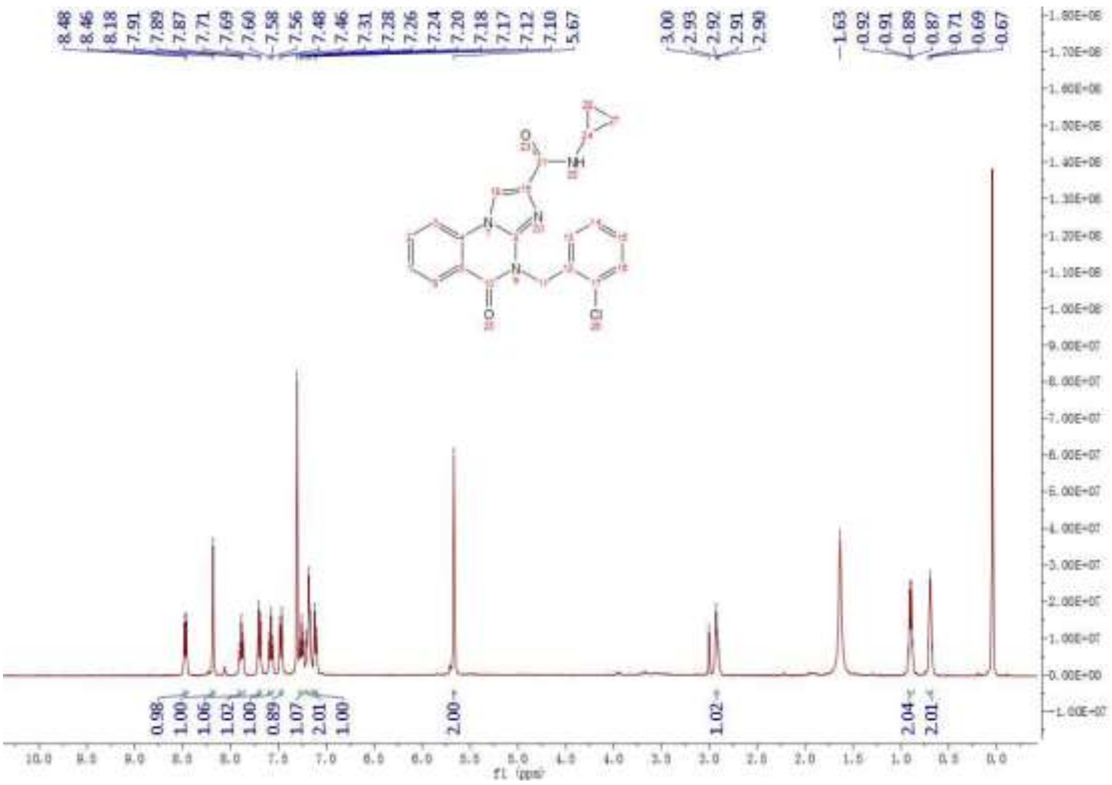

Figure S134. ^{1}H-NMR spectrum of **B26.**

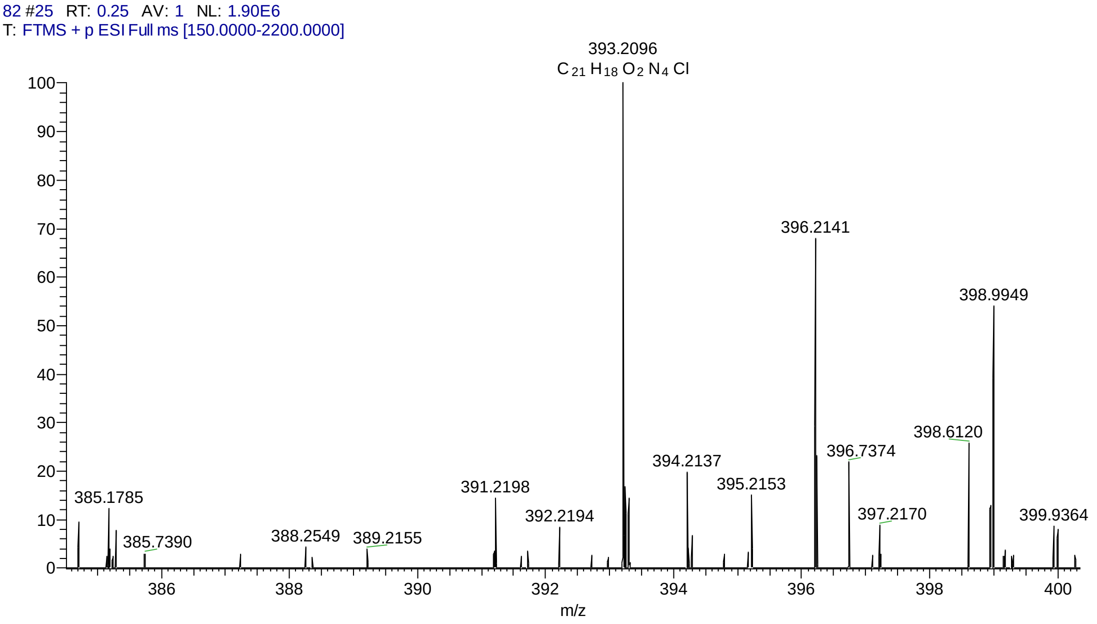

Figure S135. MS spectrum of **B26.**

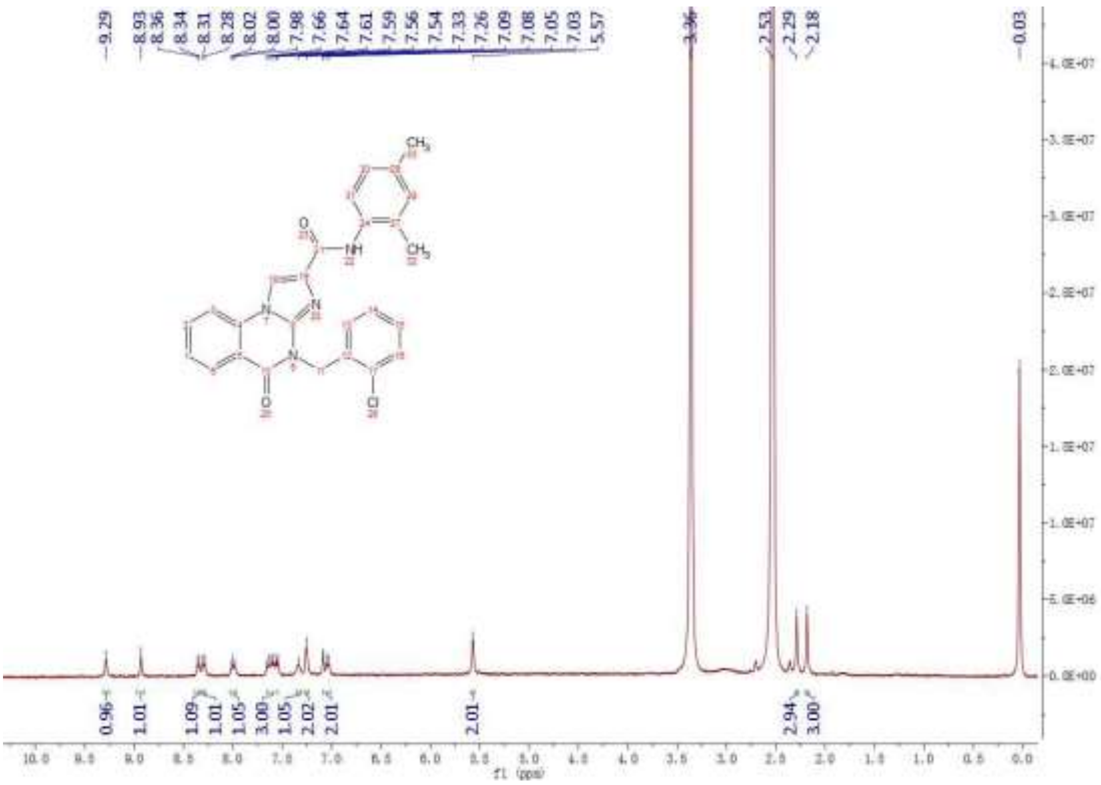

Figure S136. ^{1}H-NMR spectrum of **B27.**

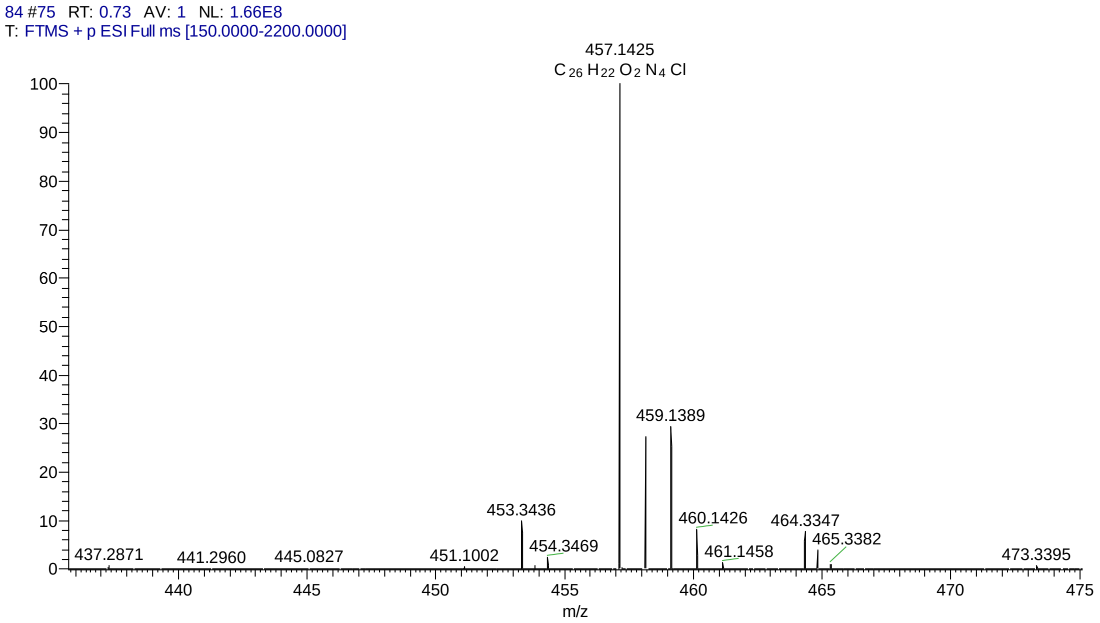

Figure S137. MS spectrum of **B27.**

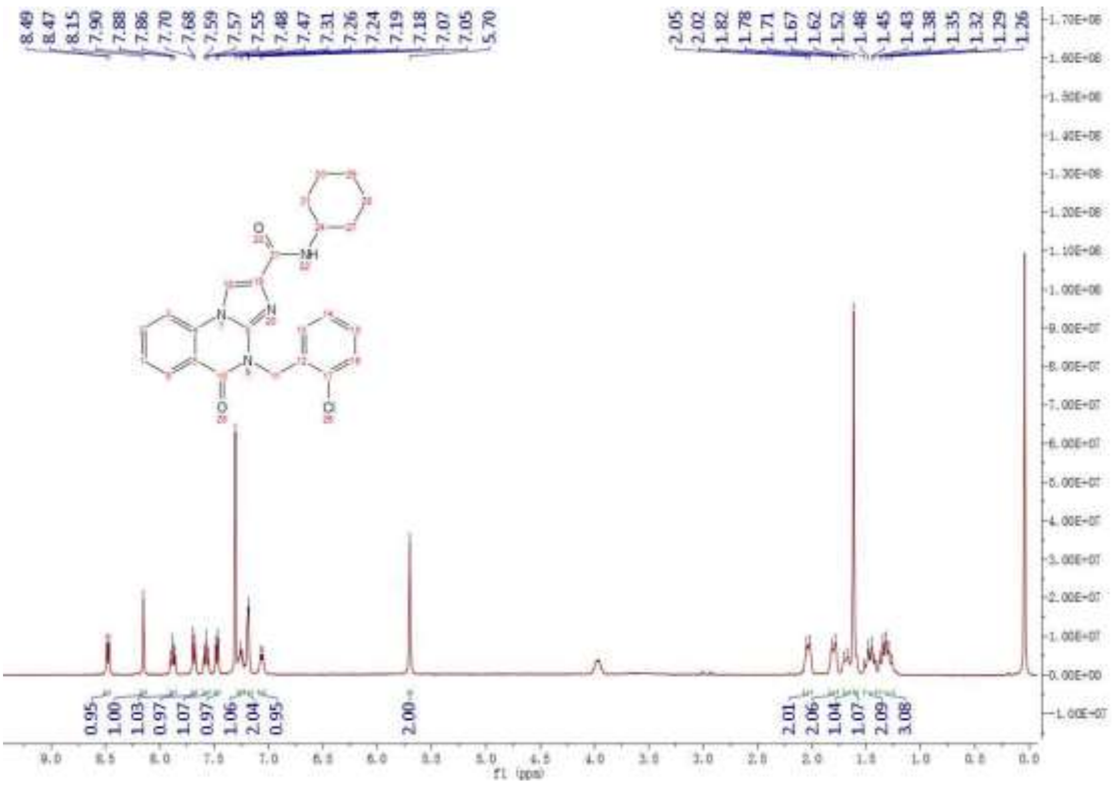

Figure S138. ^{1}H-NMR spectrum of **B28.**

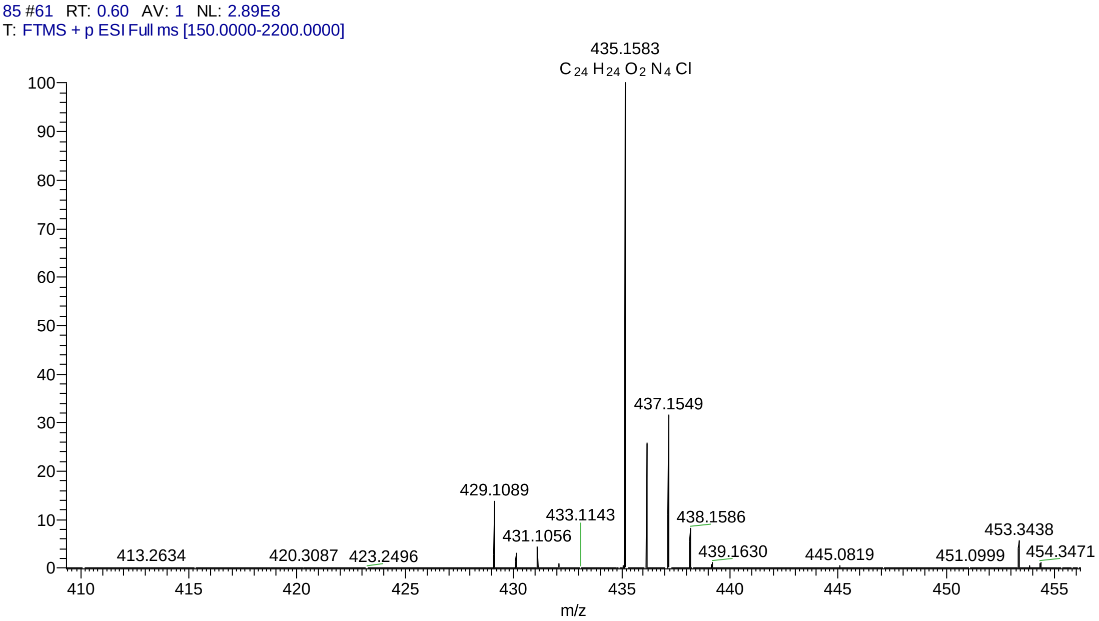

Figure S139. MS spectrum of **B28.**

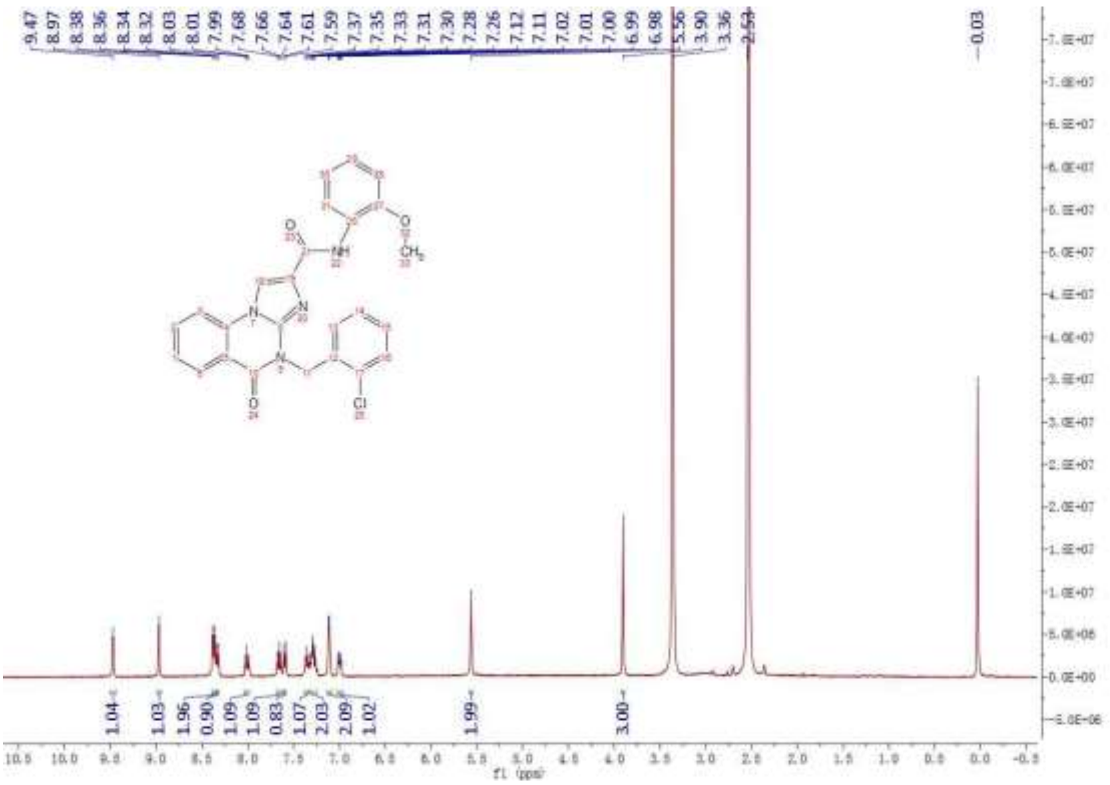

Figure S140. ^{1}H-NMR spectrum of **B29.**

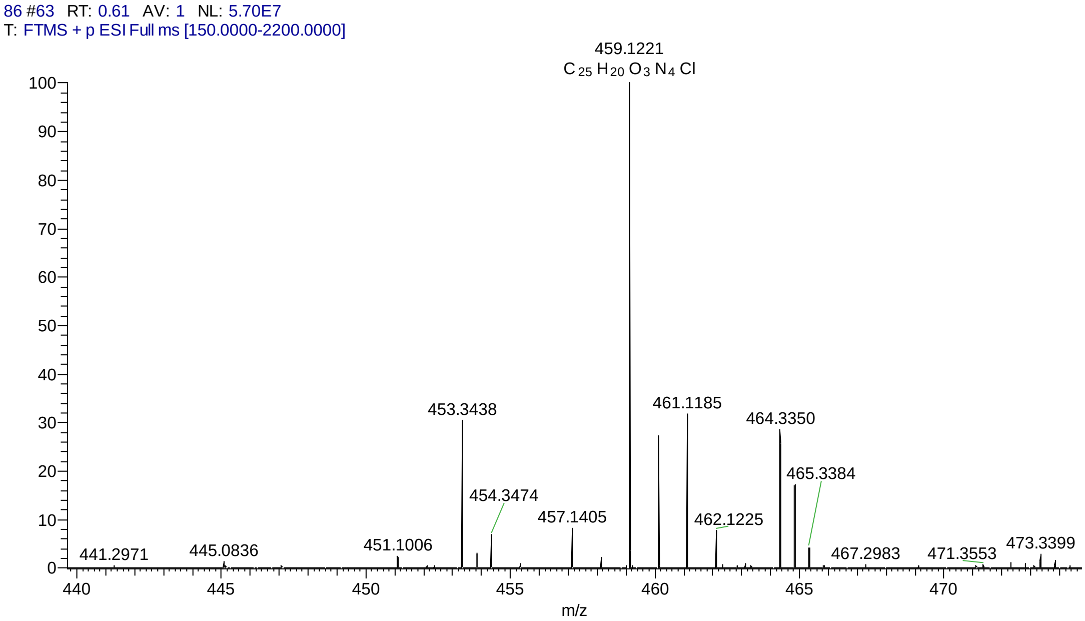

Figure S141. MS spectrum of **B29**.

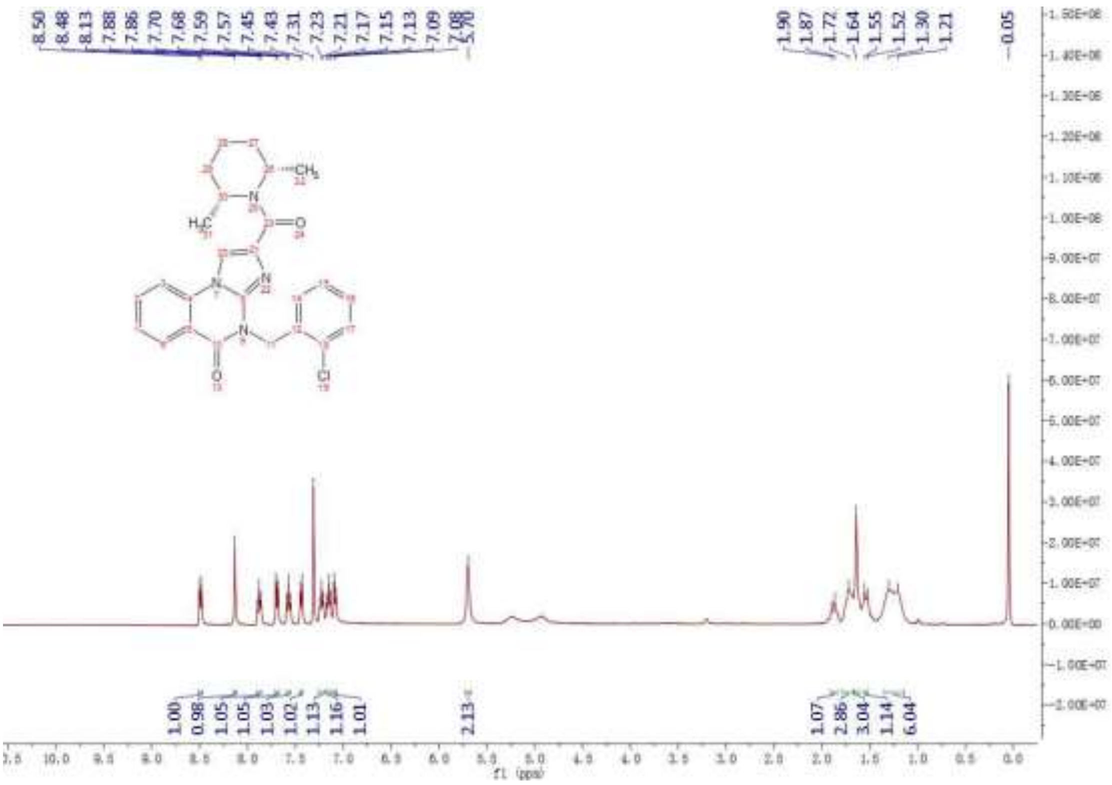

Figure S142. ^{1}H-NMR spectrum of **B30.**

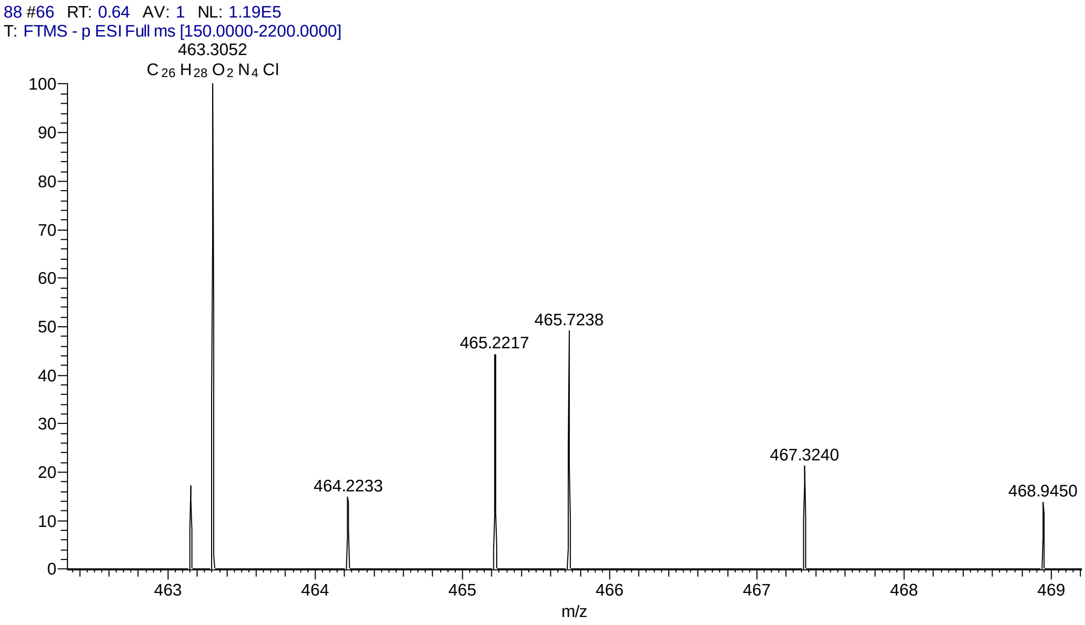

Figure S143. MS spectrum of **B30.**

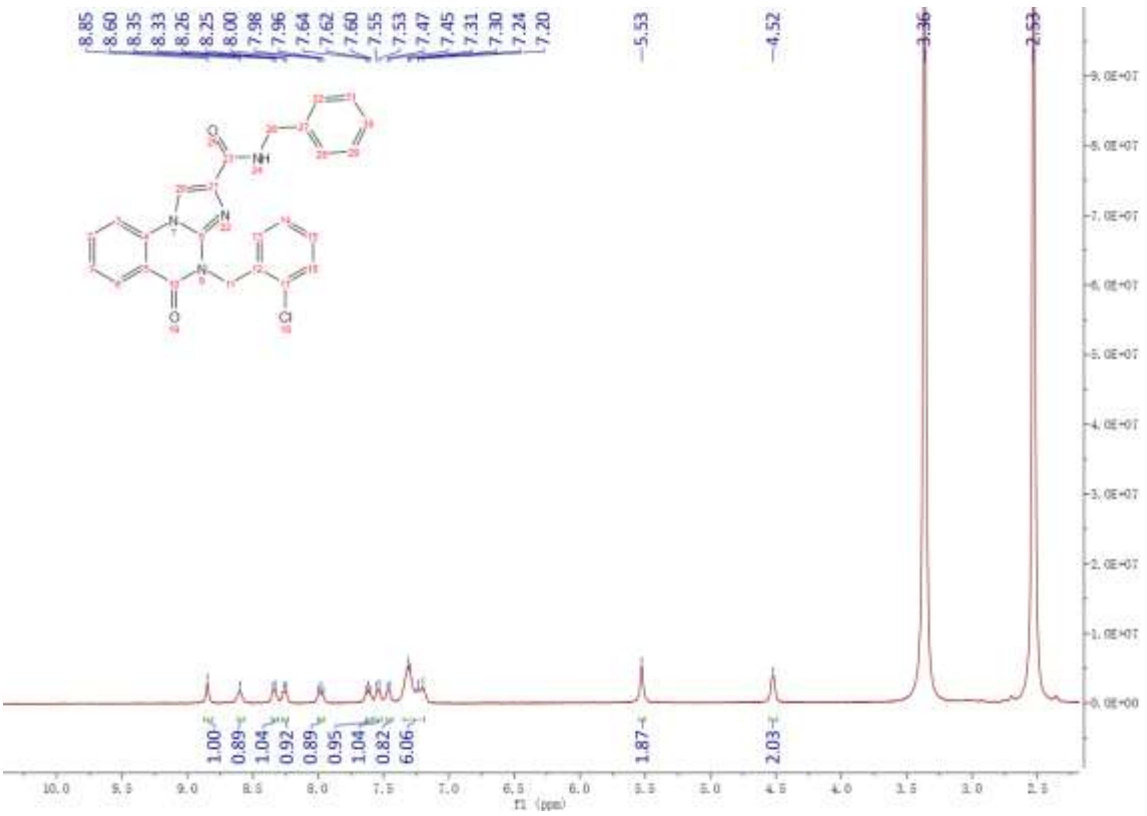

Figure S144. ^{1}H-NMR spectrum of **B31.**

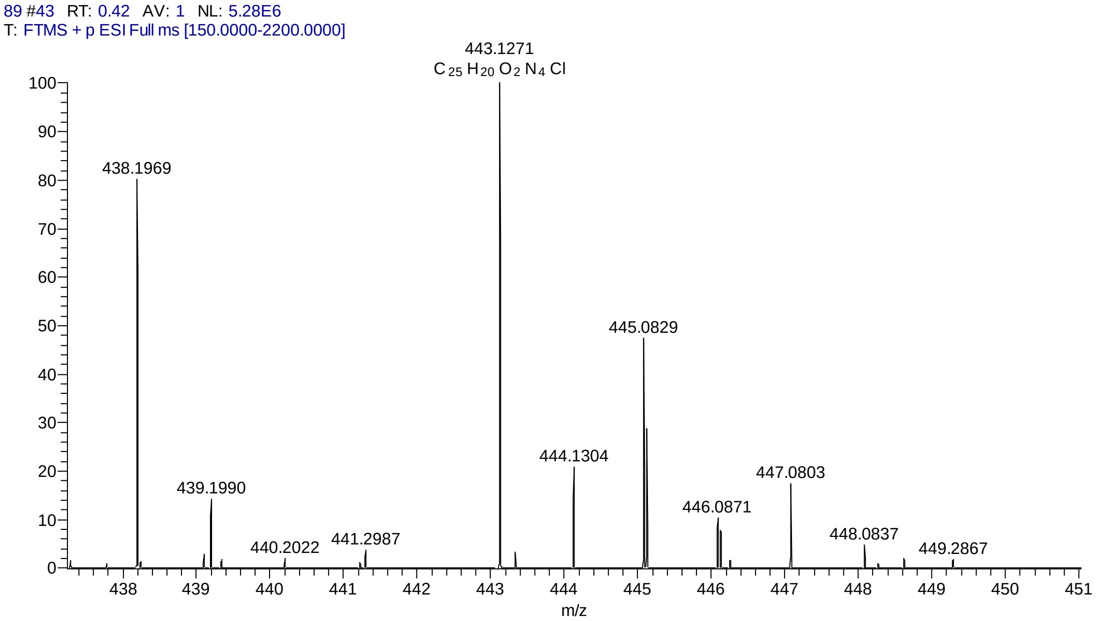

Figure S145. MS spectrum of **B31.**

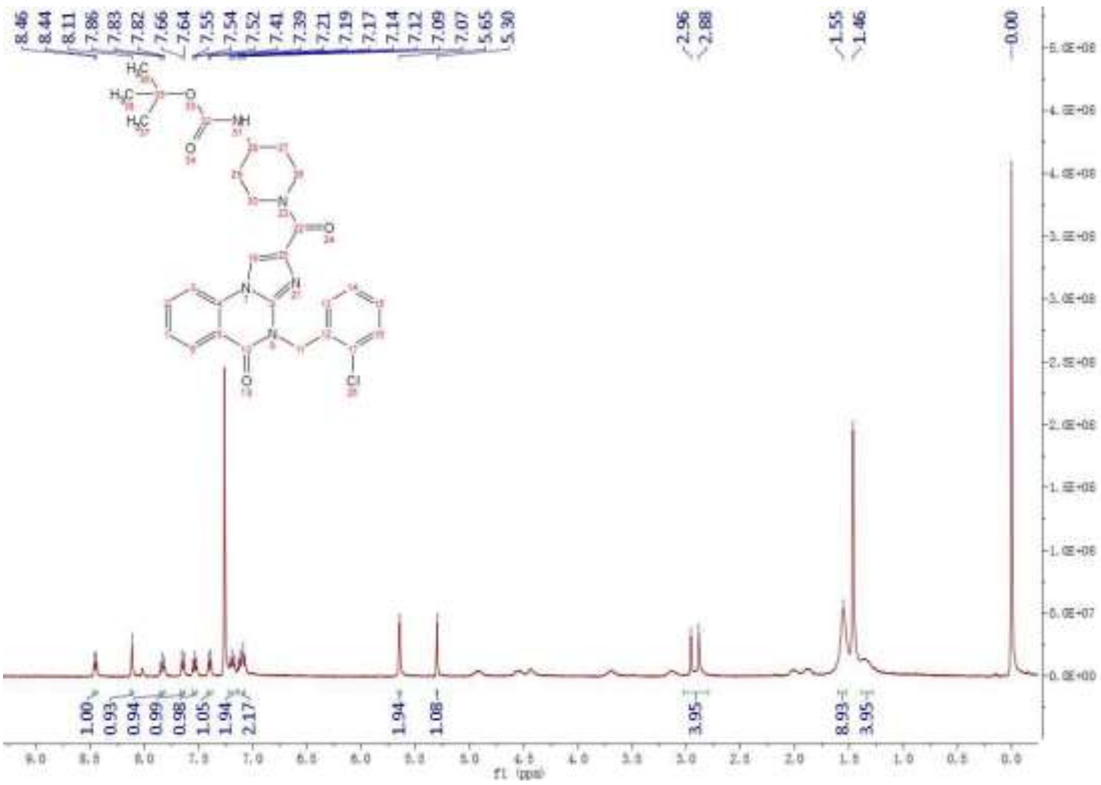

Figure S146. ^{1}H-NMR spectrum of **B32.**

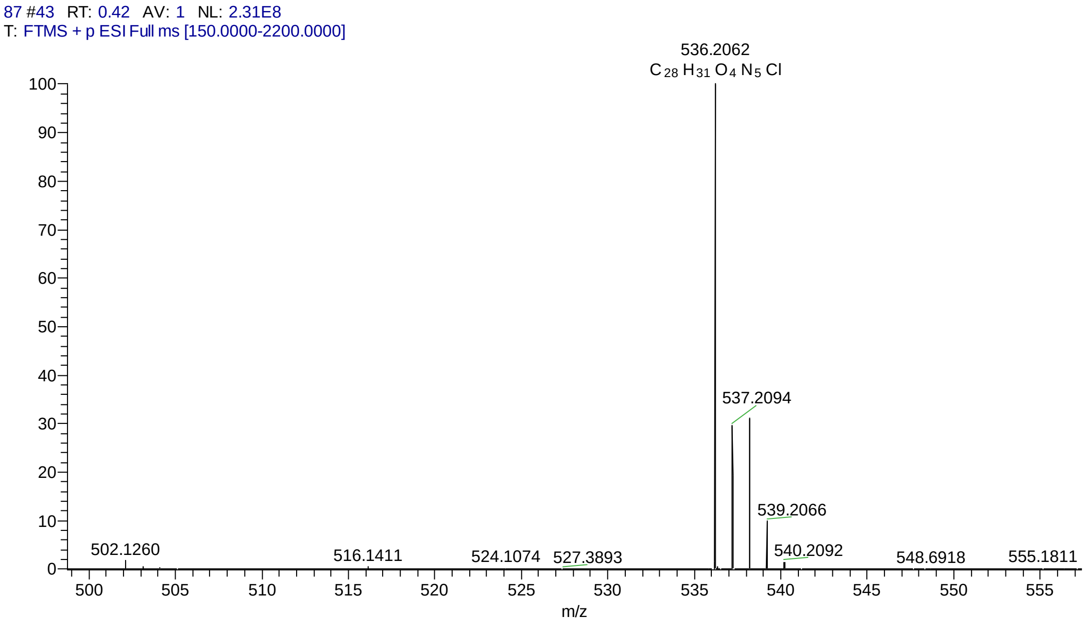

Figure S147. MS spectrum of **B32**.
